# Rapid and Scalable Synthesis of Oxazoles Directly from Carboxylic Acids

**DOI:** 10.1021/acs.joc.4c03166

**Published:** 2025-03-05

**Authors:** Lahu N. Chavan, Gouthami Pashikanti, Mark M. Goodman, Lanny S. Liebeskind

**Affiliations:** † Department of Chemistry, Emory University, 1515 Dickey Drive, Atlanta, Georgia 30322; ‡ Department of Radiology and Imaging Sciences, Wesley Woods Health Center, 1841 Clifton Rd. NE, second floor, Atlanta, Georgia 30329, United States

## Abstract

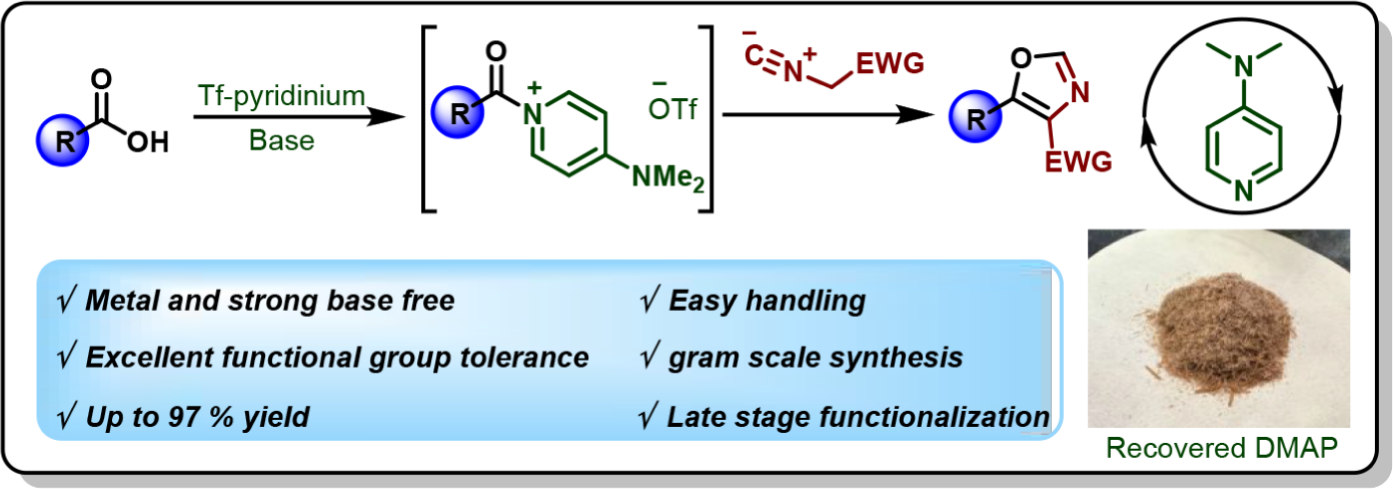

A highly efficient and expedient method for the synthesis of 4,5-disubstituted oxazoles has been developed directly from carboxylic acids, employing a stoichiometric amount of the easy-to-access and stable triflylpyridinium reagent. The overall transformation proceeds through the formation of an *in situ* generated acylpyridinium salt followed by trapping with isocyanoacetates and tosylmethyl isocyanide. This transformation has a broad substrate scope with good functional group tolerance (including hindered and less reactive substrates or those containing sensitive functional groups). The versatility of this newly developed reaction is illustrated through its application in the gram-scale production of the FDA-approved prodrug 5-aminolevulinic acid (5-ALA) and the late-stage functionalization of bioactive molecules including estrone, lipoic acid, valproic acid, and probenecid. Additionally, this process features the advantageous recovery and reuse of the base DMAP, underscoring its practical benefits.

## Introduction

**Scheme 1:**
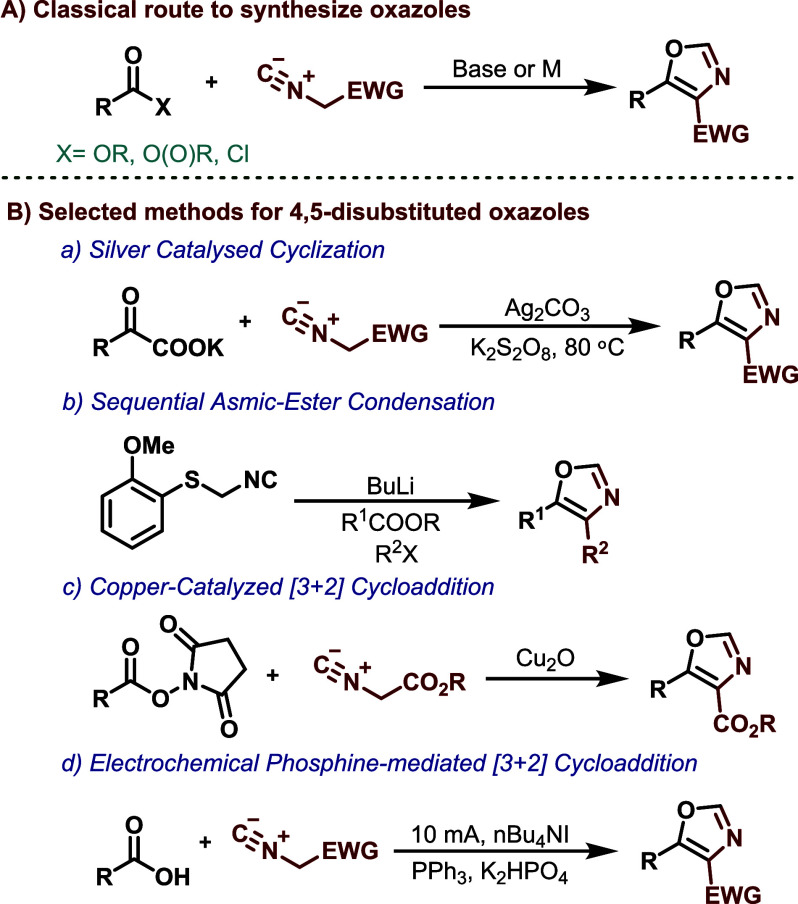
Previous Approaches

The development of synthetic pathways to nitrogen- and oxygen-containing heterocycles from easily accessible starting materials represents an important area of research in organic synthesis.^1^ Oxazoles, characterized by their nitrogen and oxygen components, possess significant pharmacological and biological properties, making them highly desirable target molecules.^2^ Particularly, 4,5-disubstituted oxazole derivatives are widely recognized as privileged building blocks for numerous natural products and bioactive molecules.^3,4^ Consequently, considerable attention has been focused on devising useful approaches for their synthesis.^5^ The most commonly employed method for obtaining 4,5-disubstituted oxazoles involves the reaction between activated carboxylic acid derivatives (such as acid chlorides, anhydrides, and esters) and activated methyl isocyanides. Most reported reactions utilize pregenerated activated carboxylic acid derivatives, sometimes requiring strong bases and corrosive reagents (Scheme 1A).^6^

In 2015, Lie and colleagues introduced a method for synthesizing oxazoles through silver-catalyzed oxidative decarboxylation of α-oxocarboxylates and cyclization with isocyanides (Scheme 1a).^7^ Mueller and Fleming proposed a sequential process for the synthesis of substituted oxazoles, the process involving the condensation of deprotonated anisyl sulfanyl methyl isocyanide (Asmic) with esters, followed by a sulfur–lithium exchange and trapping with electrophiles to yield C-4 substituted oxazoles, as depicted in Scheme 1b. It should be noted that this method necessitates the use of a strong base (*n*-BuLi) and an additional step.^8^ In 2022, Wu and coworkers reported a copper-catalyzed reaction using redox-active *N*-hydroxysuccinimide esters with isocyanoacetates^8b^ to yield corresponding 4,5-disubstituted oxazoles^9^ as shown in Scheme 1c.

Despite extensive efforts to synthesize oxazoles, very few reports in the literature detail a direct transformation from carboxylic acids to oxazoles in a single step.^10^ Carboxylic acids are versatile, easily accessible, and stable components in organic synthesis.^11^ They have been widely used in the creation of various heterocyclic compounds. Recently, Xia and others developed an electrochemical deoxygenative reaction to synthesize oxazoles directly from carboxylic acids (Scheme 1d).^12^ However, this approach required more than stoichiometric amounts of triphenylphosphine (PPh_3_) as a deoxygenation reagent, generating a stoichiometric quantity of triphenylphosphine oxide and posing difficulties in reaction workup and product purification processes, leading to significant waste generation.

Hence, the quest for a straightforward and efficient method to access 4,5-disubstituted oxazoles directly from readily available carboxylic acids remains a useful research objective. The reaction will involve the nucleophilic attack of a deprotonated, activated alkyl isocyanide group on an activated acyl electrophile. Consequently, achieving chemoselectivity in the *in situ* activation of carboxylic acids becomes a crucial consideration in selecting activating reagents. Recently, Xu and Li demonstrated the utility of the triflylpyridinium reagent, DMAP-Tf, a stable and easily accessible reagent for the *in situ* activation of carboxylic acid^13^ through an intermediate acylpyridinium species. We explored this *in situ* generated reactive acylpyridinium intermediate reacting with isocyanoacetate and related derivatives for the synthesis of 4,5-disubstituted oxazoles.

## Results and Discussion

We initially evaluated the reactivity of commercially accessible 3-fluorobenzoic acid (**1a**) when combined with ethyl isocyanoacetate **2a** (1.2 equiv). DMAP-Tf (1.3 equiv) was used as a carboxylic acid activator in CH_2_Cl_2_ at room temperature. Initial attempts to perform the reaction without a base did not provide the desired product. Further experiments using NEt_3_ or DIPEA (1.3 equiv) as bases at different temperatures likewise failed to produce oxazole **3aa**. However, the use of DABCO as the base led to oxazole **3aa** in a moderate yield of 47% in 60 min at room temperature (Table 1, entry 3). Significantly, when DBU was utilized under comparable circumstances, only trace amounts of oxazole product **3aa** were detected (Table 1, entry 7). However, DMAP as a base significantly increased the yield of oxazole product **3aa** to 70% at room temperature within 60 min (Table 1, entry 5). Increasing the amount of base from 1.3 to 1.5 equiv and increasing the reaction temperature to 40 °C produced an excellent (96%) yield of oxazole **3aa** in 30 min (Table 1, entry 5). In an effort to further enhance the reaction efficiency, the effect of various solvents was explored. At 40 °C using 1.5 equiv of DMAP as base, solvents such as DMSO, THF, 1,4-dioxane, and MeCN did not yield better results than CH_2_Cl_2_ (Table 1, entries 9–12).

**Table 1:**
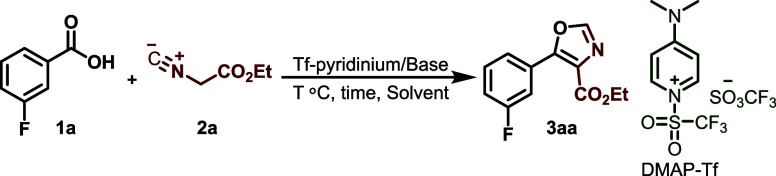
Optimization Table^a^^,^^b^

Entry	Base (equiv)	Solvents	Time (m)	T (°C)	Yield (%)^b^^b^
1	Et_3_N (1.2)	DCM	30	rt	ND
2	Et_3_N (1.2)	DCM	60	40	ND
3	DABCO (1.2)	DCM	30	rt	40
4	-	DCM	30	40	ND
5	DMAP (1.2)	DCM	60	rt	70
6	DMAP (1.5)	DCM	30	40	96
7	DBU (1.2)	DCM	30	40	trace
8	DIPEA (1.2)	DCM	30	40	ND
9	DMAP (1.5)	DMSO	30	40	ND
10	DMAP (1.5)	Dioxane	30	40	37
11	DMAP (1.5)	THF	30	40	40
12	DMAP (1.5)	MeCN	30	40	ND

a Standard reaction conditions: **1a** (1.0 equiv), **2a** (1.2 equiv), DMAP-Tf (1.3 equiv), and base (1.5 equiv) in DCM (0.1 M).

b Yield of the isolated product. ND: not detected.

**Scheme 2:**
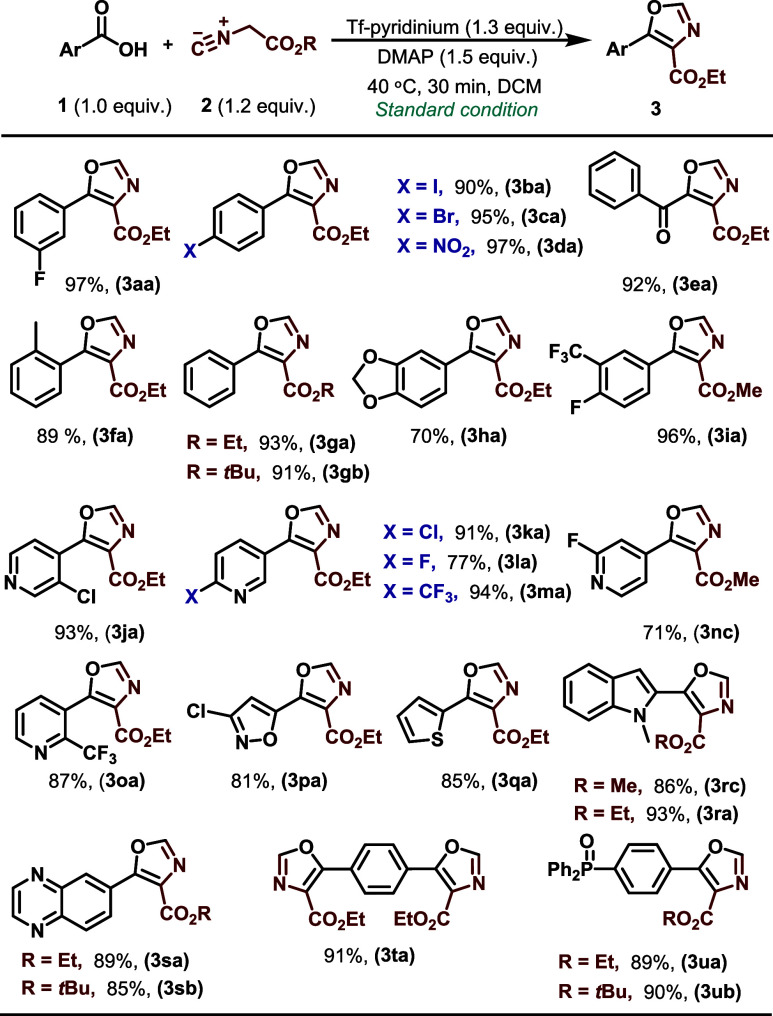
Substrate Scope for Aromatic and Heteroaromatic Carboxylic Acids Standard reaction conditions: **1** (1.0 equiv), **2** (1.2 equiv), DMAP-Tf (1.3 equiv), and base (1.5 equiv) in DCM (0.1 M). Yield of the isolated product. ND: not detected

With optimal reaction conditions in hand, the substrate scope was explored with a variety of substituted aromatic and heteroaromatic carboxylic acids, as shown in Scheme 2. The reaction conditions demonstrated versatility in accommodating a broad range of aromatic and heteroaromatic carboxylic acids, providing the corresponding oxazoles in high yields (70–97%). Ortho, meta, and para substituents were tolerated, as were heteroaromatic substrates such as pyridine, furan, thiophene, isoquinoline, isoxazole, and indole derivatives. The resulting oxazole products **3ja–3sb** were produced with yields of up to 94%. Not surprisingly, the reaction also tolerated halogen substituents on the aromatic rings (F, Cl, Br, and I), presenting valuable prospects for additional functionalization via conventional cross-coupling processes (**3aa-3ca, 3ja**). Moreover, the alpha-oxocarboxylic acid phenyl glyoxylic acid successfully generated the anticipated oxazole **3ea** with a yield of 92%. Replacing an ethyl group with methyl or *tert*-butyl on the isocyanoacetate had no substantial impact on the production of the oxazoles (**3gb** and **3ia,** respectively). Furthermore, diacids such as terephthalic acid exhibited high reactivity in generating the corresponding oxazole (**3ta**). Carboxylic acids bearing a phosphine oxide group nicely survived the reaction conditions, resulting in the anticipated oxazole compounds (**3ua, 3ub**) in high yields.

**Scheme 3:**
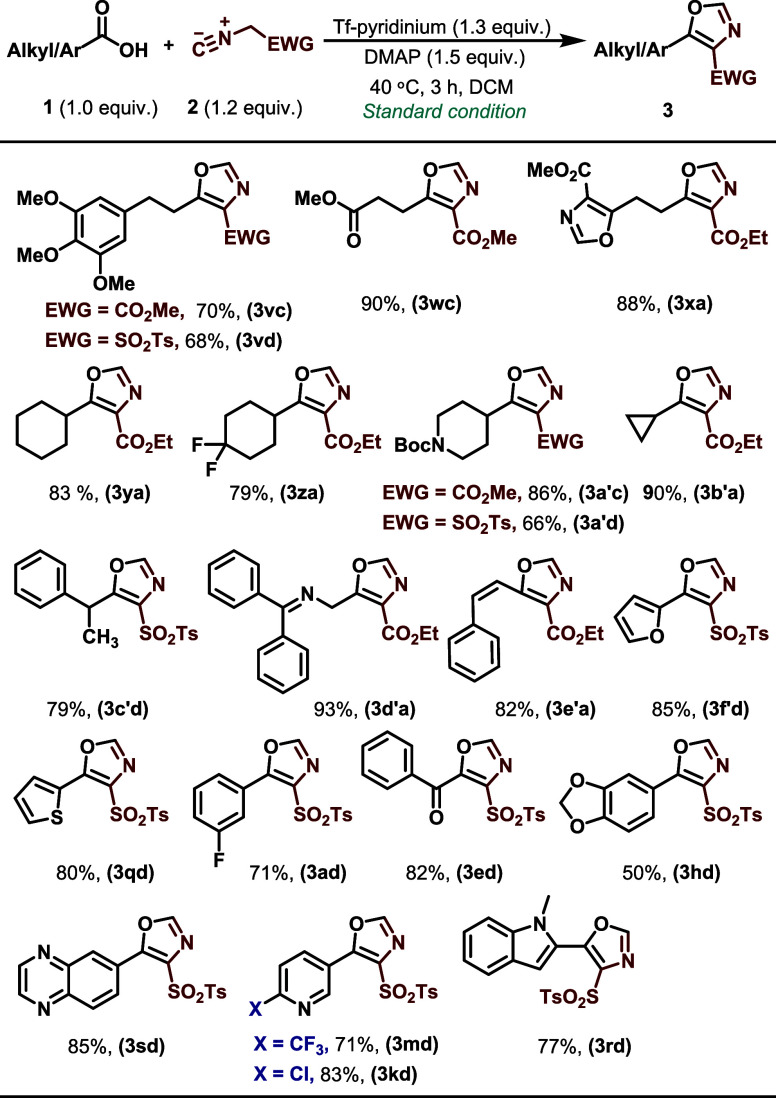
Substrate Scope for Aliphatic Acids and Isocyanoacetates and Tosylmethyl Isocyanide Standard reaction conditions: **1** (1.0 equiv), **2** (1.2 equiv), DMAP-Tf (1.3 equiv), and base (1.5 equiv) in DCM (0.1 M). Yield of the isolated product. ND: not detected

This protocol was further extended to aliphatic carboxylic acids, as summarized in Scheme 3. Primary and secondary aliphatic carboxylic acids reacted more slowly but still produced favorable yields of the desired oxazoles (**3vc–3d’a**) in 3-h reaction time (Scheme 3). (*Z*)-3-Phenylacrylic acid also gave the corresponding product in high yield (**3e’a)**. We also investigated the scope and reactivity of tosylmethyl isocyanide toward aromatic and aliphatic acids. While these reactions were slightly sluggish, they furnished the corresponding oxazoles in good to moderate yields (**3vd, 3c’d** and **3f’d–third**).

**Scheme 4:**
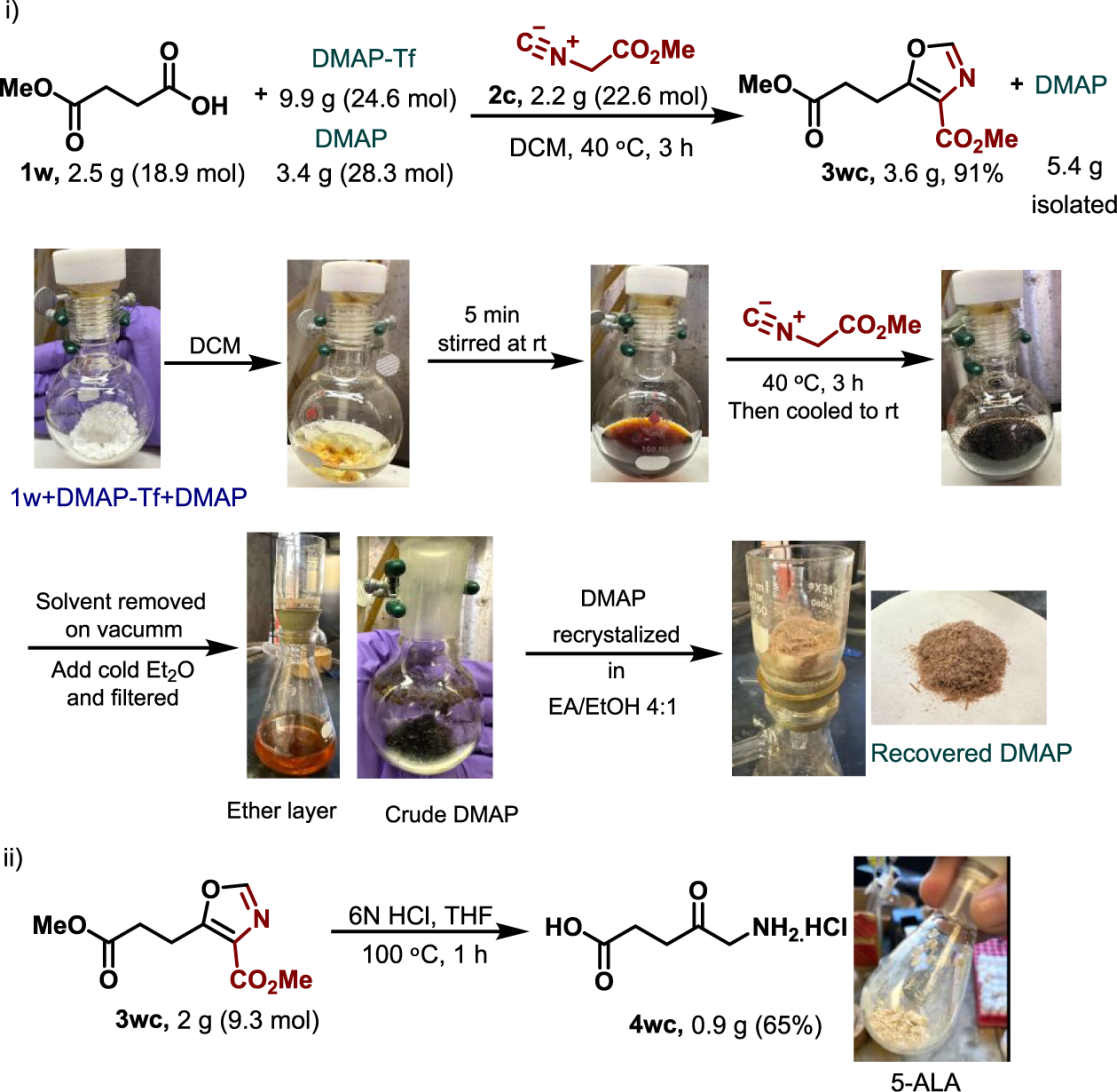
Gram-Scale Synthesis of 5-ALA with DMAP Recovery

The versatility of the isocyanoacetate addition approach was exemplified in a two-step synthesis, leading to the production of the FDA-approved prodrug, 5-aminolevulinic acid (5-ALA, **4wc**). The process was initiated with the reaction of methyl levulinate **1w** and methyl isocyanoacetate **2c**, followed by the hydrolysis of the resulting oxazole **3wc** using 6 N HCl at 100 °C.^14^ This method successfully yielded 5-ALA in 65% yield. To demonstrate the scalability of the method, the synthesis was performed on a gram scale using methyl levulinate and methyl isocyanoacetate, achieving yields consistent with those obtained on a milligram scale (Scheme 4). Additionally, the practicality of the approach was highlighted by the recovery and reuse of the base DMAP (Scheme 4; for more details, see Supporting Information).

**Scheme 5:**
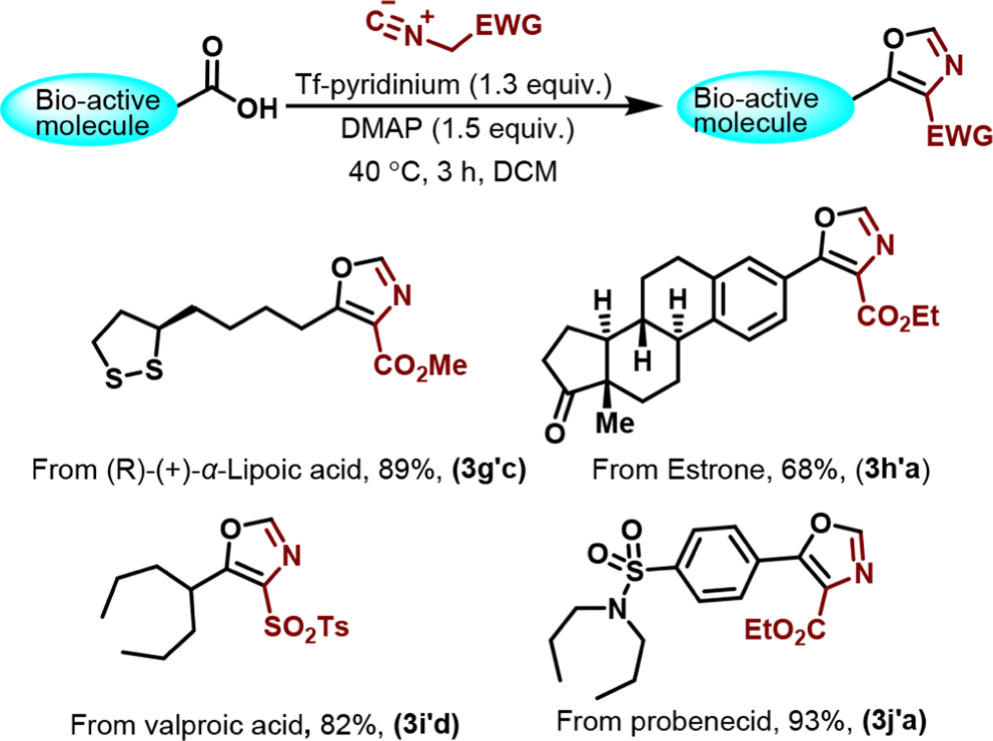
Late-Stage Functionalization

The adaptability and mildness of this established technique were further demonstrated by its successful application to the late-stage functionalization of a series of substrates derived from bioactive compounds. (*R*)-(+)-α-lipoic acid^15^ and estrone-3-carboxylic acid^16^ were subjected to the reactions to afford the desired oxazoles (**3g’c, 3h’a**) in good to excellent yields (Scheme 5). Furthermore, the method’s efficiency was underscored by applying it to FDA-approved drugs such as valproic acid and probenecid, which yielded the corresponding oxazoles in 82% and 93%, respectively (**3i’d, 3j’a,**Scheme 5).

**Scheme 6:**
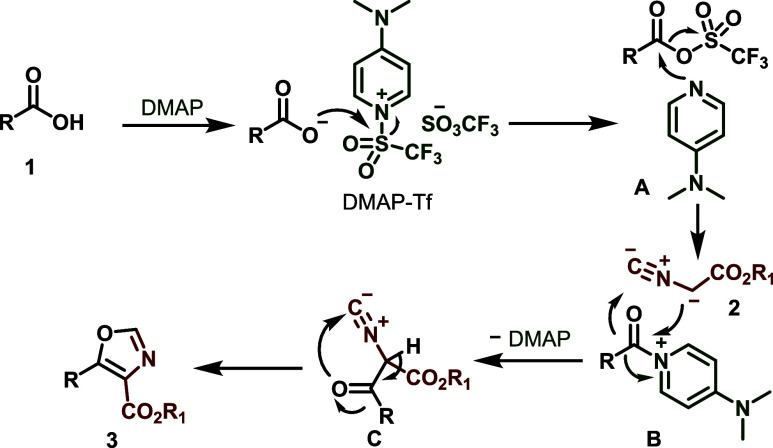
Plausible Mechanism

Based on recent findings, we suggest a plausible reaction mechanism (Scheme 6). The carboxylic acid substrate **1** is first activated *in situ*, forming a trifluorosulfonyl mixed anhydride. The activated intermediate would then undergo nucleophilic attack by DMAP, resulting in the formation of acylpyridinium salt, **B**. Intermediate **B** then reacts *via* an ionic mechanism with the deprotonated alkyl isocyanoacetate **2** to produce intermediate **C,** which further cyclizes into the desired oxazole product **3**.

In summary, we have established a practical synthesis of 4,5-disubstituted oxazoles directly from carboxylic acids utilizing a stable triflylpyridinium reagent and activated methylisocyanides. The reaction exhibits a broad substrate scope and good functional group tolerance. The successful gram-scale synthesis of 5-aminolevulinic acid and the late-stage functionalization of bioactive compounds were demonstrated, emphasizing the practical utility of this transformation in pharmaceutical applications. Additionally, the recyclability of DMAP contributes to the cost-effectiveness of the protocol.

## Experimental Section

### General Information

All solvents were purchased from Fisher Scientific or Sigma-Aldrich and dried over 4 Å molecular sieves (8–12 mesh, Sigma-Aldrich). Unless otherwise noted, all commercially available reagents and substrates were used as received. Thin-layer chromatography was performed on Merck silica gel plates and visualized by UV light and potassium permanganate. ^1^H, ^13^C, and ^19^F NMR spectra were recorded on Bruker 300, Varian INOVA 600, INOVA 500, and INOVA 400 spectrometers. Residual solvent resonances were treated as internal reference signals. ^19^F spectra were referenced to either trifluoroacetic acid (−76.55 ppm) or fluorobenzene (−113.15 ppm). Chemical shifts (δ) are reported in ppm using the residual solvent peak in CDCl_3_ (H δ = 7.26 and C δ = 77.16 ppm) as an internal standard, and coupling constants (*J*) are given in Hz. HRMS were recorded using ESI-TOF techniques at Emory University. IR spectra were recorded on a Nicolet iS10 FT-IR spectrometer, and the absorption peaks were reported in cm^–1^. The purification of products was performed via flash chromatography, unless otherwise noted. High-resolution mass spectra were obtained from the Emory University Mass Spec Facility Inc. All solvents were dried before use, following the standard procedures. Reactions were monitored using thin-layer chromatography (SiO_2_). TLC plates were visualized with UV light (254 nm), iodine treatment, or ninhydrin stain. Column chromatography was carried out using silica gel (60–120 mesh and 100–200 mesh) packed in glass columns.

#### Experimental Procedure of [3 + 2] Cycloaddition Reaction

##### General Procedure for the Syntheses of 4,5-Disubstituted Oxazoles from Aromatic Acids (**3aa–3ub**)

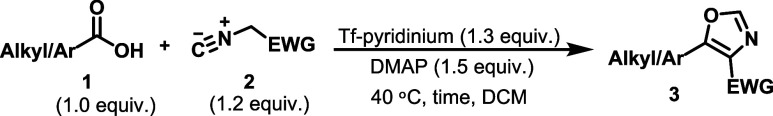



To a screw-capped vial with a spinvane triangular-shaped Teflon stir bar were added carboxylic acid **1** (0.21 mmol, 1.0 equiv), DMAP (0.32 mmol, 1.5 equiv), and solvent DCM (2.0 mL) under a dry nitrogen atmosphere. Then, the DMAP-Tf (0.27 mmol, 1.3 equiv) was added, and the reaction mixture was stirred for 5 min at room temperature. After dissolving all the solids, isocyanide **2** (0.25 mmol, 1.2 equiv) was added to the reaction mixture, and the mixture was stirred in a preheated oil bath at 40 °C for 30 min. The reaction mixture was cooled to room temperature, poured into water (30 mL), and extracted with DCM (20 mL × 3), dried over Na_2_SO_4_ and filtered. The solvents were removed under reduced pressure, and the residue was purified by column chromatography on silica gel (*n*-hexane/EtOAc) to give the desired oxazole derivatives **3**.

##### General Procedure for the Syntheses of 4,5-Disubstituted Oxazoles from Aliphatic Acids and Ts-Isocynides (**25c–49c**)

To a screw-capped vial with a spinvane triangular-shaped Teflon stir bar were added carboxylic acid (0.21 mmol, 1.0 equiv), DMAP (0.32 mmol, 1.5 equiv), and solvent DCM (2.0 mL) under a dry nitrogen atmosphere. Then, the DMAP-Tf (0.27 mmol, 1.3 equiv) was added, and the reaction mixture was stirred for 5 min at room temperature. After dissolving all the solids, isocyanide (0.25 mmol, 1.2 equiv) was added to the reaction mixture, and the mixture was stirred in a preheated oil bath at 40 °C for 3 h. The reaction mixture was cooled to room temperature, poured into water (30 mL), and extracted with DCM (20 mL × 3), dried over Na_2_SO_4_ and filtered. The solvents were removed under reduced pressure, and the residue was purified by column chromatography on silica gel (*n*-hexane/EtOAc) to give the desired oxazole derivatives.

#### Gram-Scale Synthesis Experiments

##### DMAP Recovery

To a screw-capped seal round-bottom flask with a Teflon stir bar were added 4-methoxy-4-oxobutanoic acid (**1w**, 2.5 g, 18.9 mmol, 1.0 equiv), DMAP (3.4 g, 28.3 mmol, 1.5 equiv), and DCM (∼ 50 mL) under a dry nitrogen atmosphere. Then, the DMAP-Tf (9.9 g, 24.6 mmol, 1.3 equiv) was added, and the reaction mixture was stirred for 5 min at room temperature. After dissolving all the solids, methyl isocyanide (**2c,** 2.2 g, 22.6 mmol, 1.2 equiv) was added, and the mixture was stirred in a preheated oil bath at 40 °C for 3 h. After completion of the reaction, the solvent was removed under reduced pressure. Then, the flask was cooled to 0 °C, and cold diethyl ether (100 mL) was added to the reaction mixture. The mixture was then filtered to separate solids, and the solid residue was washed with cold ether. The crude residue was recrystallized using EtOAc/EtOH to give pure solid DMAP. This was then confirmed by 1H NMR.

The filtrate of the reaction was concentrated under reduced pressure to obtain the corresponding crude oxazole product **3wc** (confirmed by 1H NMR) containing trace DMAP, which was removed by washing with 1 N HCl. The crude product was used for the next step.

##### Gram-Scale Synthesis of 5-ALA:^14^

The crude oxazole **3wc** was dissolved in THF (10 mL), and 6 N HCl (10 mL) was added. The mixture was stirred in a preheated oil bath at 100 °C for 1 h. After complete consumption of the starting material (monitored by TLC), the reaction mixture was cooled to room temperature and concentrated under reduced pressure to obtain a tan solid. The crude material, upon crystallization from ethanol (TLC: *^n^*BuOH:H_2_O:CH_3_CO_2_H, 12:5:3, *R*_*f*_ = 0.35), provided the desired compound **4wc**^**1**^**H NMR** (600 MHz, DMSO) δ 7.69 (s, 2H), 3.41 (s, 2H), 2.24–2.20 (m, 2H), 2.00–1.96 (m, 2H). ^**13**^**C {**^**1**^**H} NMR** (151 MHz, DMSO) δ: 207.89, 179.0, 51.9, 39.4, 32.5.

## Data Availability

The data underlying this study are available in the published article and its Supporting Information.

## Supporting Information Available

Copies of the ^1^H and ^13^C spectra for all new compounds (PDF)
