# Supplementary material for: Rapid and Scalable Synthesis of Oxazoles Directly from Carboxylic Acids
Source: J Org Chem. 2025 Mar 5;90(10):3727–32. doi: 10.1021/acs.joc.4c03166 (PMC11915381; doi:10.1021/acs.joc.4c03166)
Supplement: Supplementary file 1 — jo4c03166_si_001.pdf [file jo4c03166_si_001.pdf]

Supporting Information

Rapid and Scalable Synthesis of Oxazoles Directly from Carboxylic Acids

Lahu N. Chavan,[1*] Gouthami Pashikanti,[1*] Mark M. Goodman[2] and Lanny S. Liebeskind[1]

[1]Department of Chemistry, Emory University, 1515 Dickey Drive, Atlanta, Georgia 30322.

[2]Department of Radiology and Imaging Sciences, Wesley Woods Health Center, 1841 Clifton Rd. NE, 2nd floor, Atlanta, GA, 30329.

*E-mail: lchavan@emory.edu

Pages

I. ^{1}H NMR, ^{13}C NMR, 19F NMR spectral data S-03 to S-12

II. ^{1}H NMR, ^{13}C NMR, 19F NMR spectra S-13 to S-74

References S-74 to S-75

I. Spectroscopic Data of Oxazoles Derivatives Obtained in this Study:

General procedure for the syntheses of ethyl 5-(3-fluorophenyl)oxazole-4-carboxylate (3aa):[12]

Following the general procedure a: White solid (32 mg, 97% yield). R_f = 0.5 (EtOAc/Hexane, 6:4); **^{1}H NMR** (600 MHz, $CDCl_3$) δ 7.95 (s, 1H), 7.93 – 7.88 (m, 2H), 7.49 – 7.44 (m, 1H), 7.19 (tdd, J = 8.3, 2.5, 0.8 Hz, 1H), 4.45 (q, J = 7.1 Hz, 2H), 1.44 (t, J = 7.1 Hz, 3H). **^{13}C {^{1}H} NMR** (151 MHz, $CDCl_3$) δ 162.5 (d, J = 234.7 Hz), 161.8, 154.0 (d, J = 3.0 Hz), 149.2, 130.1 (d, J = 8.34 Hz), 128.5 (d, J = 9.23 Hz), 127.3, 124.1 (d, J = 3.18 Hz), 117.4 (d, J = 21.54 Hz), 115.5 (d, J = 24.61 Hz), 61.7, 14.2. **^{19}F NMR** (565 MHz, $CDCl_3$) δ -112.02 – -112.07 (m).

Ethyl 5-(4-iodophenyl)oxazole-4-carboxylate (3ba):[1]

Following the general procedure a: White solid (23 mg, 90% yield). R_f = 0.5 (EtOAc/Hexane, 6:4); (0.510 g, 85% yield). **^{1}H NMR** δ 7.94 (s, 1H), 7.87 – 7.83 (m, 4H), 4.44 (q, J = 7.1 Hz, 2H), 1.43 (t, J = 7.1 Hz, 3H). **^{13}C {^{1}H} NMR** (151 MHz, $CDCl_3$) δ 161.8, 154.6, 149.1, 137.7, 129.9, 127.1, 126.1, 97.2, 61.6, 14.3.

Ethyl 5-(4-bromophenyl)oxazole-4-carboxylate (3ca):[2]

Following the general procedure a: White solid (28 mg, 95% yield). R_f = 0.5 (EtOAc/Hexane, 6:4); **^{1}H NMR** δ 8.03 – 7.99 (m, 2H), 7.94 (s, 1H), 7.65 – 7.62 (m, 2H), 4.44 (q, J = 7.1 Hz, 2H), 1.44 (t, J = 7.1 Hz, 3H).**^{13}C {^{1}H} NMR** (151 MHz, $CDCl_3$) δ 161.9, 154.5, 149.1, 131.7, 129.9, 127.0, 125.6, 125.1, 61.6, 14.3.

Ethyl 5-(4-nitrophenyl)oxazole-4-carboxylate (3da):[3]

Following the general procedure a: White solid (30 mg, 97% yield). R_f = 0.5 (EtOAc/Hexane, 6:4); **^{1}H NMR** (300 MHz, $CDCl_3$) δ 8.37 – 8.30 (m, 4H), 8.01 (s, 1H), 4.45 (q, J = 7.1 Hz, 2H), 1.43 (t, J = 7.1 Hz, 3H). **^{13}C {^{1}H} NMR** (151 MHz, $CDCl_3$) δ 161.6, 152.8, 150.0, 148.5, 132.4, 129.3, 129.0, 123.7, 62.0, 14.

Ethyl 5-benzoyloxazole-4-carboxylate (3ea):[4]

Following the general procedure a: White solid (30 mg, 92% yield). R_f = 0.5 (EtOAc/Hexane, 6:4); **^{1}H NMR** (600 MHz, $CDCl_3$) δ 8.07 (s, 1H), 7.90 – 7.87 (m, 2H), 7.70 – 7.65 (m, 1H), 7.56 – 7.50 (m, 2H), 4.19 (q, J = 7.1 Hz, 2H), 1.08 (t, J = 7.1 Hz, 3H). **^{13}C {^{1}H} NMR** (151 MHz, $CDCl_3$) δ 183.4, 160.2, 151.2, 149.4, 136.1, 134.4, 133.9, 129.6, 128.8, 61.9, 13.6.

Ethyl 5-(o-tolyl)oxazole-4-carboxylate (3fa):

Following the general procedure a: White solid (30 mg, 89% yield). R_f = 0.5 (EtOAc/Hexane, 6:4); **^{1}H NMR** (600 MHz, $CDCl_3$) δ 7.98 (s, 1H), 7.48 – 7.45 (m, 1H), 7.43– 7.39 (m, 1H), 7.33– 7.27 (m, 2H), 4.32 (q, J = 7.1 Hz, 2H), 2.29 (s, 3H), 1.29 (t, J = 7.1 Hz, 3H).**^{13}C {^{1}H}**

NMR (151 MHz, $CDCl_3$) δ 161.4, 156.2, 149.8, 138.0, 130.9, 130.5, 130.4, 128.3, 126.6, 125.4, 61.2, 20.0, 14.1.

Ethyl 5-phenyloxazole-4-carboxylate (3ga):[2]

Following the general procedure a: White solid (33 mg, 93% yield). R_f = 0.5 (EtOAc/Hexane, 6:4); **^{1}H NMR** (300 MHz, $CDCl_3$) δ 8.11 – 8.03 (m, 2H), 7.92 (s, 1H), 7.52 – 7.44 (m, 3H), 4.42 (q, J = 7.2 Hz, 2H), 1.41 (t, J = 7.2 Hz, 3H). **^{13}C {^{1}H} NMR** (151 MHz, $CDCl_3$) δ 162.0, 155.6, 149.0, 130.5, 128.5, 128.4, 126.7, 126.6, 61.4, 14.2.

Tert-butyl 5-phenyloxazole-4-carboxylate (3gb):[2]

Following the general procedure a: White solid (36 mg, 91% yield). R_f = 0.5 (EtOAc/Hexane, 6:4); **^{1}H NMR** (300 MHz, $CDCl_3$) δ 8.01 – 7.95 (m, 2H), 7.89 (s, 1H), 7.52 – 7.43 (m, 3H), 1.59 (s, 9H). **^{13}C {^{1}H} NMR** (151 MHz, $CDCl_3$) δ 161.1, 154.8, 149.0, 130.2, 128.6, 128.3, 127.9, 127.1, 82.4, 28.2.

Ethyl 5-(benzo[d][1,3]dioxol-5-yl)oxazole-4-carboxylate (3ha):[3]

Following the general procedure a: White solid (22 mg, 70% yield). R_f = 0.5 (EtOAc/Hexane, 6:4); **^{1}H NMR** (300 MHz, $CDCl_3$) δ 7.85 (s, 1H), 7.71 – 7.66 (m, 1H), 7.64 – 7.61 (m, 1H), 6.93 – 6.88 (m, 1H), 6.04 (s, 2H), 4.42 (q, *J* = 7.1 Hz, 2H), 1.42 (t, *J* = 7.1 Hz, 3H). **^{13}C {^{1}H} NMR** (151 MHz, $CDCl_3$) δ 162.1, 155.4, 149.5, 148.4, 147.8, 125.6, 123.6, 120.5, 108.7, 108.4, 101.6, 61.4, 14.3.

Methyl 5-(4-fluoro-3-(trifluoromethyl)phenyl)oxazole-4-carboxylate (3ia):

Following the general procedure a: White solid (26 mg, 96% yield). R_f = 0.4 (EtOAc/Hexane, 6:4); **^{1}H NMR** (300 MHz, $CDCl_3$) δ 8.49 – 8.44 (m, 1H), 8.44 – 8.37 (m, 1H), 7.95 (s, 1H), 7.33 (t, *J* = 9.3 Hz, 1H), 3.97 (s, 3H). **^{13}C {^{1}H} NMR** (151 MHz, $CDCl_3$) δ 162.1, 161.6 – 159.6 (m), 153.2, 149.3, 134.1 (d, *J* = 9.0 Hz), 127.8 – 127.6 (m), 127.2, 123.2 (d, *J* = 4.0 Hz), 122.2 (q, *J* = 272.6 Hz), 119.6 – 118.6 (m), 117.4 (d, *J* = 21.3 Hz), 52.6. **^{19}F NMR** (376 MHz, $CDCl_3$) δ -61.60 (d, *J* = 12.6 Hz), -110.27 – -110.46 (m). HRMS (ESI) calcd for $C_{12}H_7F_4NO_3$ $[M+H]^+$: 290.0434; found: 290.0439.

Ethyl 5-(3-chloropyridin-4-yl)oxazole-4-carboxylate (3ja):

Following the general procedure a: White solid (29 mg, 93% yield). R_f = 0.3 (EtOAc/Hexane, 6:4); **^{1}H NMR** δ 8.78 – 8.77 (m, 1H), 8.64 (d, *J* = 4.9 Hz, 1H), 8.07 (s, 1H), 7.51 (dd, *J* = 4.9, 0.6 Hz, 1H), 4.35 (q, *J* = 7.2 Hz, 2H), 1.31 (t, *J* = 7.2 Hz, 3H).**^{13}C {^{1}H} NMR** (151 MHz, $CDCl_3$) δ 160.6, 151.0, 150.3, 149.8, 147.6, 134.0, 131.2, 130.7, 125.5, 61.7, 14.0.

Ethyl 5-(6-chloropyridin-3-yl)oxazole-4-carboxylate (3ka):[3]

Following the general procedure a: White solid (29 mg, 91% yield). R_f = 0.3 (EtOAc/Hexane, 6:4); **^{1}H NMR** (300 MHz, $CDCl_3$) δ 9.02 – 9.00 (m, 1H), 8.53 (dd, *J* = 8.5, 2.5 Hz, 1H), 7.98

(s, 1H), 7.46 (dd, *J* = 8.5, 0.7 Hz, 1H), 4.44 (q, *J* = 7.1 Hz, 2H), 1.42 (t, *J* = 7.1 Hz, 3H). **^{13}C {^{1}H} NMR** (151 MHz, $CDCl_3$) *δ* 161.6, 153.0, 151.8, 149.8, 149.1, 138.3, 128.5, 124.0, 122.1, 62.0, 14.2.

Ethyl 5-(6-fluoropyridin-3-yl)oxazole-4-carboxylate (3la):

Following the general procedure a: White solid (25 mg, 77% yield). R_f = 0.3 (EtOAc/Hexane, 6:4); **^{1}H NMR** (600 MHz, $CDCl_3$) *δ* 8.38 (d, *J* = 5.3 Hz, 1H), 8.04 (s, 1H), 7.97 (dt, *J* = 5.3, 1.5 Hz, 1H), 7.82 (s, 1H), 4.49 (q, *J* = 7.1 Hz, 2H), 1.47 (t, *J* = 7.1 Hz, 3H).; **^{13}C {^{1}H} NMR** (151 MHz, $CDCl_3$) *δ* 165.0 (d, *J* = 238.1 Hz), 161.3, 151.2 (d, *J* = 4.6 Hz), 150.2, 148.4 (d, *J* = 15.13 Hz), 138.7 (d, *J* = 9.43 Hz), 130.2, 119.4 (d, *J* = 4.72 Hz), 108.3 (d, *J* = 40.91 Hz), 62.2, 14.2.; **^{19}F NMR** (565 MHz, $CDCl_3$) *δ* -66.26 (s). HRMS (ESI) calcd for $C_{11}H_{10}O_3N_2F$ $[M+H]^+$: 237.0670; found: 237.0671.

Ethyl 5-(6-(trifluoromethyl)pyridin-3-yl)oxazole-4-carboxylate (3ma):

Following the general procedure a: White solid (28 mg, 94% yield). R_f = 0.3 (EtOAc/Hexane, 6:4); **^{1}H NMR** (300 MHz, $CDCl_3$) *δ* 9.34 – 9.30 (m, 1H), 8.79 – 8.72 (m, 1H), 8.04 (s, 1H), 7.81 (d, *J* = 8.4 Hz, 1H), 4.45 (q, *J* = 7.1 Hz, 2H), 1.43 (t, *J* = 7.2 Hz, 3H). **^{13}C {^{1}H} NMR** (151 MHz, $CDCl_3$) *δ* 161.4, 151.2, 150.3, 149.0, 148.9 (q, *J* = 35.2 Hz), 137.2, 129.5, 125.8, 121.1 (q, *J* = 274.3 Hz), 120.1, 62.0, 14.2. HRMS (ESI) calcd for $C_{12}H_{10}F_3N_2O_3$ $[M+H]^+$: 287.0638; found: 287.0634.

Methyl 5-(2-fluoropyridin-4-yl)oxazole-4-carboxylate (3nc):

Following the general procedure a: White solid (22 mg, 71% yield). R_f = 0.3 (EtOAc/Hexane, 6:4); **^{1}H NMR** (300 MHz, $CDCl_3$) *δ* 8.36 (d, *J* = 5.4 Hz, 1H), 8.03 (s, 1H), 7.96 (dt, *J* = 5.4, 1.5 Hz, 1H), 7.81 – 7.78 (m, 1H), 4.00 (s, 3H). **^{13}C {^{1}H} NMR** (151 MHz, $CDCl_3$) *δ* 164.2 (d, *J* = 238.5 Hz), 161.7, 151.4 (d, *J* = 4.3 Hz), 150.2, 148.4 (d, *J* = 15.1 Hz), 138.6 (d, *J* = 9.1 Hz), 129.9, 119.4 (d, *J* = 4.4 Hz), 108.1 (d, *J* = 41.1 Hz), 52.9. **^{19}F NMR** (565 MHz, $CDCl_3$) *δ* -66.15. HRMS (ESI) calcd for $C_{10}H_7FN_2O_3$ $[M+H]^+$: 223.0513; found: 223.0516.

Ethyl 5-(2-(trifluoromethyl)pyridin-3-yl)oxazole-4-carboxylate (3oa):

Following the general procedure a: White solid (26 mg, 87% yield). R_f = 0.3 (EtOAc/Hexane, 6:4); **^{1}H NMR** (600 MHz, $CDCl_3$) *δ* 8.90 (d, *J* = 5.2 Hz, 1H), 8.50 (s, 1H), 8.32 (dd, *J* = 5.1, 1.4 Hz, 1H), 8.07 (s, 1H), 4.50 (q, *J* = 7.1 Hz, 2H), 1.46 (t, *J* = 7.2 Hz, 3H).**^{13}C {^{1}H} NMR** (151 MHz, $CDCl_3$) *δ* 161.2, 151.0, 150.7, 150.4, 149.1 (q, *J* = 35.0 Hz), 135.5, 130.5, 124.27, 124.1, 122.2, 120.4, 118.6, 62.3, 14.2. **^{19}F NMR** (565 MHz, $CDCl_3$) *δ* -68.16. HRMS (ESI) calcd for $C_{12}H_{10}O_3N_2F_3$ $[M+H]^+$: 287.0638; found: 287.0643.

Ethyl 5-(3-chloroisoxazol-5-yl)oxazole-4-carboxylate (3pa):

Following the general procedure a: White solid (26 mg, 81% yield). R_f = 0.3 (EtOAc/Hexane, 6:4); **^{1}H NMR** (600 MHz, $CDCl_3$) δ 8.07 (s, 1H), 7.53 (s, 1H), 4.49 (q, J = 7.1 Hz, 2H), 1.47 (t, J = 7.1 Hz, 3H). **^{13}C {^{1}H} NMR** (151 MHz, $CDCl_3$) δ 160.5, 158.7, 154.2, 151.0, 142.7, 130.3, 108.0, 62.3, 14.2.

Ethyl 5-(thiophen-2-yl)oxazole-4-carboxylate (3qa):

Following the general procedure a: White solid (29 mg, 85% yield). R_f = 0.4 (EtOAc/Hexane, 6:4); **^{1}H NMR** (300 MHz, $CDCl_3$) δ 8.09 (dd, J = 3.8 Hz,1.1 Hz, 1H), 7.81 (s, 1H), 7.55 (dd, J = 5.0 Hz, 1.2 Hz, 1H), 7.16 (dd, J = 5.0 Hz, 3.8 Hz, 1H), 4.46 (q, J = 7.1 Hz,, 2H), 1.44 (t, J = 7.1 Hz, 3H). **^{13}C {^{1}H} NMR** (151 MHz, $CDCl_3$) δ 161.9, 151.4, 148.1, 130.5, 129.9, 128.1, 127.8, 124.6, 61.5, 14.4. HRMS (ESI) calcd for $C_{10}H_9NO_3S$ $[M+H]^+$: 224.0375; found: 224.0378.

Methyl 5-(1-methyl-1H-indol-2-yl)oxazole-4-carboxylate (3rc):

Following the general procedure a: White solid (25 mg, 86% yield). R_f = 0.3 (EtOAc/Hexane, 6:4); **^{1}H NMR** (300 MHz, $CDCl_3$) δ 8.00 (s, 1H), 7.71 – 7.67 (m, 1H), 7.41 – 7.37 (m, 1H), 7.36 – 7.32 (m, 1H), 7.32 – 7.30 (m, 1H), 7.20 – 7.13 (m, 1H), 3.93 (s, 3H), 3.83 (s, 3H). **^{13}C {^{1}H} NMR** (151 MHz, $CDCl_3$) δ 161.6, 149.6, 148.9, 138.8, 128.3, 127.1, 125.1, 124.0, 121.9, 120.4, 109.8, 108.6, 52.4, 31.8. HRMS (ESI) calcd for $C_{14}H_{13}O_3N_2$ $[M+H]^+$: 257.0920; found: 257.0919.

Ethyl 5-(1-methyl-1H-indol-2-yl)oxazole-4-carboxylate (3ra):

Following the general procedure a: White solid (28 mg, 93% yield). R_f = 0.3 (EtOAc/Hexane, 6:4); **^{1}H NMR** (300 MHz, $CDCl_3$) δ 8.00 (s, 1H), 7.71 – 7.66 (m, 1H), 7.42 – 7.36 (m, 1H), 7.36 – 7.30 (m, 1H), 7.30 – 7.28 (m, 1H), 7.20 – 7.12 (m, 1H), 4.40 (q, J = 7.1 Hz, 2H), 3.82 (s, 3H), 1.41 – 1.33 (t, J = 7.1 Hz, 3H). **^{13}C {^{1}H} NMR** (151 MHz, $CDCl_3$) δ 161.2, 149.7, 148.7, 138.8, 128.7, 127.1, 125.2, 123.9, 121.8, 120.4, 109.8, 108.6, 61.5, 31.8, 14.2. HRMS (ESI) calcd for $C_{15}H_{15}O_3N_2$ $[M+H]^+$: 271.1077; found: 271.1073.

Ethyl 5-(quinoxalin-6-yl)oxazole-4-carboxylate (3sa):

Following the general procedure a: White solid (27 mg, 89% yield). R_f = 0.3 (EtOAc/Hexane, 6:4); **^{1}H NMR** (300 MHz, $CDCl_3$) δ 8.91 (dd, J = 5.3, 1.8 Hz, 2H), 8.88 (d, J = 2.0 Hz, 1H), 8.53 – 8.48 (m, 1H), 8.23 – 8.19 (m, 1H), 8.04 (s, 1H), 4.47 (q, J = 7.2 Hz, 2H), 1.44 (t, J = 7.1 Hz, 3H). **^{13}C {^{1}H} NMR** (151 MHz, $CDCl_3$) δ 161.7, 153.9, 149.7, 146.1, 145.9, 143.5, 142.6, 130.1, 129.7, 129.4, 128.3, 128.2, 61.8, 14.3. HRMS (ESI) calcd for $C_{14}H_{12}O_3N_3$ $[M+H]^+$: 270.0873; found: 270.0870.

Tert-butyl 5-(quinoxalin-6-yl)oxazole-4-carboxylate (3sb):

Following the general procedure a: White solid (29 mg, 85% yield). R_f = 0.3 (EtOAc/Hexane, 6:4); **^{1}H NMR** (300 MHz, $CDCl_3$) δ 8.91 (dd, J = 6.0, 1.8 Hz, 2H), 8.82 (d, J = 1.9 Hz, 1H), 8.41 (dd, J = 8.9, 2.0 Hz, 1H), 8.20 (d, J = 8.9 Hz, 1H), 8.01 (s, 1H), 1.62 (s, 9H). **^{13}C {^{1}H} NMR** (151 MHz, $CDCl_3$) δ 160.8, 153.1, 149.7, 145.9, 145.8, 143.3, 142.5, 130.1, 129.6, 129.6, 129.5, 128.6, 83.0, 28.2. HRMS (ESI) calcd for $C_{16}H_{16}O_3N_3$ $[M+H]^+$: 298.1186; found: 298.1185.

Diethyl 5,5'-(1,4-phenylene)bis(oxazole-4-carboxylate) (3ta):

Following the general procedure a using acid **1t** (1 equiv.), DMAP (3 equiv.), DMAP-Tf (2.6 equiv.), **2a** (2.1 equiv.). White solid (39 mg, 91% yield). R_f = 0.3 (EtOAc/Hexane, 6:4); **^{1}H NMR** (300 MHz, $CDCl_3$) δ 8.24 (s, 4H), 7.96 (s, 2H), 4.45 (q, J = 7.1 Hz, 4H), 1.42 (t, J = 4.4 Hz, 6H). **^{13}C {^{1}H} NMR** (151 MHz, $CDCl_3$) δ 161.8, 154.4, 149.3, 128.4, 128.3, 127.6, 61.7, 14.3. HRMS (ESI) calcd for $C_{18}H_{16}O_6N_2$ $[M+H]^+$: 357.1081; found: 357.1087.

Ethyl 5-(4-(diphenylphosphaneyl)phenyl)oxazole-4-carboxylate (3ua):

Following the general procedure a: White solid (23 mg, 89% yield). R_f = 0.2 (EtOAc/Hexane, 8:2); **^{1}H NMR** (300 MHz, $CDCl_3$) δ 8.24 – 8.17 (m, 2H), 7.95 (s, 1H), 7.83 – 7.78 (m, 1H), 7.78 – 7.74 (m, 1H), 7.73 – 7.70 (m, 1H), 7.70 – 7.66 (m, 2H), 7.66 – 7.64 (m, 1H), 7.61 – 7.53 (m, 2H), 7.52 – 7.44 (m, 4H), 4.42 (q, J = 7.1 Hz, 2H), 1.40 (t, J = 7.1 Hz, 3H). **^{13}C {^{1}H} NMR** (151 MHz, $CDCl_3$) δ 161.8, 154.2, 149.5, 135.2, 134.5, 132.3, 132.2, 132.2, 132.1, 132.1, 132.0, 131.6, 129.9 (d, J = 3.0 Hz), 128.6 (d, J = 12.2 Hz), 128.2 (d, J = 12.1 Hz), 128.0, 61.7, 14.2. **^{31}P NMR** (162 MHz, $CDCl_3$) δ 29.0. HRMS (ESI) calcd for $C_{24}H_{21}NO_4P$ $[M+H]^+$: 418.1202; found: 418.1197.

Tert-butyl 5-(4-(diphenylphosphaneyl)phenyl)oxazole-4-carboxylate (3ub):

Following the general procedure a: White solid (25 mg, 90% yield). R_f = 0.2 (EtOAc/Hexane, 8:2); **^{1}H NMR** (300 MHz, $CDCl_3$) δ 8.17 – 8.10 (m, 2H), 7.93 (s, 1H), 7.82 – 7.77 (m, 1H), 7.77 – 7.73 (m, 1H), 7.73 – 7.70 (m, 1H), 7.70 – 7.66 (m, 2H), 7.66 – 7.64 (m, 1H), 7.60 – 7.53 (m, 2H), 7.52 – 7.44 (m, 4H), 1.58 (s, 9H). **^{13}C {^{1}H} NMR** (151 MHz, $CDCl_3$) δ 160.9, 153.4, 149.5, 134.9, 134.2, 132.3, 132.2, 132.2, 132.1, 132.0, 131.6, 130.2 (d, J = 2.8 Hz), 129.2, 128.6 (d, J = 12.2 Hz), 128.3 (d, J = 12.0 Hz), 82.9, 28.2. **^{31}P NMR** (162 MHz, $CDCl_3$) δ 30.3.

Methyl 5-(3,4,5-trimethoxyphenethyl)oxazole-4-carboxylate (3vc):

Following the general procedure b: White solid (18 mg, 70% yield). R_f = 0.3 (EtOAc/Hexane, 6:4); **^{1}H NMR** (300 MHz, $CDCl_3$) δ 7.76 (s, 1H), 6.38 (s, 2H), 3.89 (s, 3H), 3.82 (s, 6H), 3.81 (s, 3H), 3.37 (t, J = 8.5, 7.3 Hz, 2H), 2.95 (t, J = 8.5, 7.3 Hz, 2H). **^{13}C {^{1}H} NMR** (151 MHz, $CDCl_3$) δ 162.4, 159.2, 153.2, 149.0, 136.6, 135.6, 127.2, 105.2, 60.8, 56.1, 52.0, 34.0, 27.6.

4-tosyl-5-(3,4,5-trimethoxyphenethyl)oxazole (3vd):

Following the general procedure b: White solid (23 mg, 68% yield). R_f = 0.3 (EtOAc/Hexane, 6:4); **^{1}H NMR** (300 MHz, $CDCl_3$) δ 7.74 (d, J = 8.0 Hz, 2H), 7.72 (s, 1H), 7.30 (d, J = 8.0 Hz, 2H), 6.37 (s, 2H), 3.83 (s, 3H), 3.79 (s, 6H), 3.45 (t, J = 7.5 Hz, 2H), 3.00 (t, J = 7.5 Hz, 2H), 2.41 (s, 3H). **^{13}C NMR** (151 MHz, $CDCl_3$) δ 156.0, 153.3, 149.6, 144.9, 136.9, 136.7, 135.9, 135.2, 130.4, 129.8, 129.5, 127.9, 105.3, 60.8, 56.1, 34.3, 27.3, 21.7. HRMS (ESI) calcd for $C_{21}H_{24}O_6N^{32}S$ $[M+H]^+$: 418.1318; found: 418.1313.

Methyl 5-(3-methoxy-3-oxopropyl)oxazole-4-carboxylate (3wc):[5]

Following the general procedure b: Colourless liquid (29 mg, 90% yield). R_f = 0.5 (EtOAc/Hexane, 6:4); **^{1}H NMR** (300 MHz, $CDCl_3$) δ 7.77 (s, 1H), 3.91 (s, 3H), 3.68 (s, 3H), 3.38 (t, J = 7.6 Hz, 2H), 2.73 (t, J = 7.5 Hz, 2H). **^{13}C {^{1}H} NMR** (151 MHz, $CDCl_3$) δ 172.1, 162.2, 158.0, 149.1, 127.3, 52.1, 51.9, 31.4, 21.4.

Ethyl 5-(2-(4-(methoxycarbonyl)oxazol-5-yl)ethyl)oxazole-4-carboxylate (3xa):[6]

Following the general procedure b: White solid (26 mg, 88% yield). R_f = 0.4 (EtOAc/Hexane, 6:4); **^{1}H NMR** (600 MHz, $CDCl_3$) δ 7.76 (s, 2H), 4.35 (q, J = 7.2 Hz, 2H), 3.88 (s, 3H), 3.46 (s, 4H), 1.38 (t, J = 7.2 Hz, 3H). **^{13}C {^{1}H} NMR** (151 MHz, $CDCl_3$) δ 162.1, 161.7, 157.4, 157.2, 149.3, 127.9, 127.7, 61.2, 52.1, 24.1, 14.3.

Ethyl 5-cyclohexyloxazole-4-carboxylate (3ya):[7]

Following the general procedure b: White solid (29 mg, 83% yield). R_f = 0.5 (EtOAc/Hexane, 6:4); **^{1}H NMR** (600 MHz, $CDCl_3$) δ 7.76 (s, 1H), 4.41 (q, J = 7.1 Hz, 2H), 3.50 (tt, J = 12.1, 3.3 Hz, 1H), 1.93 – 1.83 (m, 4H), 1.81 – 1.74 (m, 1H), 1.60 – 1.54 (m, 2H), 4.41 (t, J = 7.1 Hz, 3H), 1.44 – 1.41 (m, 1H), 1.34 – 1.28 (m, 1H). **^{13}C {^{1}H} NMR** (151 MHz, $CDCl_3$) δ 164.0, 162.2, 148.6, 125.5, 60.9, 35.5, 30.7, 26.0, 25.7, 14.3.

Ethyl 5-(4,4-difluorocyclohexyl)oxazole-4-carboxylate (3za):[8]

Following the general procedure b: White solid (24 mg, 79% yield). R_f = 0.4 (EtOAc/Hexane, 6:4); **^{1}H NMR** (600 MHz, $CDCl_3$) δ 7.77 (s, 1H), 4.39 (q, J = 7.1 Hz, 2H), 3.66 – 3.51 (m, 1H), 2.29 – 2.13 (m, 2H), 2.03 – 1.89 (m, 5H), 1.88 – 1.75 (m, 1H), 1.40 (t, J = 7.1 Hz, 3H). **^{13}C {^{1}H} NMR** (151 MHz, $CDCl_3$) δ 162.0, 161.4, 148.9, 126.4, 124.0, 122.4, 120.8, 61.2, 33.5 (m), 26.8 (d, J = 9.8 Hz), 14.3. **^{19}F NMR** (565 MHz, $CDCl_3$) δ -92.3 (d, J = 236 Hz), -102.2 (d, J = 241 Hz).

Methyl 5-(1-(tert-butoxycarbonyl)piperidin-4-yl)oxazole-4-carboxylate (3a'c):[8]

White solid (23 mg, 86% yield). R_f = 0.5 (EtOAc/Hexane, 6:4); **^{1}H NMR** (300 MHz, $CDCl_3$) δ 7.76 (s, 1H), 4.30 – 4.09 (m, 2H), 3.91 (s, 3H), 3.64 (tt, J = 11.6, 4.3 Hz, 1H), 2.95 – 2.75 (m, 2H), 1.89 – 1.65 (m, 4H), 1.46 (s, 9H). **^{13}C {^{1}H} NMR** (151 MHz, $CDCl_3$) δ 162.5, 162.0, 154.7, 148.9, 125.9, 79.7, 52.1, 43.4, 33.7, 29.6, 28.4.

Tert-butyl 4-(4-(tosylsulfonyl)oxazol-5-yl)piperidine-1-carboxylate (3a'd):

Following the general procedure b: White solid (23 mg, 66% yield). R_f = 0.3 (EtOAc/Hexane, 6:4); **^{1}H NMR** (300 MHz, $CDCl_3$) δ 7.91 (d, *J* = 8.3 Hz, 2H), 7.73 (s, 1H), 7.35 (d, *J* = 8.3 Hz, 2H), 4.34 – 4.09 (m, 2H), 3.82 – 3.68 (m, 2H), 2.95 – 2.55 (m, 2H), 2.43 (s, 3H), 1.90 – 1.77 (m, 2H), 1.77 – 1.67 (m, 2H), 1.47 (s, 9H). **^{13}C {^{1}H} NMR** (151 MHz, $CDCl_3$) δ 158.5, 154.6, 149.6, 145.0, 137.1, 134.7, 129.9, 128.0, 79.8, 43.4, 33.3, 29.9, 28.4, 21.7. HRMS (ESI) calcd for $C_{20}H_{26}N_2O_5S$ $[M+H]^+$: 405.1489; found: 405.1496.

Ethyl 5-cyclopropyloxazole-4-carboxylate (3b'a):[3]

Following the general procedure b: White solid (37 mg, 90% yield). R_f = 0.4 (EtOAc/Hexane, 6:4); **^{1}H NMR** (600 MHz, $CDCl_3$) δ 7.63 (s, 1H), 4.42 (q, *J* = 7.1 Hz, 2H), 2.83 – 2.77 (m, 1H), 1.43 (t, *J* = 7.1 Hz, 3H), 1.20 – 1.14 (m, 2H), 1.13 – 1.08 (m, 2H). **^{13}C {^{1}H} NMR** (151 MHz, $CDCl_3$) δ 162.5, 161.1, 147.3, 126.7, 60.9, 14.4, 9.1, 7.8.

5-(1-phenylethyl)-4-tosyloxazole (3c'd):

White solid (34 mg, 79% yield). R_f = 0.4 (EtOAc/Hexane, 6:4); **^{1}H NMR** δ 7.79 (d, *J* = 8.3 Hz, 2H), 7.75 (s, 1H), 7.40 – 7.37 (m, 2H), 7.37 – 7.33 (m, 2H), 7.31 – 7.27 (m, 3H), 5.21 (q, *J* = 7.3 Hz, 1H), 2.42 (s, 3H), 1.72 (d, *J* = 7.3 Hz, 3H). **^{13}C {^{1}H} NMR** (151 MHz, $CDCl_3$) δ 158.6, 149.6, 144.8, 141.0, 137.2, 134.2, 129.8, 128.8, 128.0, 127.5, 127.3, 35.8, 21.6, 19.3. HRMS (ESI) calcd for $C_{18}H_{18}O_3NS$ $[M+H]^+$: 328.1001; found: 328.1008.

Ethyl 5-(((diphenylmethylene)amino)methyl)oxazole-4-carboxylate (3d'a):

Following the general procedure b: White solid (32 mg, 93% yield). R_f = 0.3 (EtOAc/Hexane, 6:4); **^{1}H NMR** (600 MHz, $CDCl_3$) δ 7.0 – 7.66 (m, 2H), 7.52 – 7.46 (m, 2H), 7.45 – 7.40 (m, 1H), 7.38 – 7.34 (m, 2H), 7.24 – 7.19 (m, 2H), 4.24 (q, *J* = 7.1 Hz, 2H), 4.23 (s, 2H), 1.30 (t, *J* = 7.1 Hz, 3H). **^{13}C {^{1}H} NMR** (151 MHz, $CDCl_3$) δ 171.9, 170.7, 139.3, 136.0, 130.5, 128.8, 128.8, 128.7, 128.1, 127.1, 60.9, 55.7, 14.2. HRMS (ESI) calcd for $C_{20}H_{19}N_2O_3$ $[M+H]^+$: 335.1390; found: 335.1388.

Ethyl (*Z*)-5-styryloxazole-4-carboxylate (3e'a):

Following the general procedure b: White solid (27 mg, 82 % yield). R_f = 0.4 (AcOEt/Hexane, 6:4); **^{1}H NMR** (300 MHz, $CDCl_3$) δ 7.81 (s, 1H), 7.66 – 7.59 (m, 1H), 7.58 – 7.54 (m, 2H), 7.43 – 7.38 (m, 2H), 7.38 – 7.31 (m, 2H), 4.44 (q, *J* = 7.1 Hz, 2H), 1.45 (t, *J* = 7.1 Hz, 3H).**^{13}C {^{1}H} NMR** (151 MHz, $CDCl_3$) δ 162.0, 154.8, 149.0, 135.6, 135.3, 129.4, 128.9, 127.4, 126.8, 112.9, 61.3, 14.4. HRMS (ESI) calcd for $C_{14}H_{14}O_3N$ $[M+H]^+$: 244.0968; found: 244.0971.

5-(furan-2-yl)-4-tosyloxazole (3f'd):[2]

Following the general procedure b: White solid (43 mg, 85% yield). R_f = 0.3 (EtOAc/Hexane, 7:3); **^{1}H NMR** (300 MHz, $CDCl_3$) δ 7.96 – 7.90 (m, 2H), 7.80 (s, 1H), 7.64 – 7.62 (m, 1H),

7.60 – 7.57 (m, 1H), 7.35 – 7.30 (m, 2H), 6.62 (dd, *J* = 3.6, 1.8 Hz, 1H), 2.41 (s, 3H). **^{13}C {^{1}H} NMR** (151 MHz, $CDCl_3$) *δ* 148.8, 145.3, 145.1, 144.1, 140.4, 137.1, 133.6, 129.9, 128.0, 116.1, 112.6, 21.7. HRMS (ESI) calcd for $C_{14}H_{11}NO_4S$ $[M+H]^+$: 290.0481; found: 290.0485.

5-(thiophen-2-yl)-4-tosyloxazole (3qd):

Following the general procedure b: White solid (38 mg, 80% yield). R_f = 0.3 (EtOAc/Hexane, 7:3); **^{1}H NMR** (300 MHz, $CDCl_3$) *δ* 8.11 (dd, *J* = 3.8, 1.2 Hz, 1H), 7.93 (d, *J* = 8.4 Hz, 2H), 7.76 (s, 1H), 7.58 (dd, *J* = 5.1, 1.2 Hz, 1H), 7.33 (d, *J* = 8.0 Hz, 2H), 7.19 (dd, *J* = 5.0, 3.8 Hz, 1H), 2.42 (s, 3H). **^{13}C {^{1}H} NMR** (151 MHz, $CDCl_3$) *δ* 148.4, 148.1, 145.1, 137.2, 133.3, 131.6, 130.4, 129.9, 128.3, 128.0, 126.5, 21.7. HRMS (ESI) calcd for $C_{14}H_{11}NO_3S_2$ $[M+H]^+$: 306.0253; found: 306.0258.

5-(3-fluorophenyl)-4-tosyloxazole (3ad):[9]

Following the general procedure b: White solid (32 mg, 71% yield). R_f = 0.3 (EtOAc/Hexane, 7:3); **^{1}H NMR** (600 MHz, $CDCl_3$) *δ* 7.94 – 7.91 (m, 2H), 7.90 (s, 1H), 7.85 (ddd, *J* = 7.8, 1.6, 0.9 Hz, 1H), 7.74 (ddd, *J* = 9.7, 2.4, 1.8 Hz, 1H), 7.51 (dt, *J* = 8.3, 5.5 Hz, 1H), 7.38 – 7.33 (m, 2H), 7.24 (tdd, *J* = 8.3, 2.6, 0.9 Hz, 1H), 2.45 (s, 3H). **^{13}C {^{1}H} NMR** (151 MHz, $CDCl_3$) *δ* 163.3, 161.6, 151.1 (d, *J* = 2.8 Hz), 149.4, 145.2, 136.8, 136.4, 130.4 (d, *J* = 8.2 Hz), 129.1, (d, *J* = 139.7 Hz), 127.3 (d, *J* = 8.7 Hz), 124.9 (d, *J* = 3.2 Hz), 118.0 (d, *J* = 21.1 Hz), 115.9 (d, *J* = 24.5 Hz), 21.7. **^{19}F NMR** (565 MHz, $CDCl_3$) *δ* -111.54 --111.59 (m).

Phenyl(4-tosyloxazol-5-yl)methanone (3ed):

Following the general procedure b: White solid (44 mg, 82% yield). R_f = 0.4 (EtOAc/Hexane, 7:3); **^{1}H NMR** (600 MHz, $CDCl_3$) *δ* 8.10 – 8.06 (m, 2H), 7.99 (s, 1H), 7.95 – 7.91 (m, 2H), 7.73 – 7.69 (m, 1H), 7.58 – 7.53 (m, 2H), 7.41 – 7.38 (m, 2H), 2.47 (s, 3H). **^{13}C NMR** (151 MHz, $CDCl_3$) *δ* 181.9, 150.4, 147.9, 145.6, 143.1, 135.9, 135.2, 134.9, 130.0, 129.9, 129.1, 128.9, 21.8. HRMS (ESI) calcd for $C_{17}H_{13}NO_4S$ $[M+H]^+$: 328.0638; found: 328.0640.

5-(benzo[d][1,3]dioxol-5-yl)-4-tosyloxazole (3hd):

Following the general procedure b: White solid (20 mg, 50% yield). R_f = 0.4 (EtOAc/Hexane, 6:4); **^{1}H NMR** (300 MHz, $CDCl_3$) *δ* 7.93 – 7.86 (m, 2H), 7.79 (s, 1H), 7.57 – 7.53 (m, 1H), 7.47 – 7.44 (m, 1H), 7.35 – 7.30 (m, 2H), 6.93 (d, *J* = 8.3 Hz, 1H), 6.06 (s, 1H), 2.42 (s, 3H). **^{13}C {^{1}H} NMR** (151 MHz, $CDCl_3$) *δ* 152.5, 149.9, 148.6, 147.9, 144.9, 137.2, 134.6, 129.8, 128.2, 124.3, 119.2, 109.1, 108.5, 101.8, 21.7. HRMS (ESI) calcd for $C_{17}H_{13}NO_5S$ $[M+H]^+$: 344.0587; found: 344.0589.

5-(quinoxalin-6-yl)-4-tosyloxazole (3sd):

Following the general procedure b: White solid (34 mg, 85% yield). R_f = 0.3 (EtOAc/Hexane, 6:4); **^{1}H NMR** (600 MHz, $CDCl_3$) *δ* 8.96 (d, *J* = 7.7 Hz, 2H), 8.77 (s, 1H), 8.47 – 8.43 (m, 1H),

8.28 – 8.25 (m, 1H), 8.00 – 7.96 (m, 3H), 7.37 (d, J = 7.9 Hz, 2H), 2.44 (s, 3H). **^{13}C {^{1}H} NMR** (151 MHz, $CDCl_3$) δ 150.9, 149.9, 146.3, 146.1, 145.3, 143.6, 142.4, 137.4, 136.7, 130.7, 130.0, 129.9, 129.8, 128.5, 127.1, 21.7. HRMS (ESI) calcd for $C_{18}H_{13}N_3O_3S$ [M+H]$^+$: 352.0750; found: 352.0756.

5-(6-methylpyridin-3-yl)-4-tosyloxazole (3md):

Following the general procedure b: White solid (19 mg, 71% yield). R_f = 0.3 (EtOAc/Hexane, 6:4); **^{1}H NMR** (300 MHz, $CDCl_3$) δ 9.22 – 9.19 (m, 1H), 8.68 – 8.62 (m, 1H), 7.97 (s, 1H), 7.94 – 7.89 (m, 2H), 7.88 – 7.84 (m, 1H), 7.39 – 7.34 (m, 2H), 2.44 (s, 3H). **^{13}C {^{1}H} NMR** (151 MHz, $CDCl_3$) δ 150.5, 149.4 (q, *J* = 35.4 Hz), 149.2, 148.1, 145.7, 138.7, 138.2, 136.1, 130.0, 128.5, 124.7, 123.9, 122.0, 120.3-120.1 (m), 118.4, 21.7.

5-(6-chloropyridin-3-yl)-4-tosyloxazole (3kd):[3]

Following the general procedure b: White solid (35 mg, 83% yield). R_f = 0.3 (EtOAc/Hexane, 6:4); **^{1}H NMR** (300 MHz, $CDCl_3$) δ 8.88 (dd, *J* = 2.5, 0.6 Hz, 1H), 8.41 (dd, *J* = 8.4, 2.5 Hz, 1H), 7.92 (s, 1H), 7.92 – 7.87 (m, 2H), 7.50 (dd, *J* = 8.4, 0.7 Hz, 1H), 7.38 – 7.32 (m, 2H), 2.43 (s, 3H).**^{13}C {^{1}H} NMR** (151 MHz, $CDCl_3$) δ 153.6, 150.1, 149.2, 148.7, 145.6, 139.1, 137.7, 136.4, 130.0, 128.4, 124.2, 121.0, 21.7.

5-(1-methyl-1H-indol-2-yl)-4-tosyloxazole (3rd):

Following the general procedure b: White solid (31 mg, 77% yield). R_f = 0.3 (EtOAc/Hexane, 6:4); **^{1}H NMR** (300 MHz, $CDCl_3$) δ 7.95 (s, 1H), 7.91 – 7.86 (m, 2H), 7.72 (dt, *J* = 8.0, 1.0 Hz, 1H), 7.40 – 7.34 (m, 2H), 7.33 – 7.28 (m, 2H), 7.24 –7.22 (m, 1H), 7.22 –7.16 (m, 1H), 3.78 (s, 3H), 2.41 (s, 3H).**^{13}C {^{1}H} NMR** (151 MHz, $CDCl_3$) δ 150.0, 145.8, 145.1, 138.9, 138.0, 137.0, 129.8, 128.3, 127.0, 124.2, 123.3, 122.0, 120.5, 109.9, 109.5, 31.7, 21.7. HRMS (ESI) calcd for $C_{19}H_{16}N_2O_3S$ [M+H]$^+$: 353.0954; found: 353.0957.

Methyl (*R*)-5-(4-(1,2-dithiolan-3-yl)butyl)oxazole-4-carboxylate (3g'c):

Following the general procedure b: White solid (26 mg, 89% yield). R_f = 0.5 (EtOAc/Hexane, 7:3); **^{1}H NMR** (600 MHz, $CDCl_3$) δ 7.78 (s, 1H), 3.93 (s, 3H), 3.61 – 3.54 (m, 1H), 3.23 – 3.18 (m, 1H), 3.16 – 3.12 (m, 1H), 3.09 (t, *J* = 7.5 Hz, 2H), 2.48 (qd, *J* = 6.6, 5.5 Hz, 1H), 1.93 (dq, *J* = 13.7, 6.9 Hz, 1H), 1.79 – 1.71 (m, 4H), 1.60 – 1.44 (m, 2H). **^{13}C {^{1}H} NMR** (151 MHz, $CDCl_3$) δ 162.5, 160.0, 149.0, 127.0, 56.3, 52.0, 40.2, 38.5, 34.5, 28.6, 27.3, 25.6. HRMS (ESI) calcd for $C_{12}H_{17}NO_3S_2$ [M+H]$^+$: 288.0722; found: 288.0728.

Ethyl 5-((8R,9S,13S,14S)-13-methyl-17-oxo-7,8,9,11,12,13,14,15,16,17-decahydro-6H-cyclopenta [a]phenanthrene-3-yl)oxazole-4-carboxylate (3h'a):

Following the general procedure b: White solid (17 mg, 68% yield). R_f = 0.5 (EtOAc/Hexane, 7:3); **^{1}H NMR** (600 MHz, $CDCl_3$) δ 7.91 (s, 1H), 7.87 (dd, *J* = 8.2, 1.8 Hz, 1H), 7.84 (s, 1H),

7.43 (d, *J* = 8.2 Hz, 1H), 4.45 (q, *J* = 7.1 Hz, 2H), 3.07 – 2.97 (m, 2H), 2.55 (dd, *J* = 19.2, 8.4 Hz, 1H), 2.51 – 2.46 (m, 1H), 2.39 (td, *J* = 11.3, 4.2 Hz, 1H), 2.19 (dt, *J* = 18.9, 8.9 Hz, 1H), 2.14 – 2.07 (m, 2H), 2.02 (dt, *J* = 12.8, 3.2 Hz, 1H), 1.71 – 1.63 (m, 3H), 1.61 – 1.53 (m, 3H), 1.44 (t, *J* = 7.1 Hz, 3H), 0.95 (s, 3H). **^{13}C {^{1}H} NMR** (151 MHz, $CDCl_3$) δ 162.0, 155.8, 148.8, 142.6, 136.8, 129.0, 126.3, 125.9, 125.5, 124.2, 61.4, 50.5, 47.9, 44.6, 37.9, 35.8, 31.6, 29.4, 26.4, 25.6, 21.6, 14.3, 13.8. HRMS (ESI) calcd for $C_{24}H_{27}NO_4$ $[M+H]^+$: 394.2012; found: 394.2021.

5-(heptan-4-yl)-4-tosyloxazole (3i’d):

Following the general procedure b: White solid (32 mg, 82% yield). R_f = 0.5 (EtOAc/Hexane, 7:3); **^{1}H NMR** (600 MHz, $CDCl_3$) δ 7.94 (d, *J* = 8.3 Hz, 2H), 7.76 (d, *J* = 4.3 Hz, 1H), 7.36 (d, *J* = 8.0 Hz, 2H), 3.74 (p, *J* = 7.5 Hz, H), 2.45 (s, 3H), 1.68 – 1.63 (m, 4H), 1.35 – 1.26 (m, 2H), 1.19 – 1.10 (m, 2H), 0.91 (t, *J* = 7.3 Hz, 6H).**^{13}C {^{1}H} NMR** (151 MHz, $CDCl_3$) δ 159.7, 149.5, 144.7, 137.5, 136.3, 129.8, 128.1, 36.2, 36.0, 21.7, 20.6, 14.0. HRMS (ESI) calcd for $C_{17}H_{23}NO_3S$ $[M+H]^+$: 322.1471; found: 322.1475.

Ethyl 5-(4-(*N*,*N*-dipropylsulfamoyl)phenyl)oxazole-4-carboxylate (3j’a):

Following the general procedure b: White solid (29 mg, 93% yield). R_f = 0.5 (EtOAc/Hexane, 7:3); **^{1}H NMR** (600 MHz, $CDCl_3$) δ 8.31 – 8.27 (m, 2H), 8.00 (s, 1H), 7.95 – 7.90 (m, 2H), 4.46 (q, *J* = 7.1 Hz, 2H), 3.15 – 3.10 (m, 4H), 1.59 (h, *J* = 7.72 Hz, 4H), 1.45 (t, *J* = 7.1 Hz, 3H), 0.90 (t, *J* = 7.4 Hz, 6H). **^{13}C {^{1}H} NMR** (151 MHz, $CDCl_3$) δ 161.7, 153.6, 149.6, 141.6, 130.2, 128.9, 128.2, 127.1, 61.8, 50.1, 22.1, 14.2, 11.2.

II. ^{1}H NMR, ^{13}C NMR spectra:

Ethyl 5-(3-fluorophenyl)oxazole-4-carboxylate (3aa):

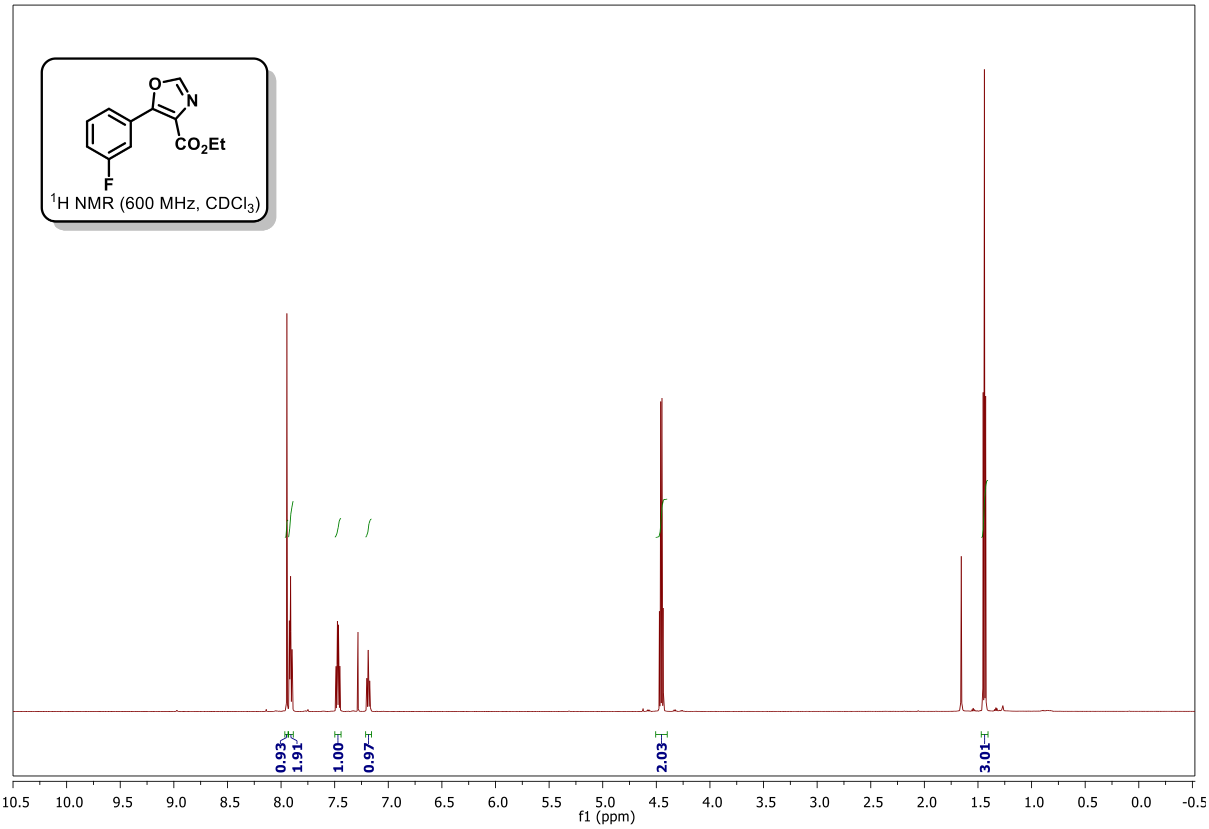

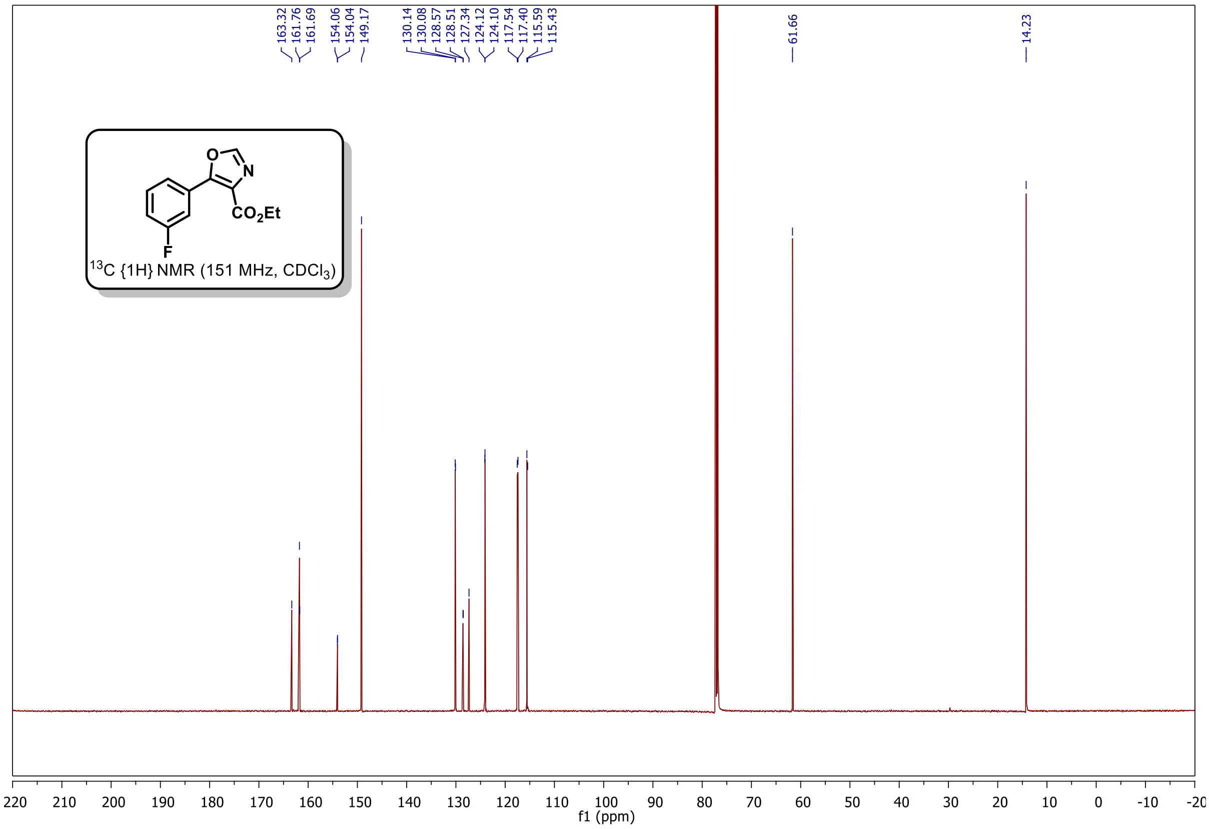

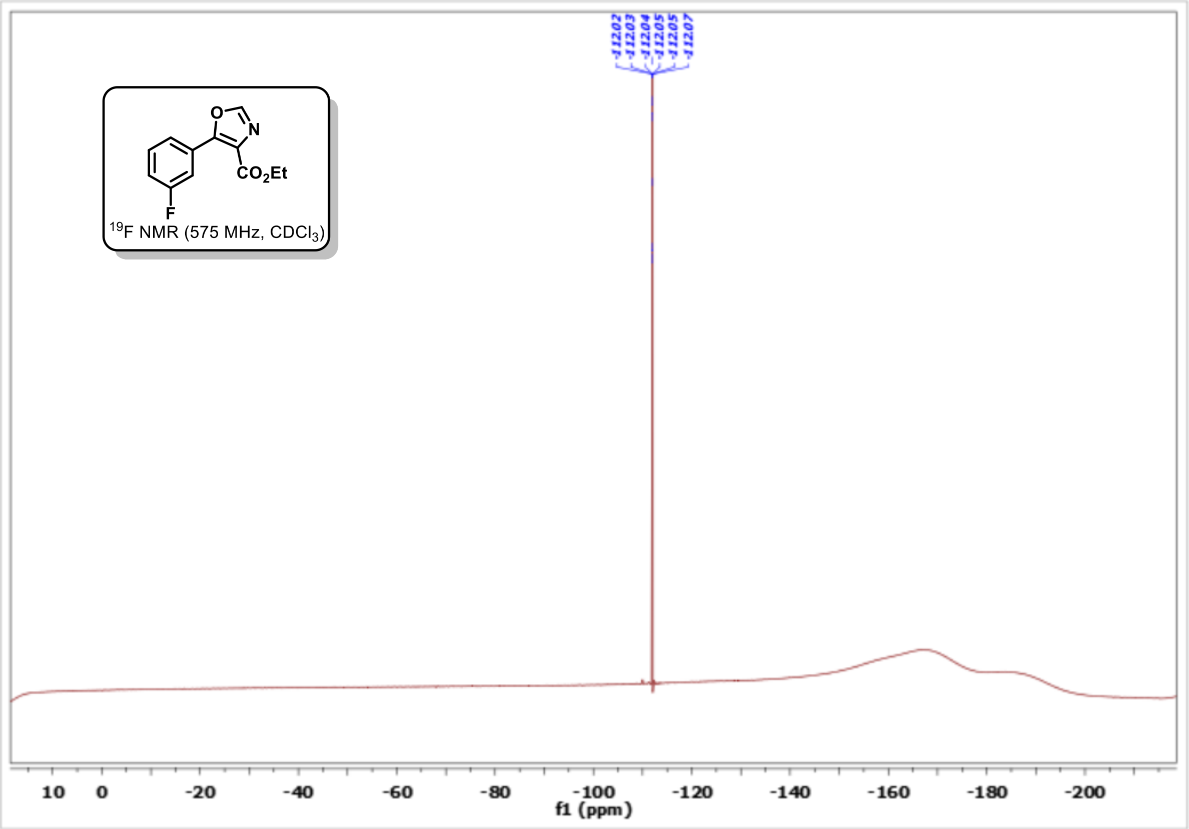
-112.02
-112.03
-112.04
-112.05
-112.05
-112.07
O
N
CO2Et
F
19F NMR (575 MHz, CDCl3)
10
0
-20
-40
-60
-80
-100
-120
-140
-160
-180
-200
f1 (ppm)

Ethyl 5-(4-iodophenyl)oxazole-4-carboxylate (3ba):

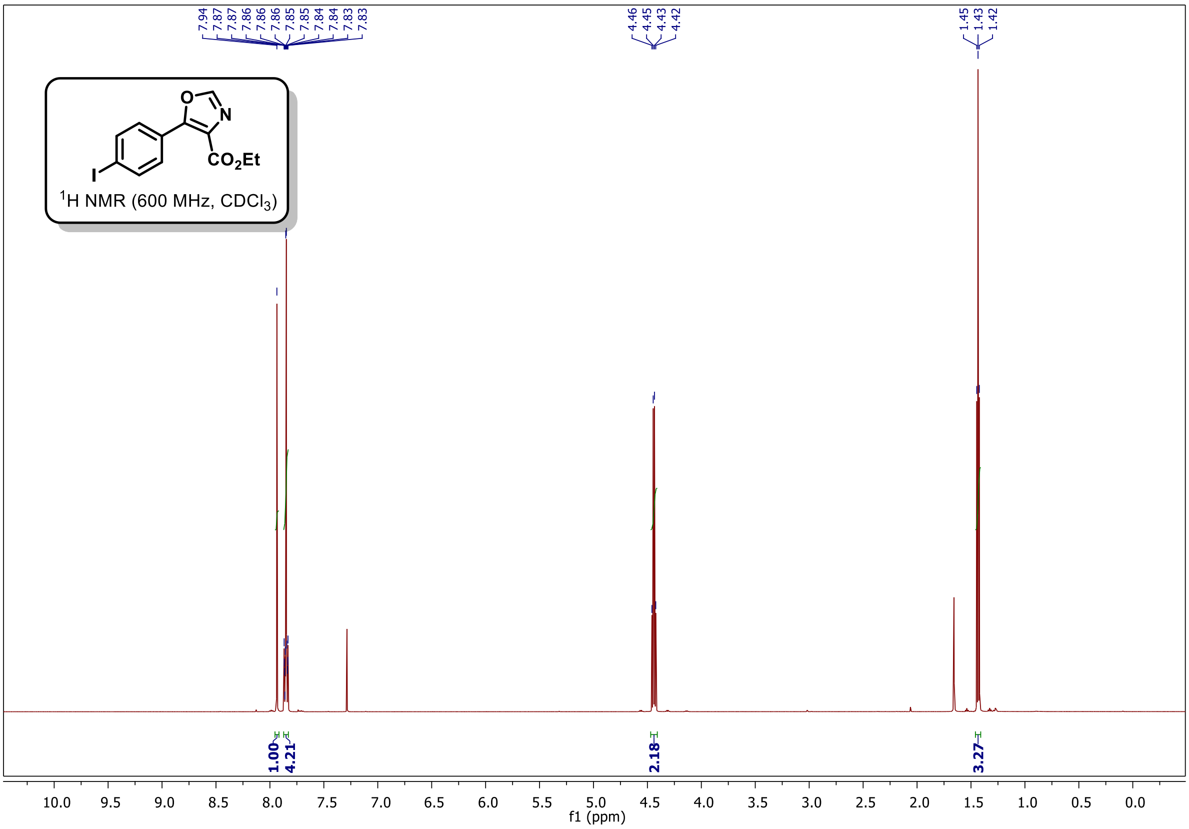

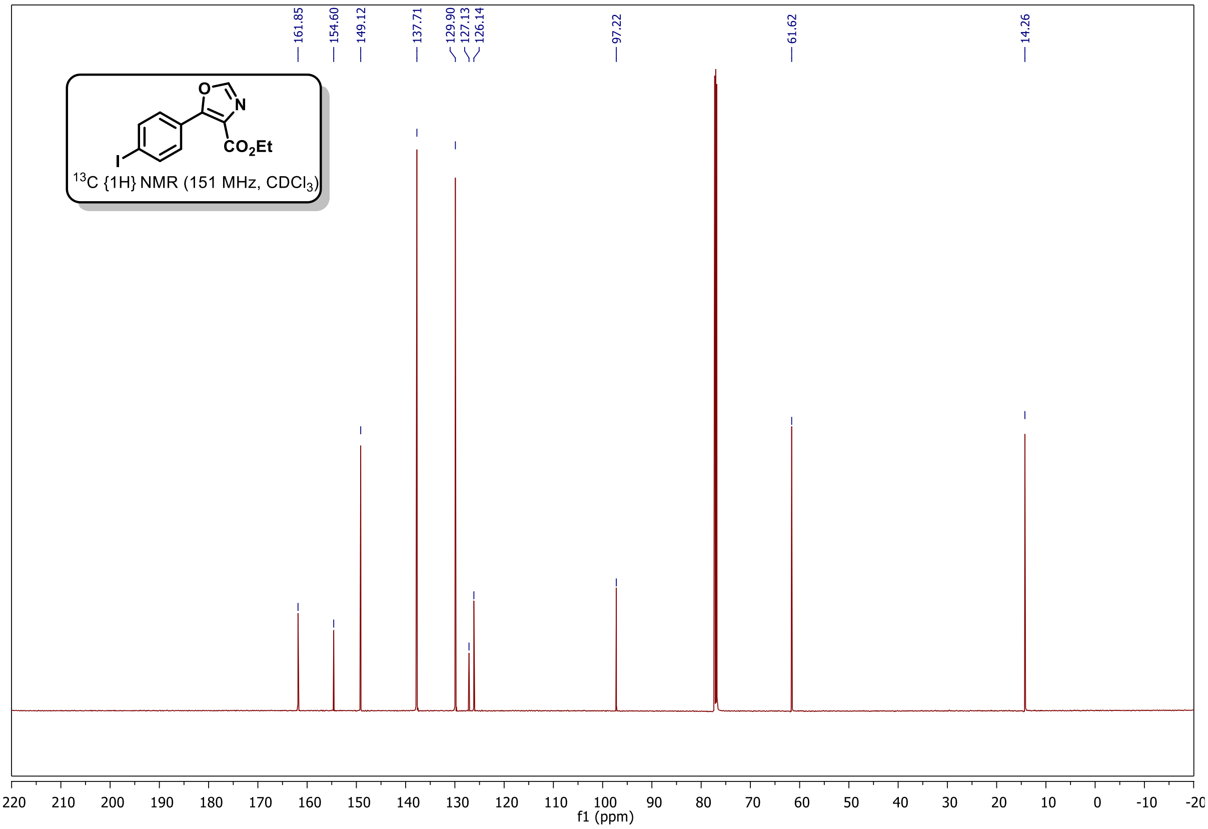

Ethyl 5-(4-bromophenyl)oxazole-4-carboxylate (3ca):

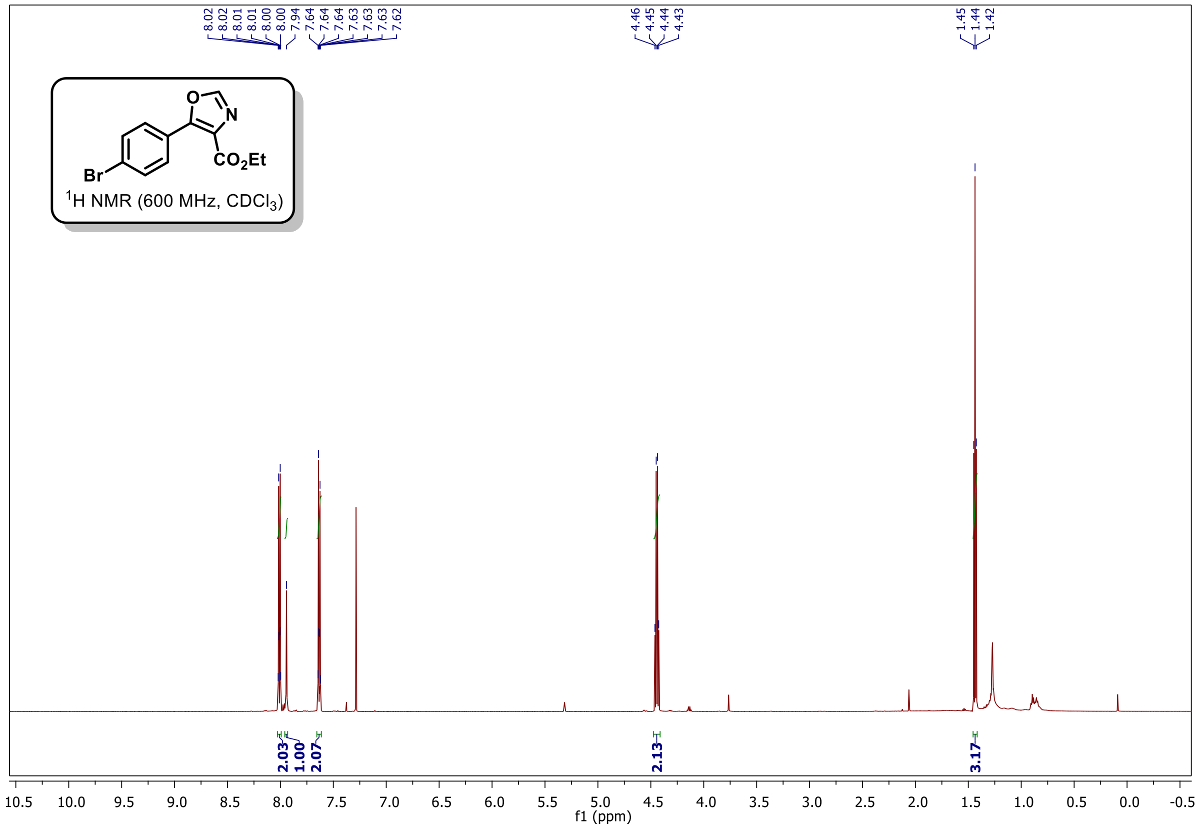

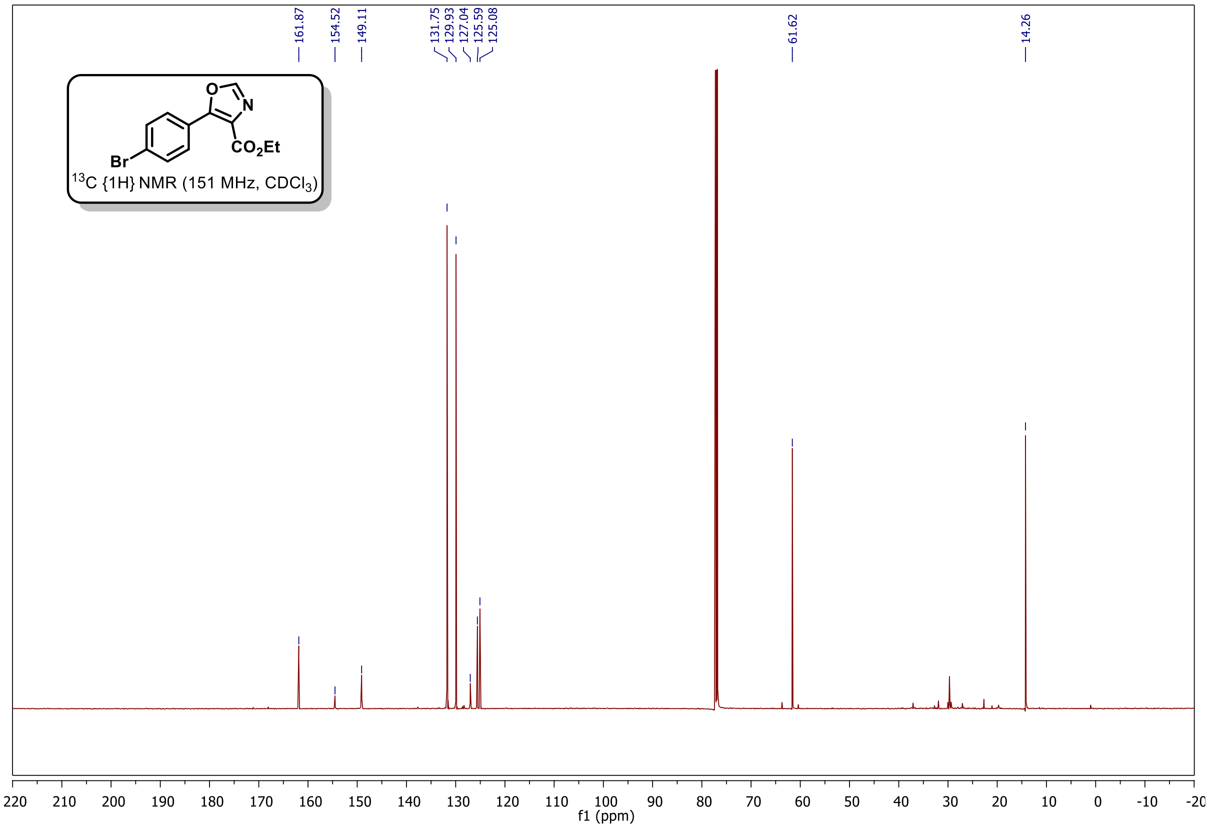

Ethyl 5-(4-nitrophenyl)oxazole-4-carboxylate (3da):

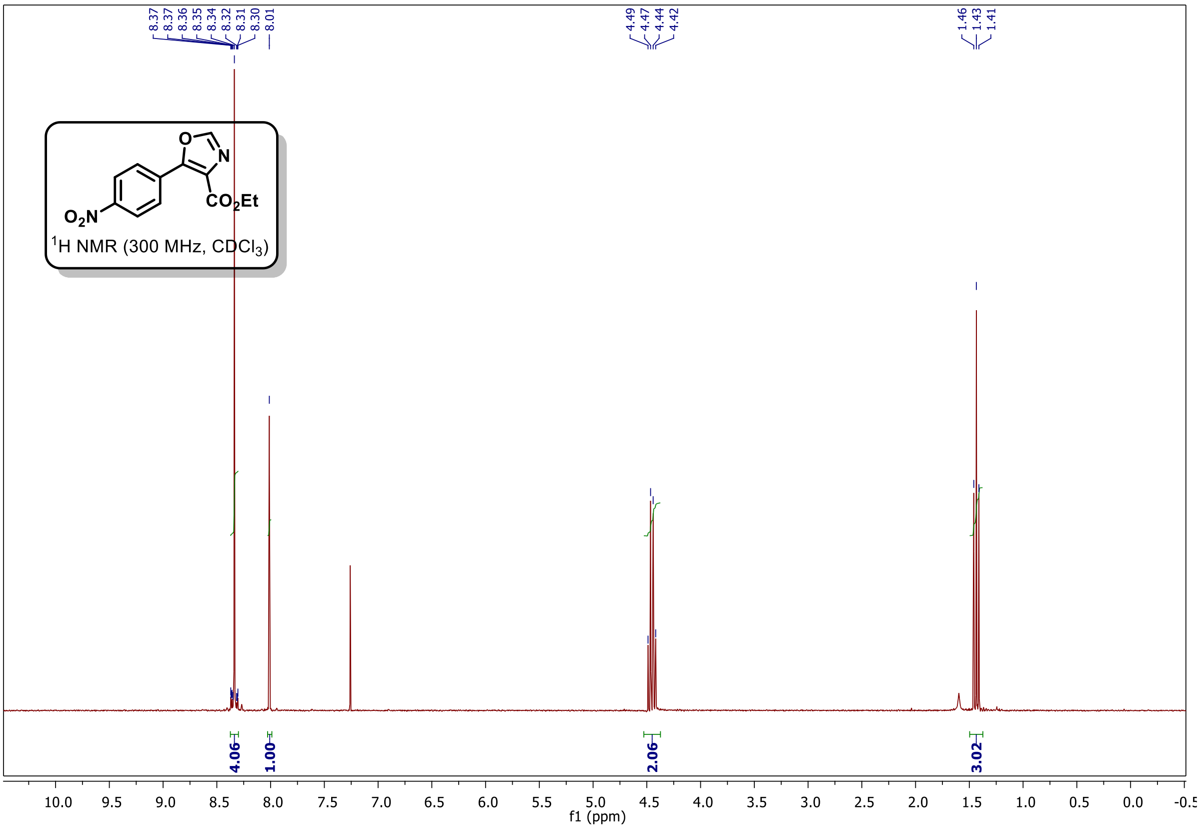

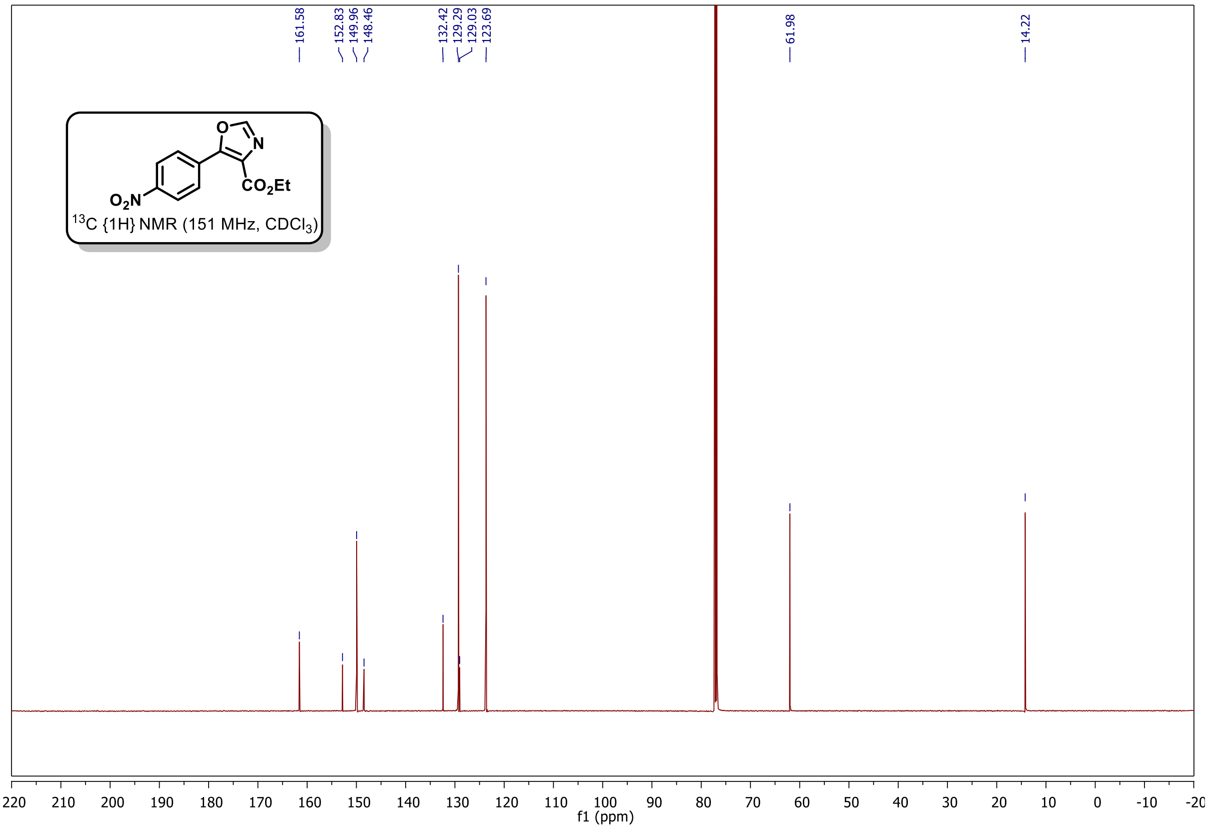

Ethyl 5-benzoyloxazole-4-carboxylate (3ea):

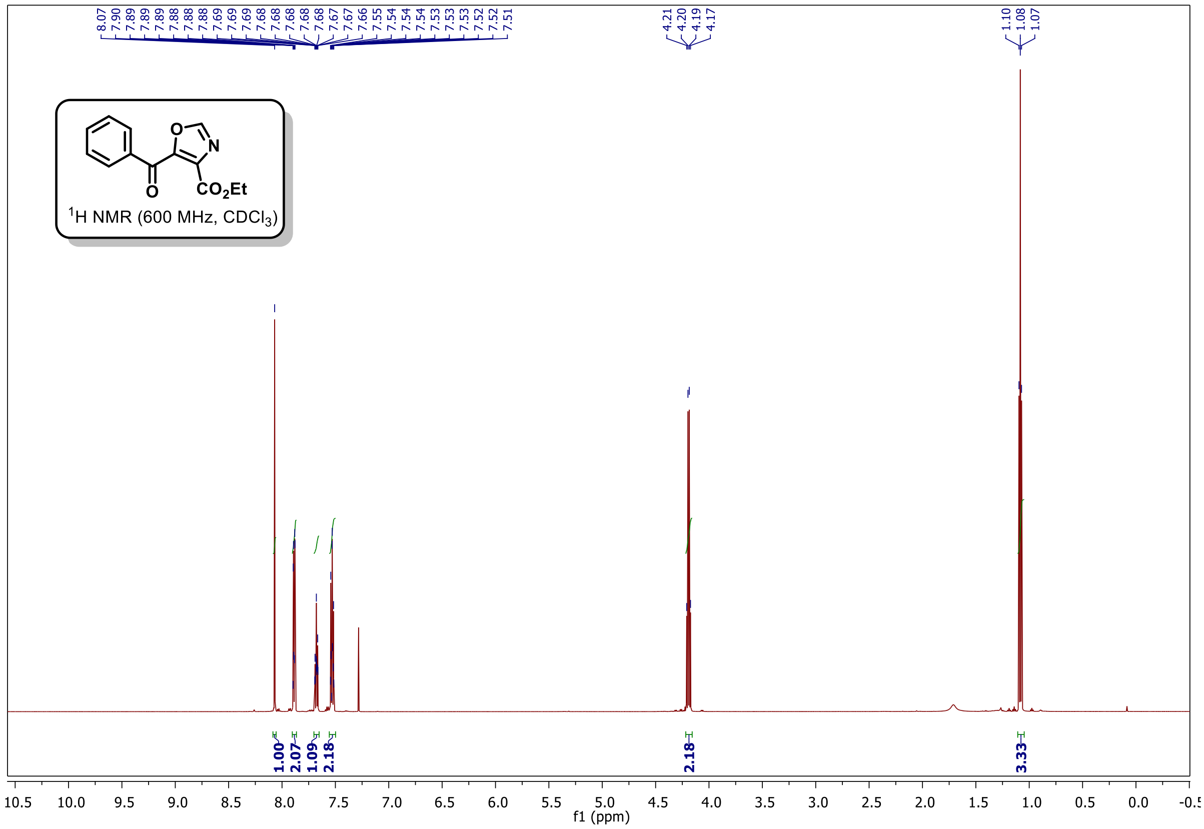

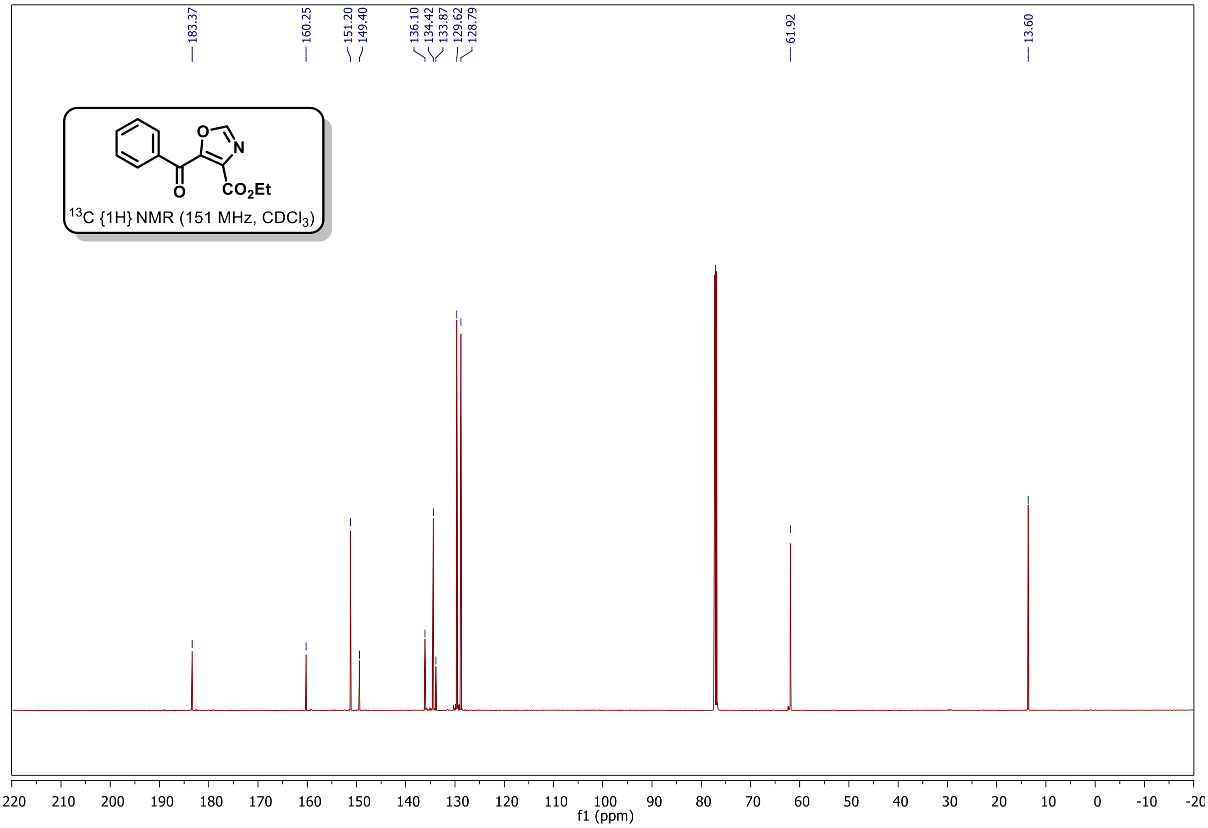

Ethyl 5-(o-tolyl)oxazole-4-carboxylate (3fa):

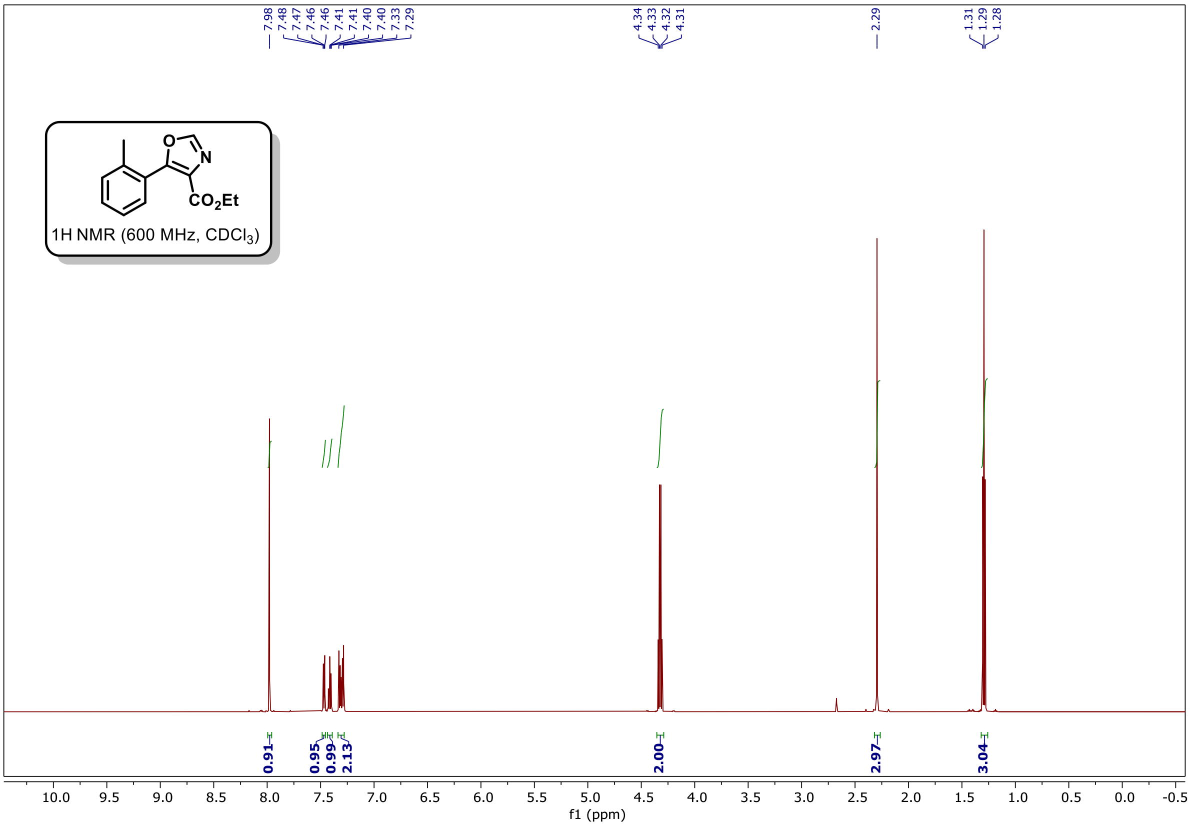

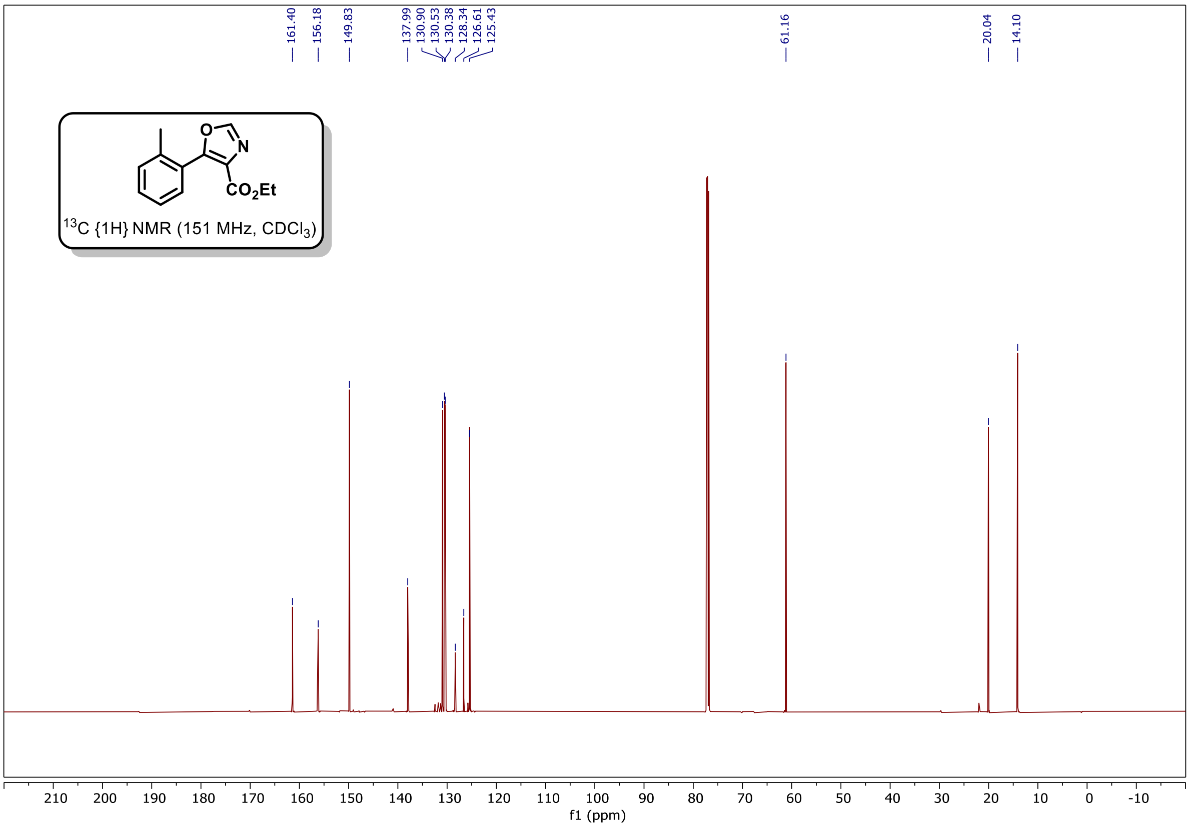

Ethyl 5-phenyloxazole-4-carboxylate (3ga):

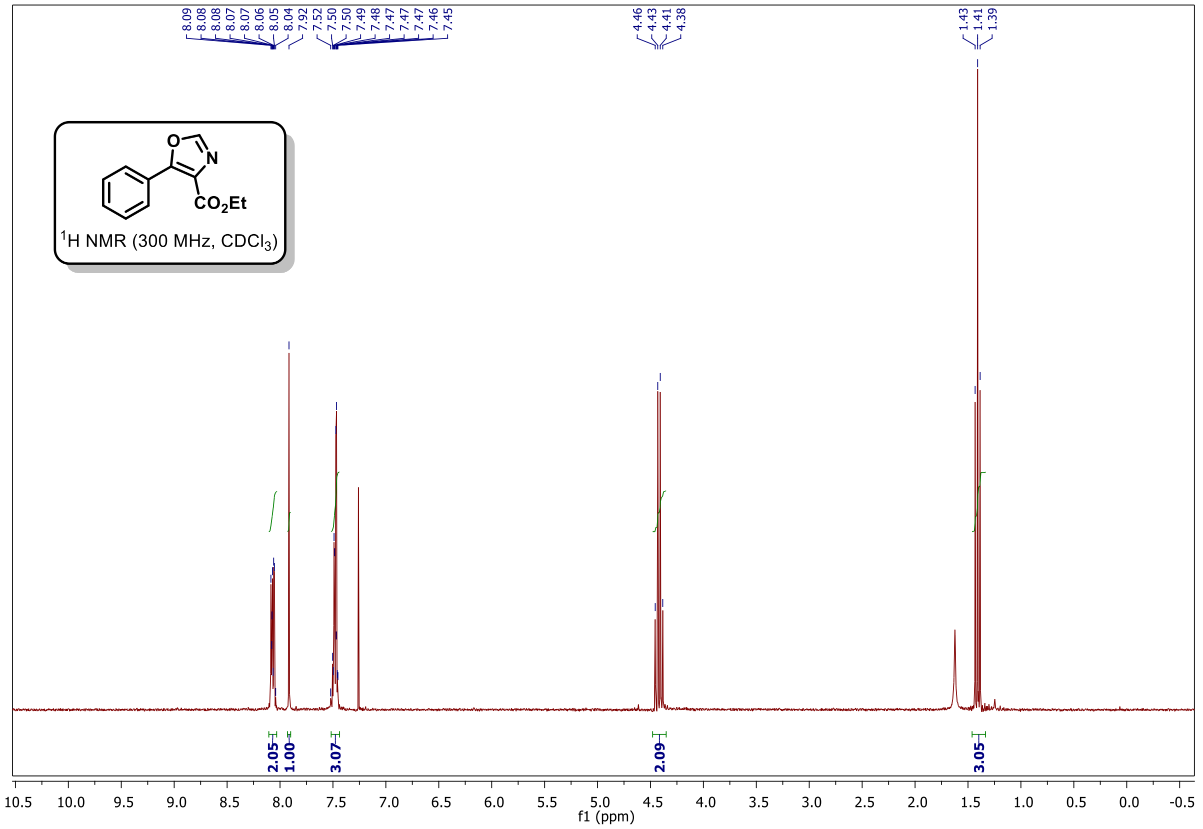

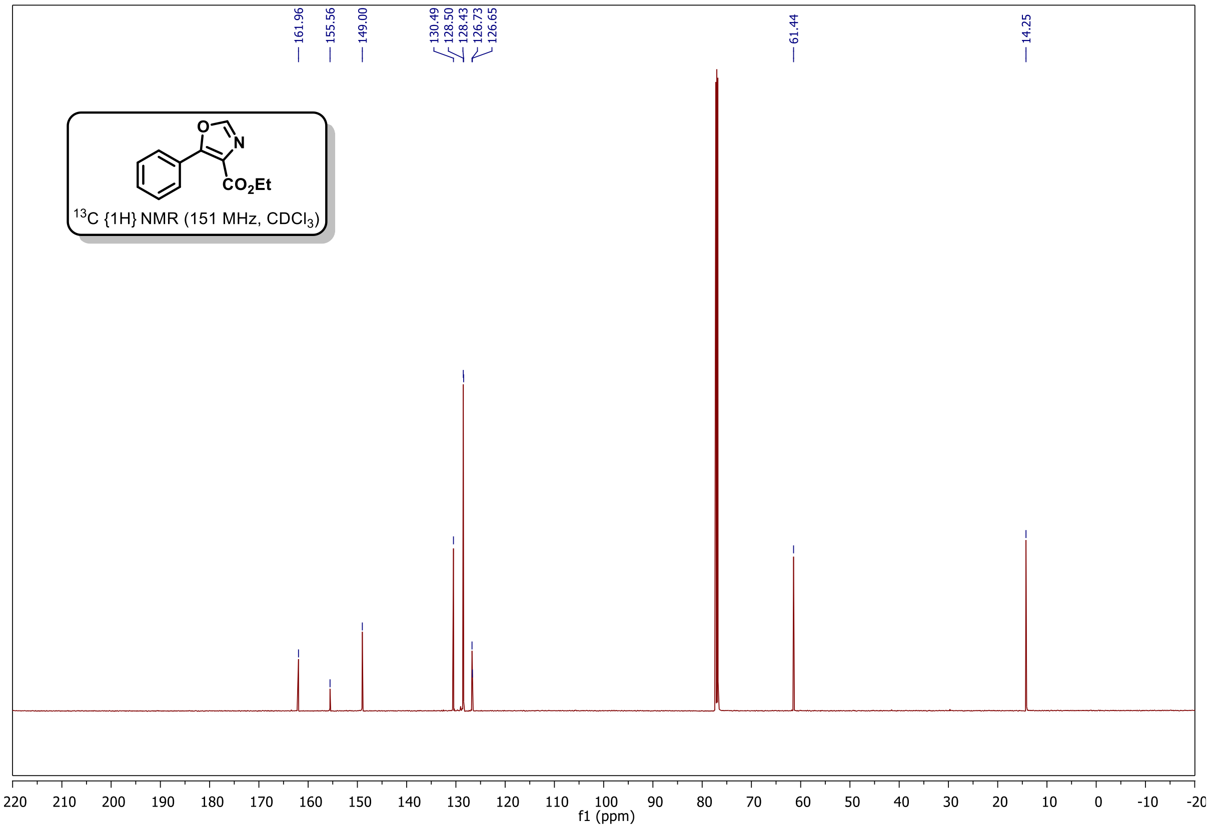

Tert-butyl 5-phenyloxazole-4-carboxylate (3gb):

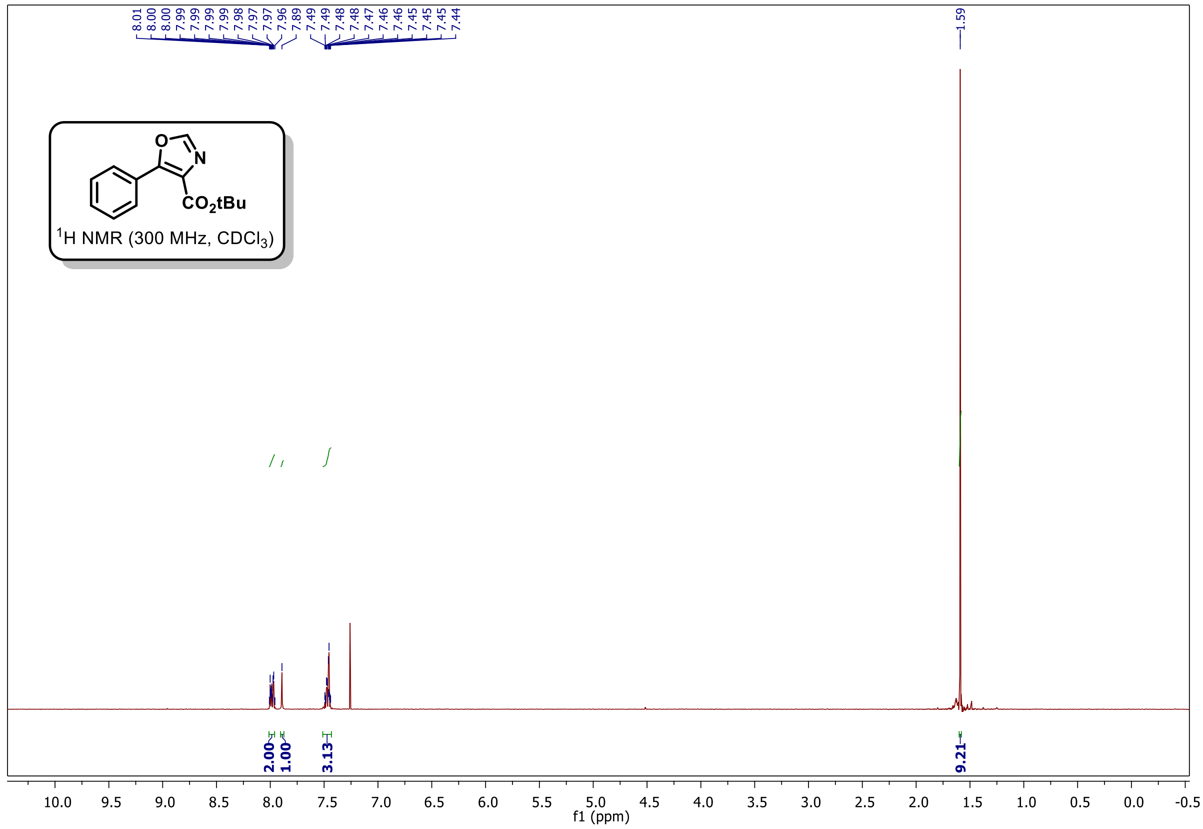

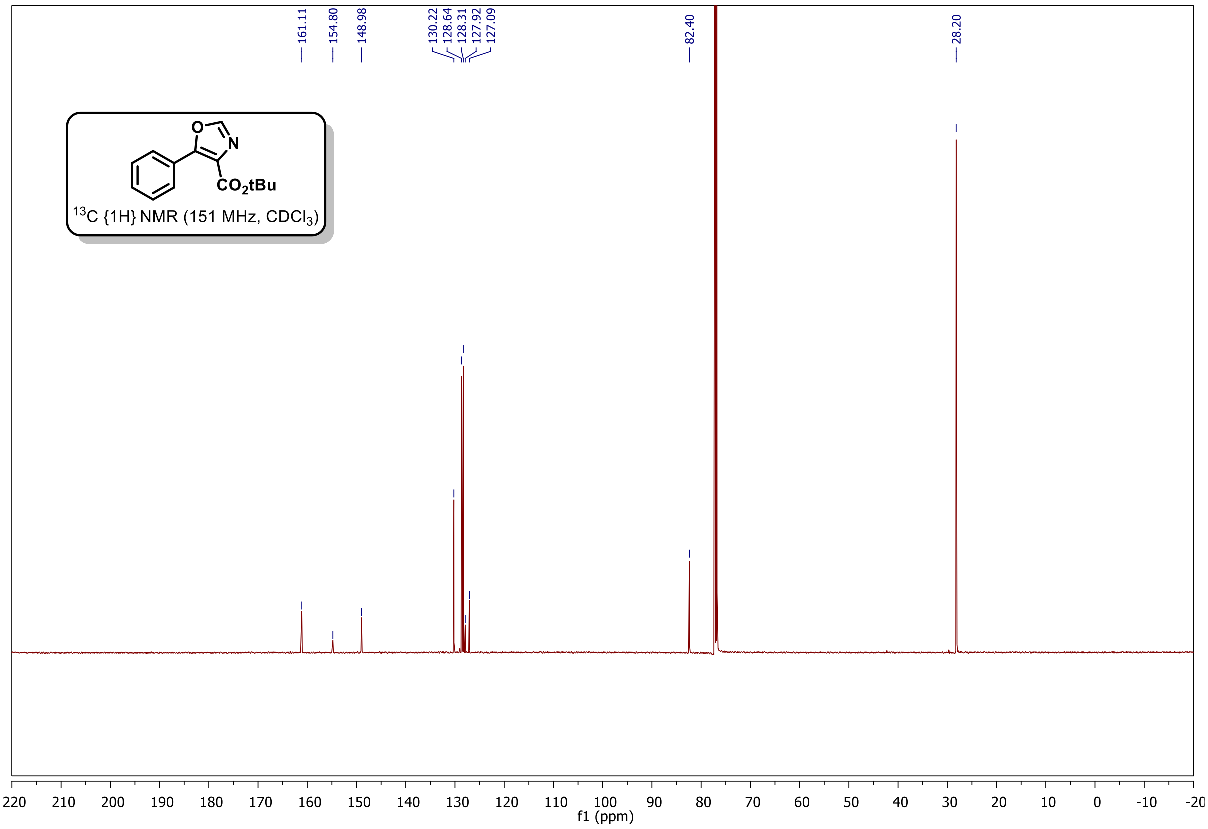

Ethyl 5-(benzo[d][1,3]dioxol-5-yl)oxazole-4-carboxylate (3ha):

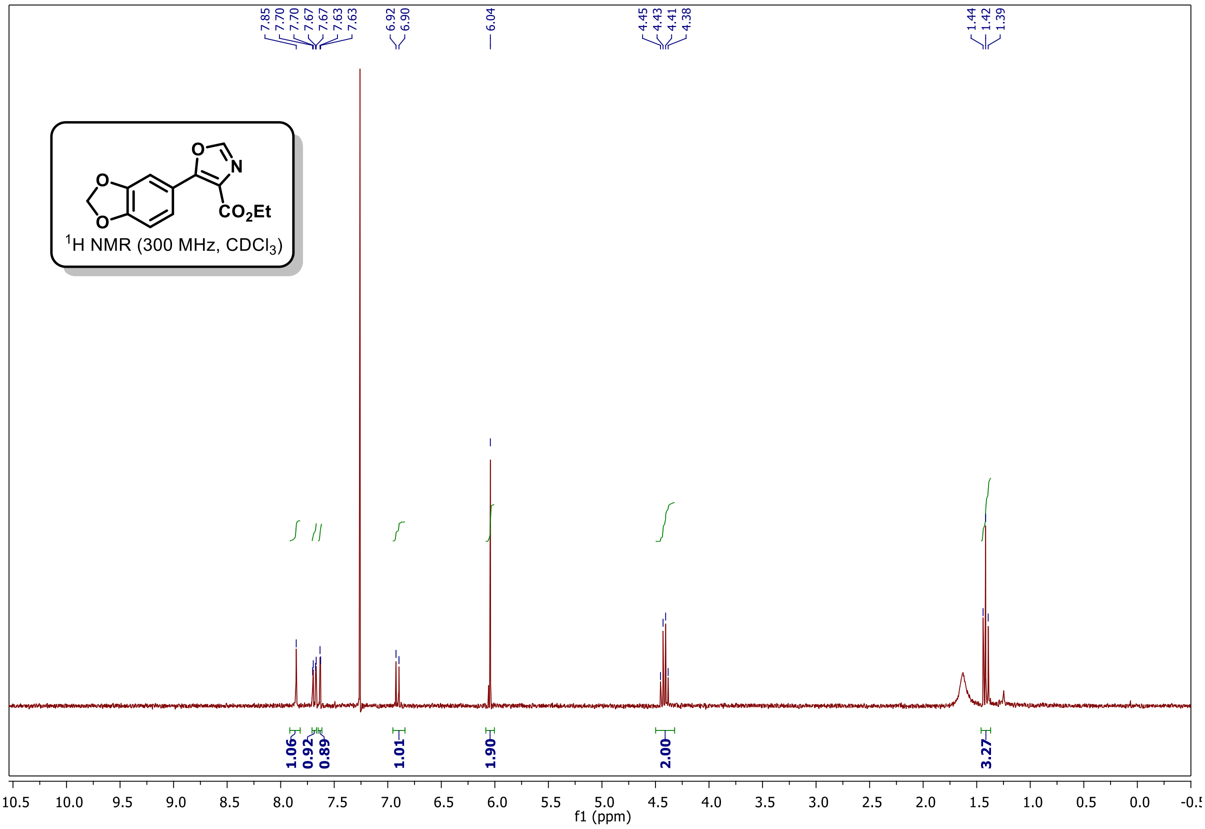

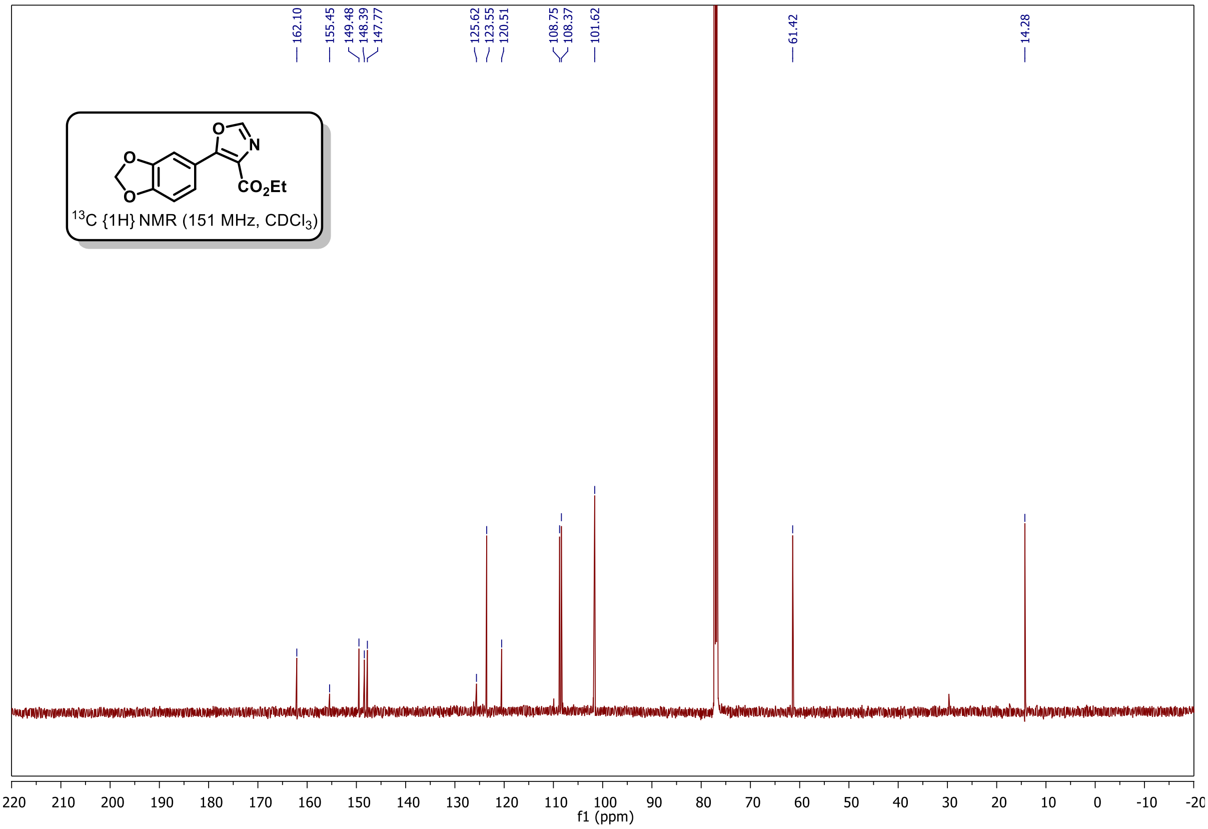

Methyl 5-(4-fluoro-3-(trifluoromethyl)phenyl)oxazole-4-carboxylate (3ia):

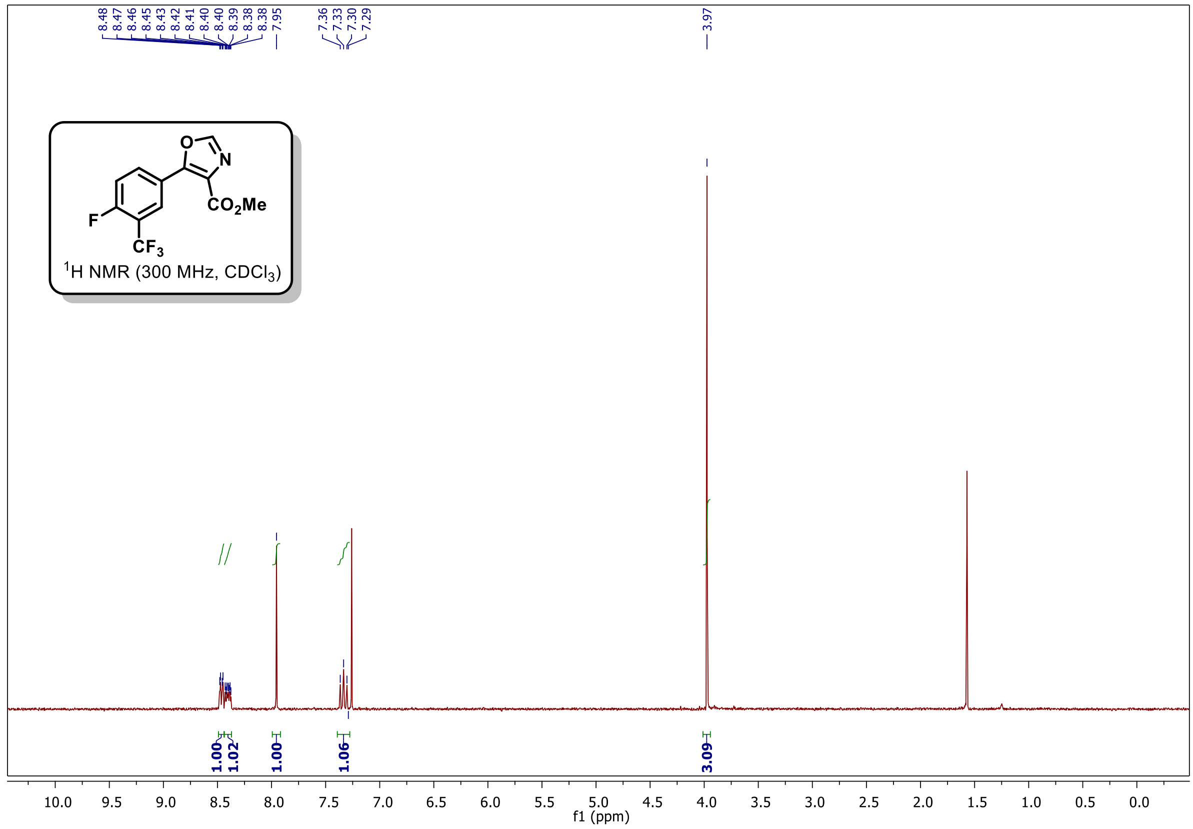

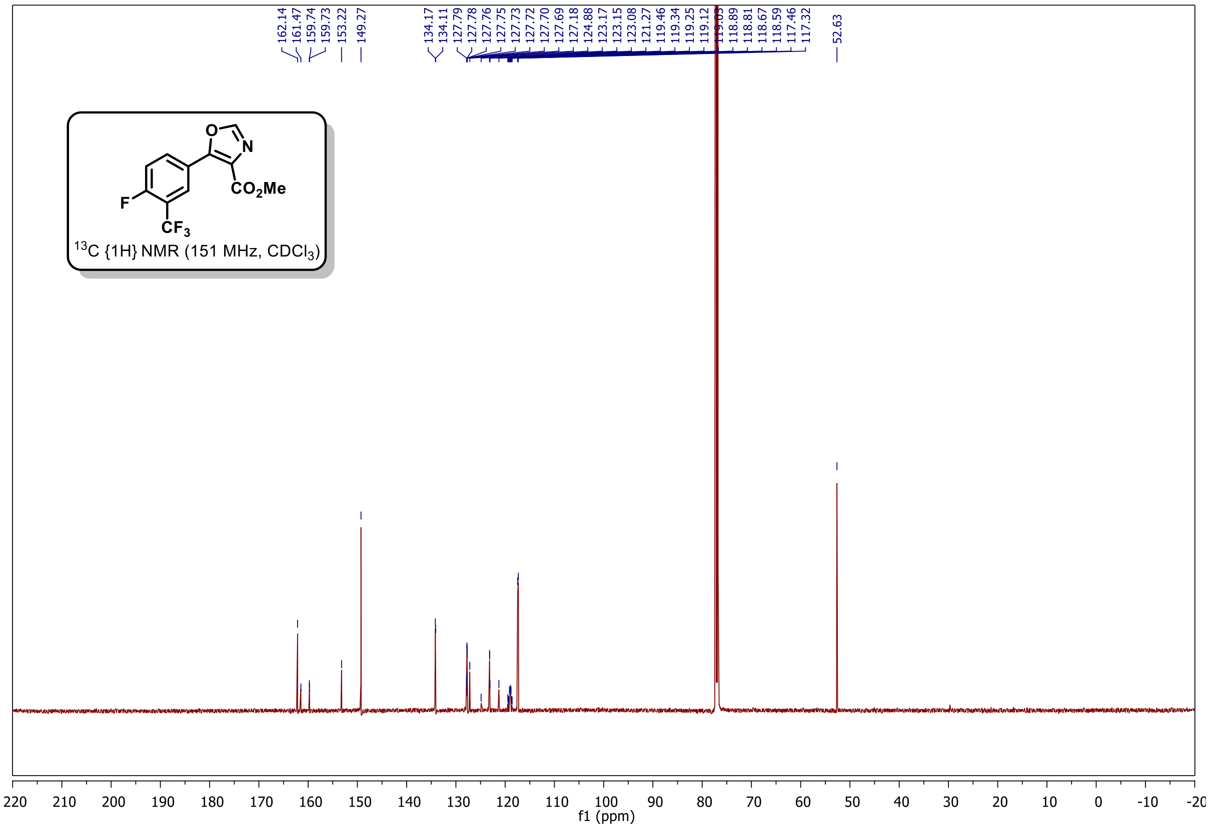

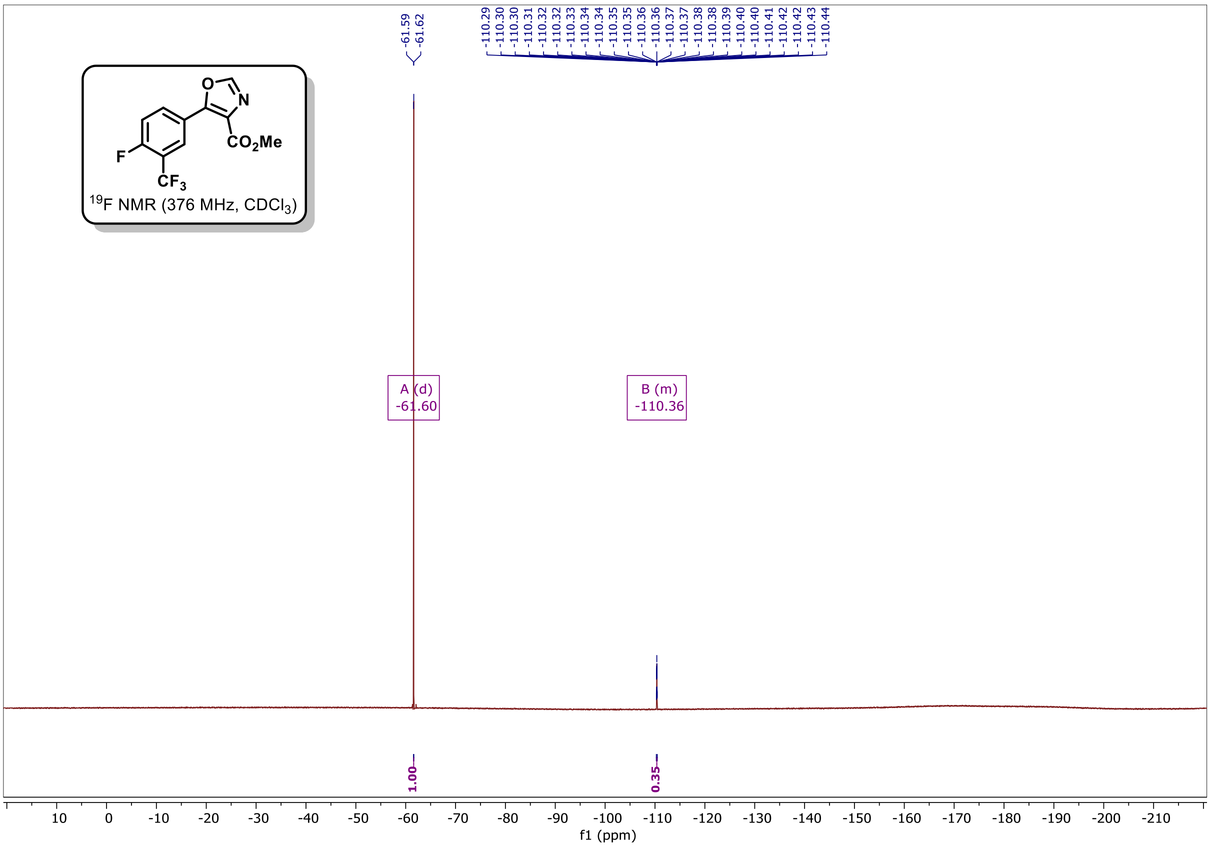

O
N
F
CO2Me
CF3
19F NMR (376 MHz, CDCl3)
-61.59
-61.62
-110.29
-110.30
-110.30
-110.31
-110.32
-110.32
-110.33
-110.34
-110.34
-110.35
-110.35
-110.36
-110.36
-110.37
-110.37
-110.38
-110.38
-110.39
-110.40
-110.40
-110.41
-110.42
-110.42
-110.43
-110.44
A (d)
-61.60
B (m)
-110.36
1.00
0.35
10 0 -10 -20 -30 -40 -50 -60 -70 -80 -90 -100 -110 -120 -130 -140 -150 -160 -170 -180 -190 -200 -210
f1 (ppm)

Ethyl 5-(3-chloropyridin-4-yl)oxazole-4-carboxylate (3ja):

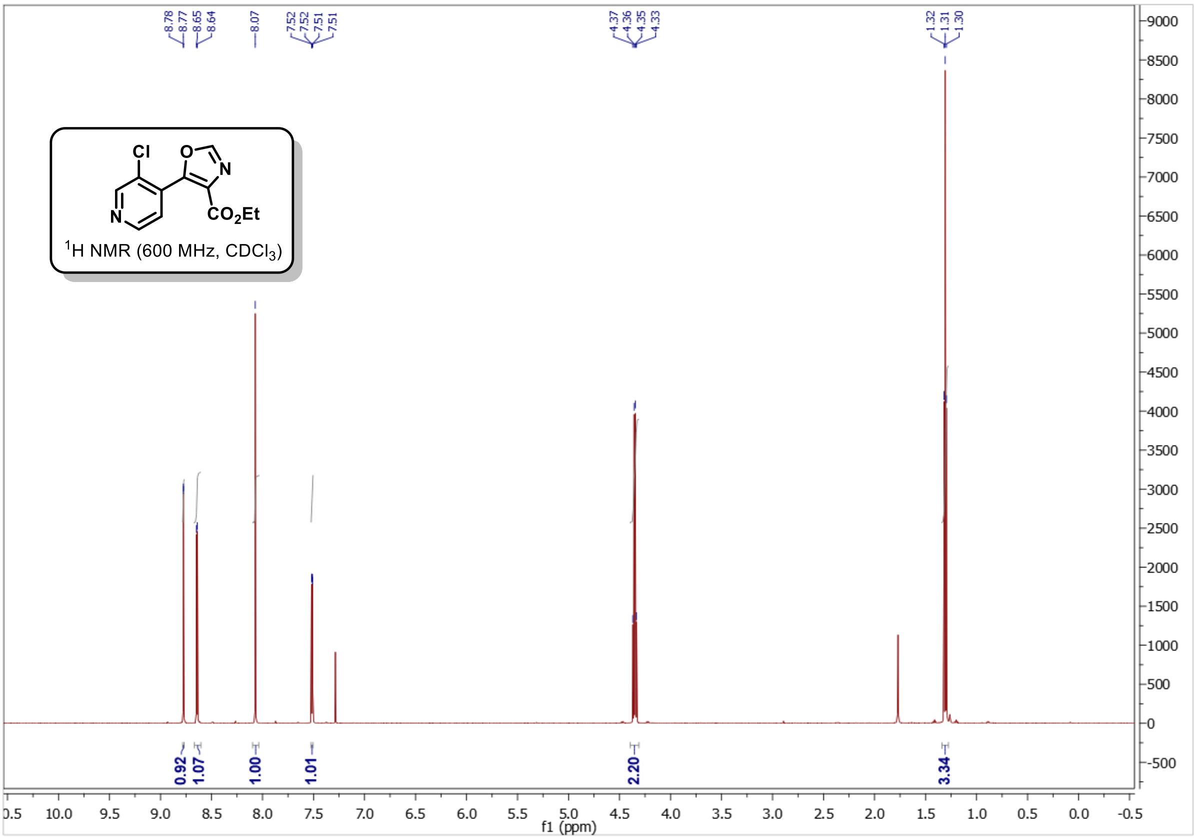

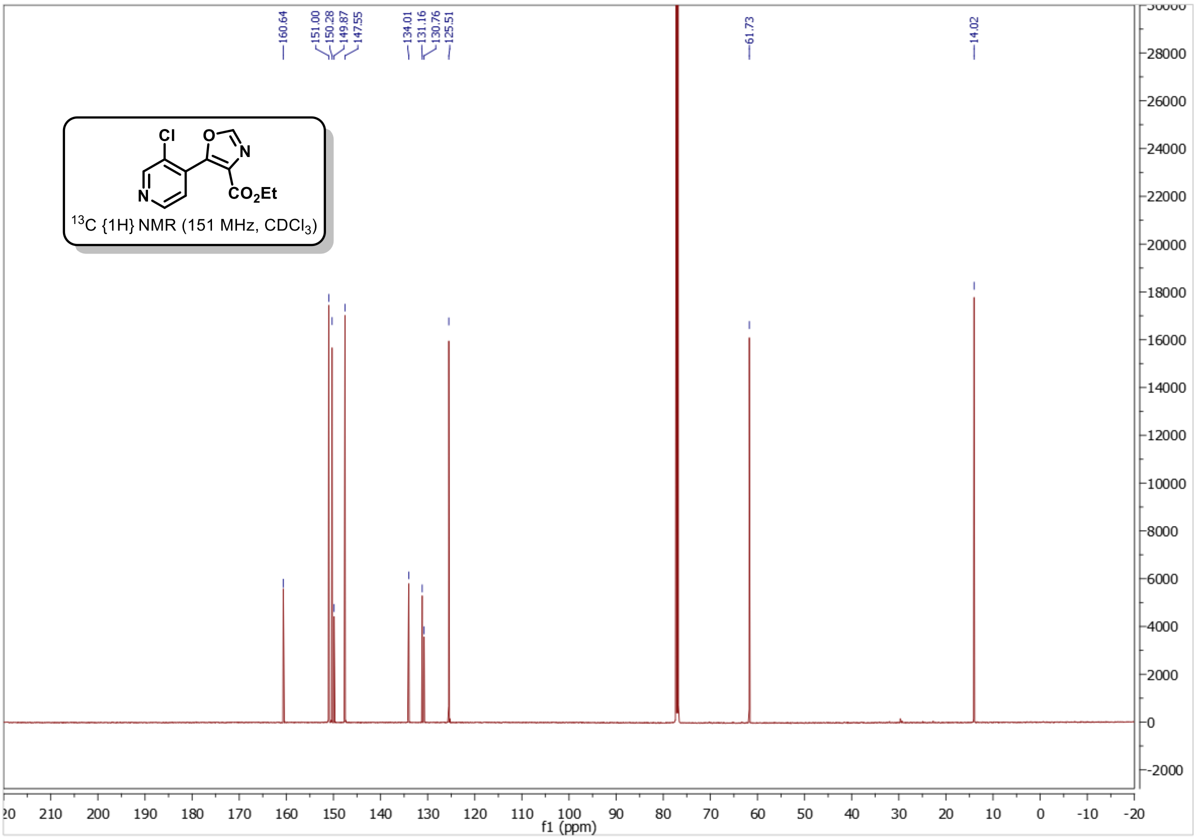

ethyl 5-(6-chloropyridin-3-yl)oxazole-4-carboxylate (3ka):

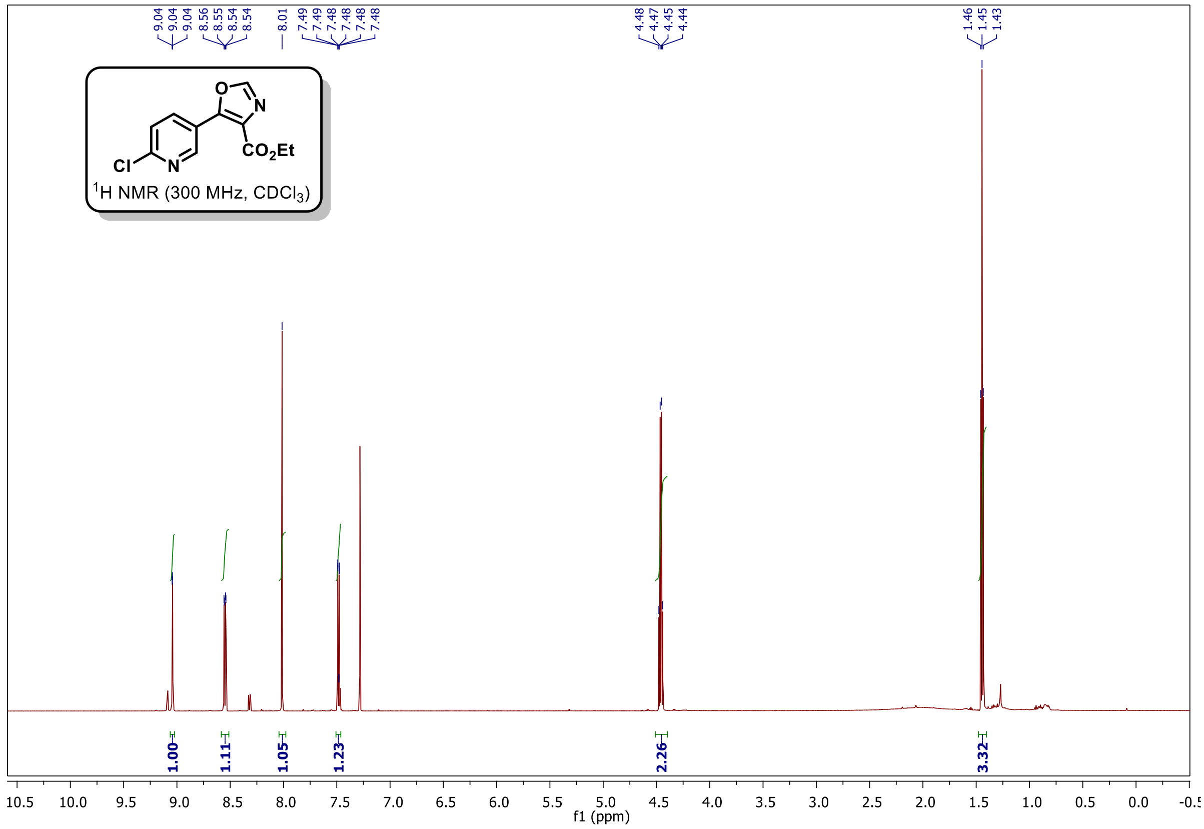

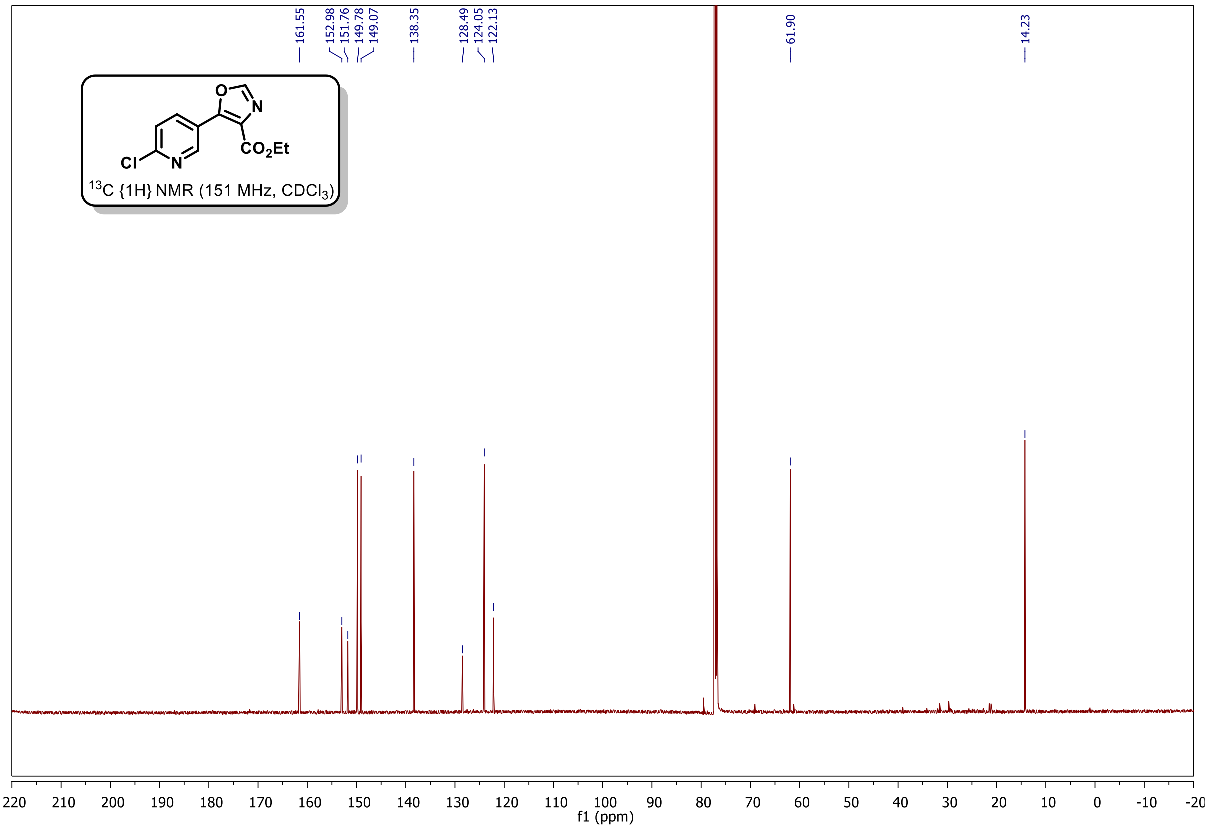

Ethyl 5-(6-fluoropyridin-3-yl)oxazole-4-carboxylate (3la):

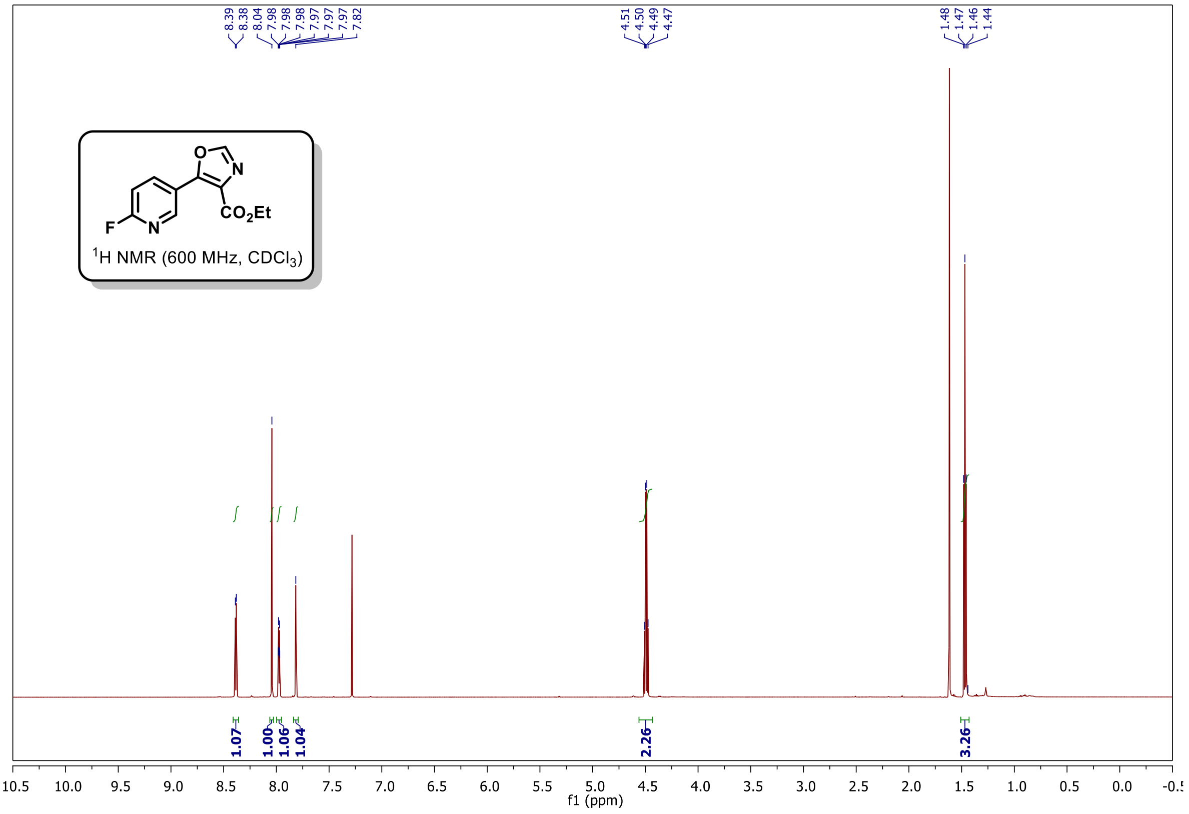

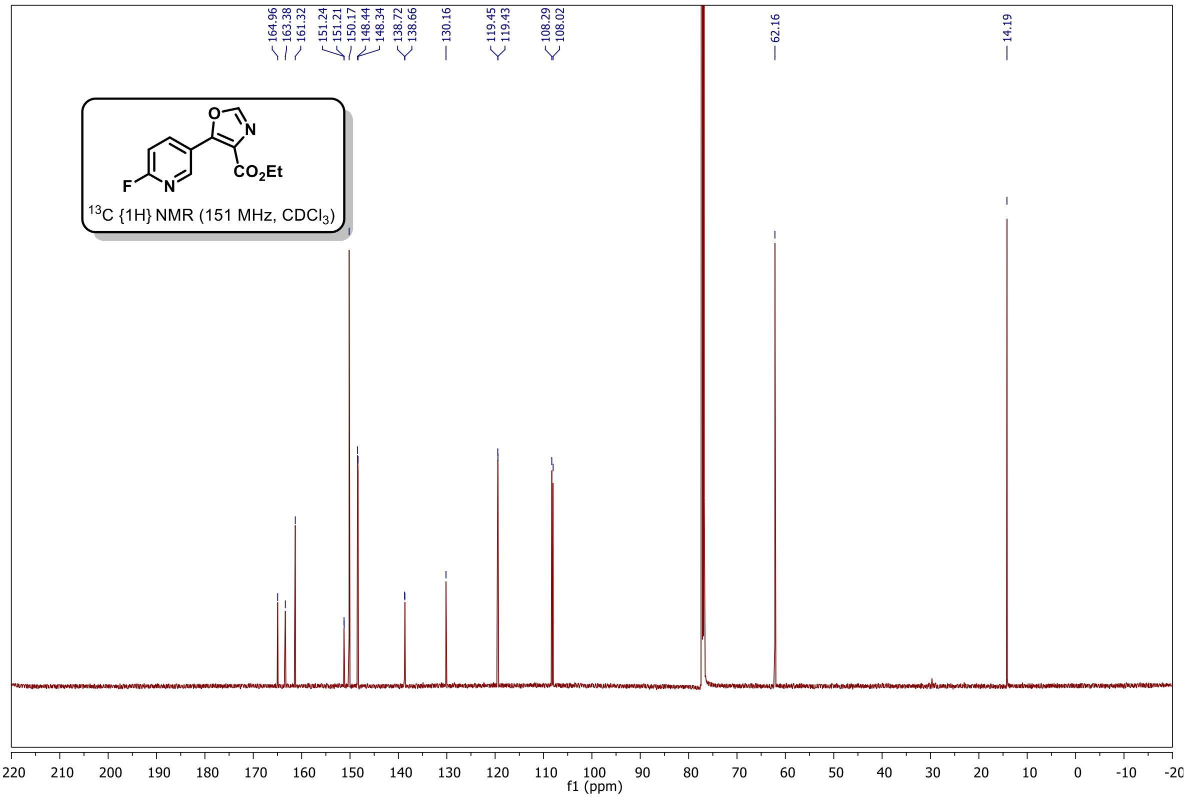

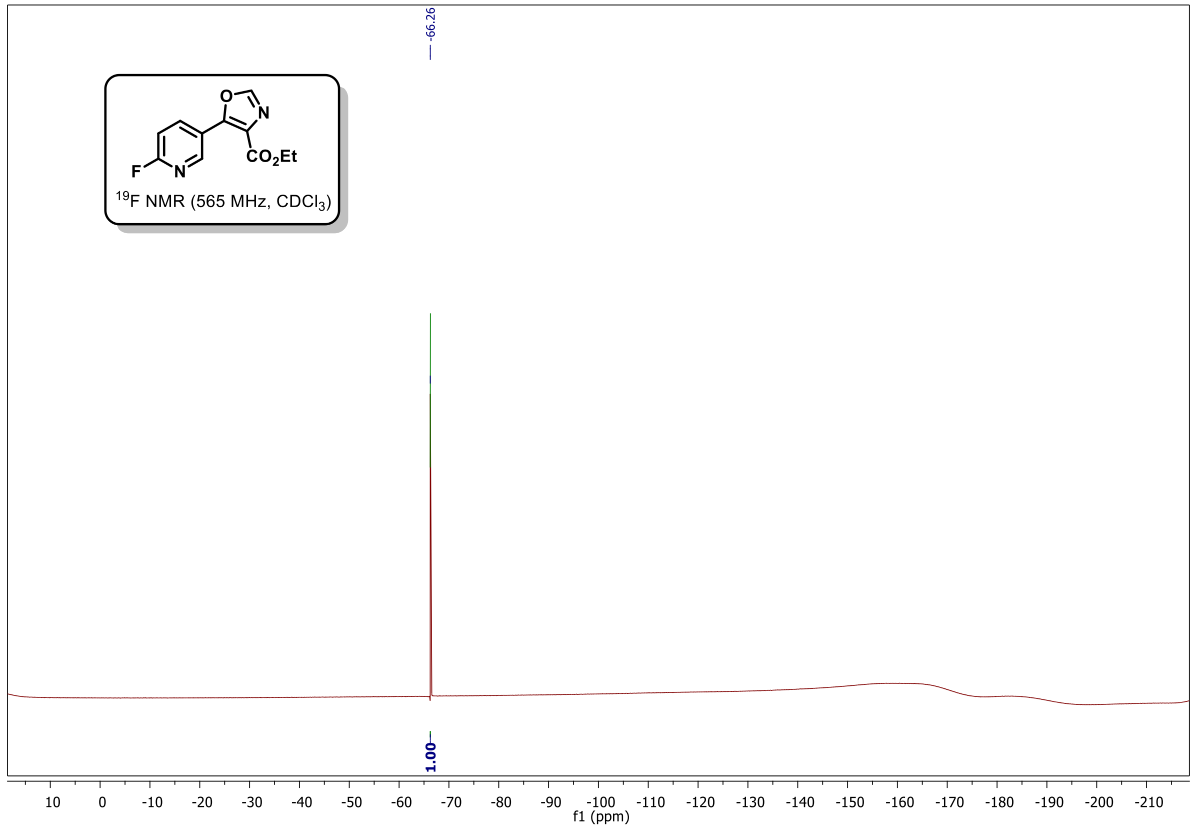
-66.26
F
N
O
N
CO2Et
19F NMR (565 MHz, CDCl3)
1.00
10 0 -10 -20 -30 -40 -50 -60 -70 -80 -90 -100 -110 -120 -130 -140 -150 -160 -170 -180 -190 -200 -210
f1 (ppm)

Ethyl 5-(6-(trifluoromethyl)pyridin-3-yl)oxazole-4-carboxylate (3ma):

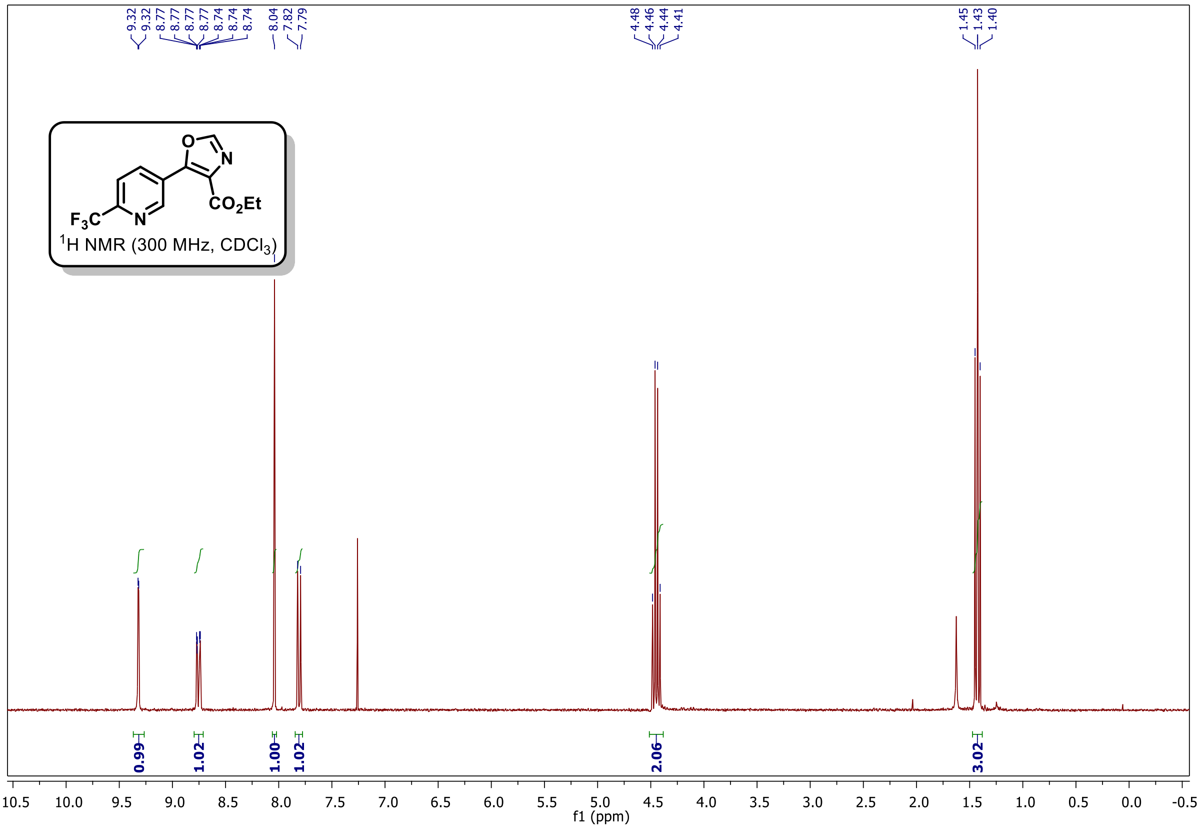

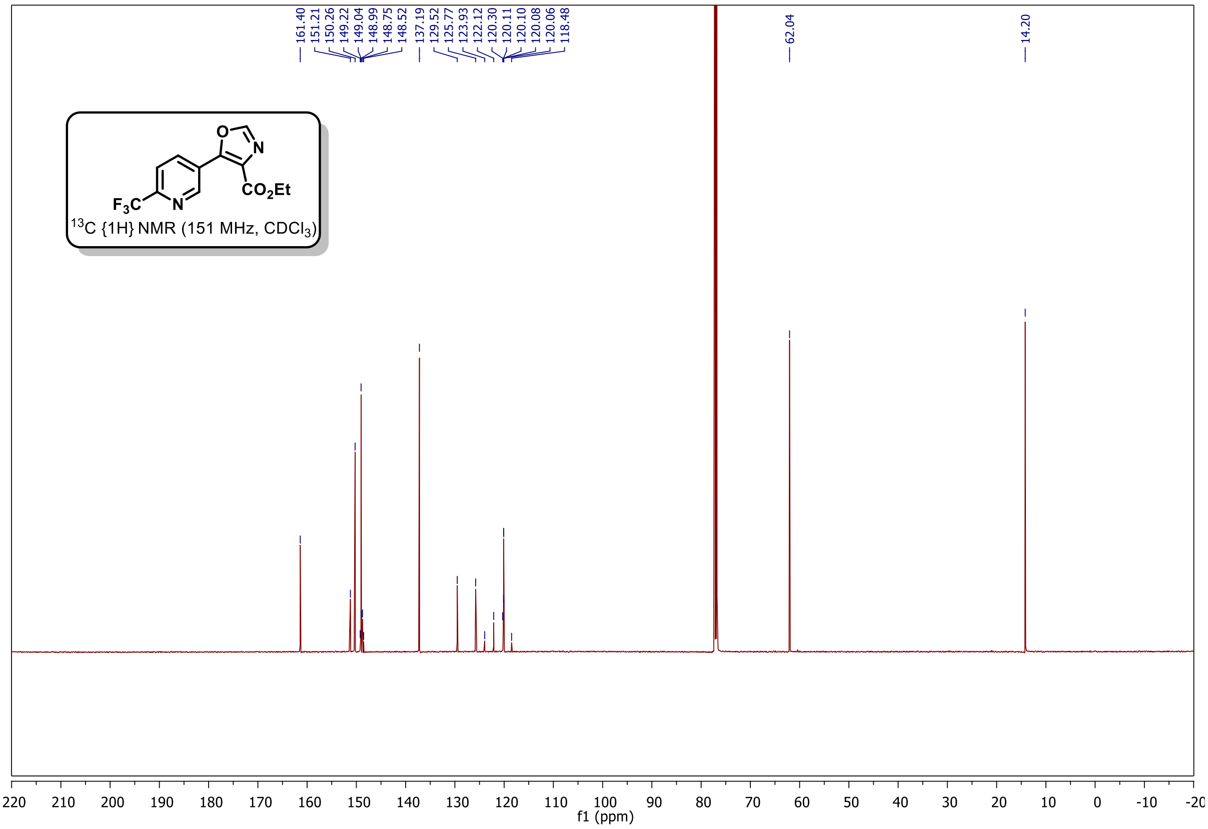

Methyl 5-(2-fluoropyridin-4-yl)oxazole-4-carboxylate (3nc):

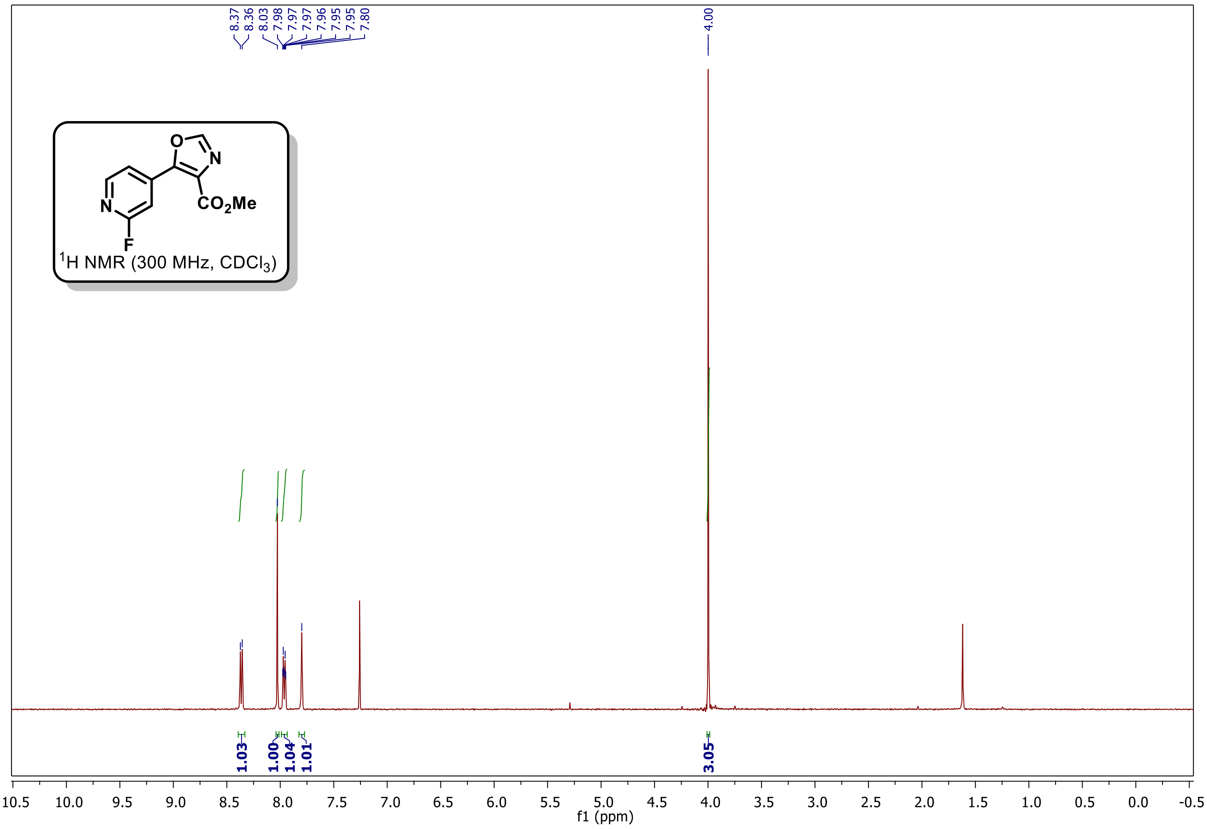

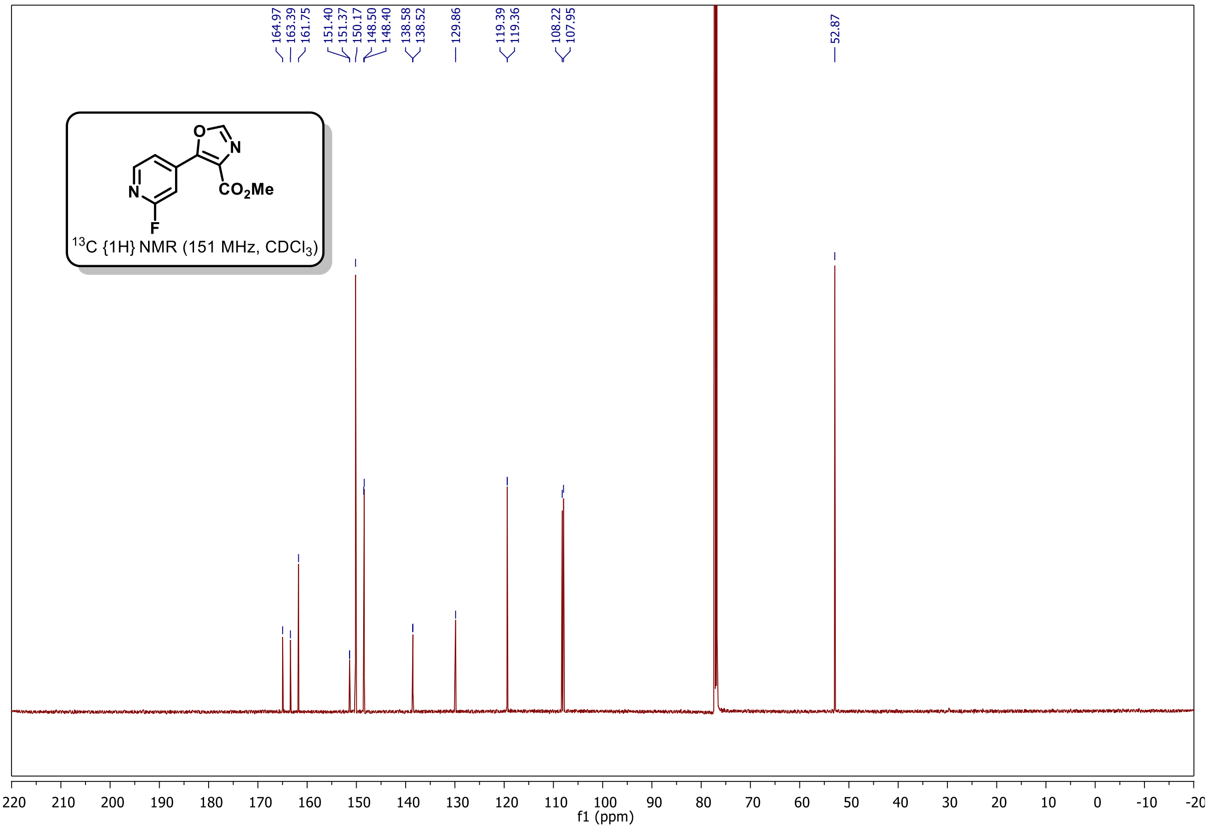

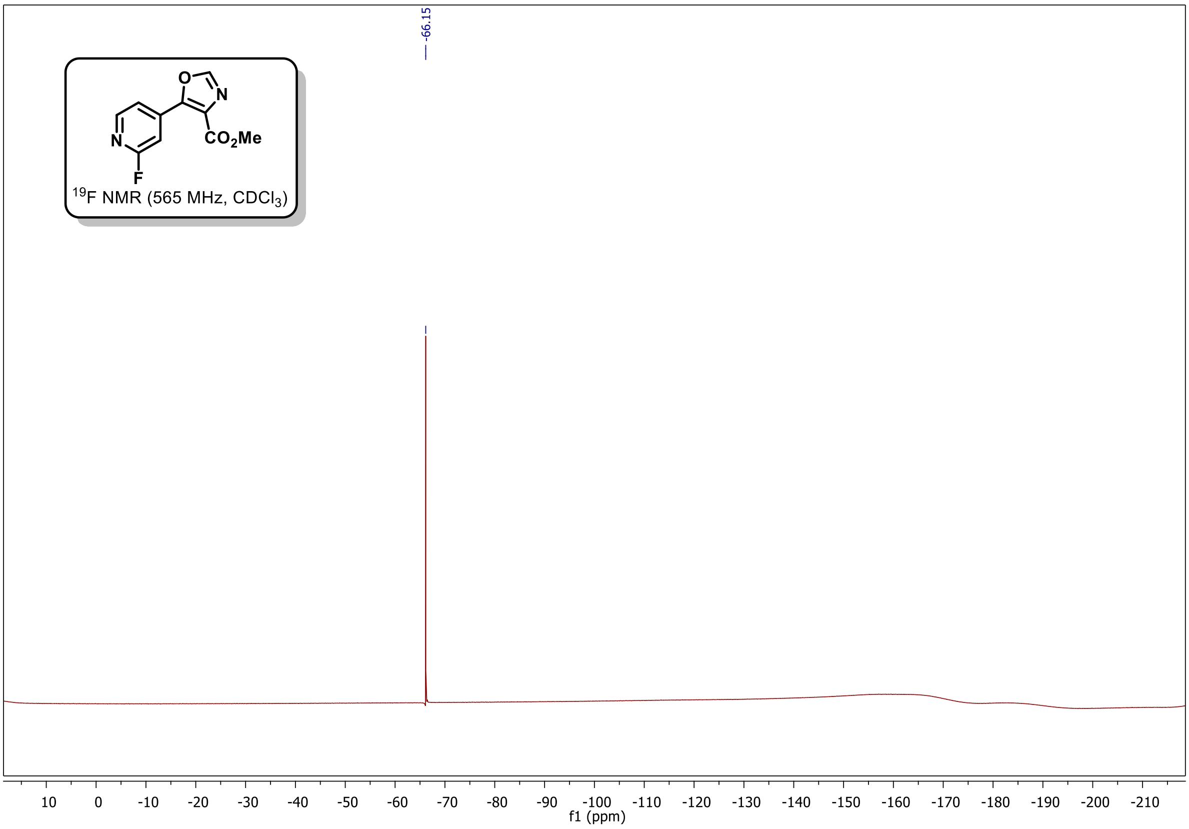
-66.15
CO2Me
^{19}F NMR (565 MHz, CDCl$_3$)
10 0 -10 -20 -30 -40 -50 -60 -70 -80 -90 -100 -110 -120 -130 -140 -150 -160 -170 -180 -190 -200 -210
f1 (ppm)

Ethyl 5-(2-(trifluoromethyl)pyridin-3-yl)oxazole-4-carboxylate (3oa):

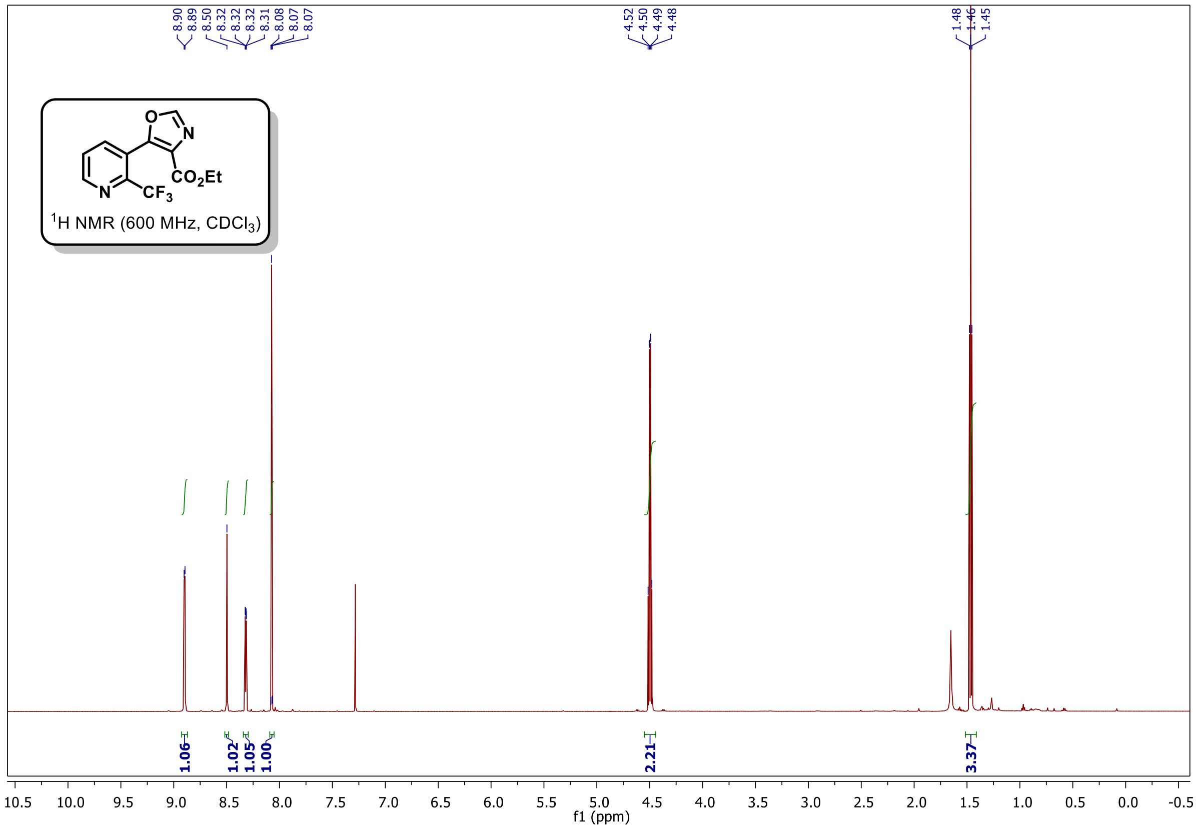

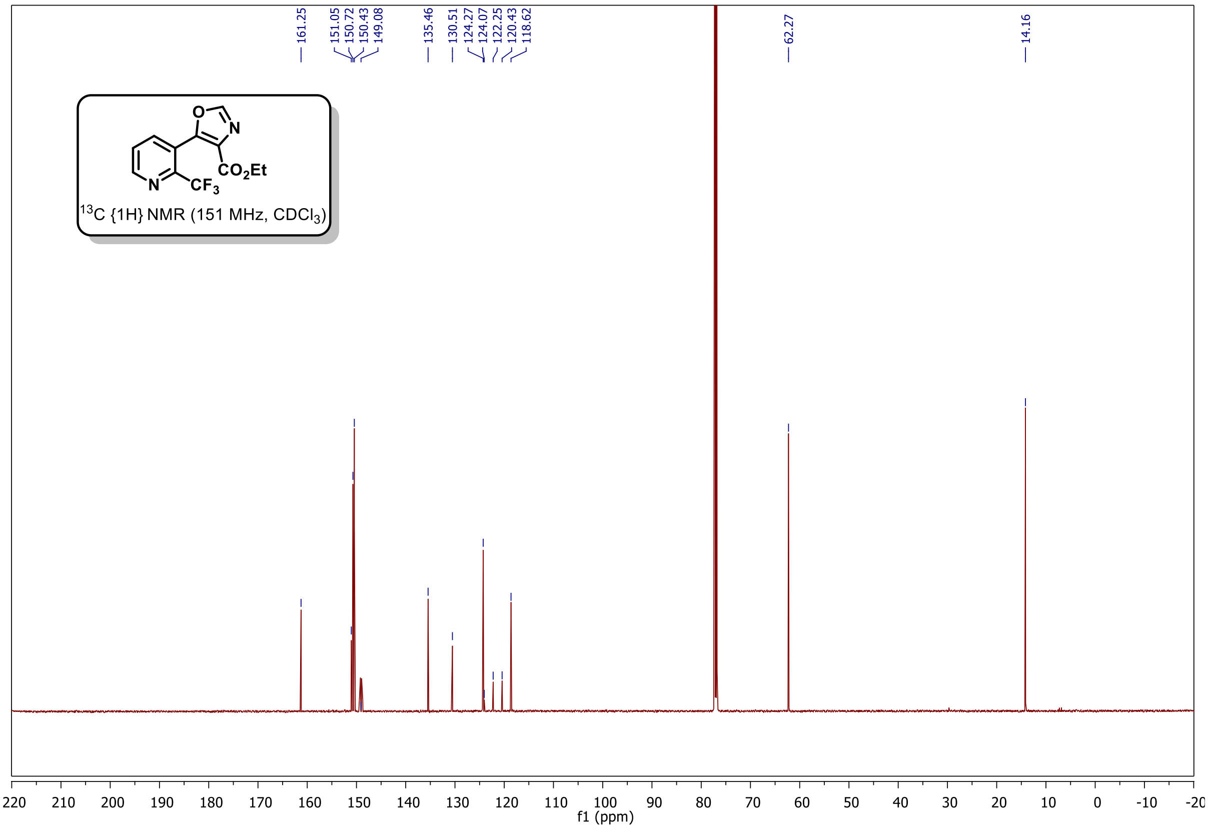

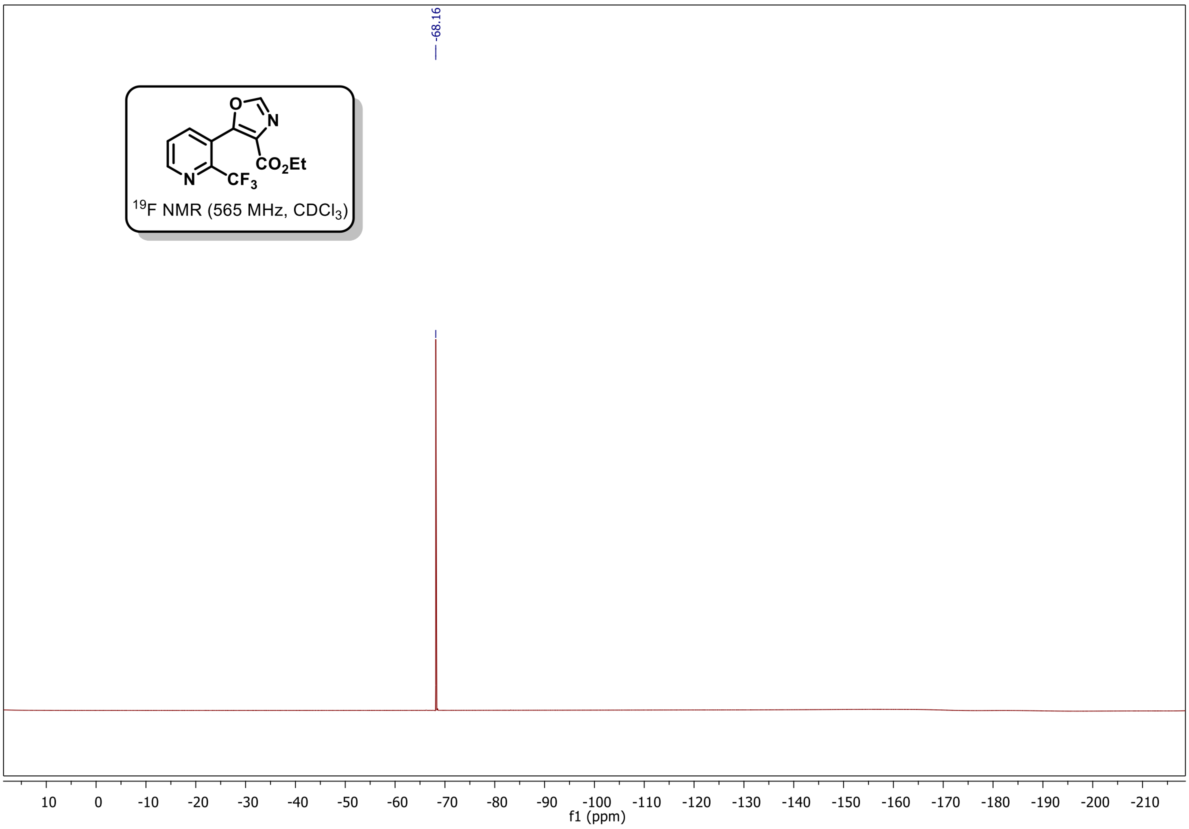
-68.16
CO2Et
CF3
19F NMR (565 MHz, CDCl3)
10 0 -10 -20 -30 -40 -50 -60 -70 -80 -90 -100 -110 -120 -130 -140 -150 -160 -170 -180 -190 -200 -210
f1 (ppm)

Ethyl 5-(3-chloroisoxazol-5-yl)oxazole-4-carboxylate (3pa):

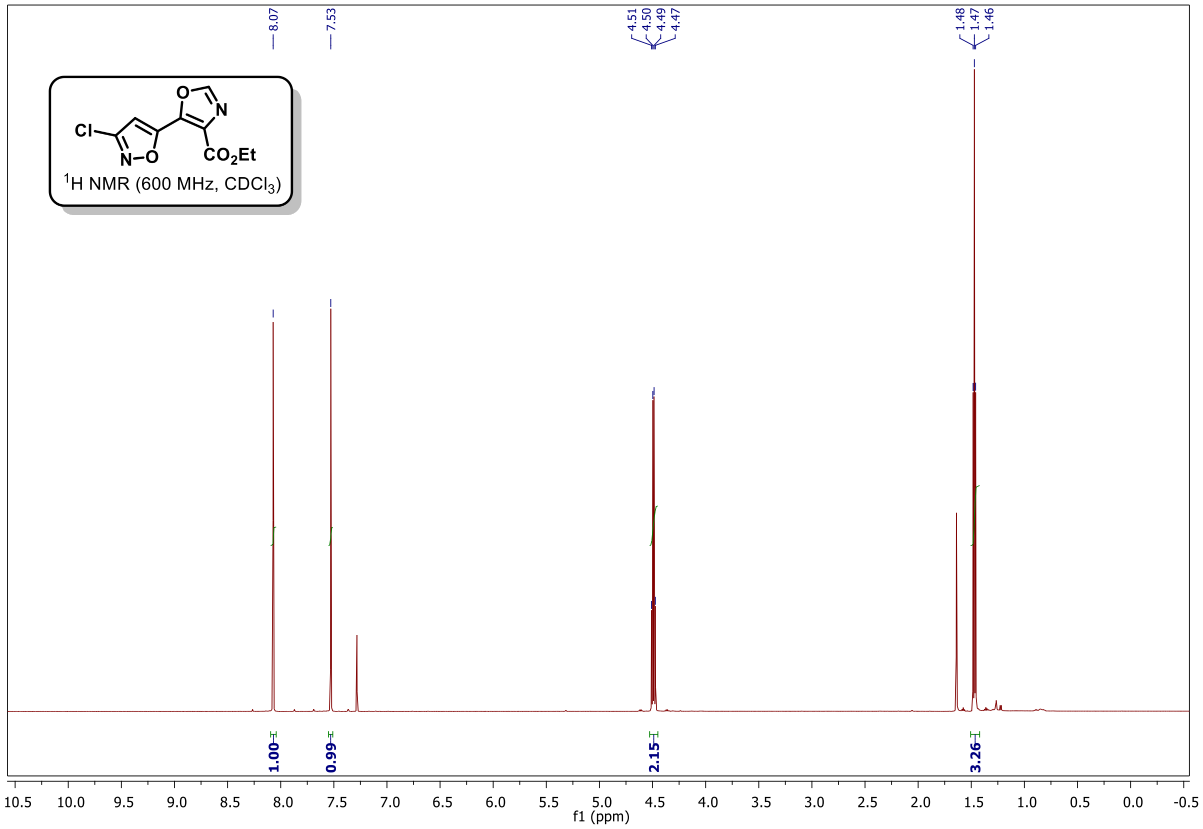

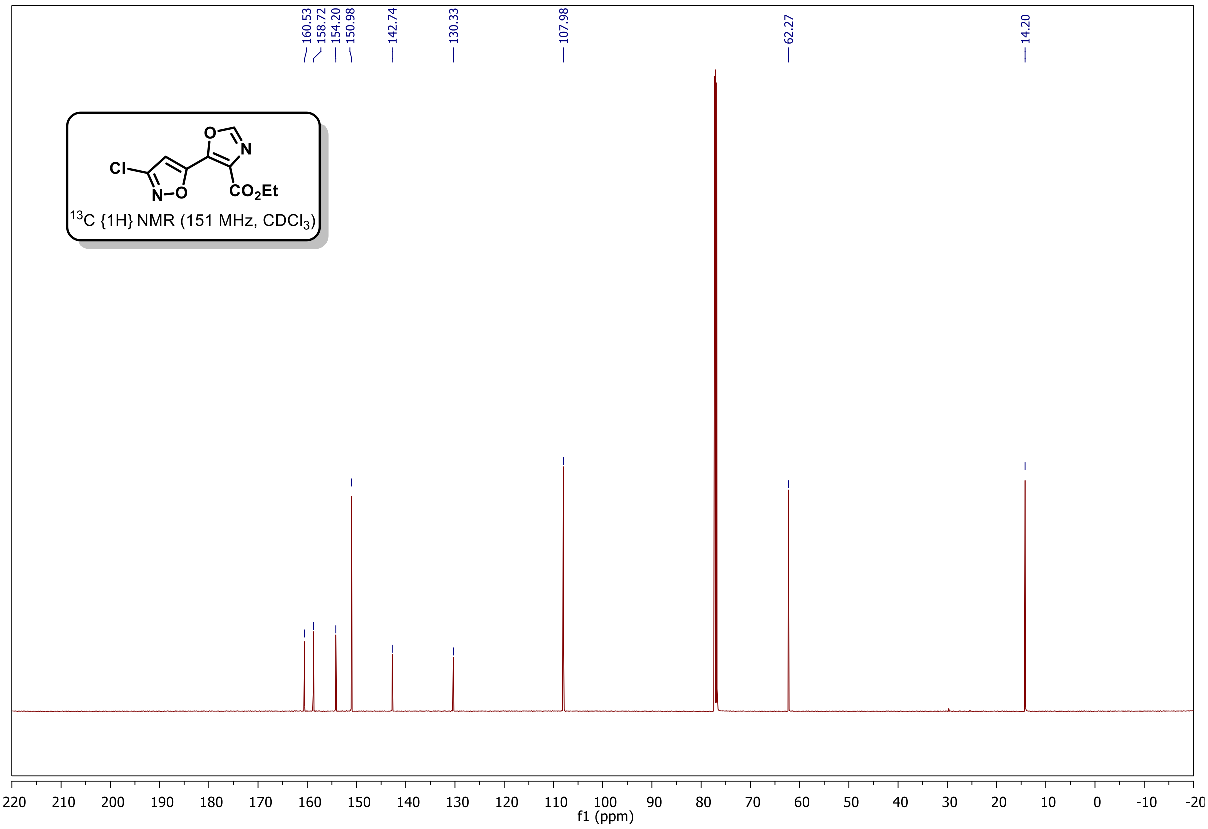

Ethyl 5-(thiophen-2-yl)oxazole-4-carboxylate (3qa):

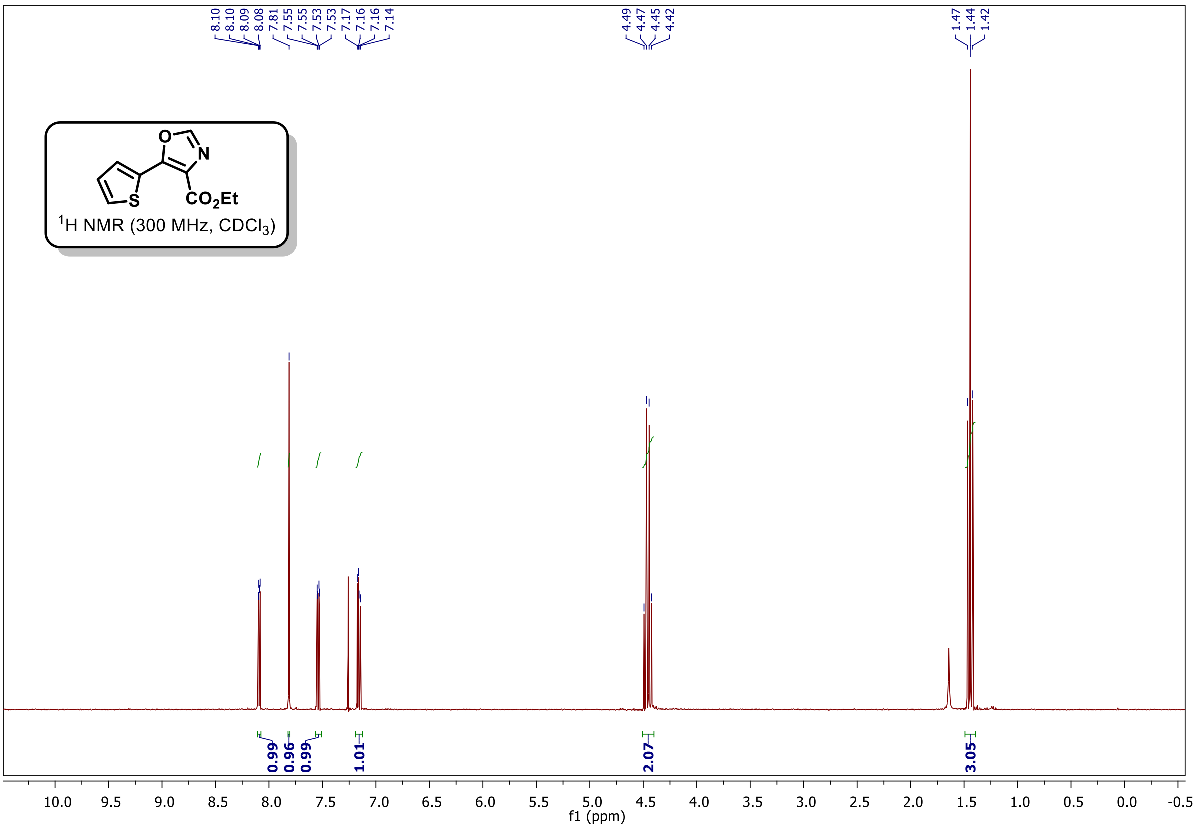

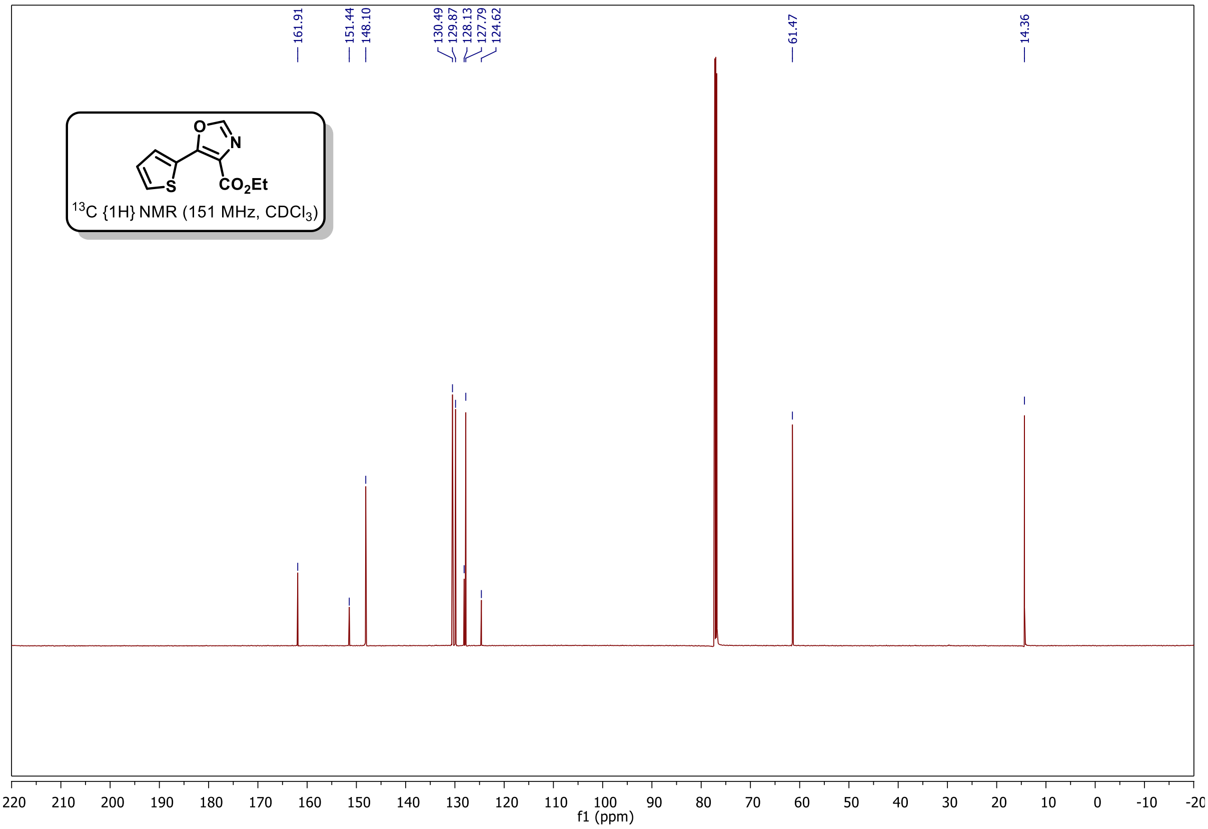

Methyl 5-(1-methyl-1H-indol-2-yl)oxazole-4-carboxylate (3rc):

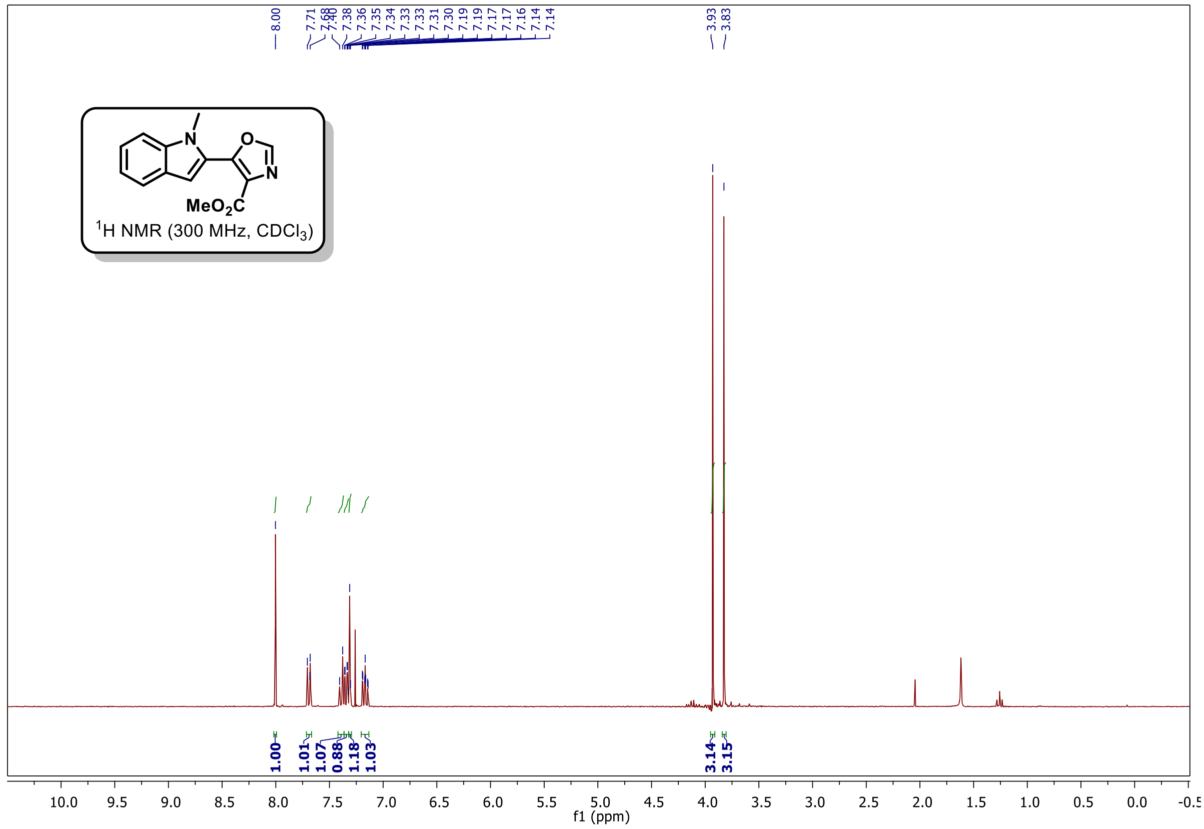

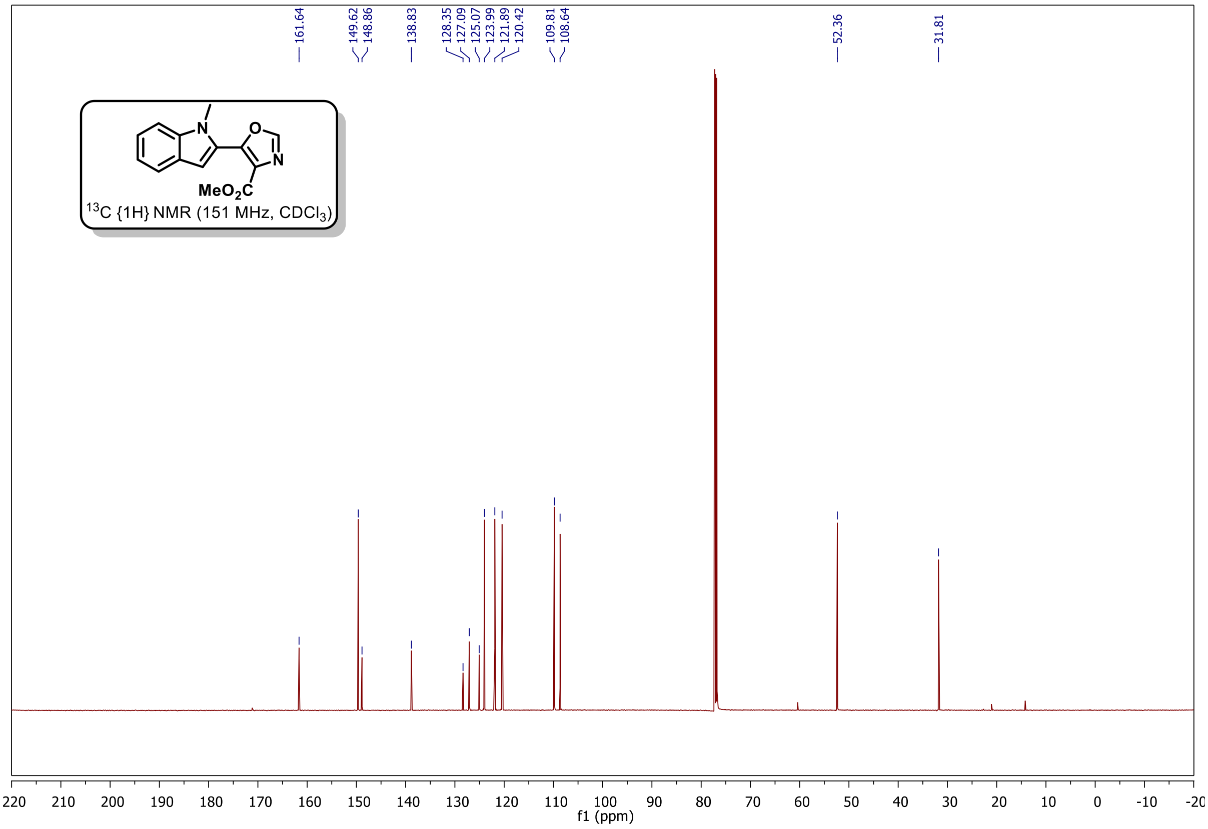

Ethyl 5-(1-methyl-1H-indol-2-yl)oxazole-4-carboxylate (3ra):

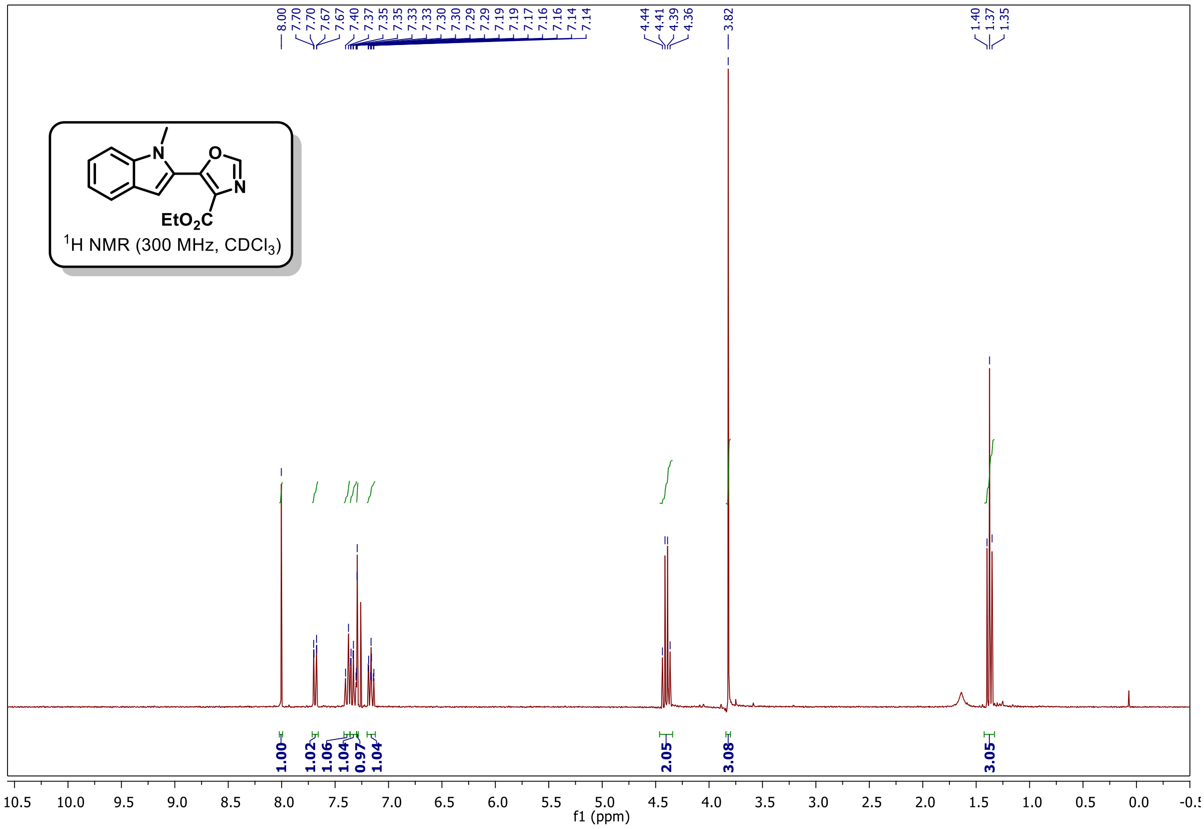

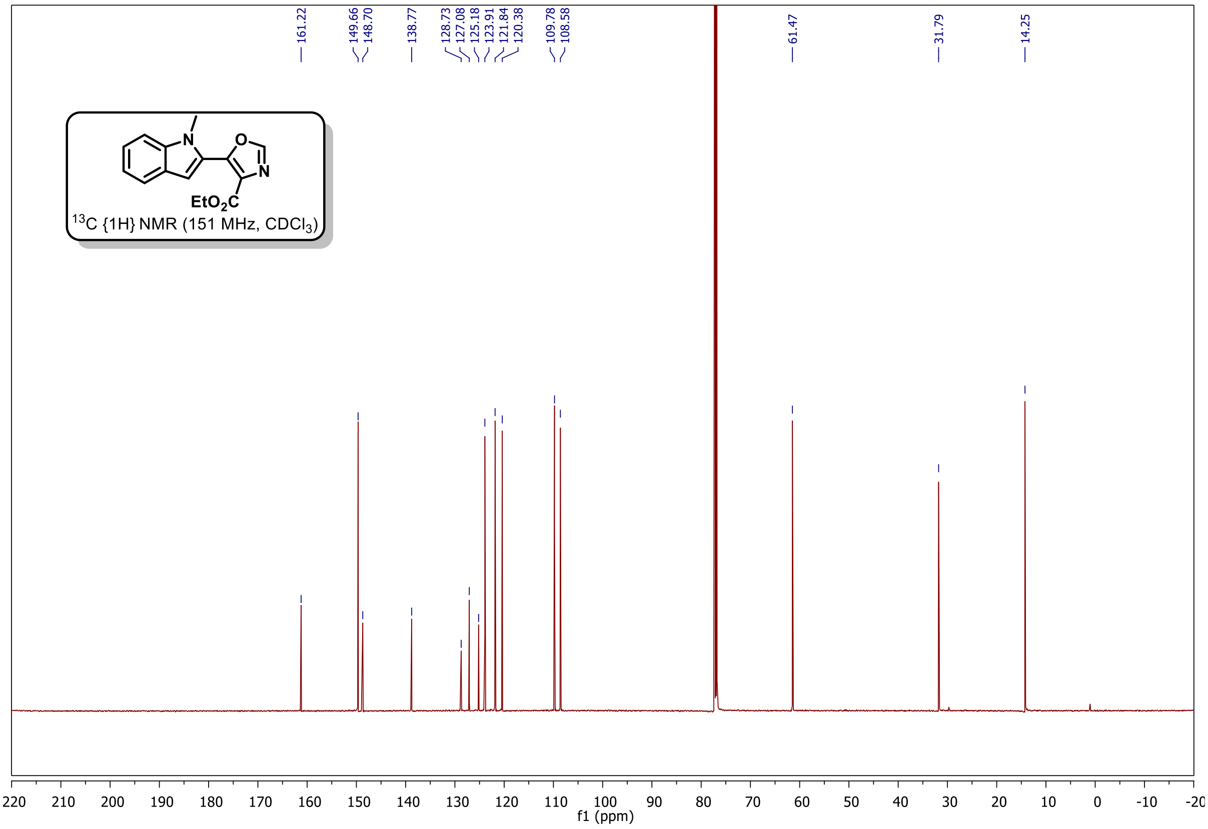

Ethyl 5-(quinoxalin-6-yl)oxazole-4-carboxylate (3sa):

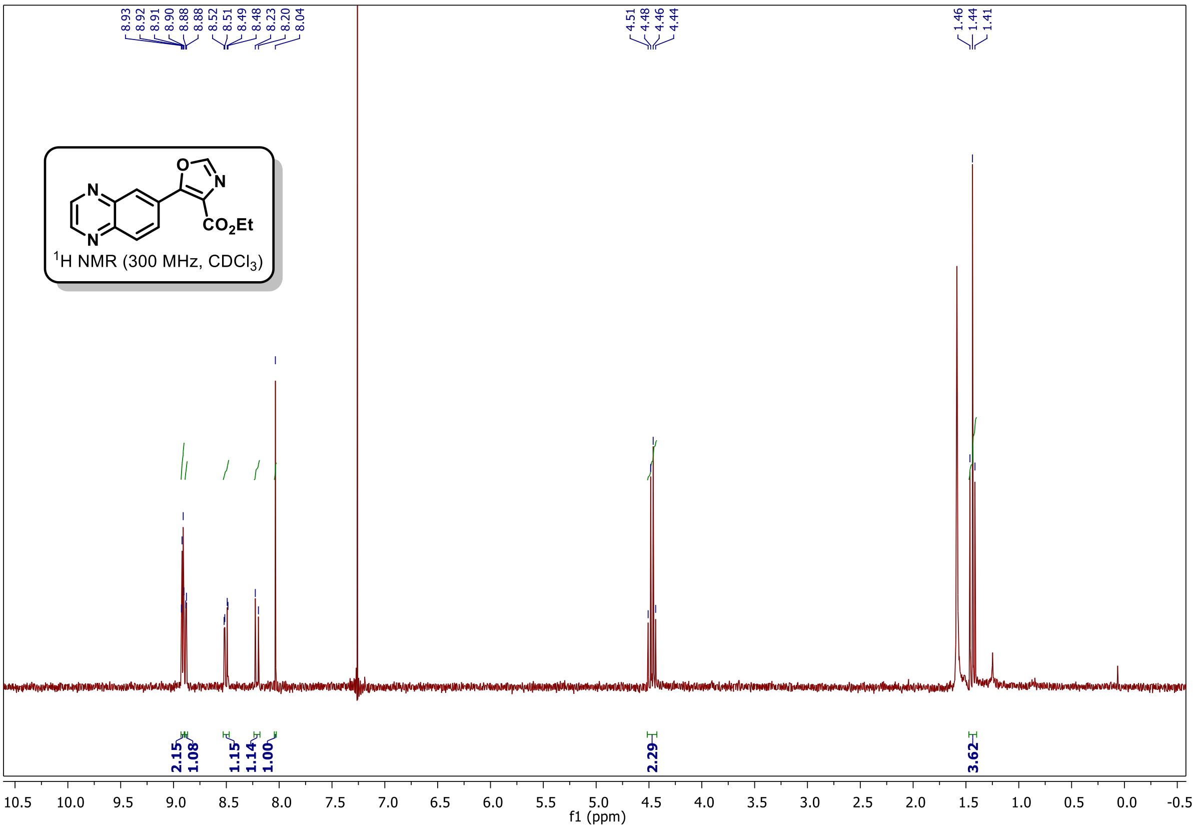

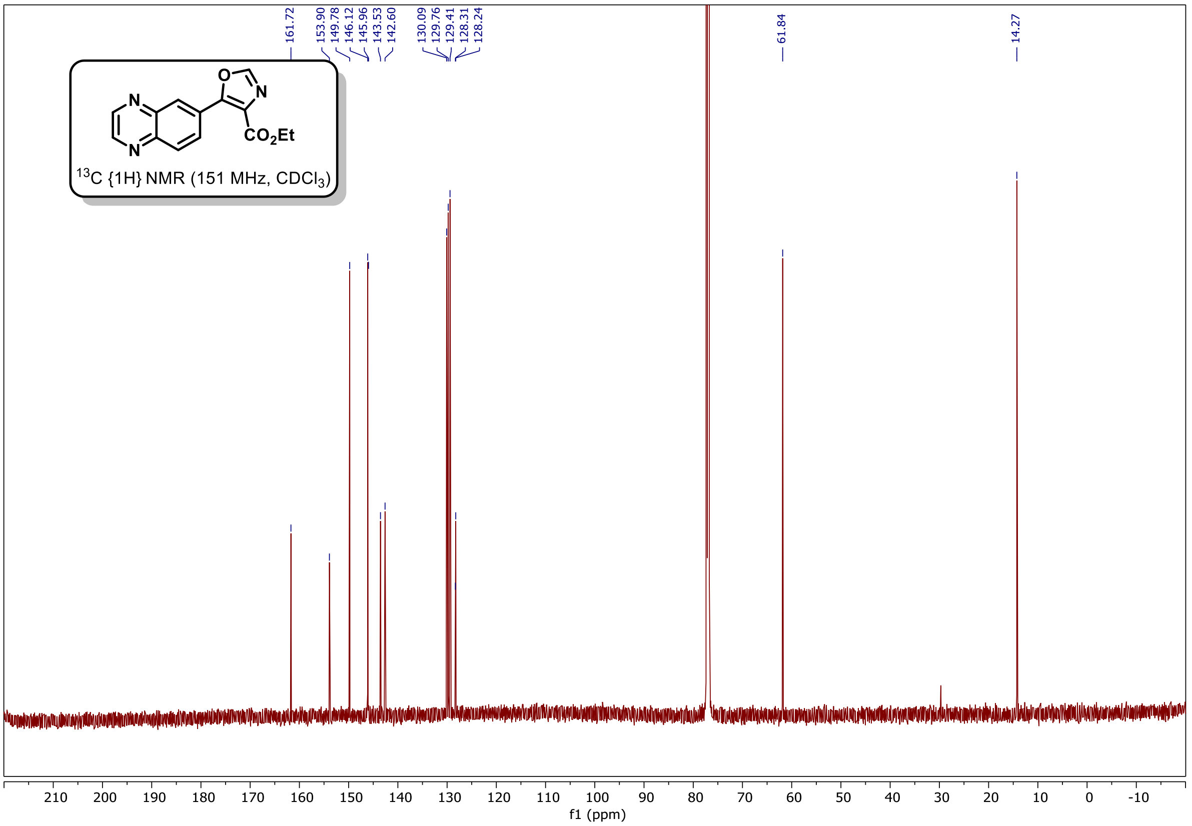

Tert-butyl 5-(quinoxalin-6-yl)oxazole-4-carboxylate (3sb):

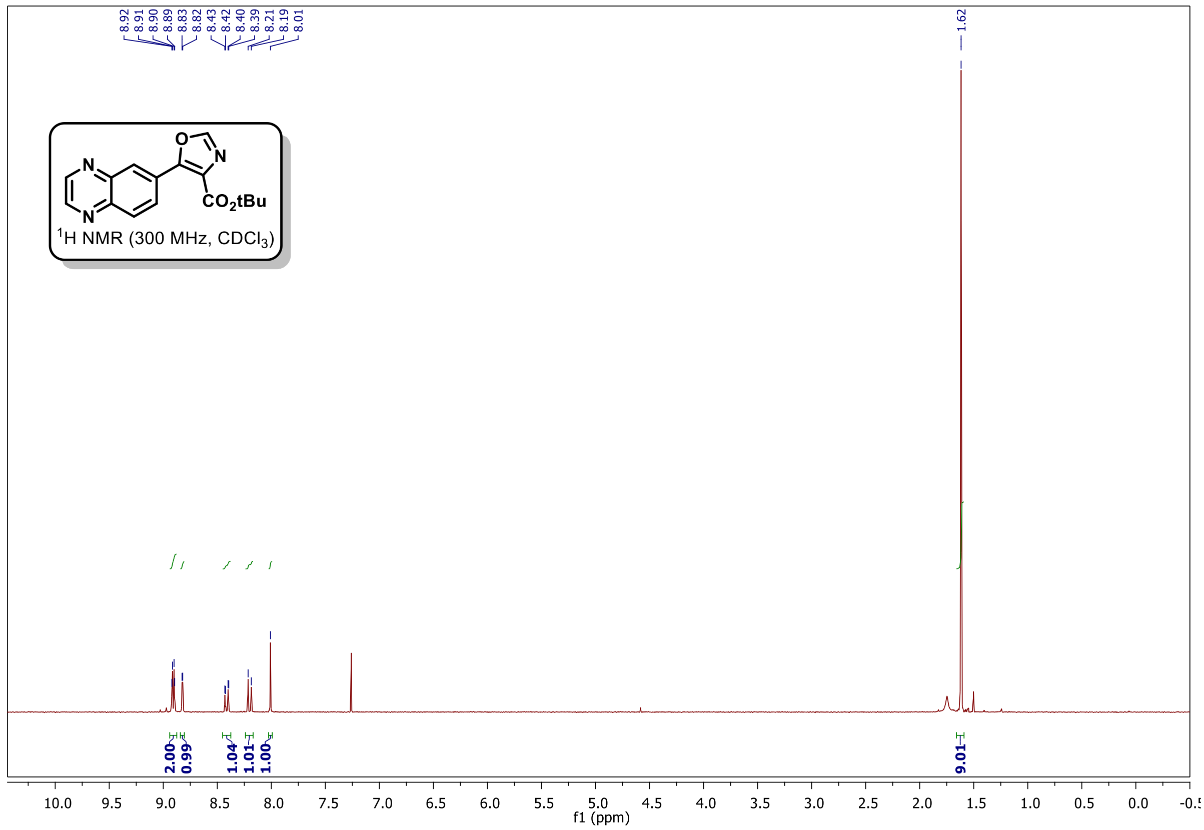

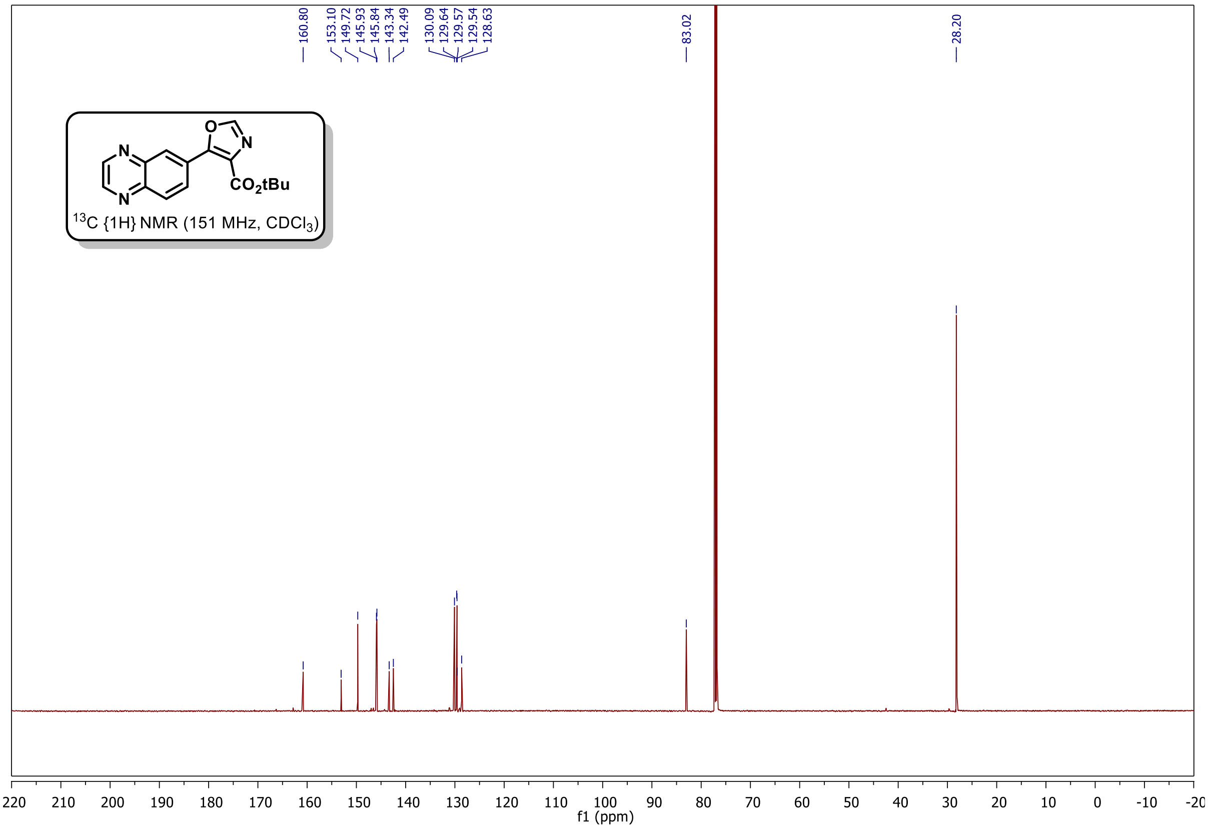

Diethyl 5,5'-(1,4-phenylene)bis(oxazole-4-carboxylate) (3ta):

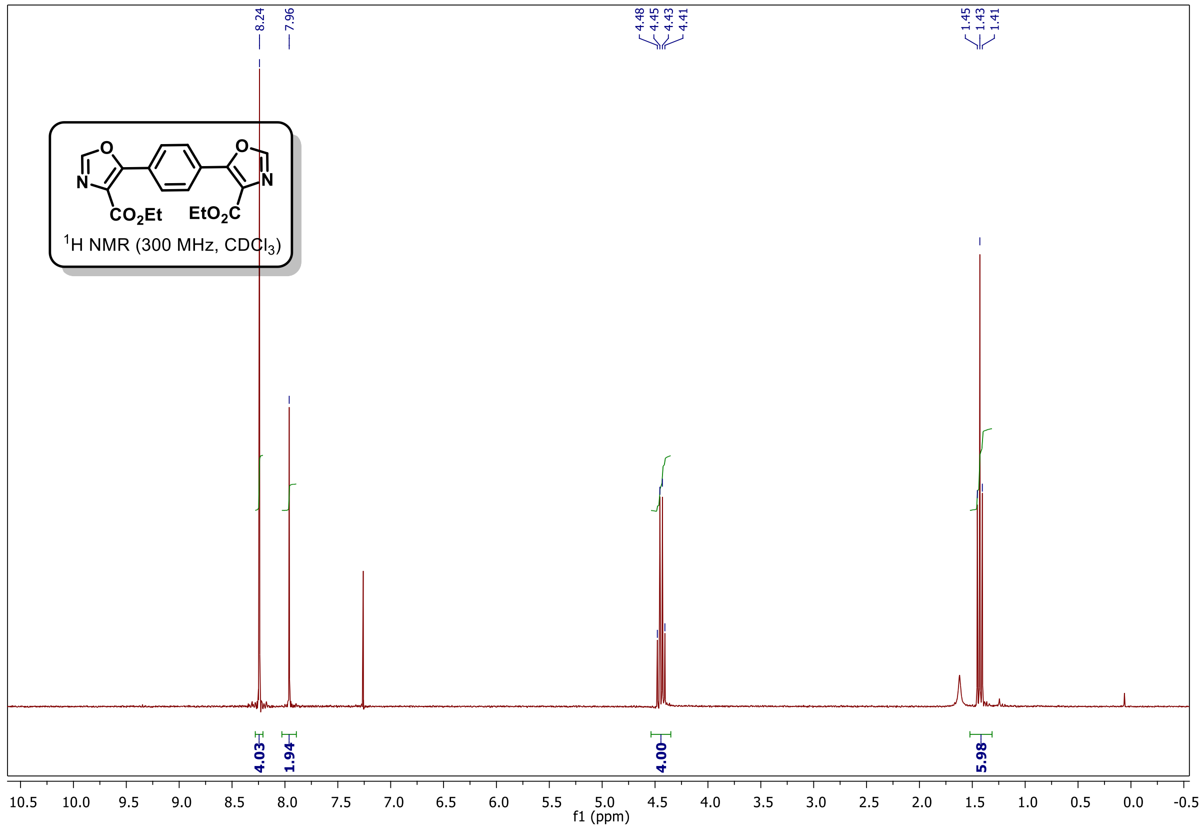

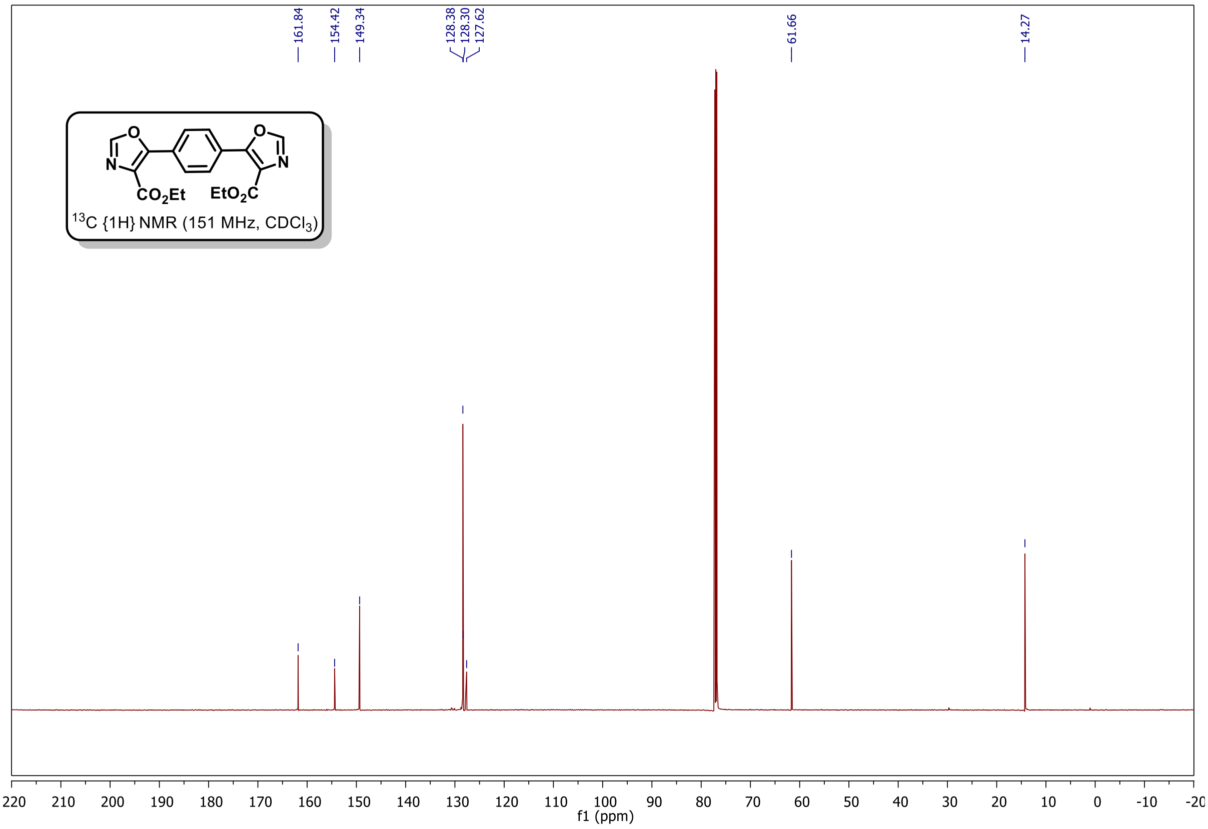

Ethyl 5-(4-(diphenylphosphaneyl)phenyl)oxazole-4-carboxylate (3ua):

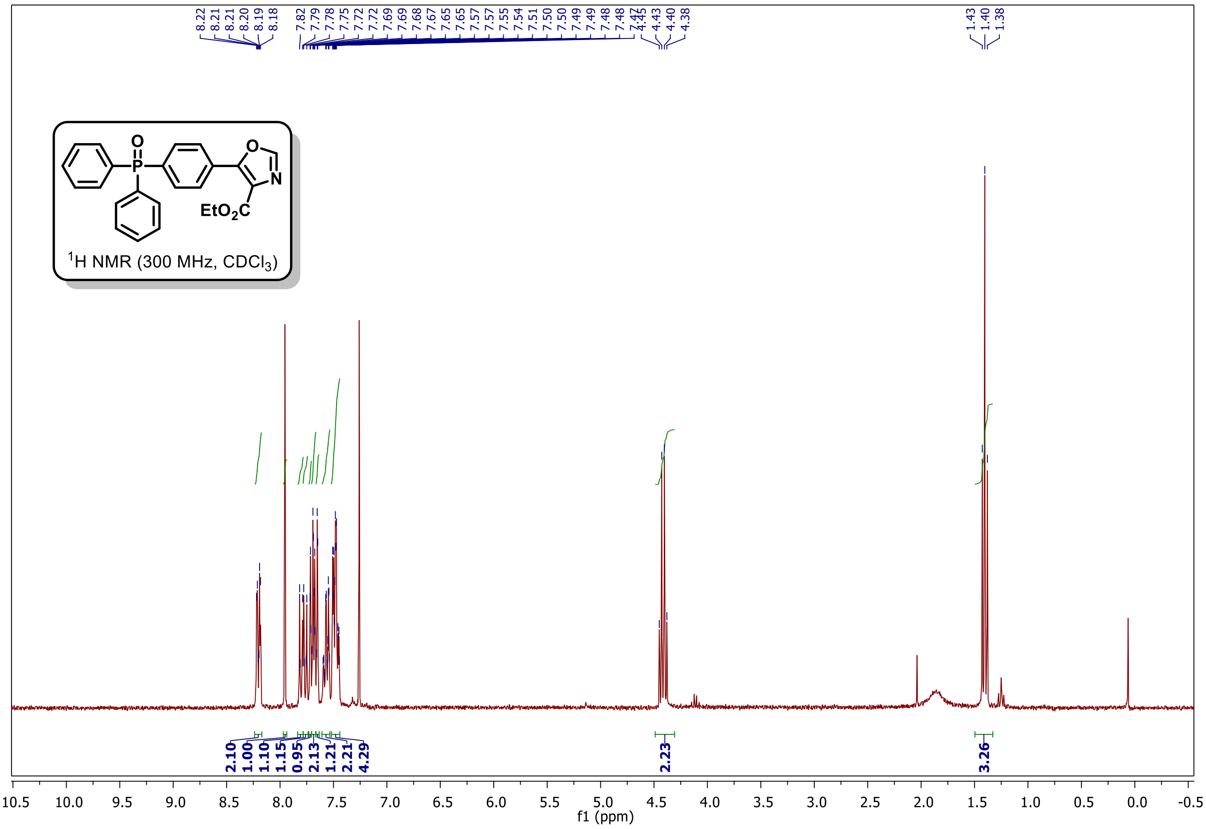

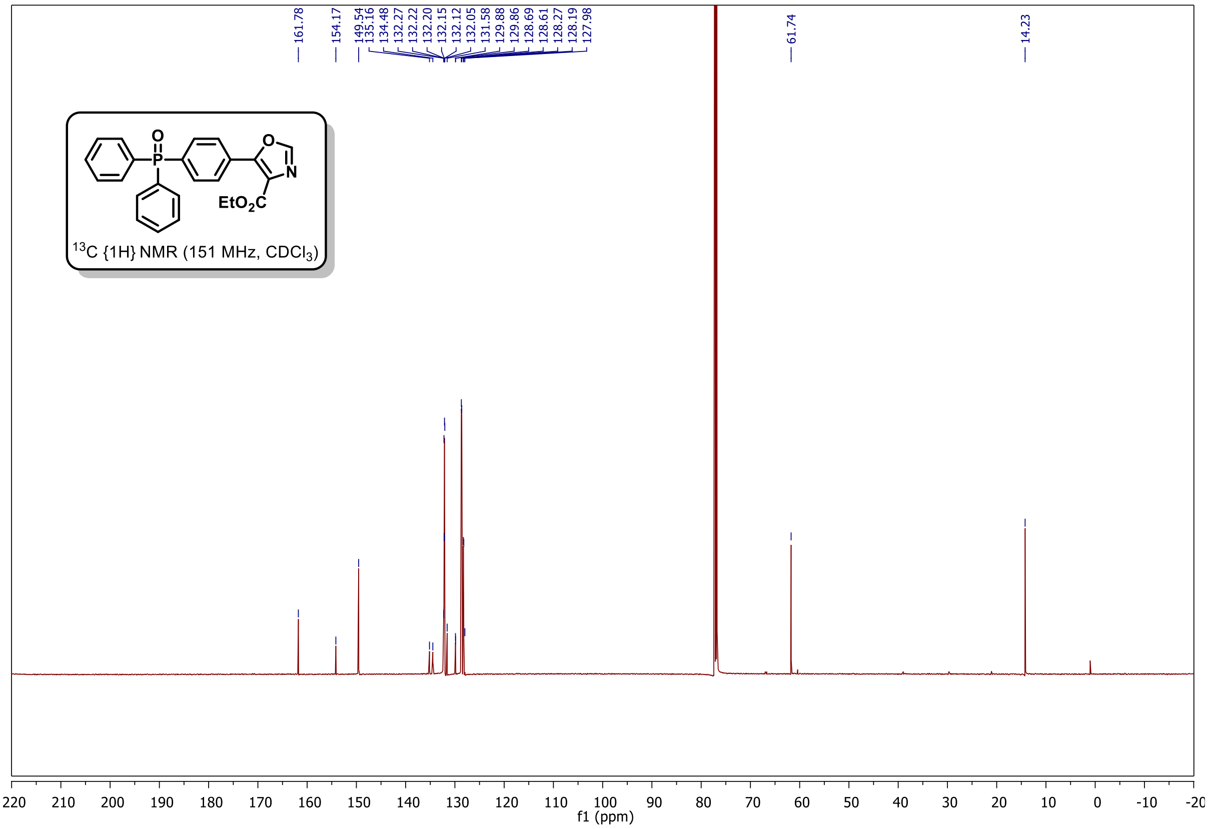

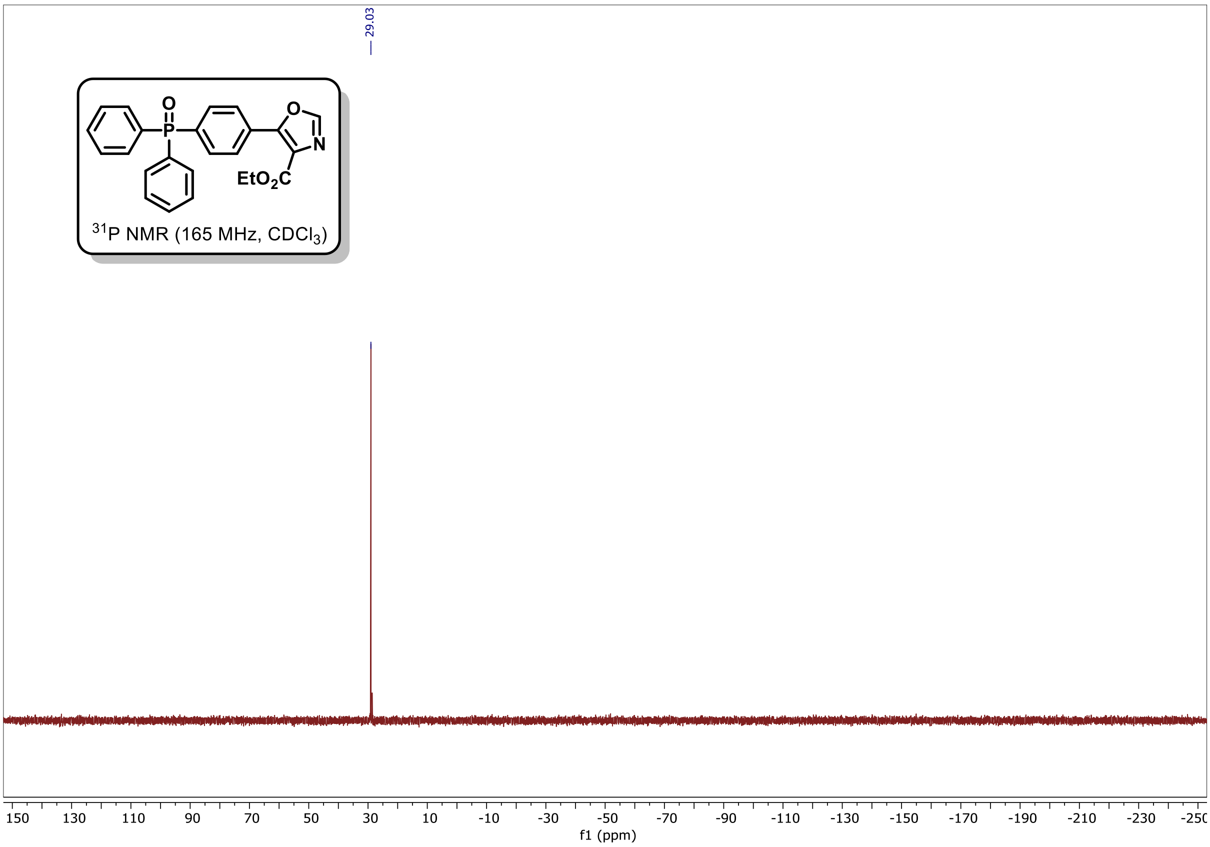

29.03
EtO2C
O
N
P
O
31P NMR (165 MHz, CDCl3)
150
130
110
90
70
50
30
10
-10
-30
-50
-70
-90
-110
-130
-150
-170
-190
-210
-230
-250
f1 (ppm)

Tert-butyl 5-(4-(diphenylphosphaneyl)phenyl)oxazole-4-carboxylate (3ub):

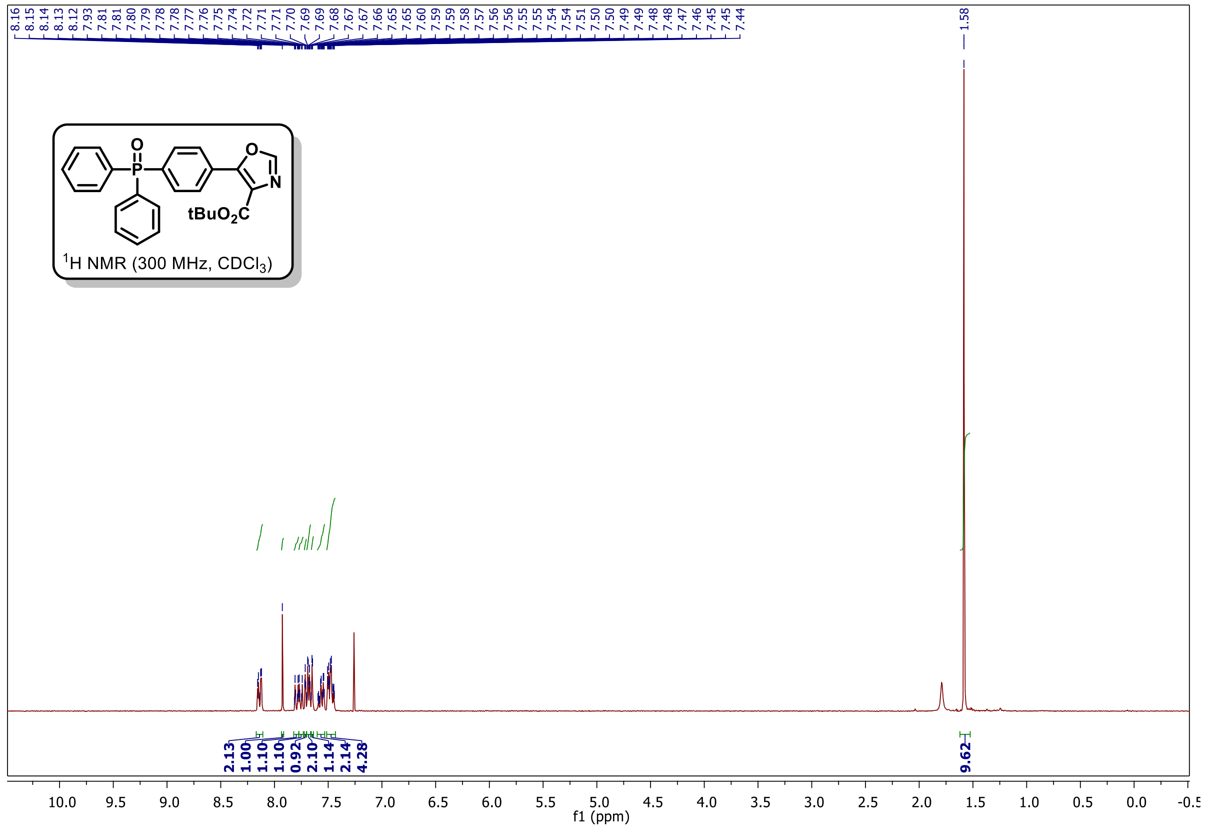

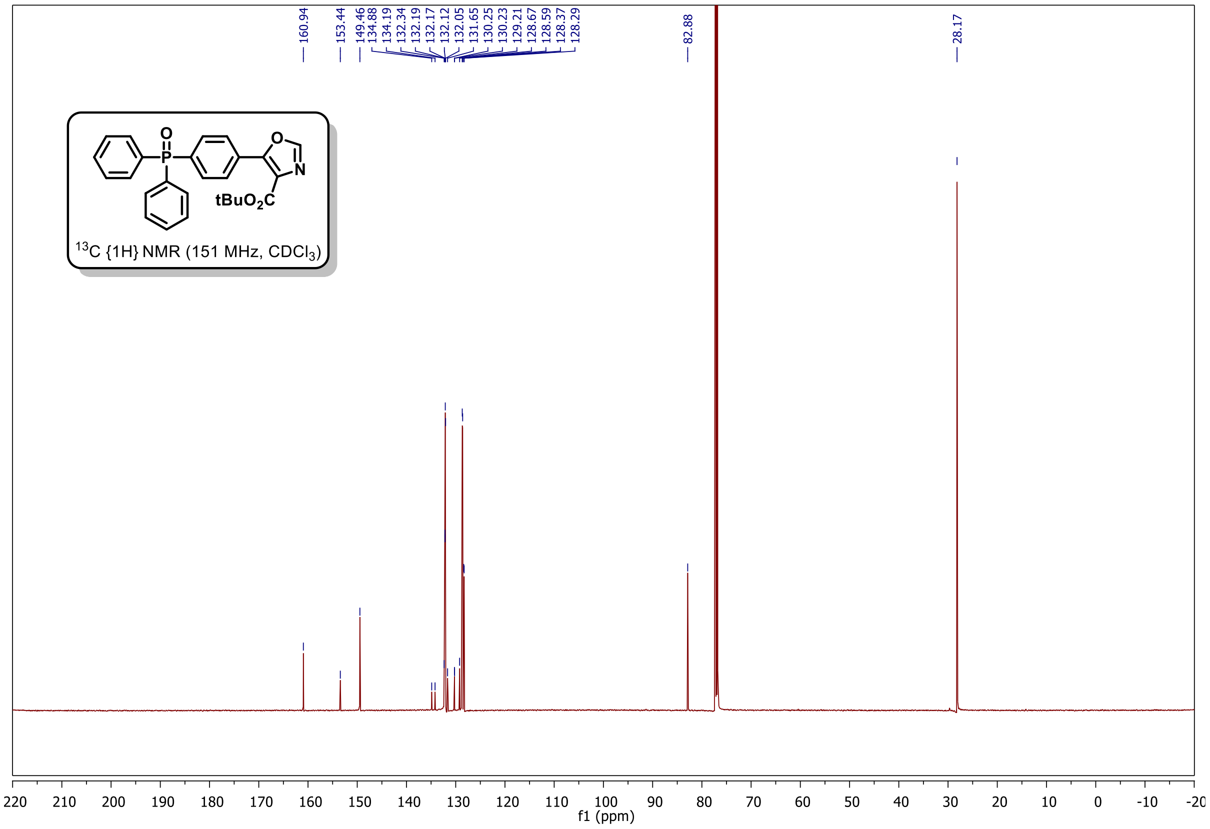

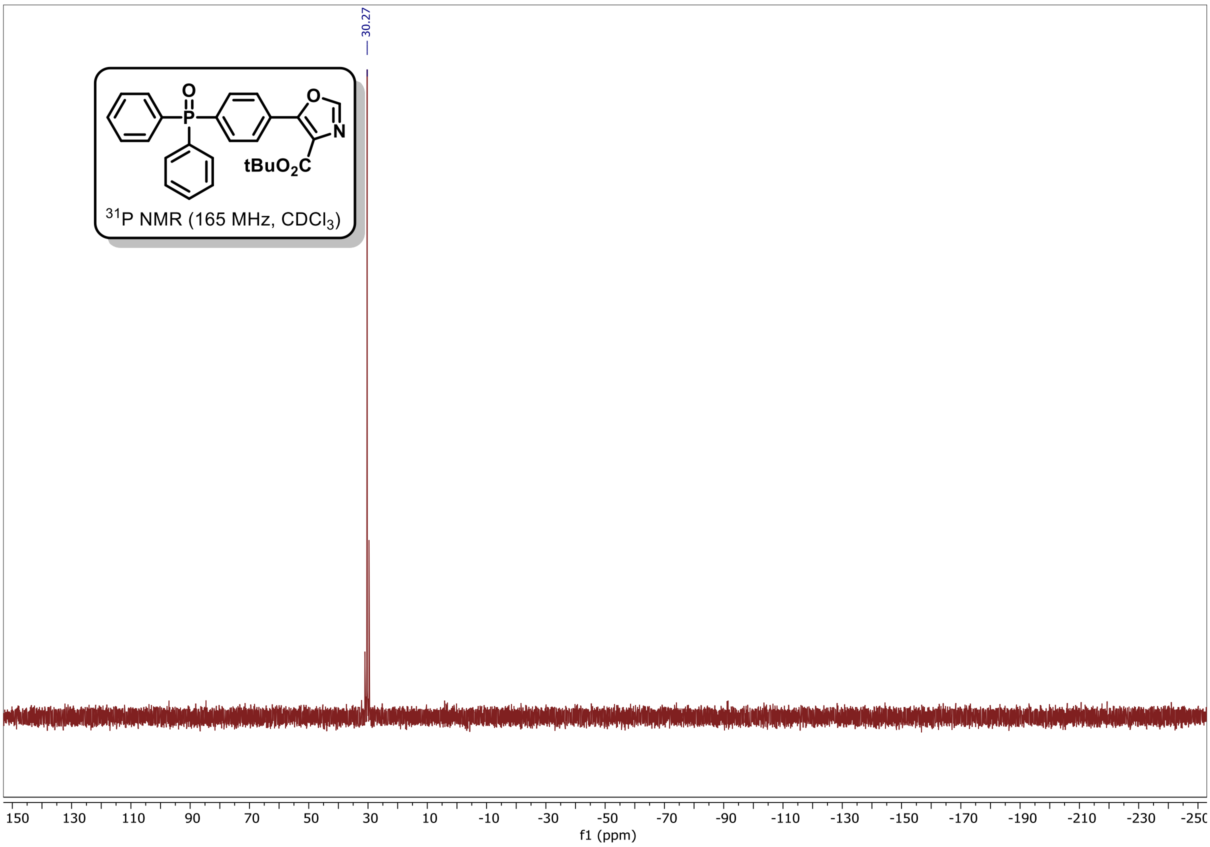
30.27
O
P
O
N
tBuO2C
31P NMR (165 MHz, CDCl3)
150 130 110 90 70 50 30 10 -10 -30 -50 -70 -90 -110 -130 -150 -170 -190 -210 -230 -250
f1 (ppm)

Methyl 5-(3,4,5-trimethoxyphenethyl)oxazole-4-carboxylate (3vc):

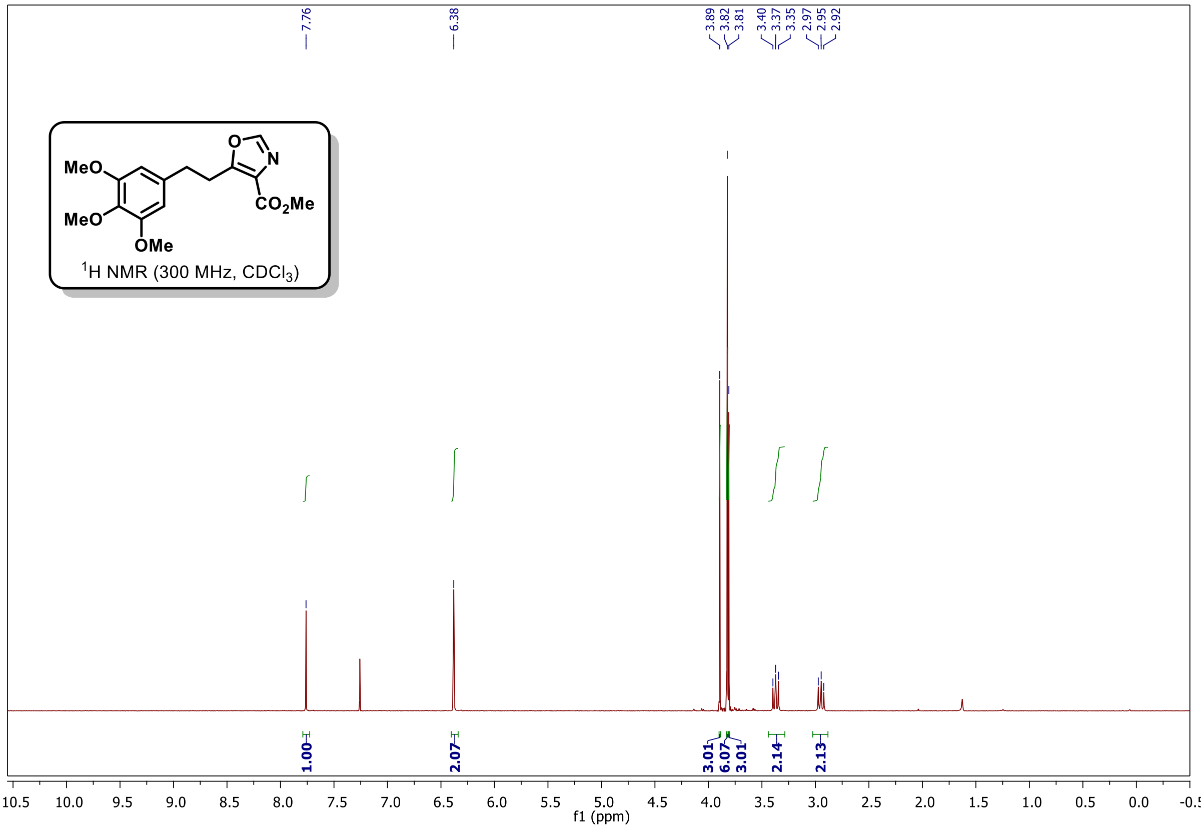

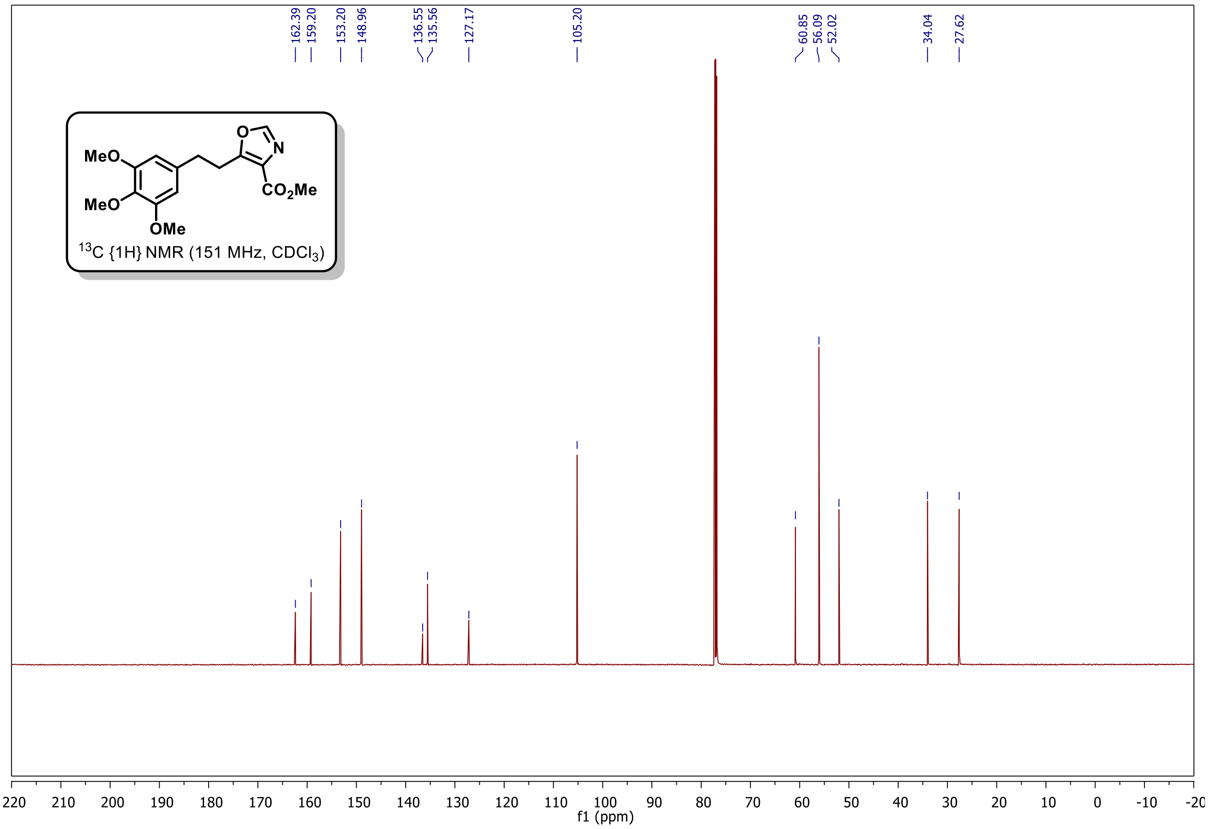

4-tosyl-5-(3, 4, 5-trimethoxyphenethyl)oxazole (3vd):

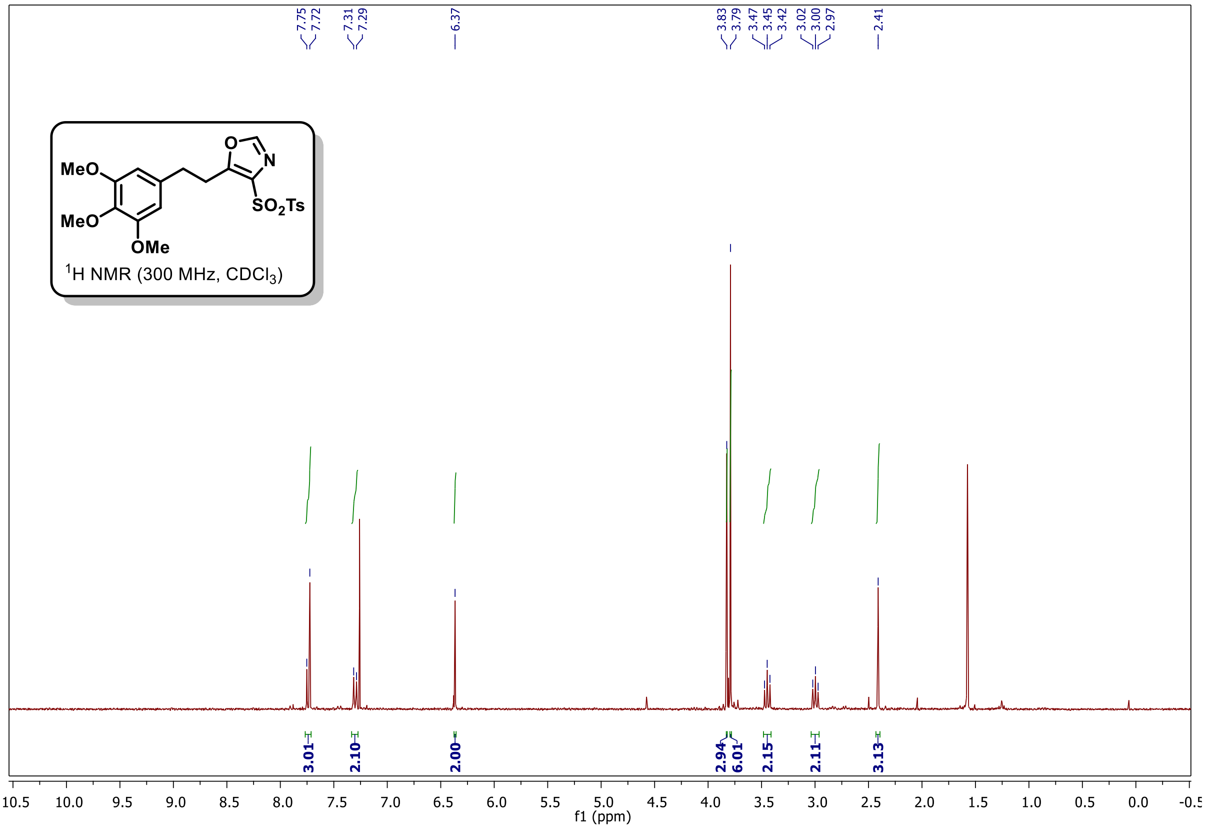

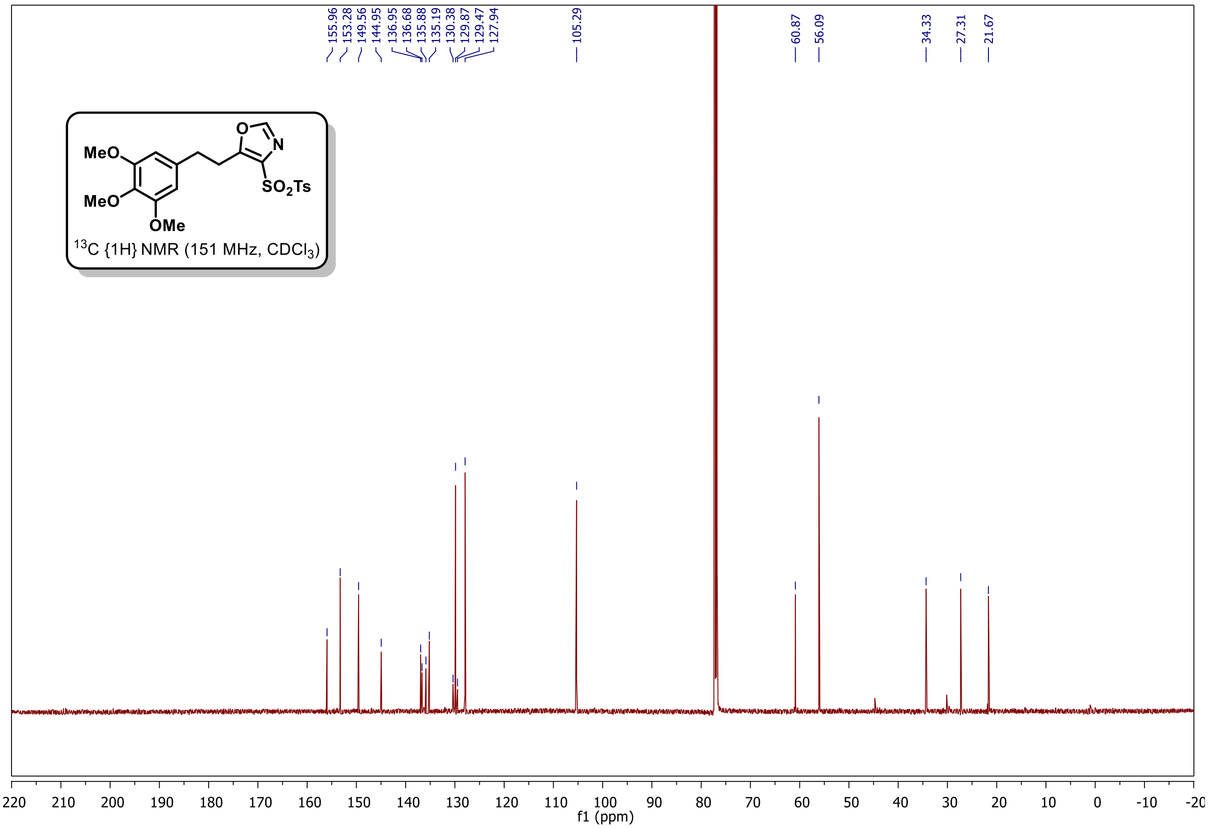

Methyl 5-(3-methoxy-3-oxopropyl)oxazole-4-carboxylate (3wc):

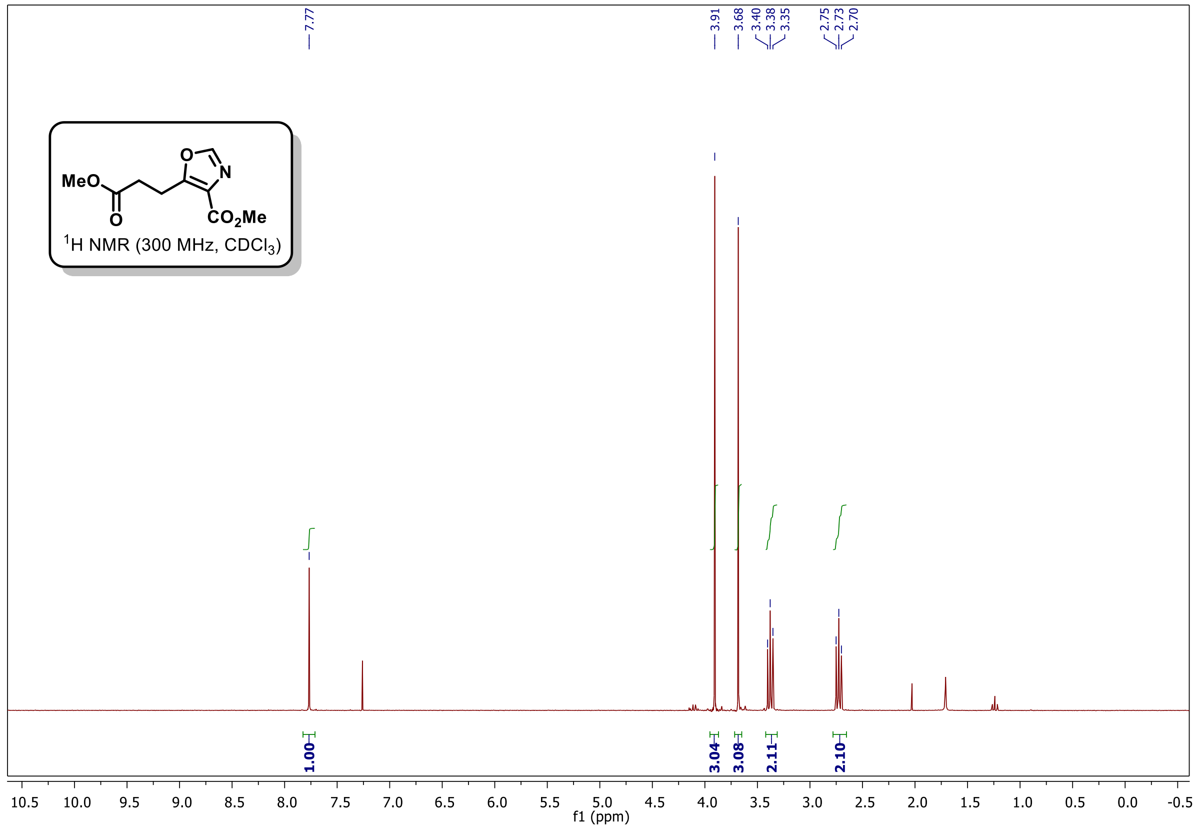

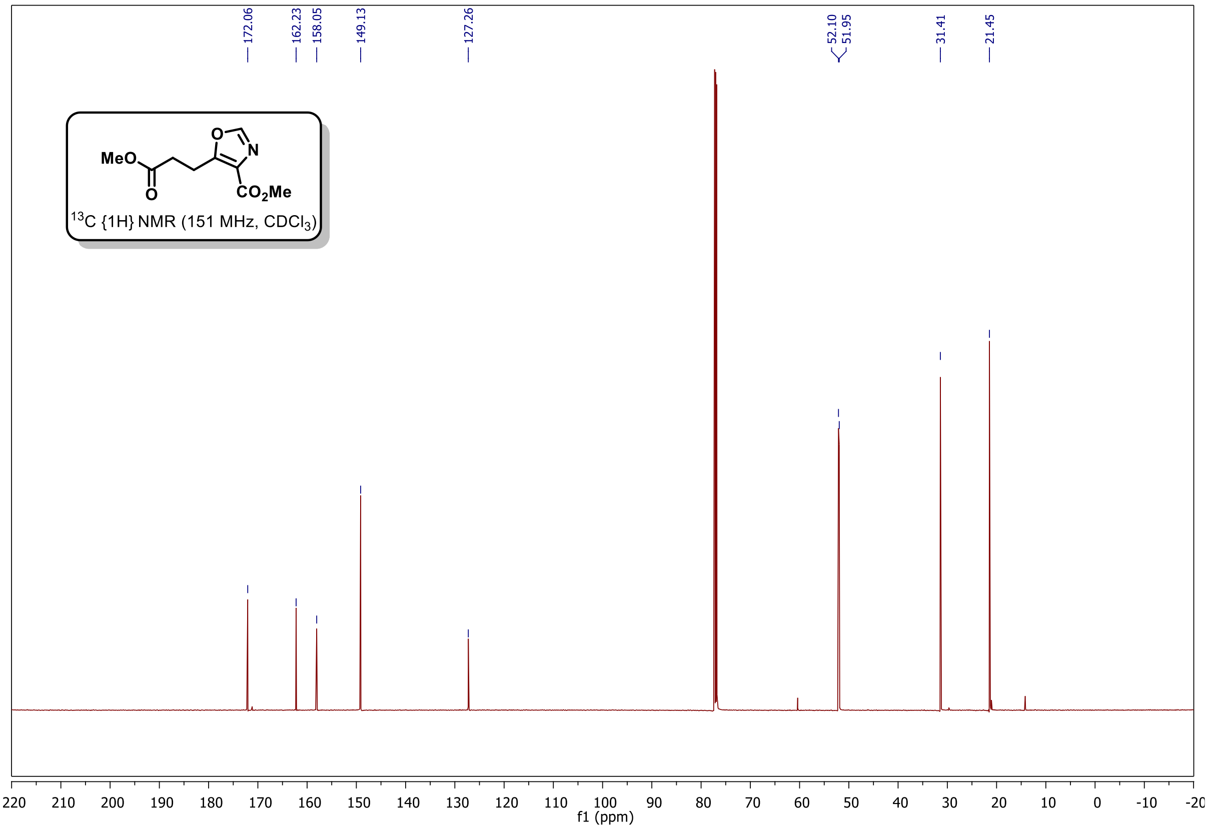

5-amino-4-oxopentanoic acid hydrochloride (4wc):[6]

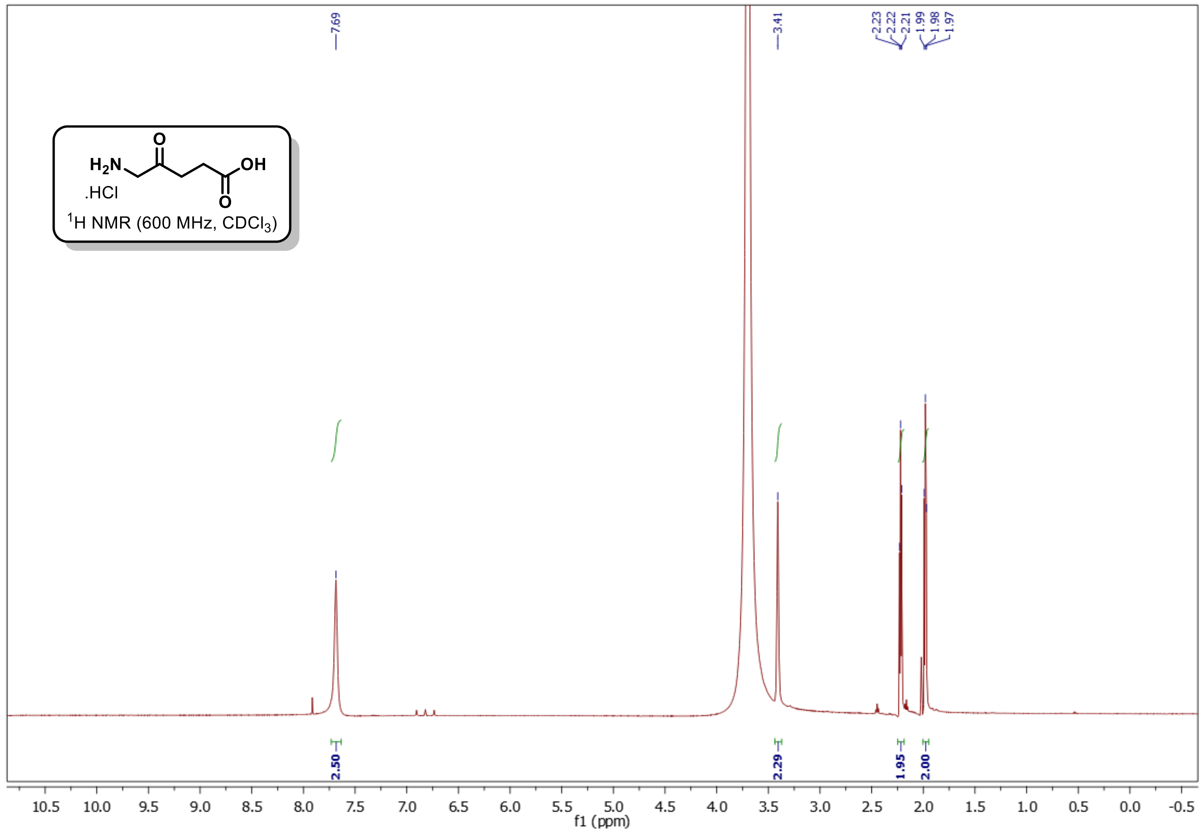

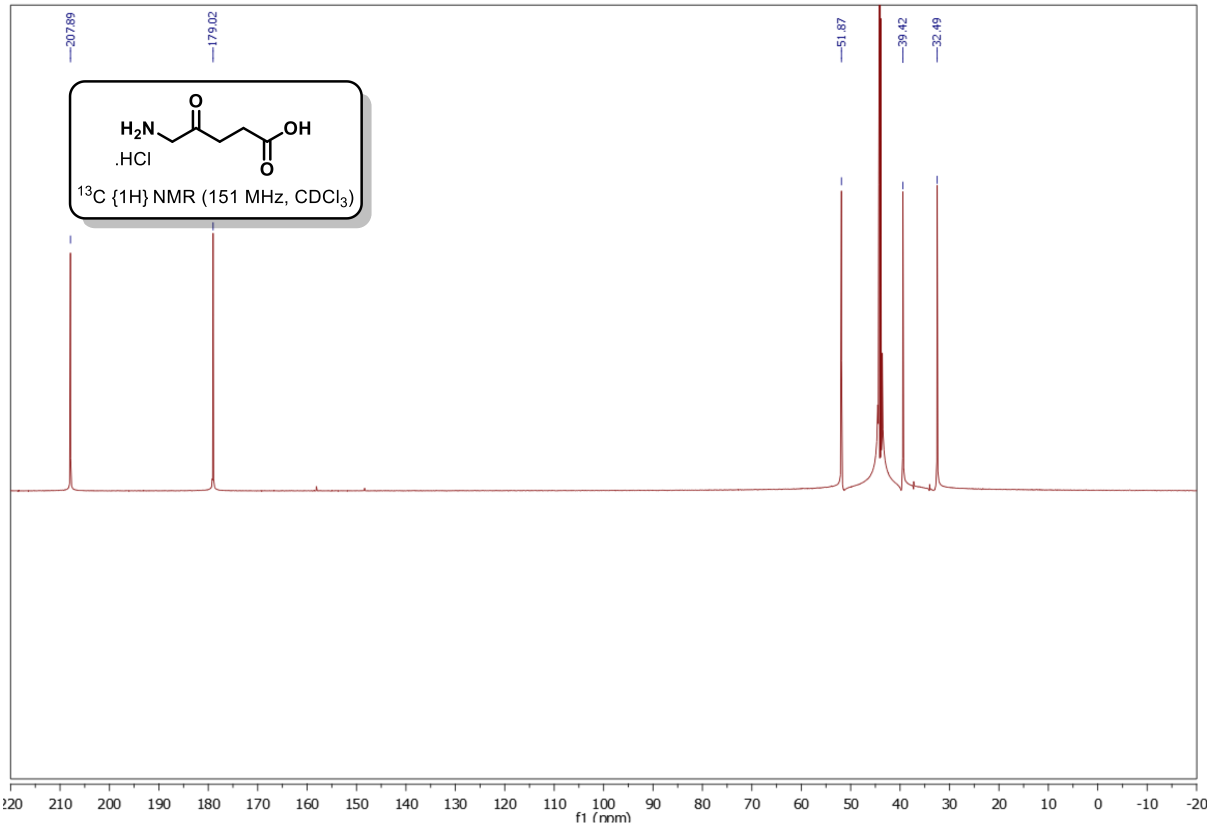

Ethyl 5-(2-(4-(methoxycarbonyl)oxazol-5-yl)ethyl)oxazole-4-carboxylate (3xa):

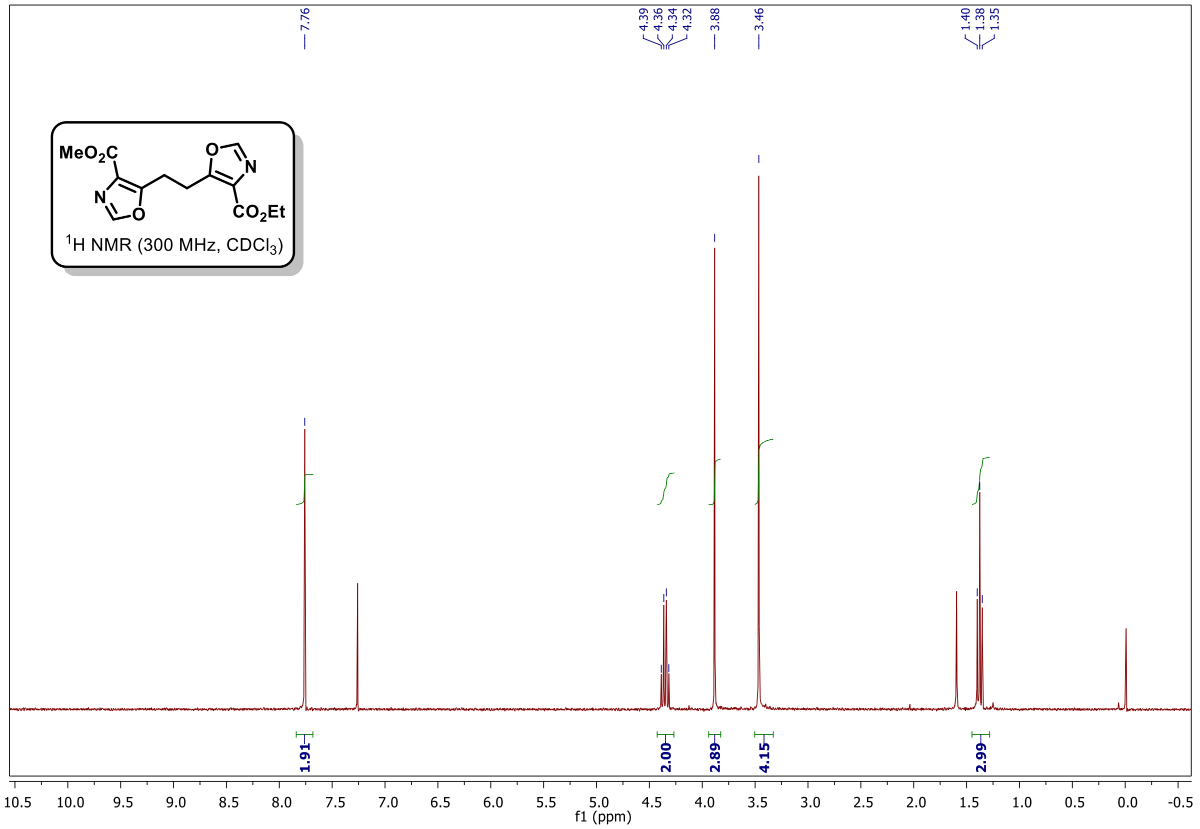

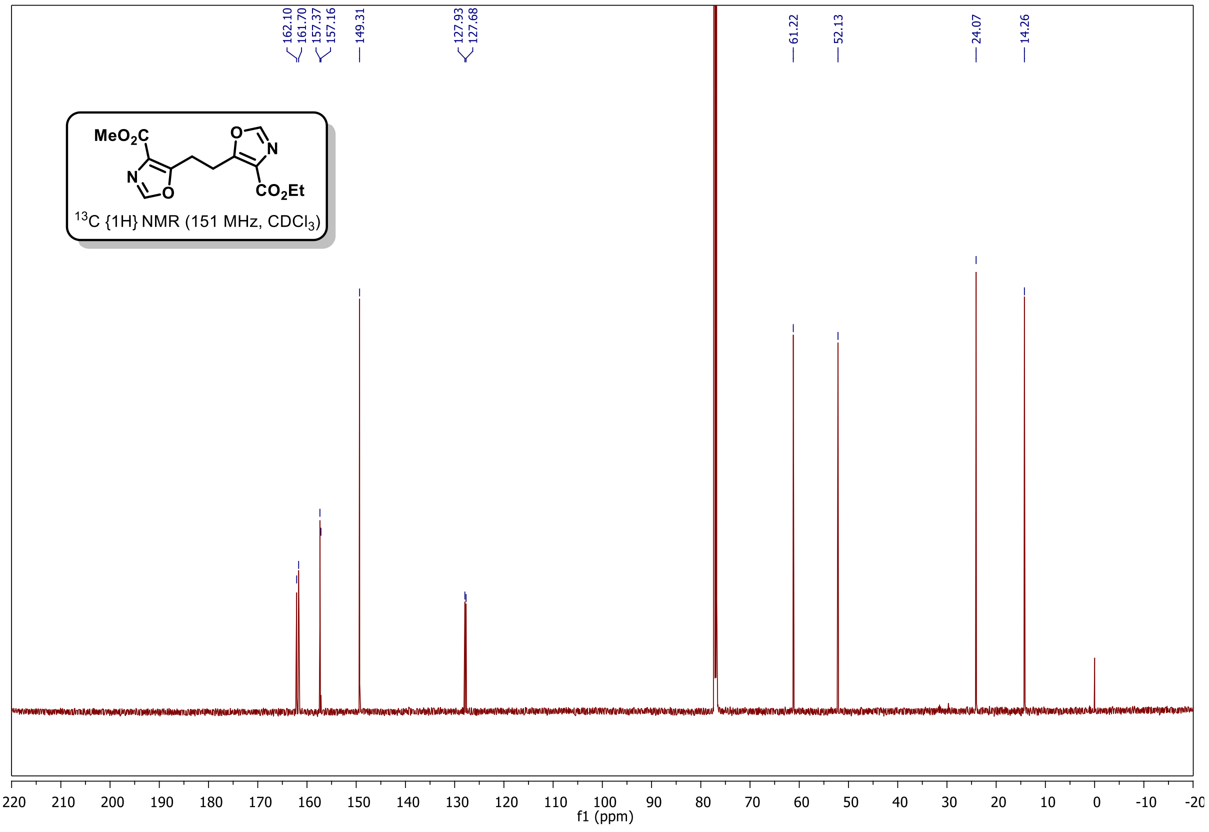

Ethyl 5-cyclohexyloxazole-4-carboxylate (3ya):

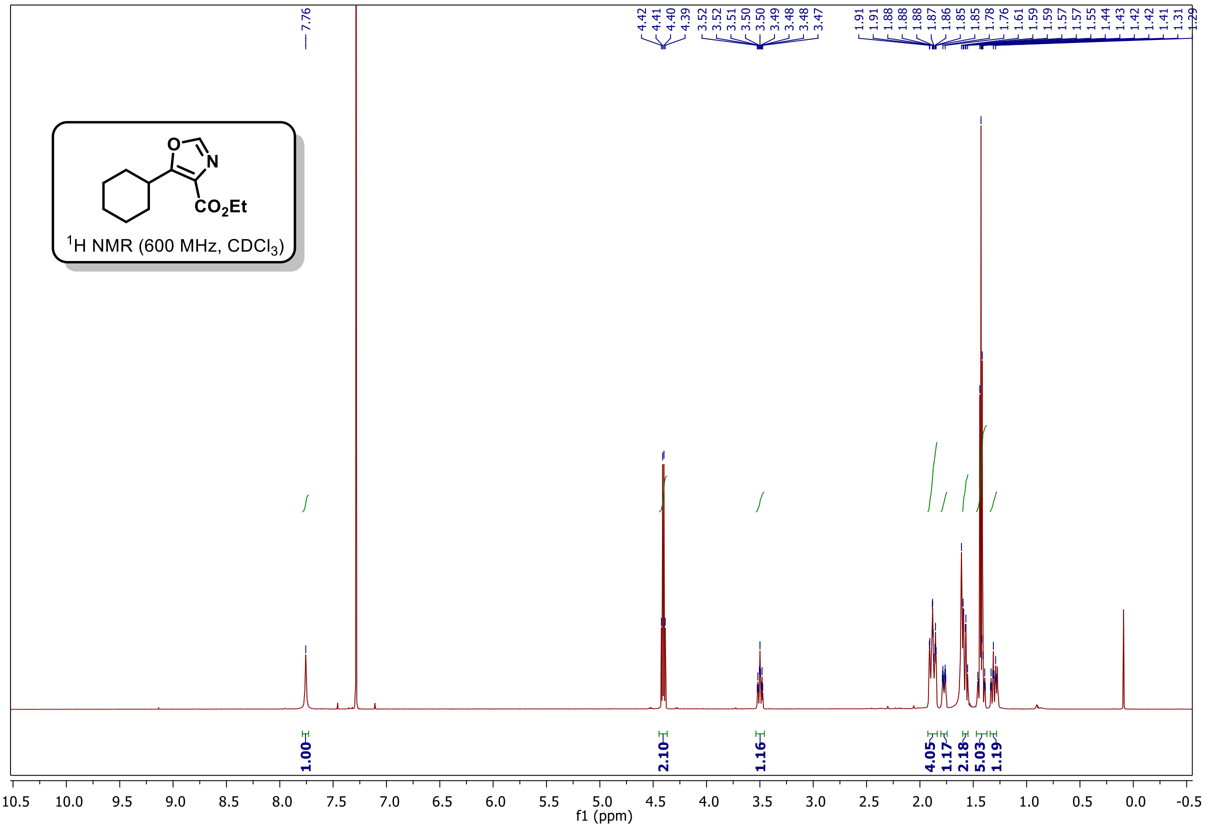

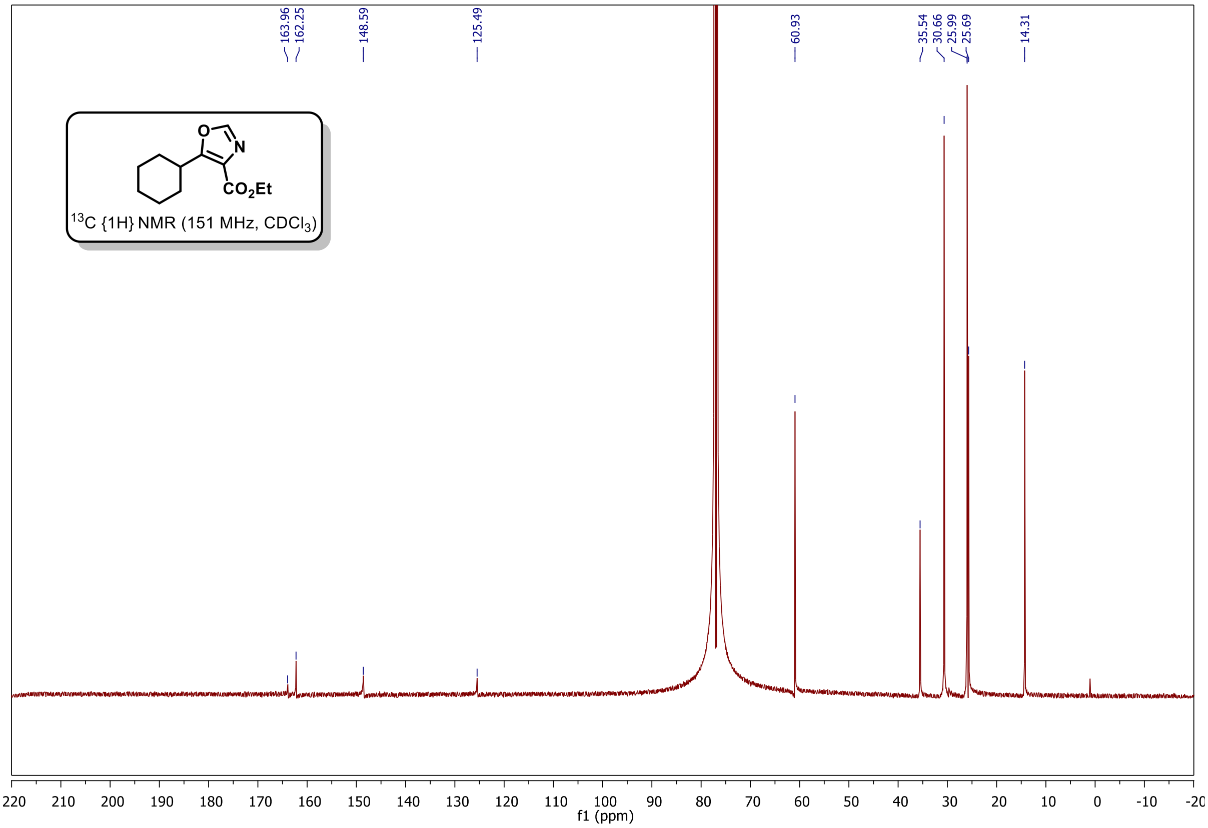

Ethyl 5-(4,4-difluorocyclohexyl)oxazole-4-carboxylate (3za):

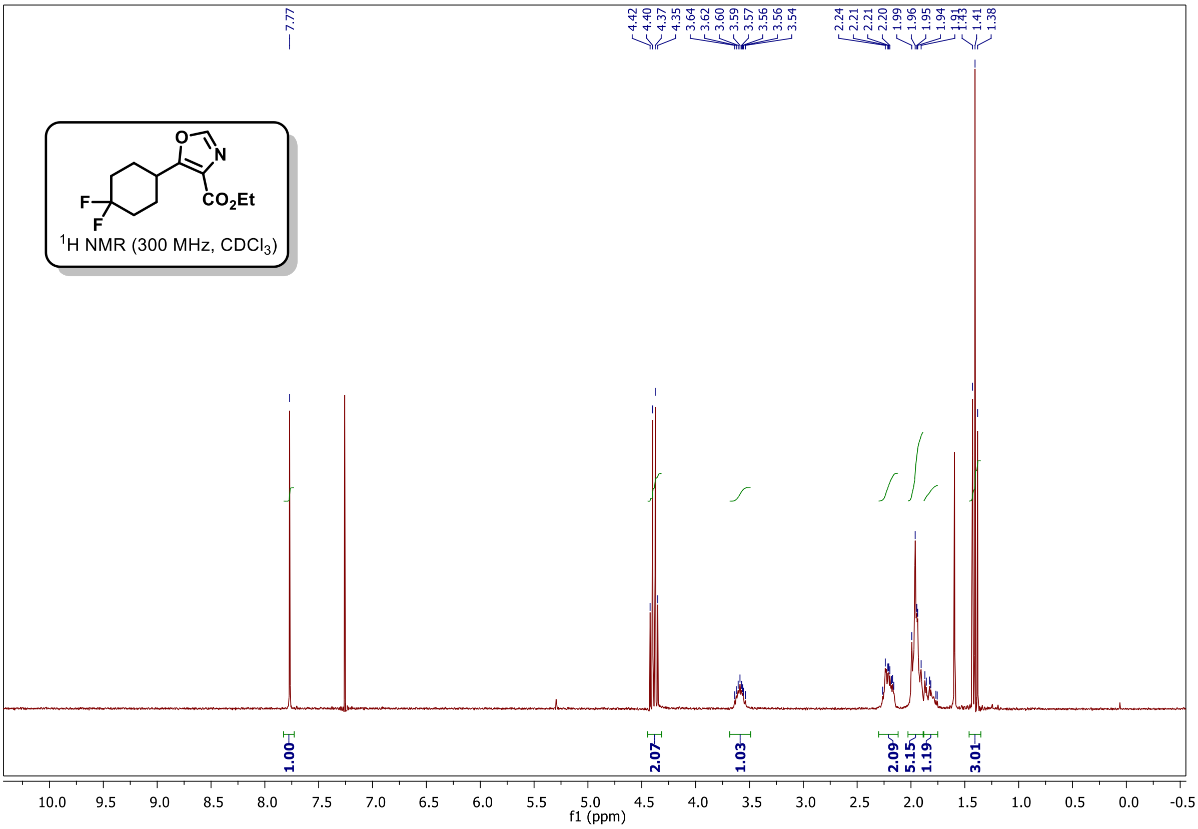

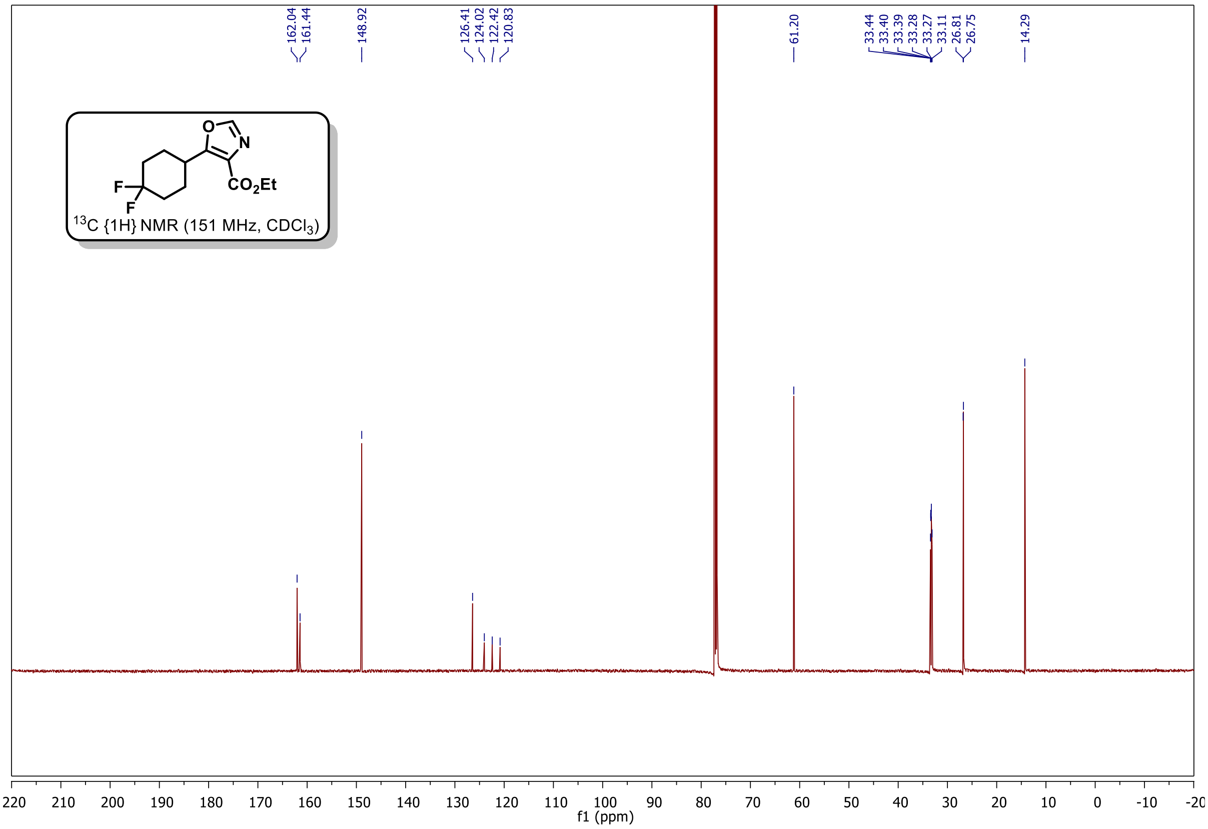

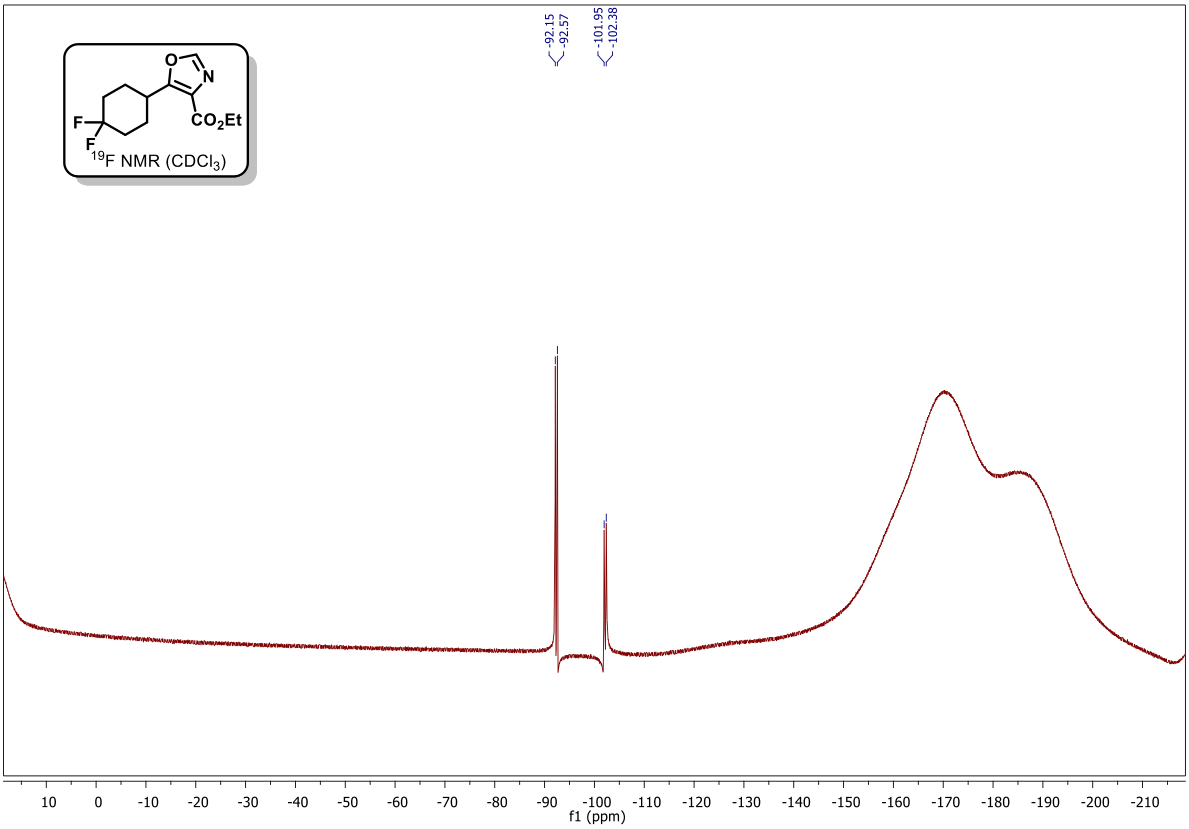
-92.15
-92.57
-101.95
-102.38
O
N
F
F
CO2Et
19F NMR (CDCl3)
10
0
-10
-20
-30
-40
-50
-60
-70
-80
-90
-100
-110
-120
-130
-140
-150
-160
-170
-180
-190
-200
-210
f1 (ppm)

Methyl 5-(1-(tert-butoxycarbonyl)piperidin-4-yl)oxazole-4-carboxylate (3a'c):

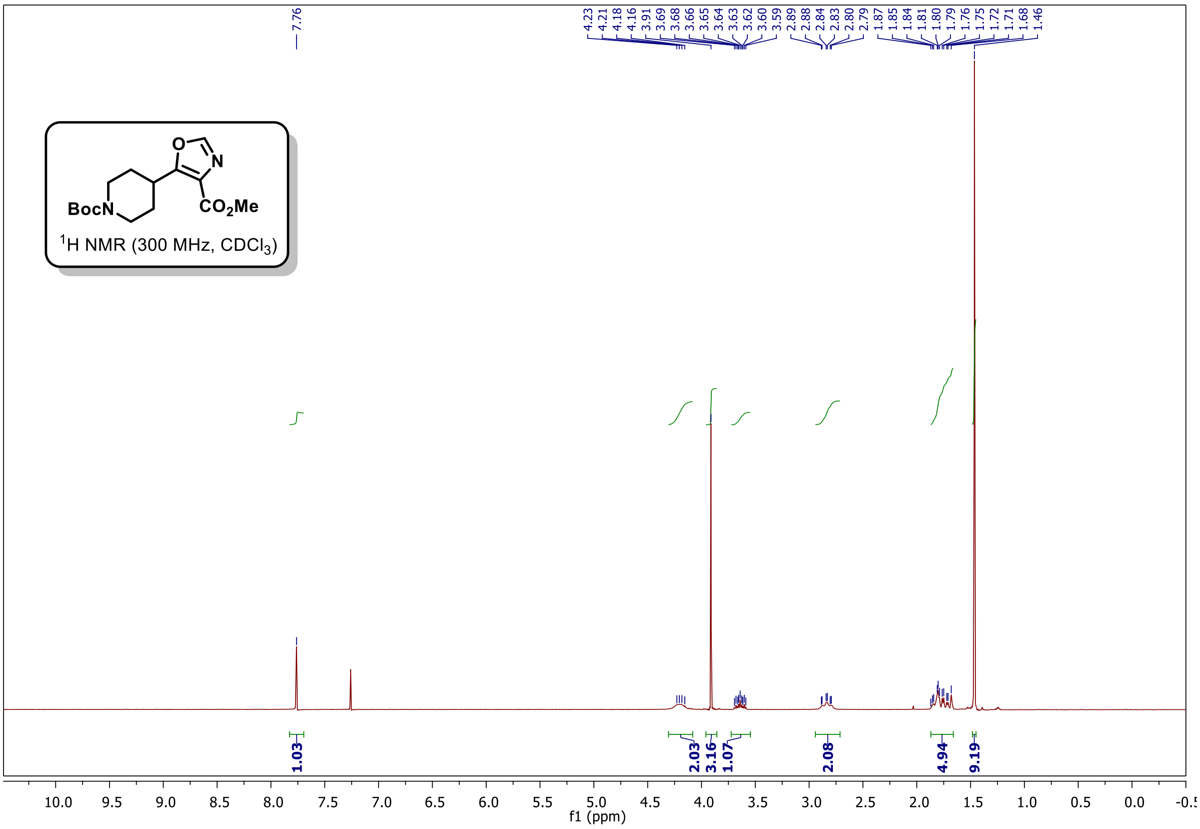

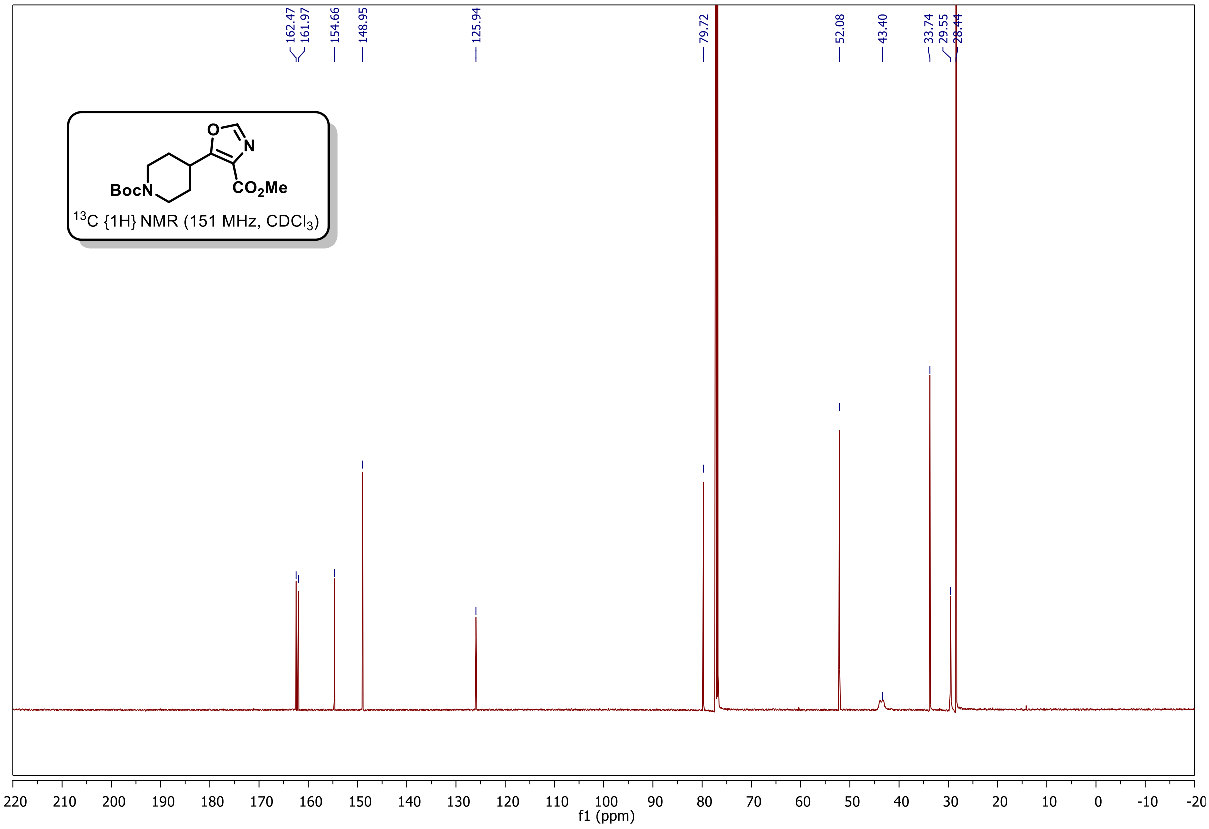

Tert-butyl 4-(4-(tosylsulfonyl)oxazol-5-yl)piperidine-1-carboxylate (3a'd):

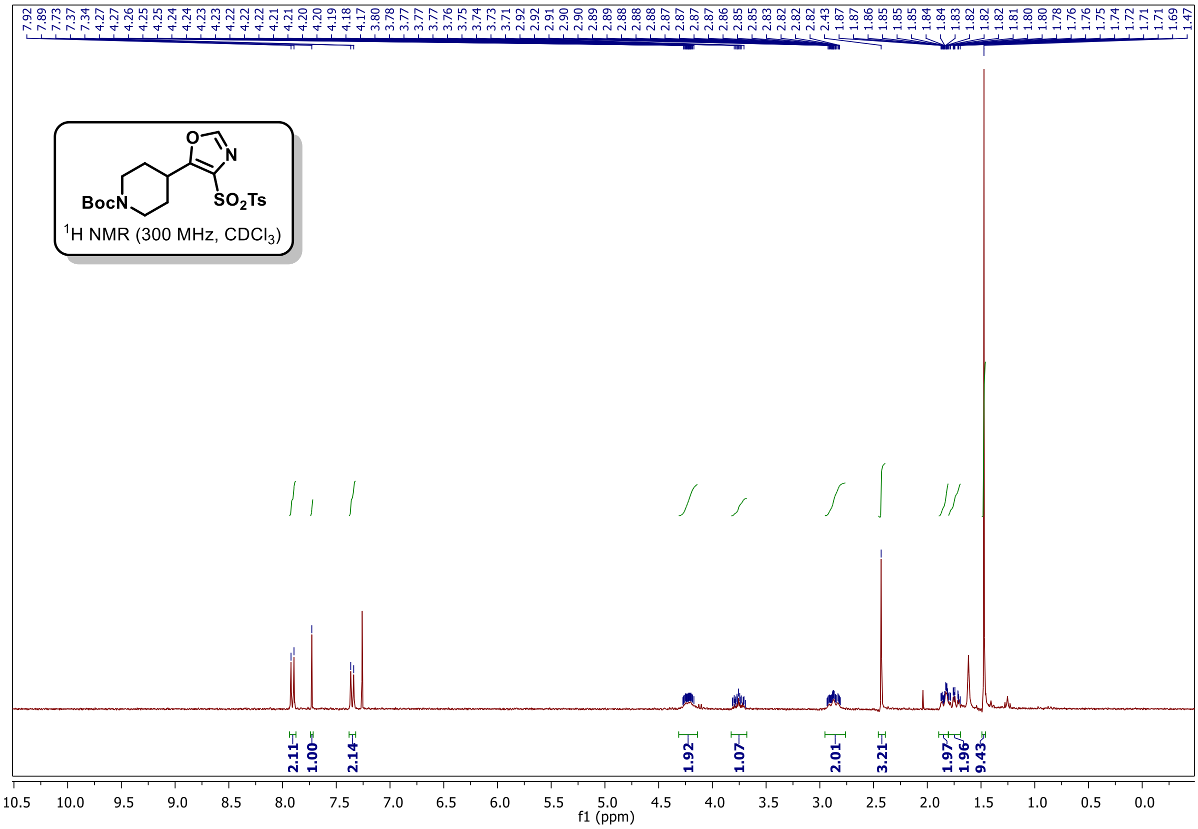

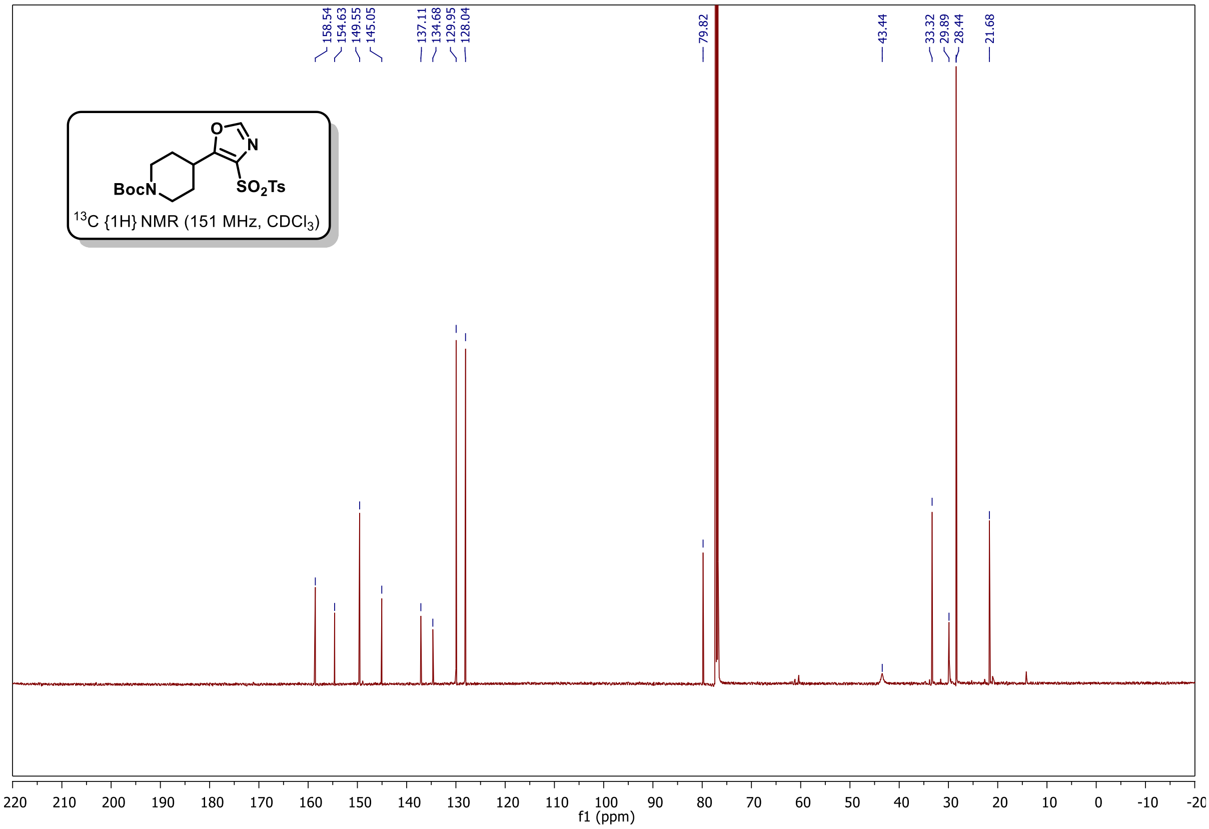

Ethyl 5-cyclopropyloxazole-4-carboxylate (3b'a):

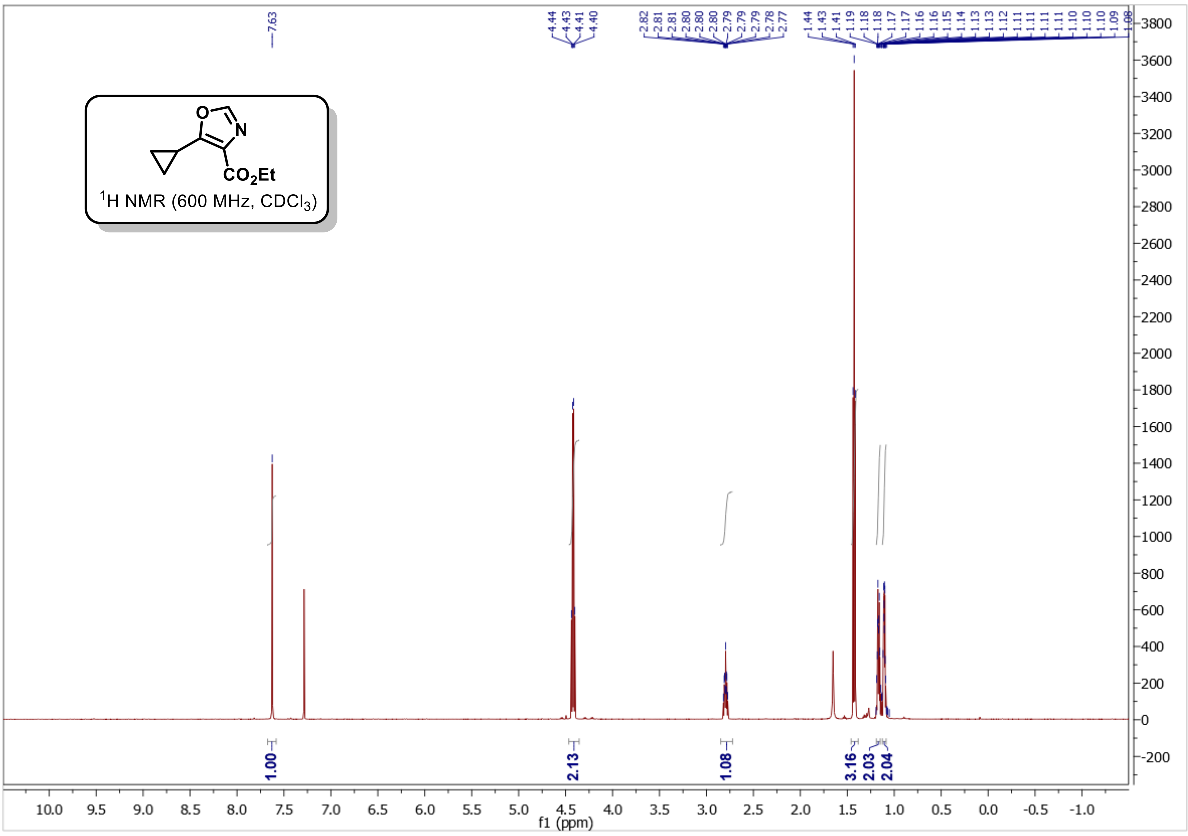

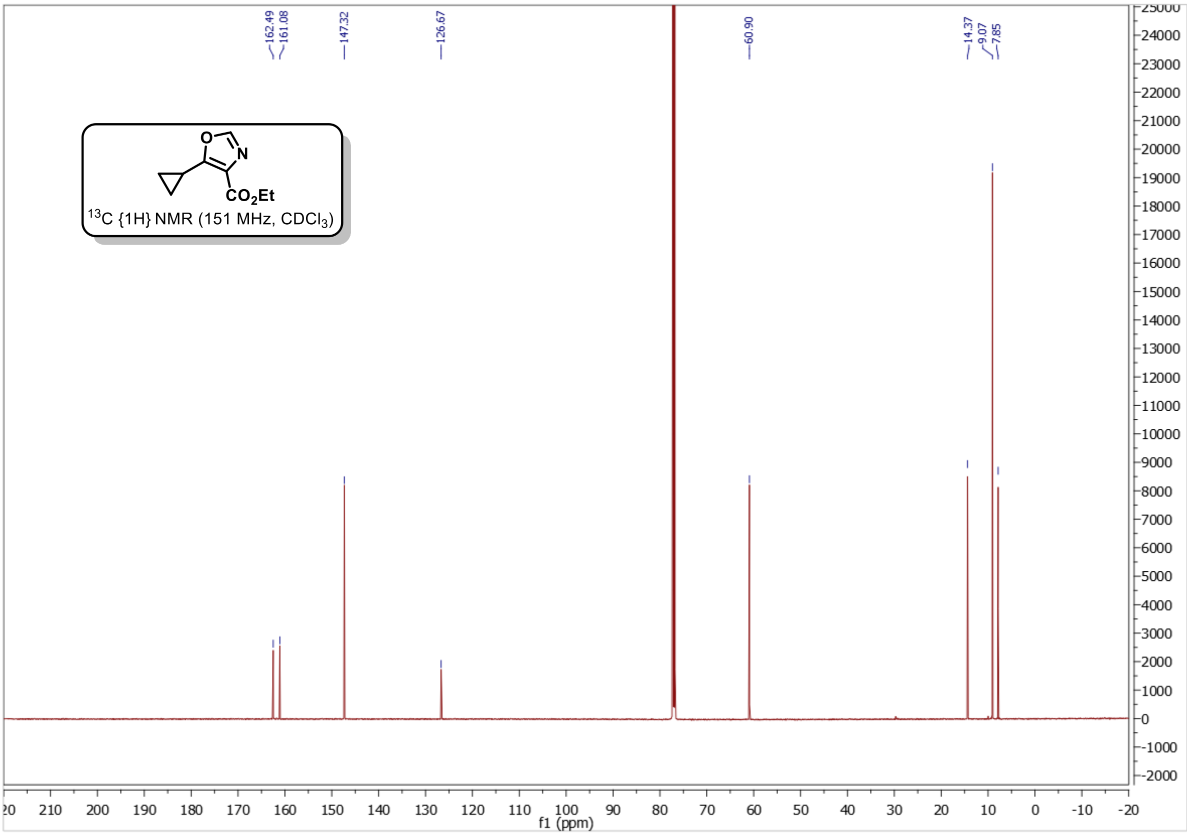

5-(1-phenylethyl)-4-tosyloxazole (3c'd):

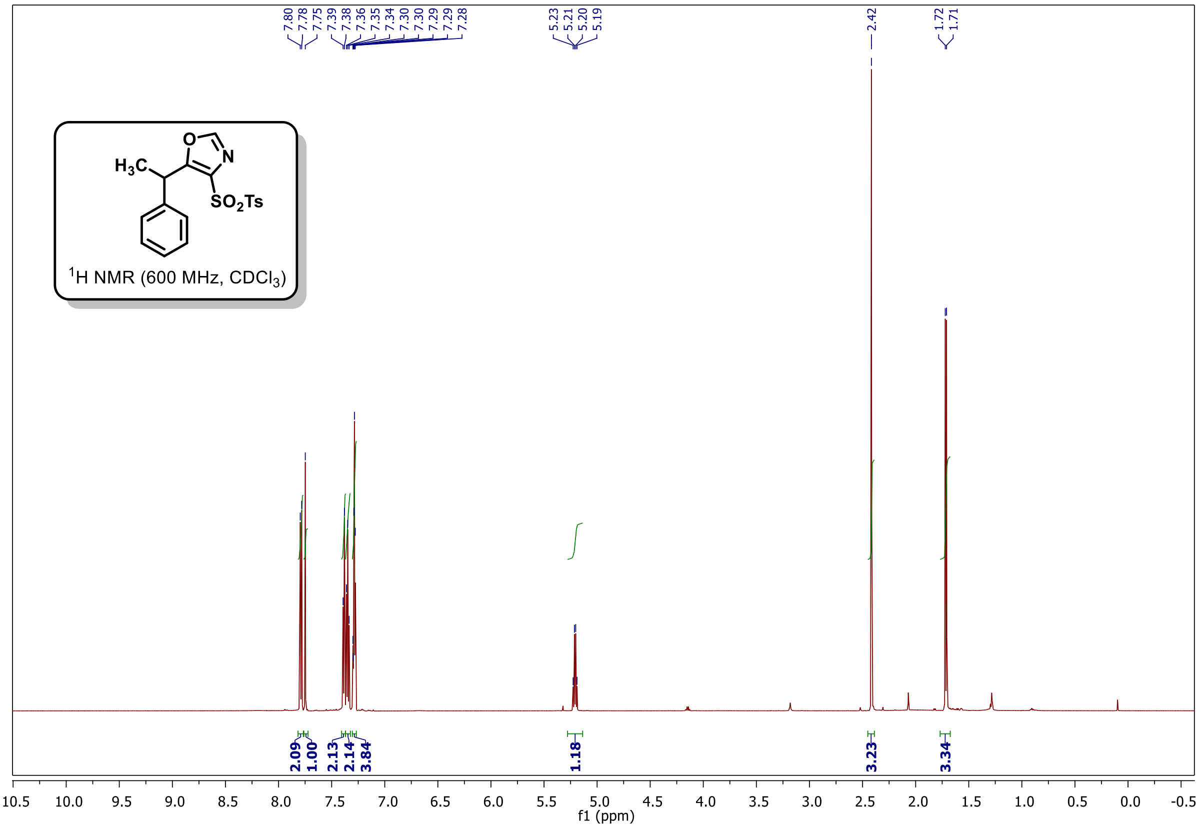

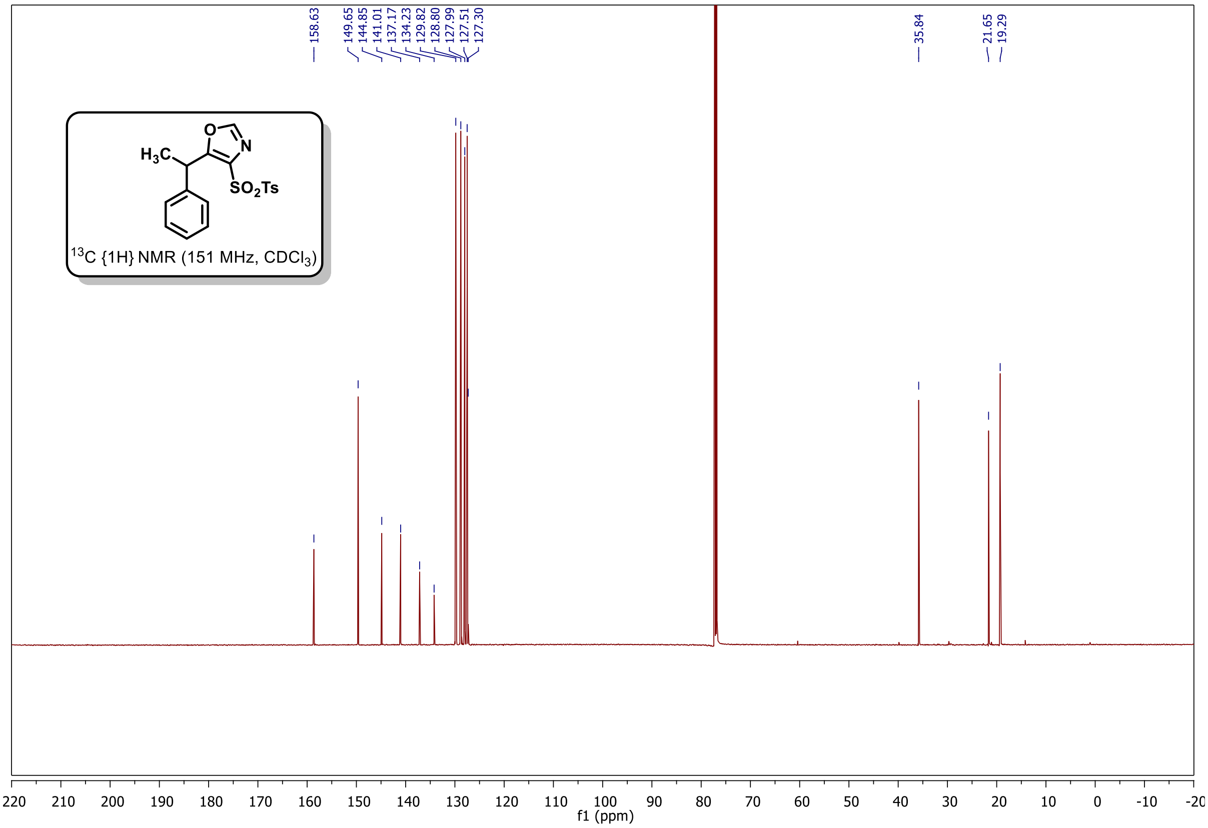

Ethyl 5-(((diphenylmethylene)amino)methyl)oxazole-4-carboxylate (3d'a):

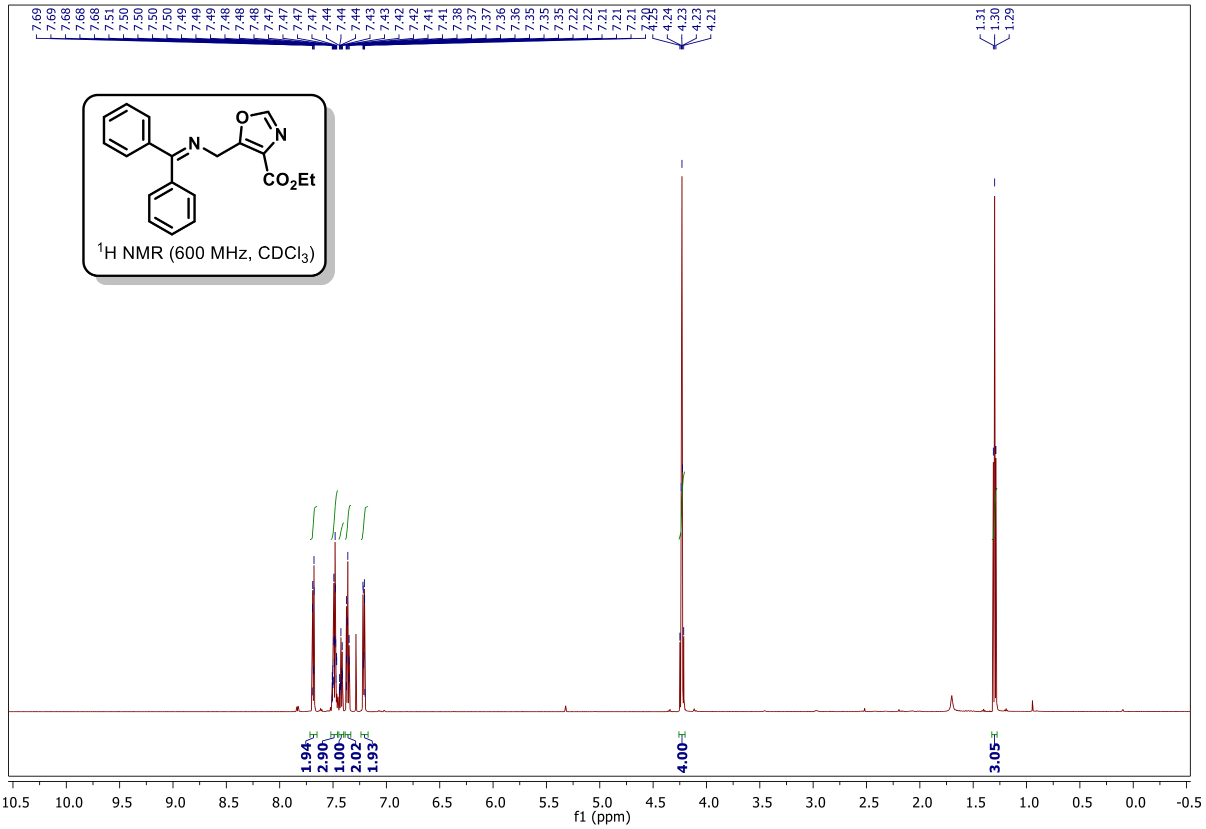

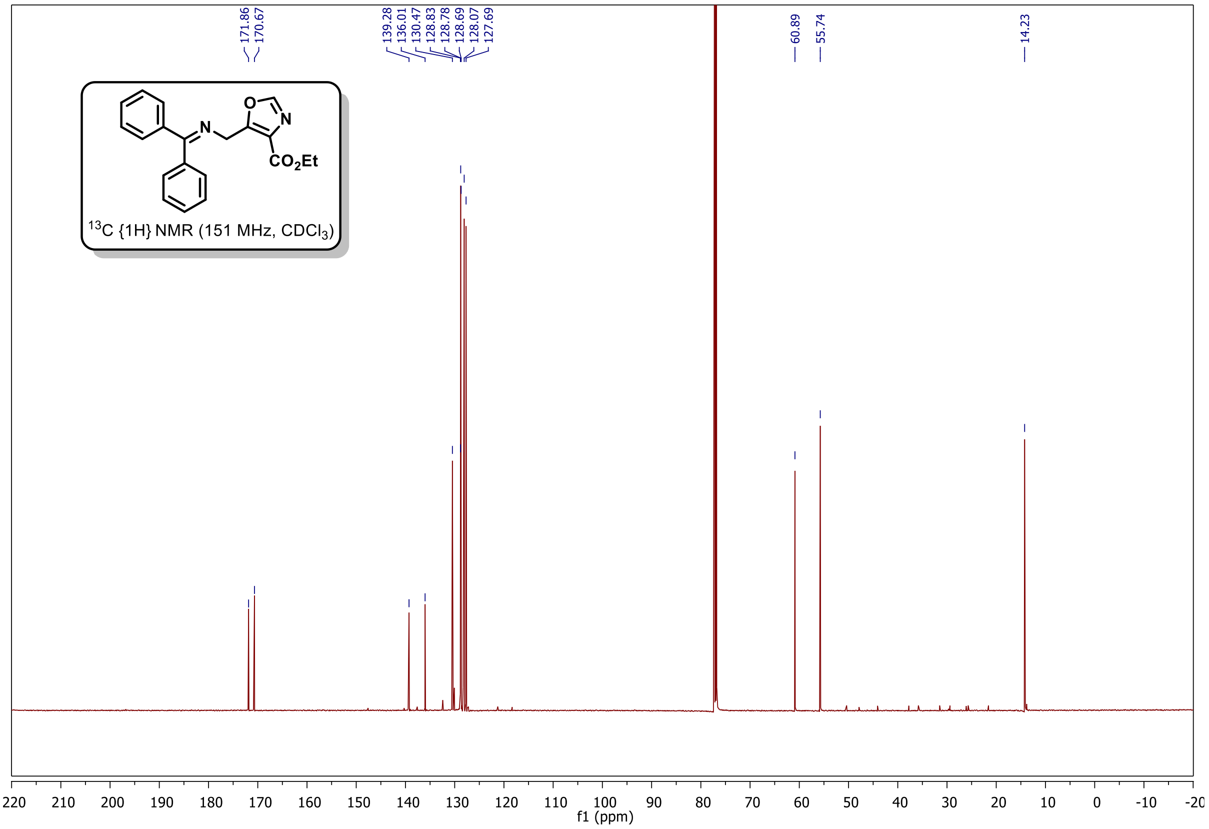

Ethyl (*Z*)-5-styryloxazole-4-carboxylate (3e'a):

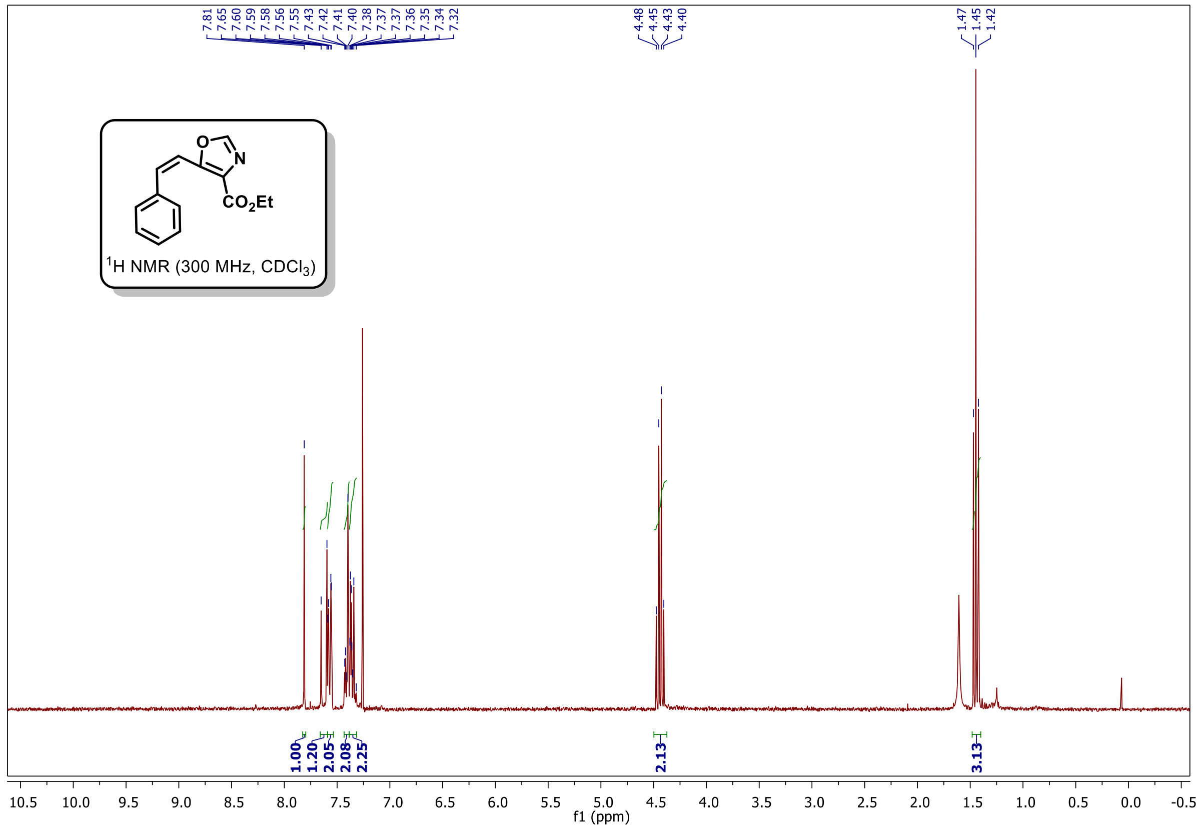

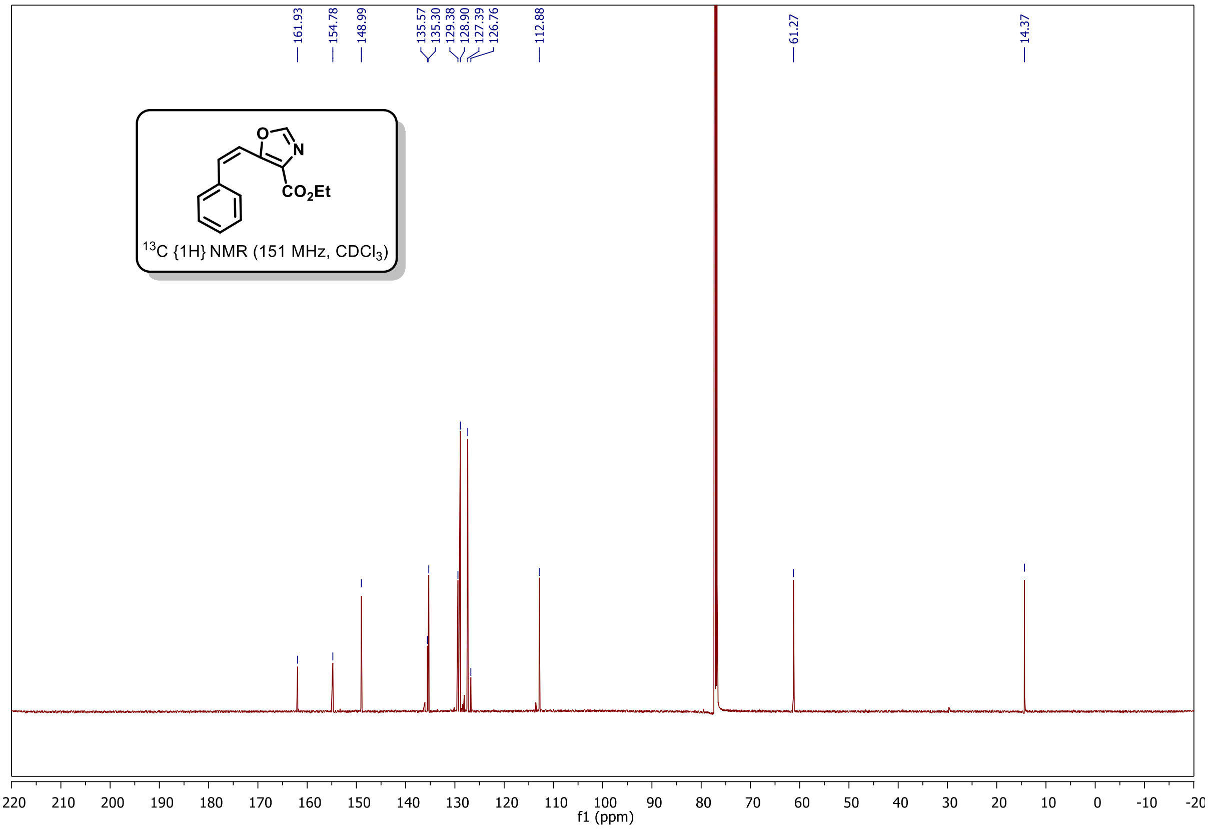

5-(furan-2-yl)-4-tosyloxazole (3f'd):

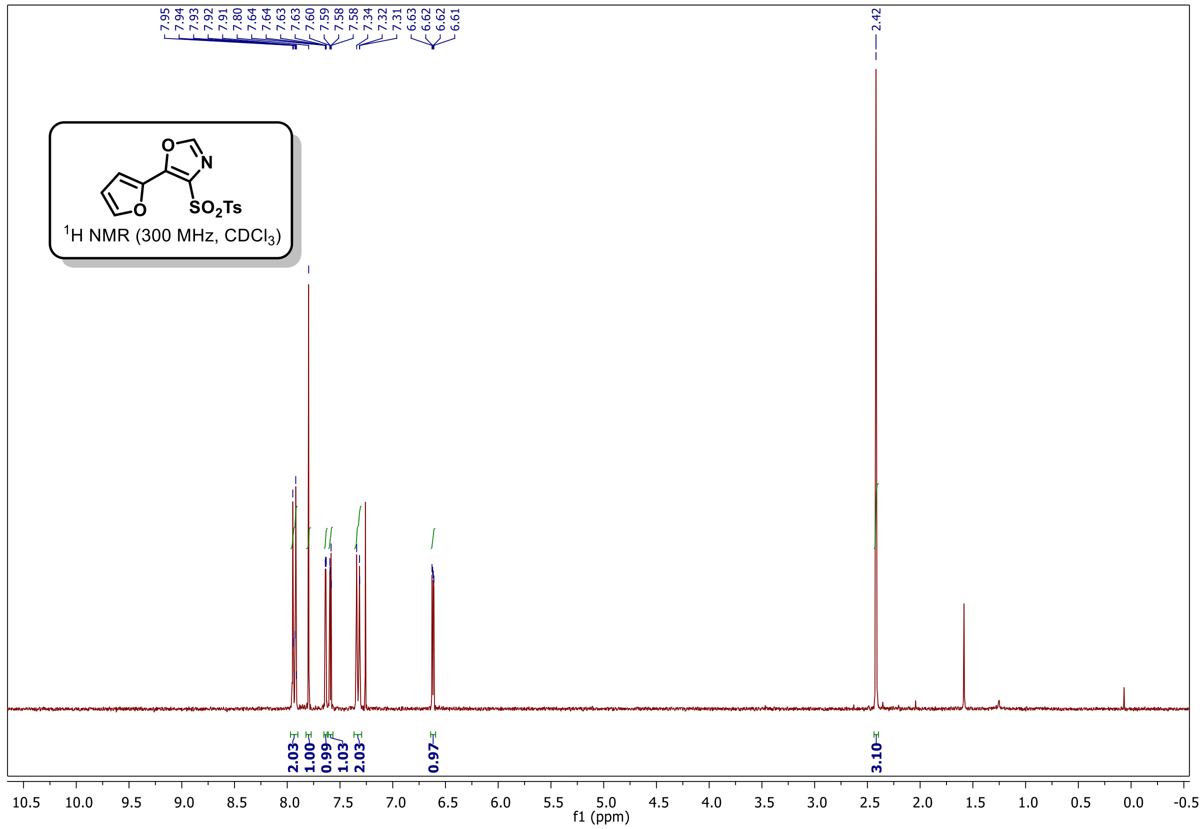

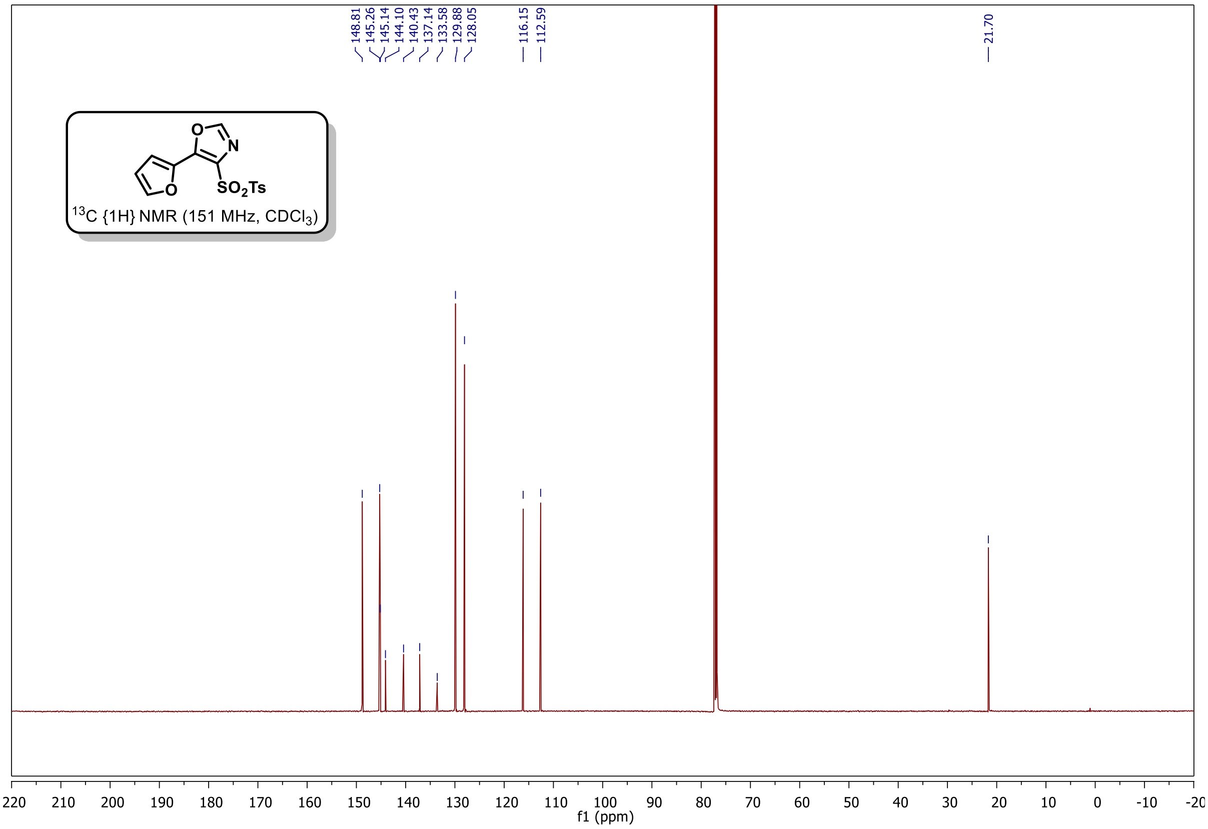

5-(thiophen-2-yl)-4-tosyloxazole (3qd):

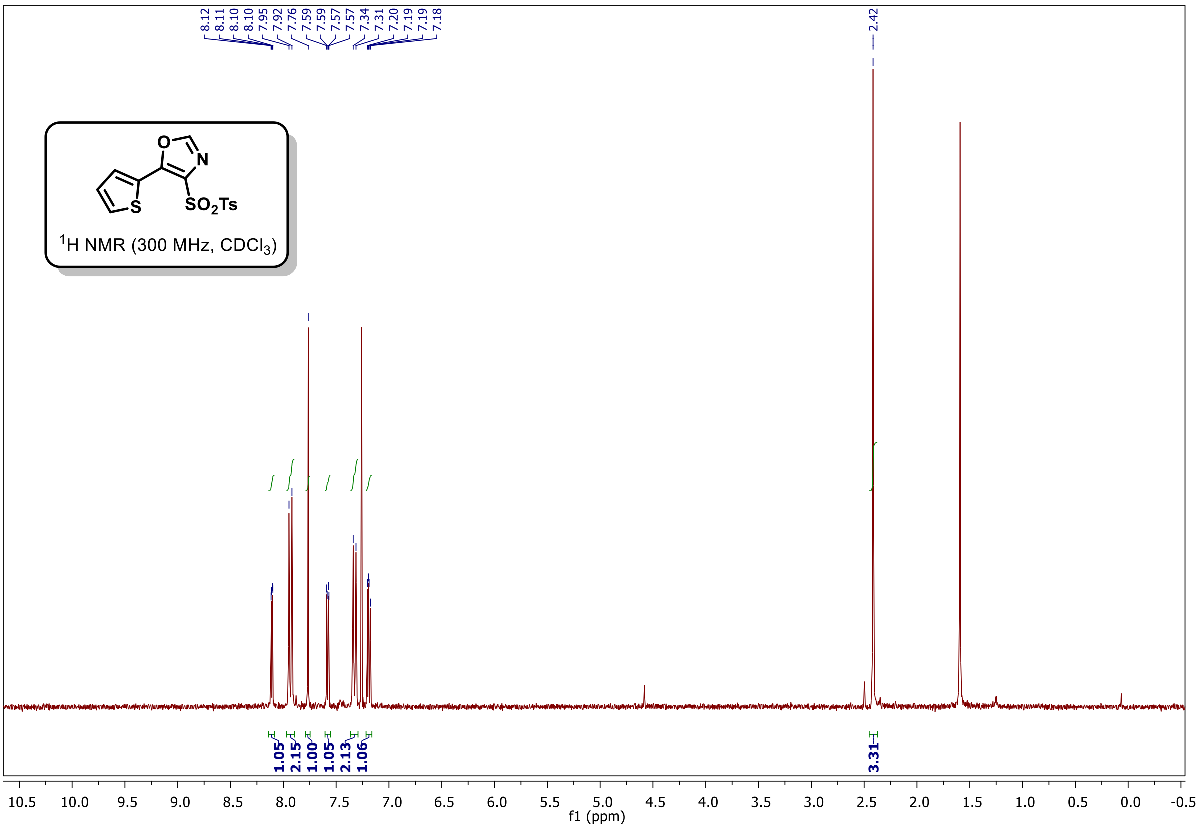

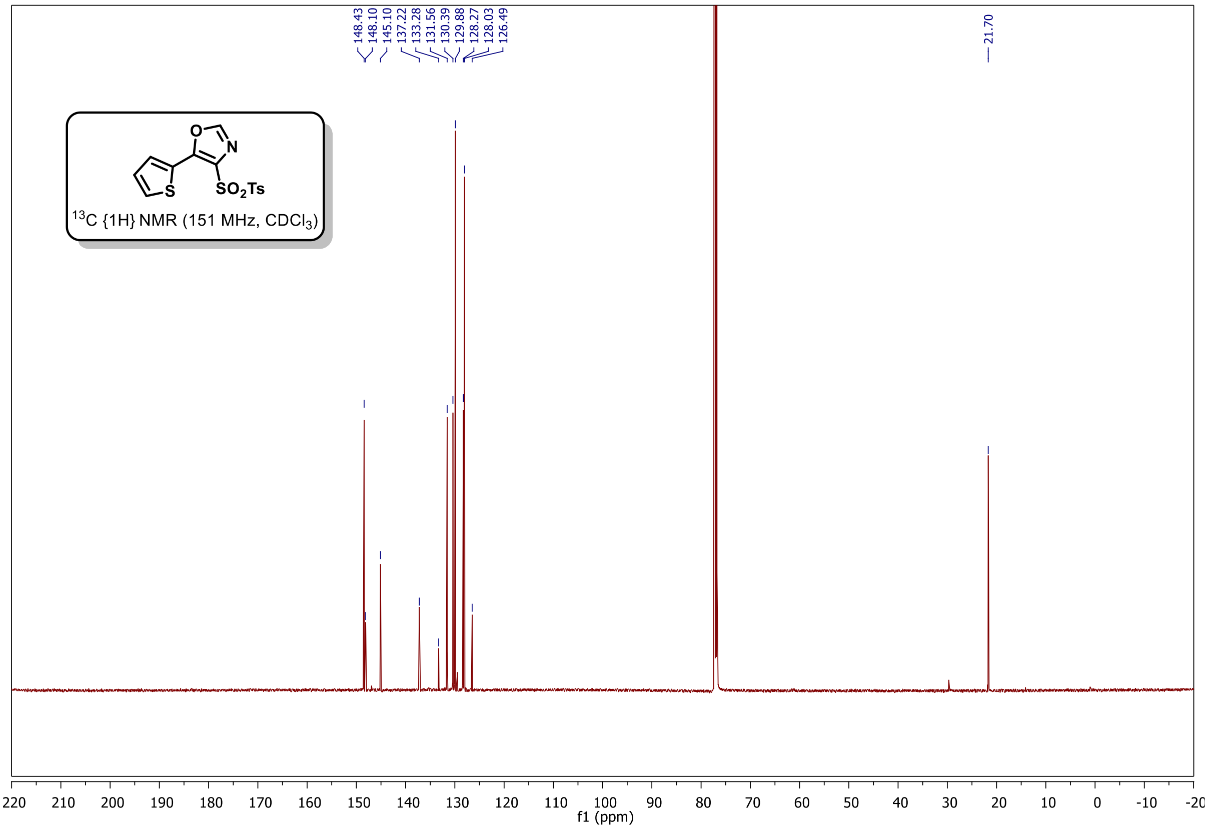

5-(3-fluorophenyl)-4-tosyloxazole (3ad):

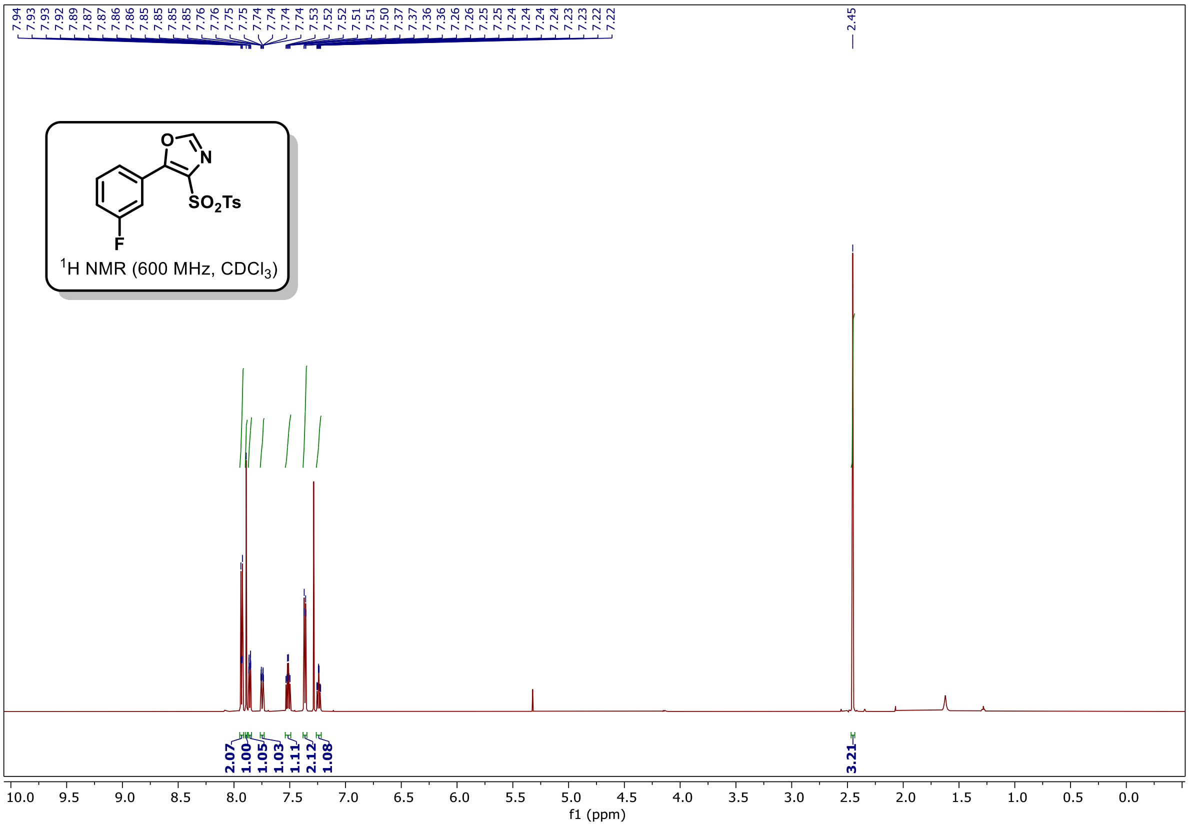

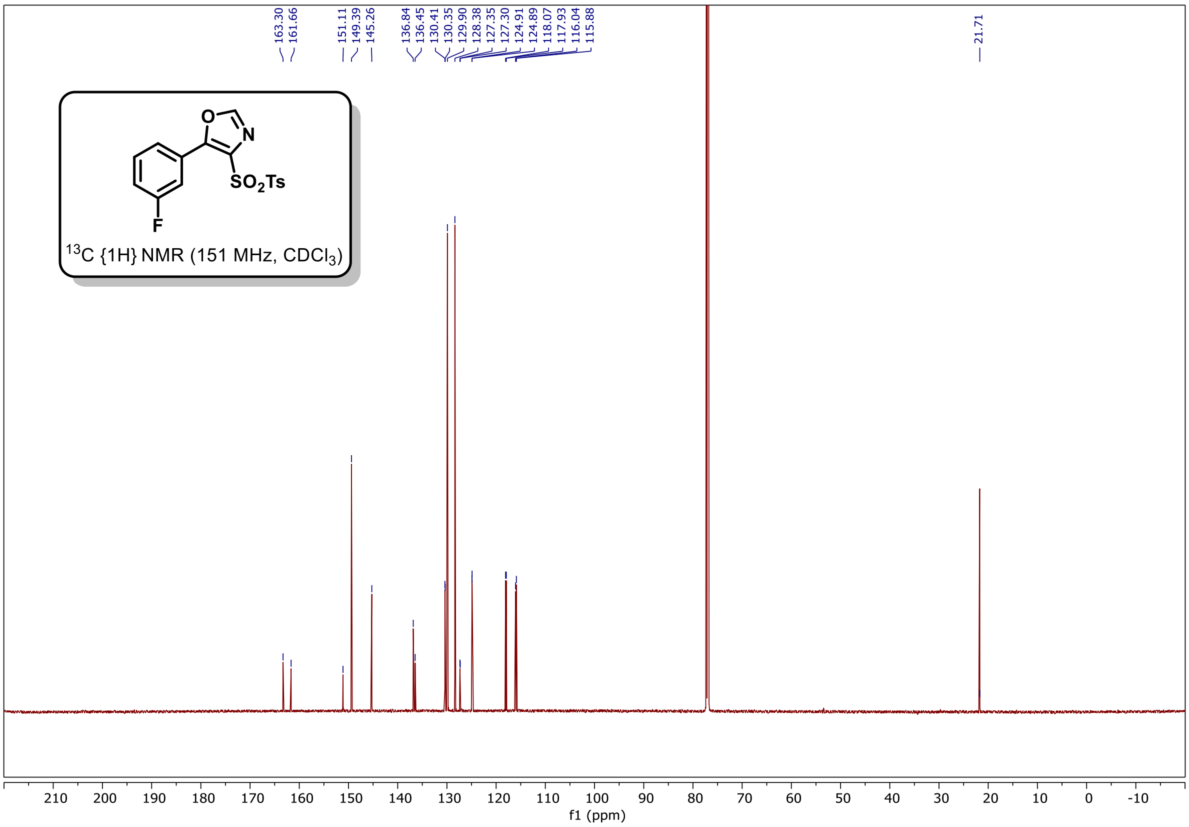

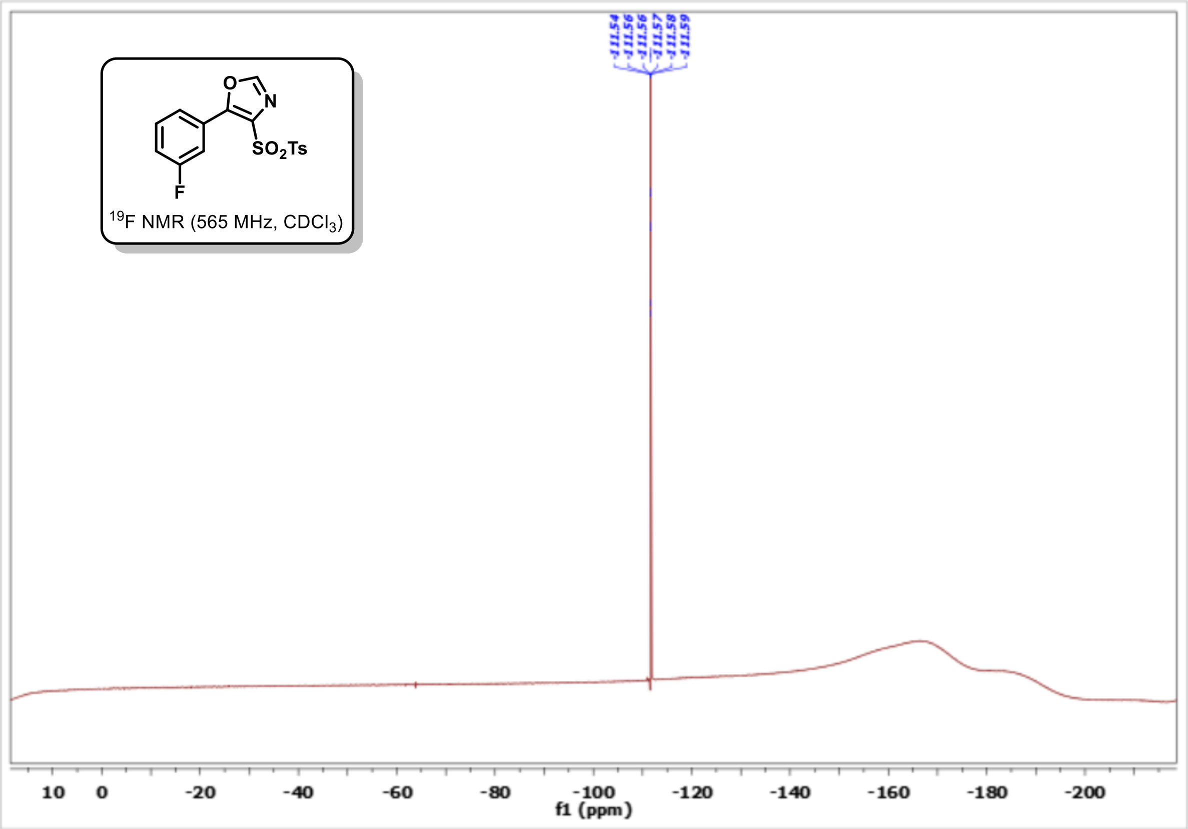

-111.54
-111.56
-111.56
-111.57
-111.58
-111.59
SO2Ts
O
N
F
19F NMR (565 MHz, CDCl3)
10
0
-20
-40
-60
-80
-100
-120
-140
-160
-180
-200
f1 (ppm)

Phenyl(4-tosyloxazol-5-yl)methanone (3ed):

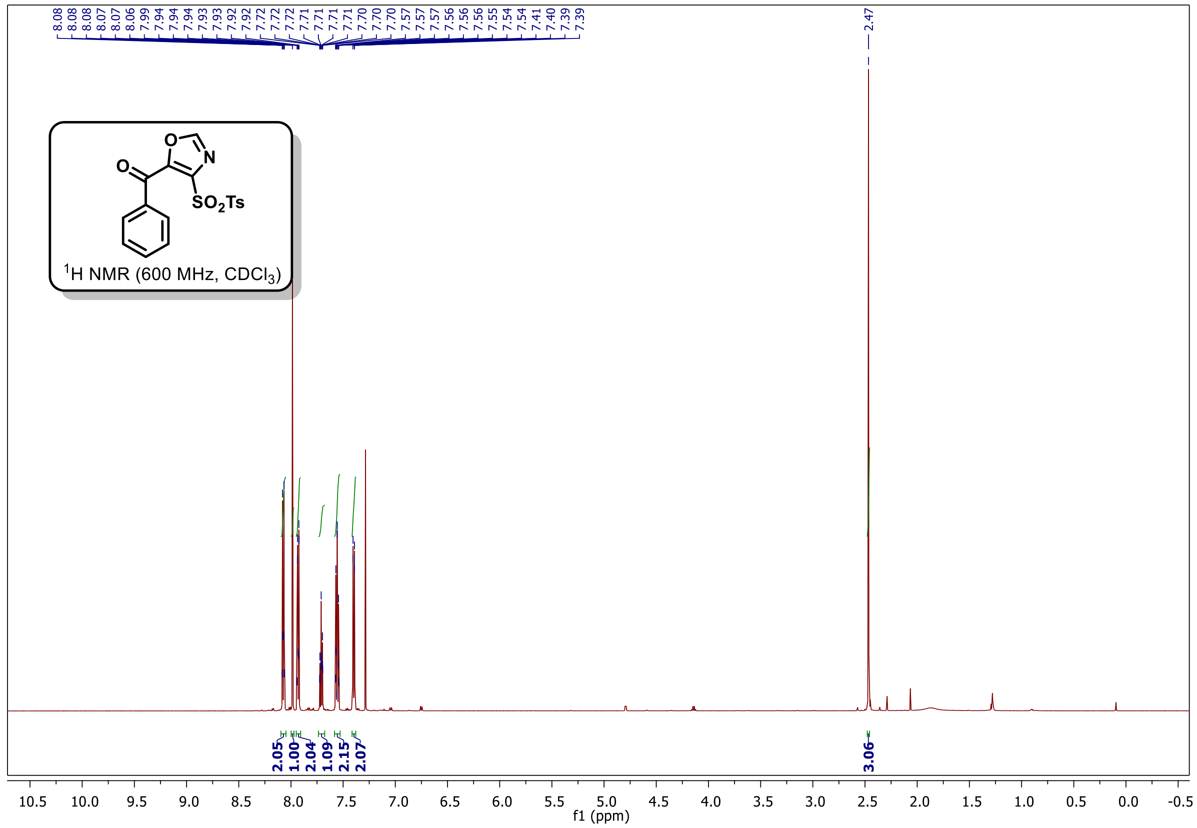

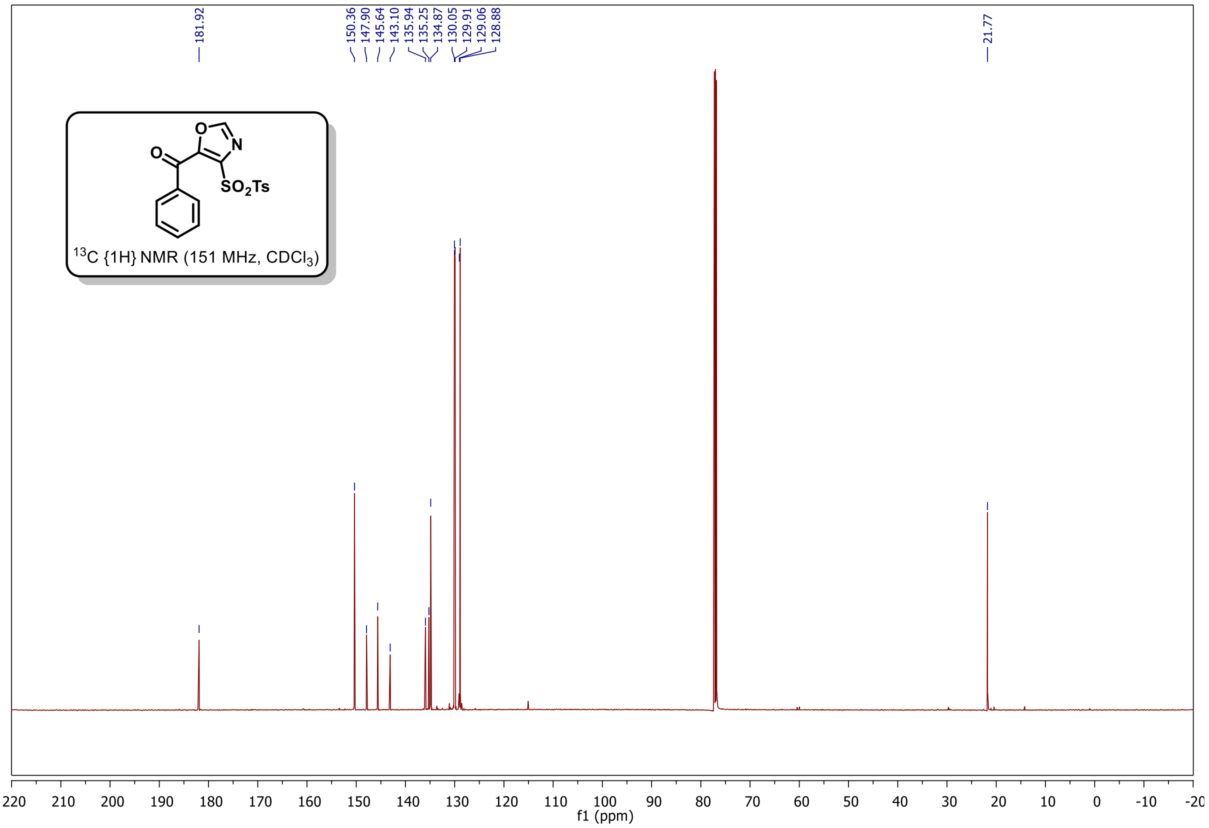

5-(benzo[d][1,3]dioxol-5-yl)-4-tosyloxazole (3hd):

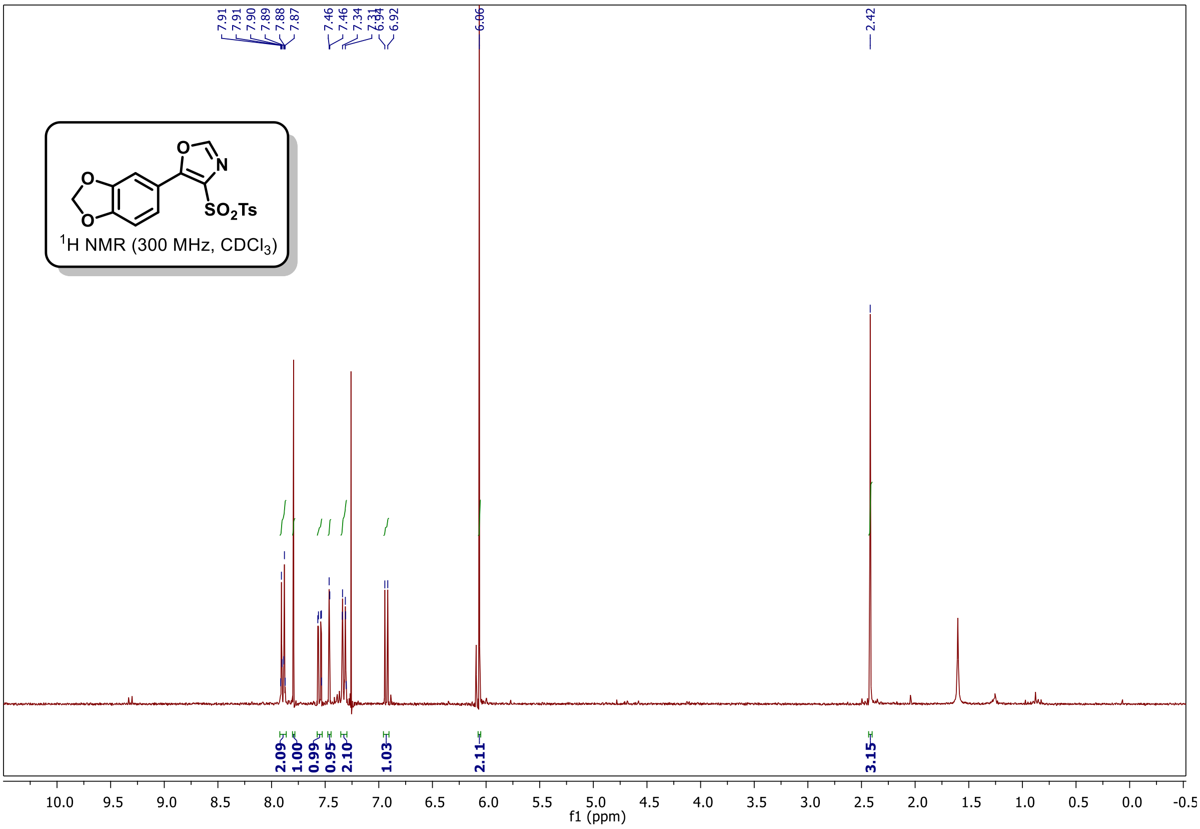

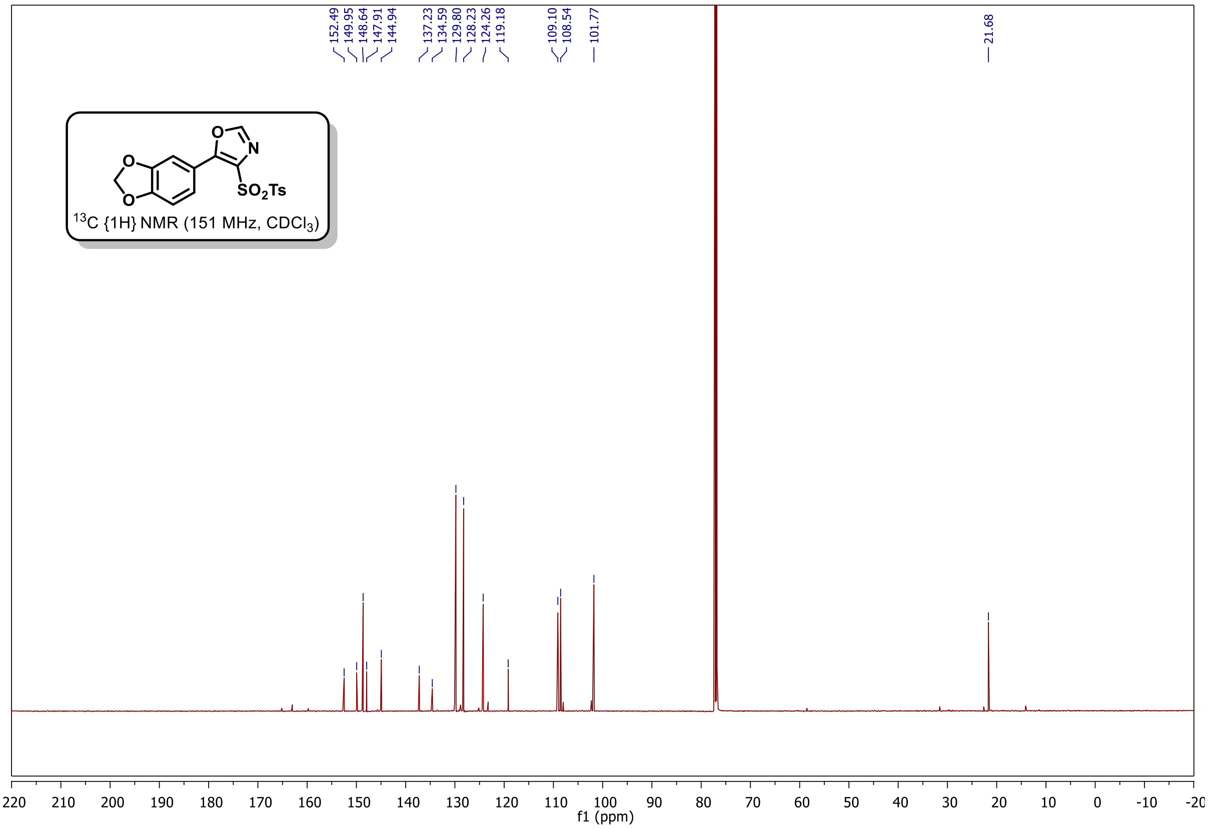

5-(quinoxalin-6-yl)-4-tosyloxazole (3sd):

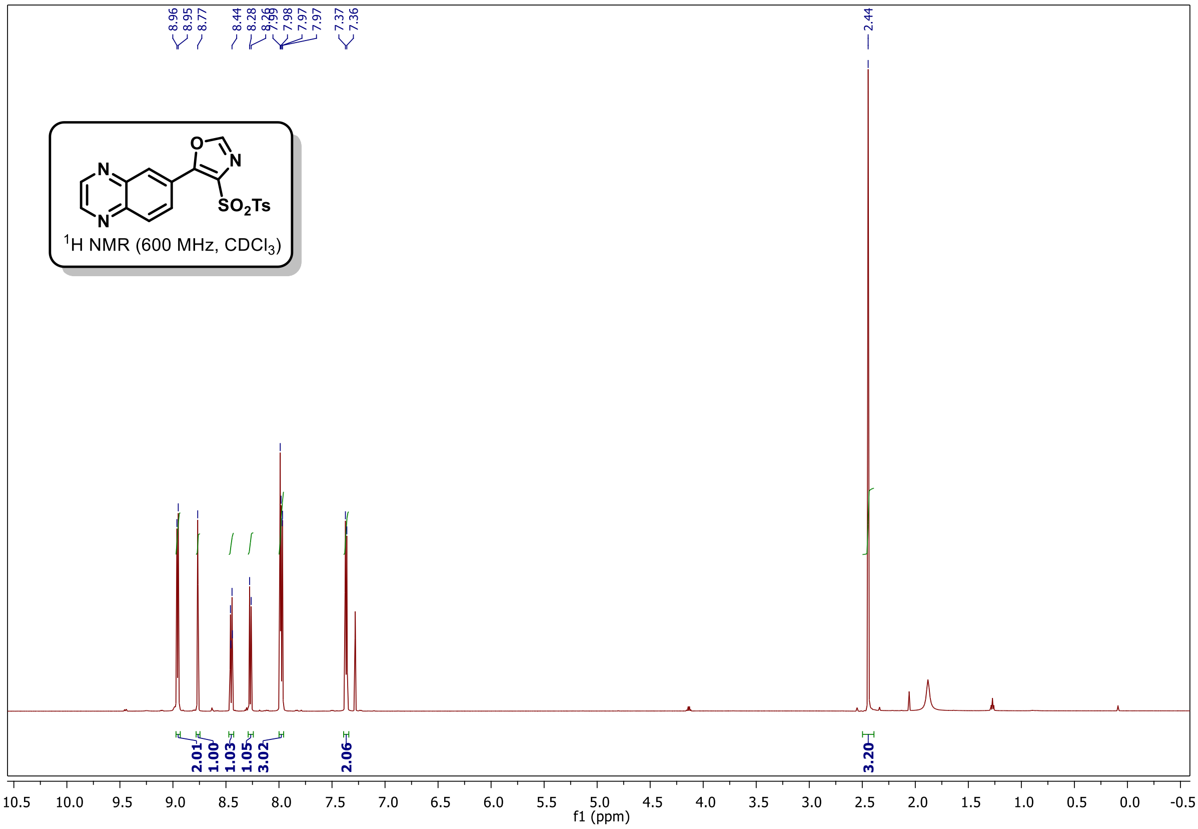

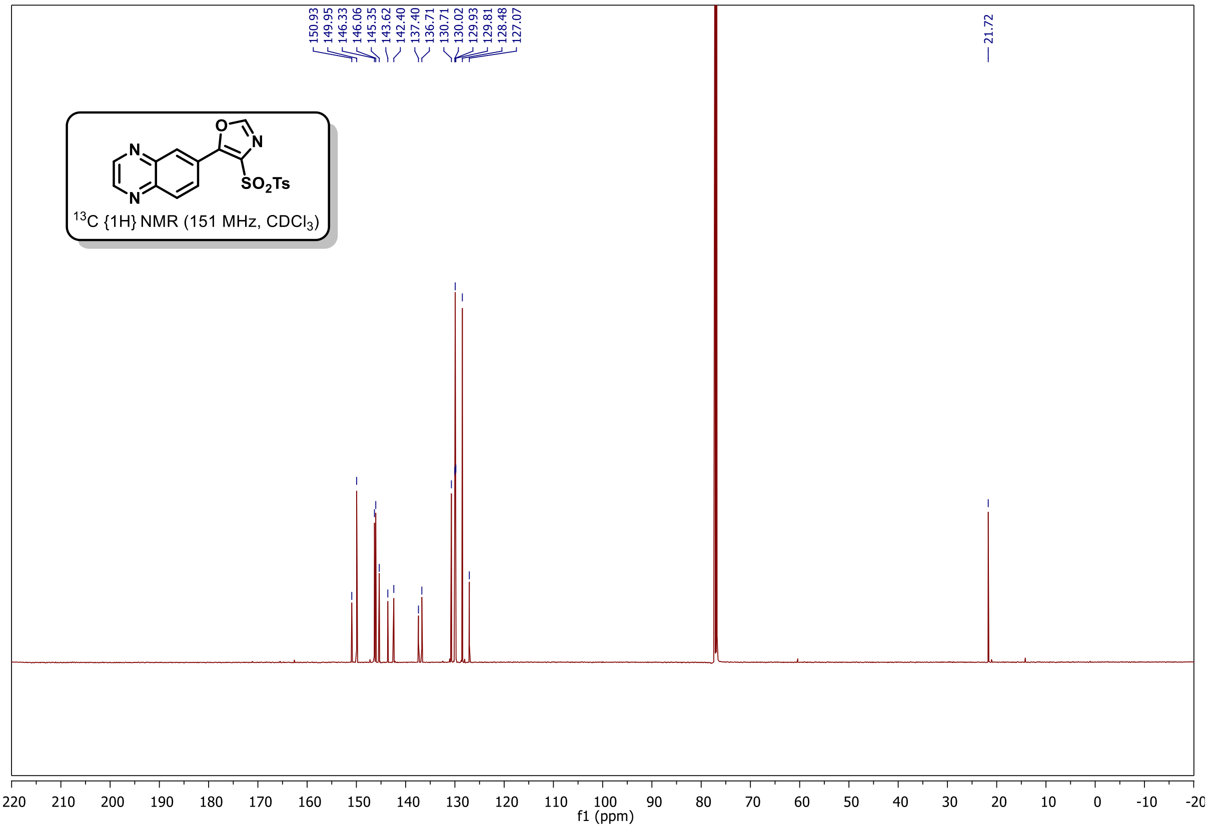

5-(6-methylpyridin-3-yl)-4-tosyloxazole (3md):

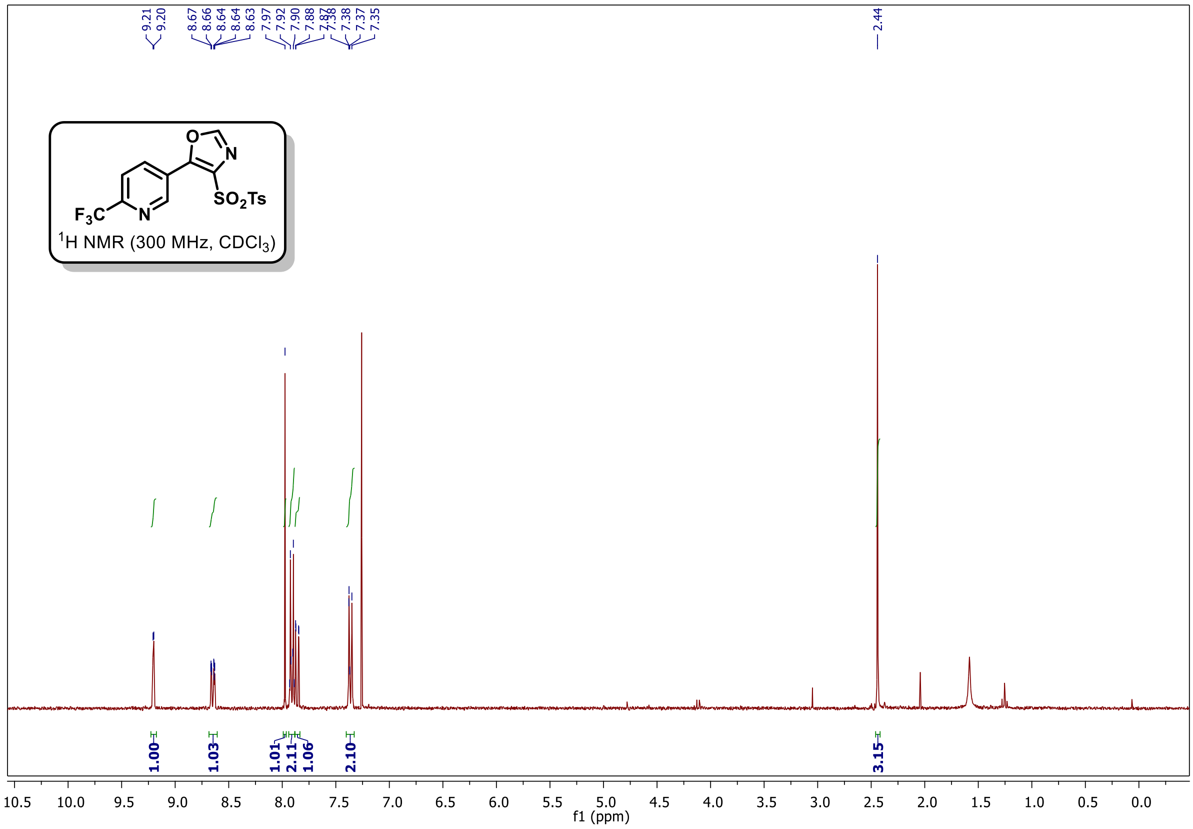

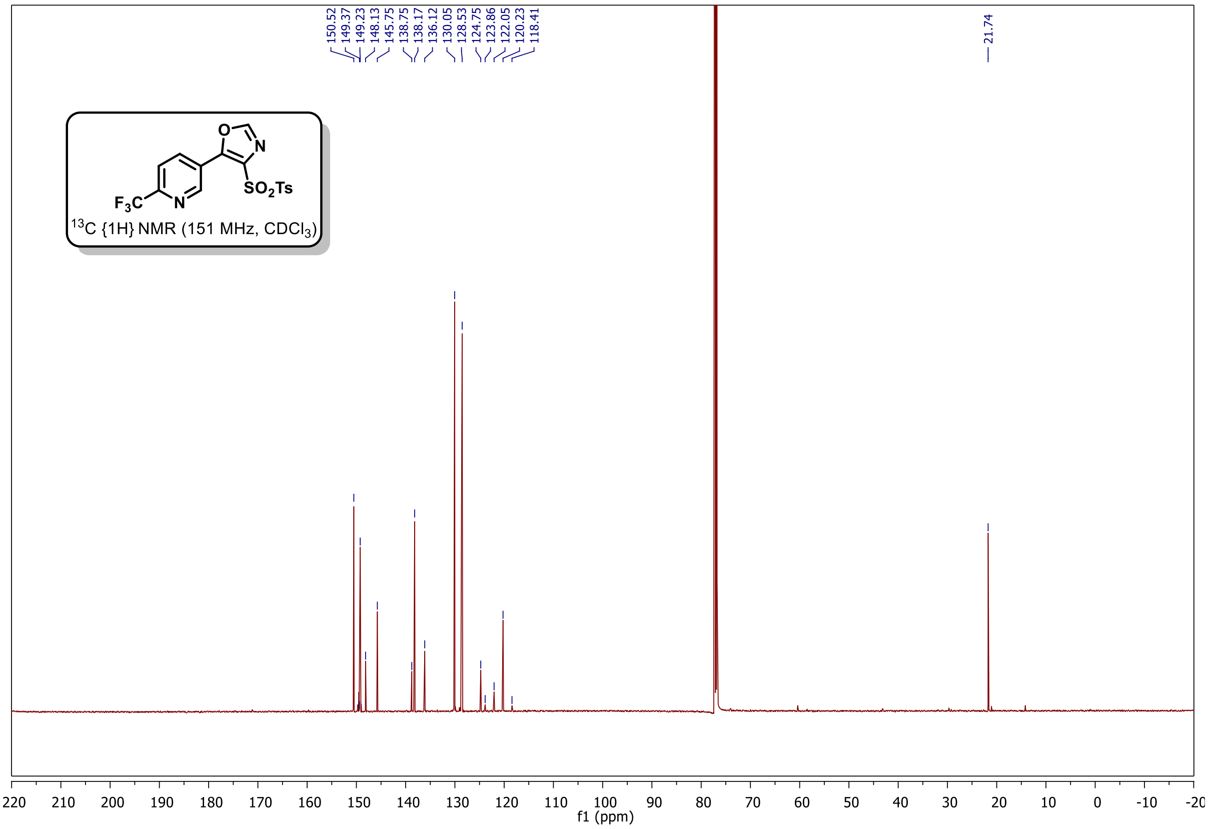

5-(6-chloropyridin-3-yl)-4-tosyloxazole (3kd):

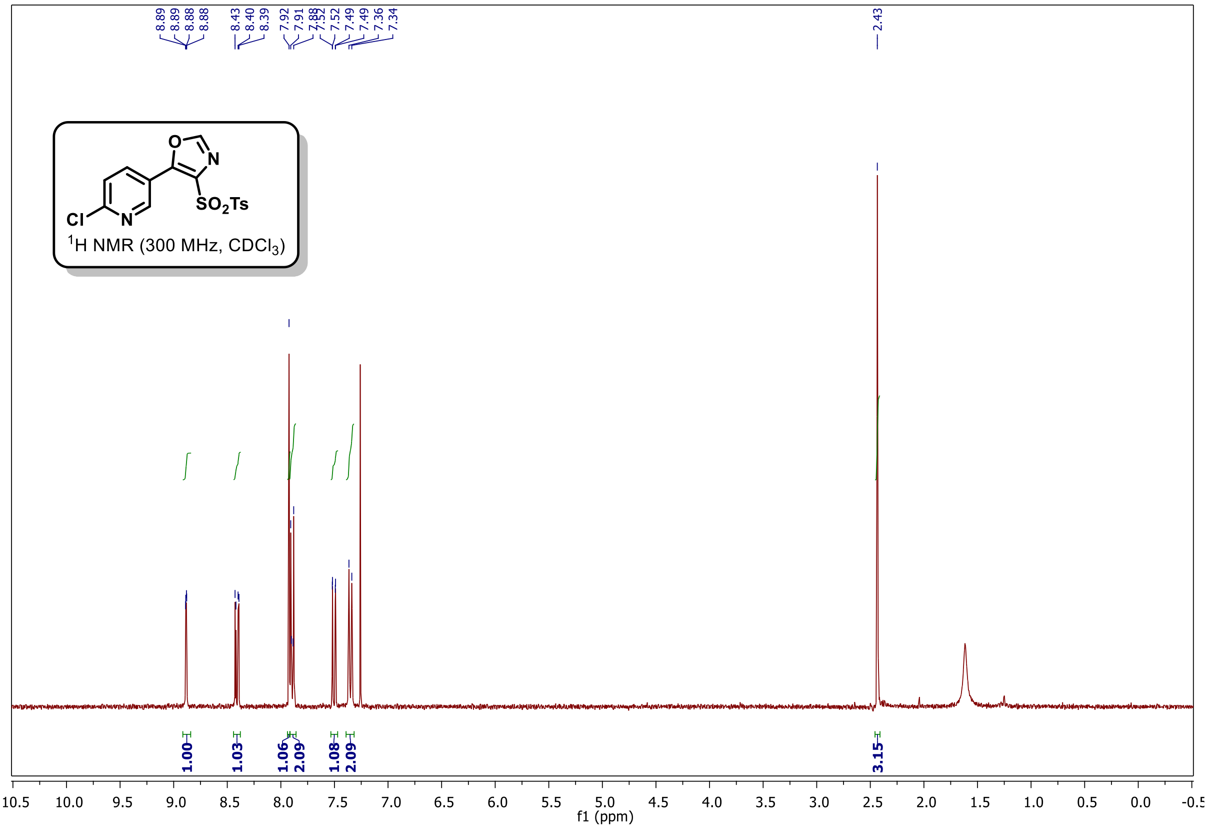

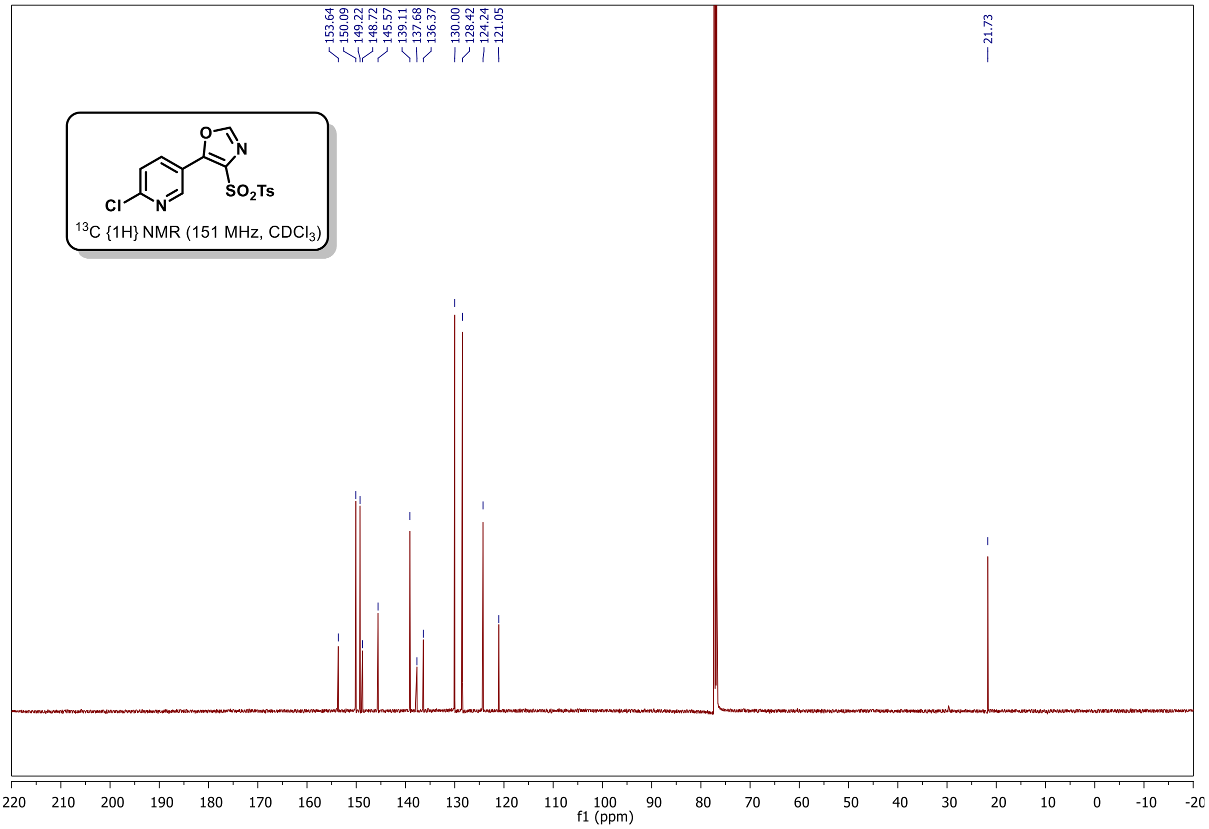

5-(1-methyl-1H-indol-2-yl)-4-tosyloxazole (3rd):

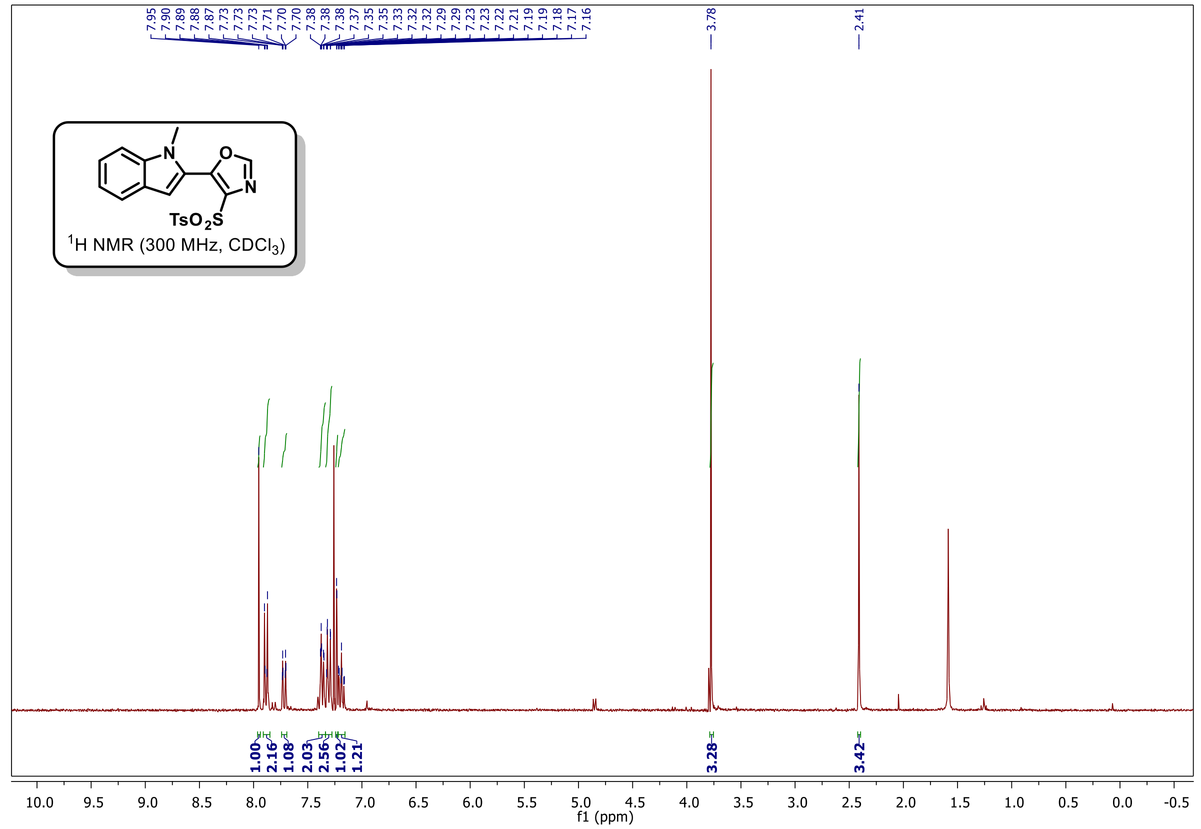

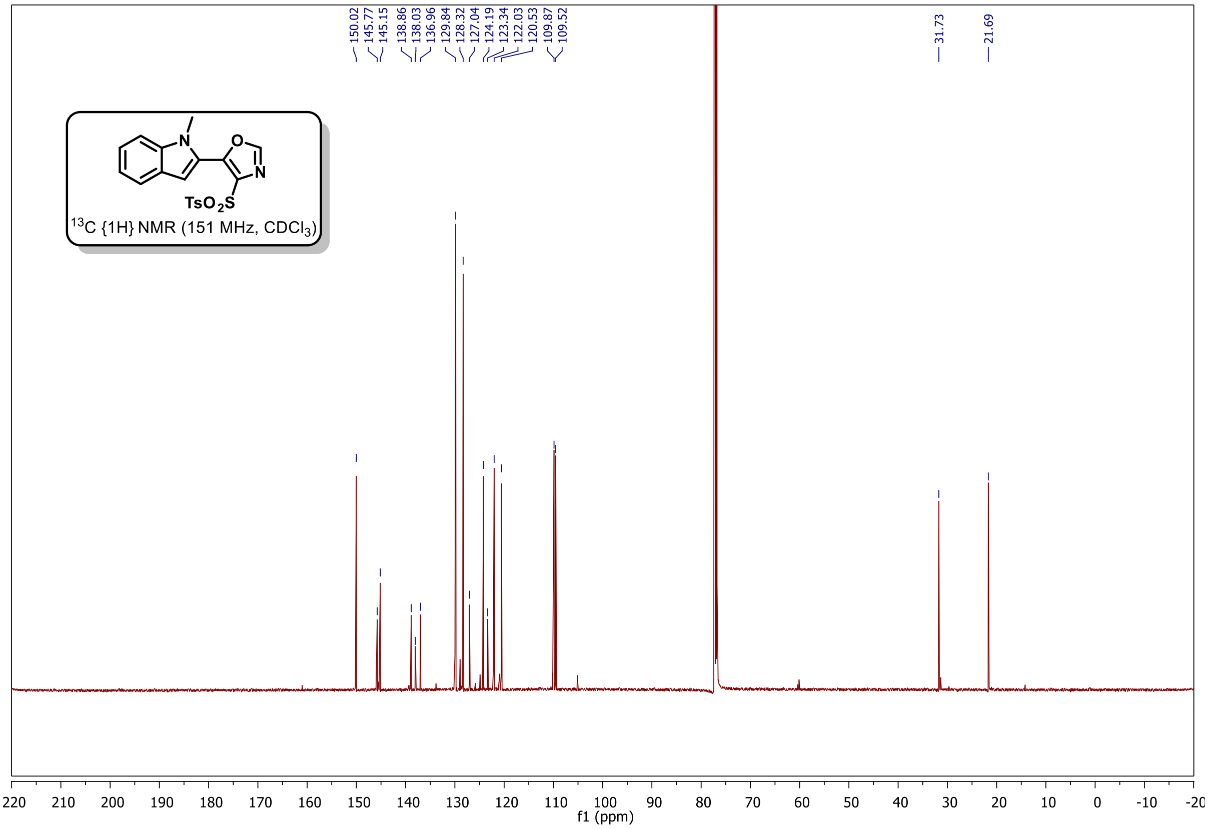

Methyl (*R*)-5-(4-(1,2-dithiolan-3-yl)butyl)oxazole-4-carboxylate (3g'c):

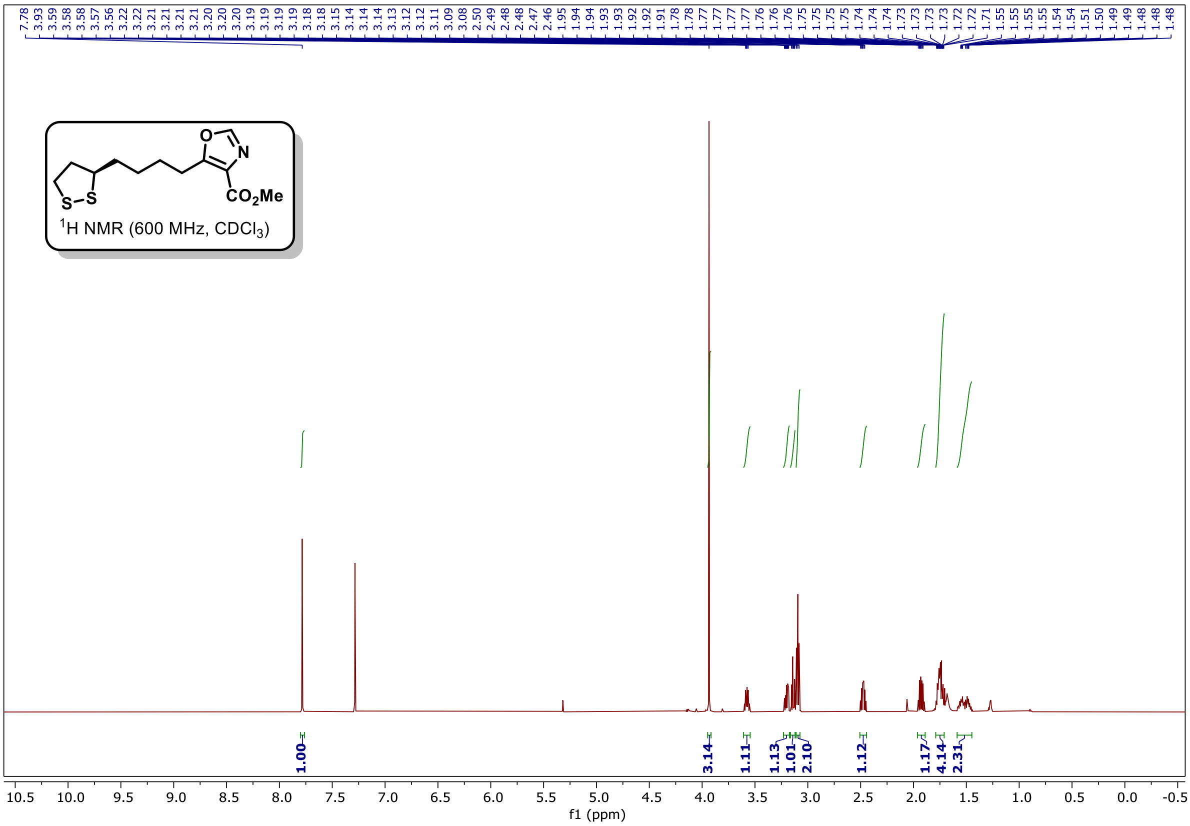

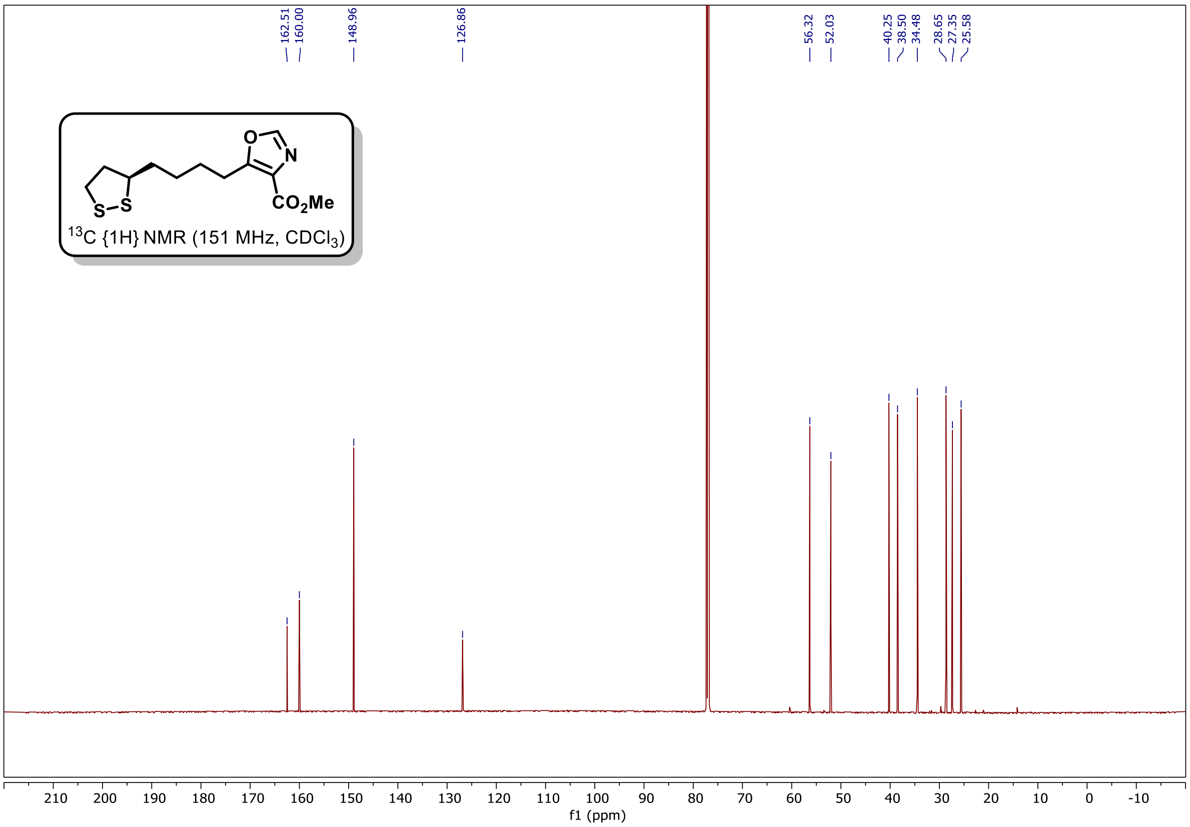

Ethyl 5-((8R,9S,13S,14S)-13-methyl-17-oxo-7,8,9,11,12,13,14,15,16,17-decahydro-6*H*-cyclopenta[a]phenanthrene-3-yl)oxazole-4-carboxylate (3h’a):

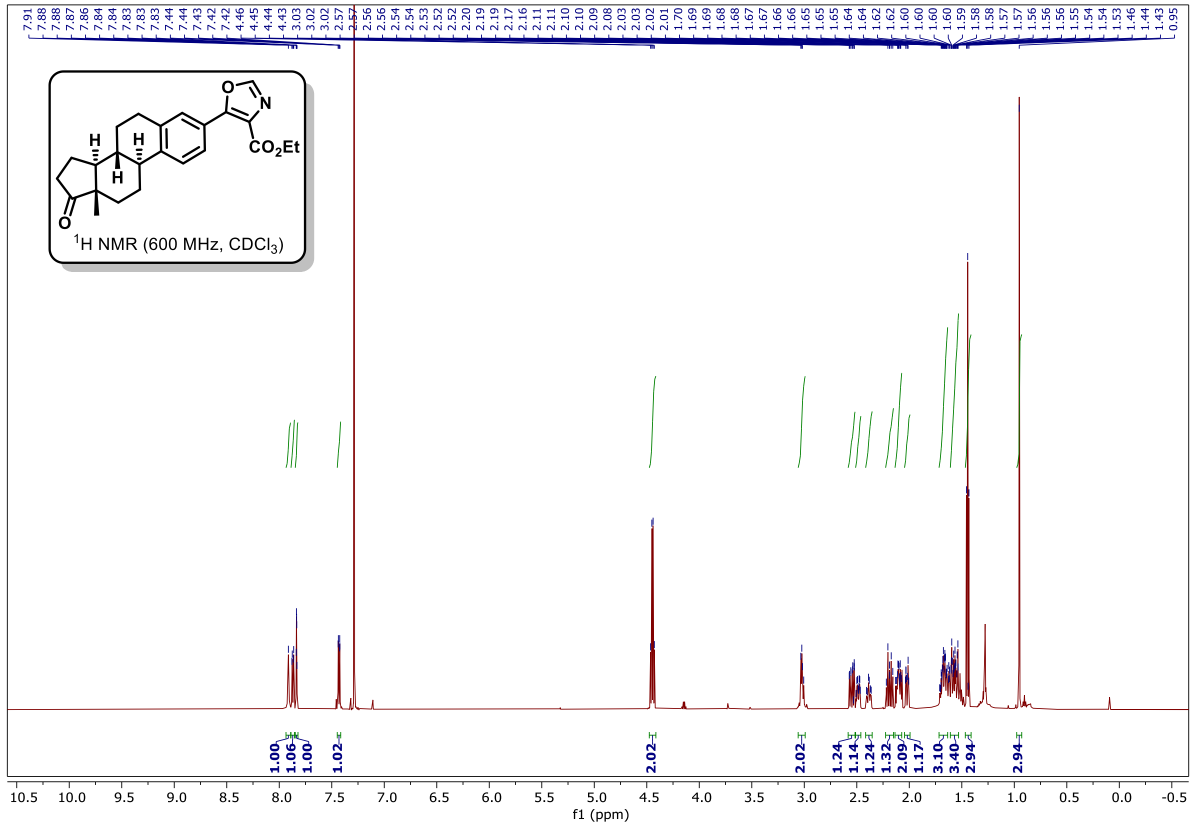

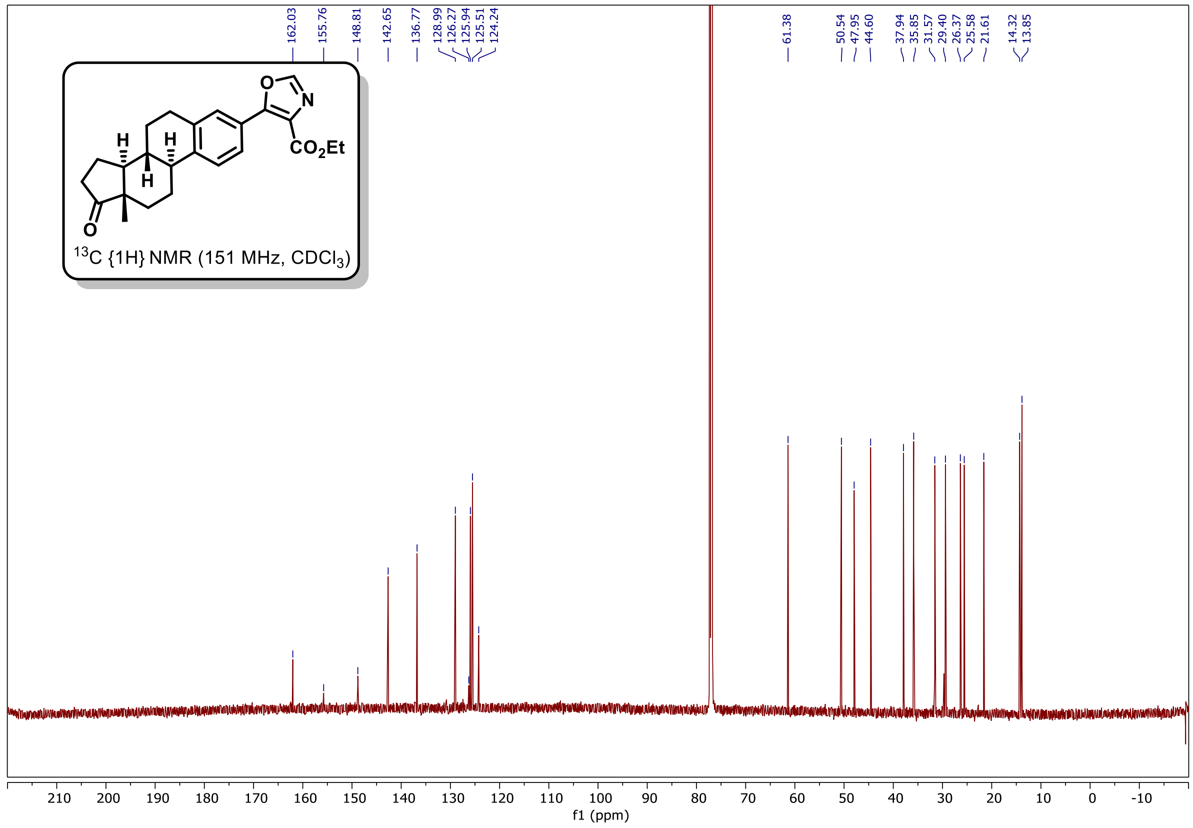

5-(heptan-4-yl)-4-tosyloxazole (3i'd):

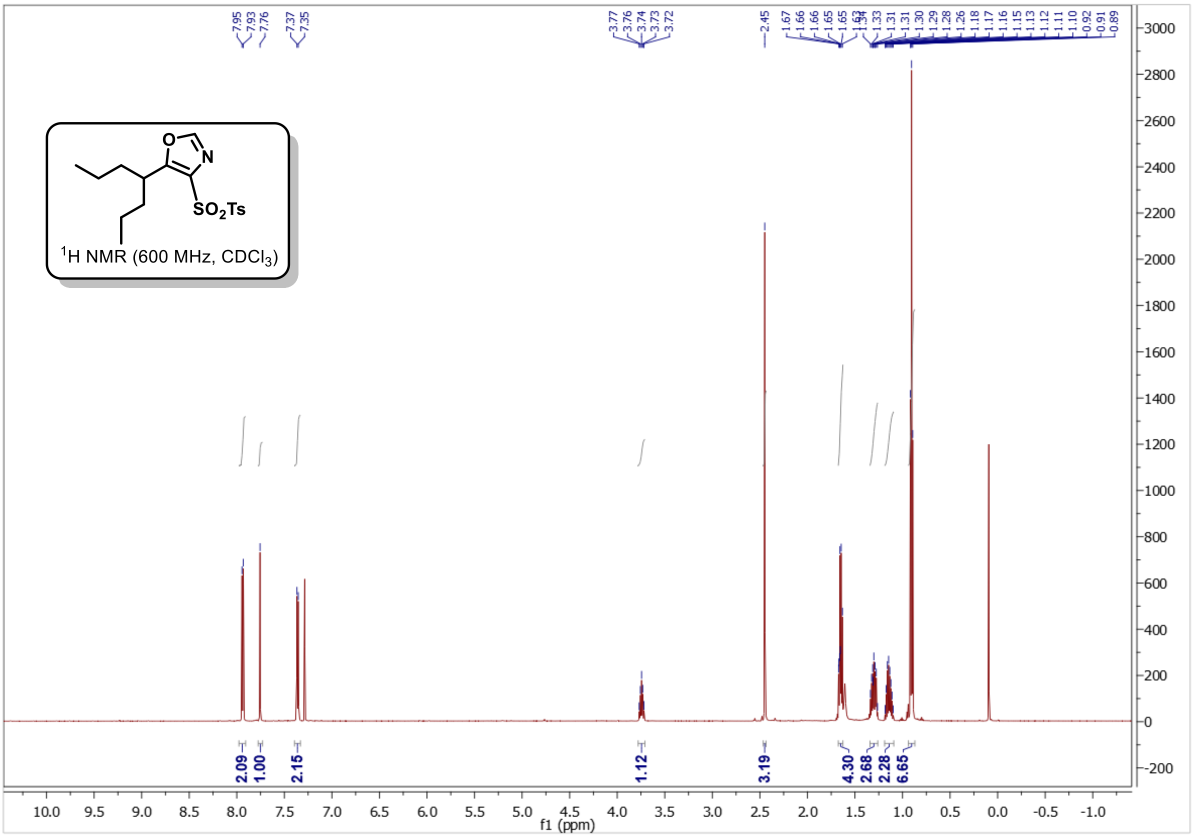

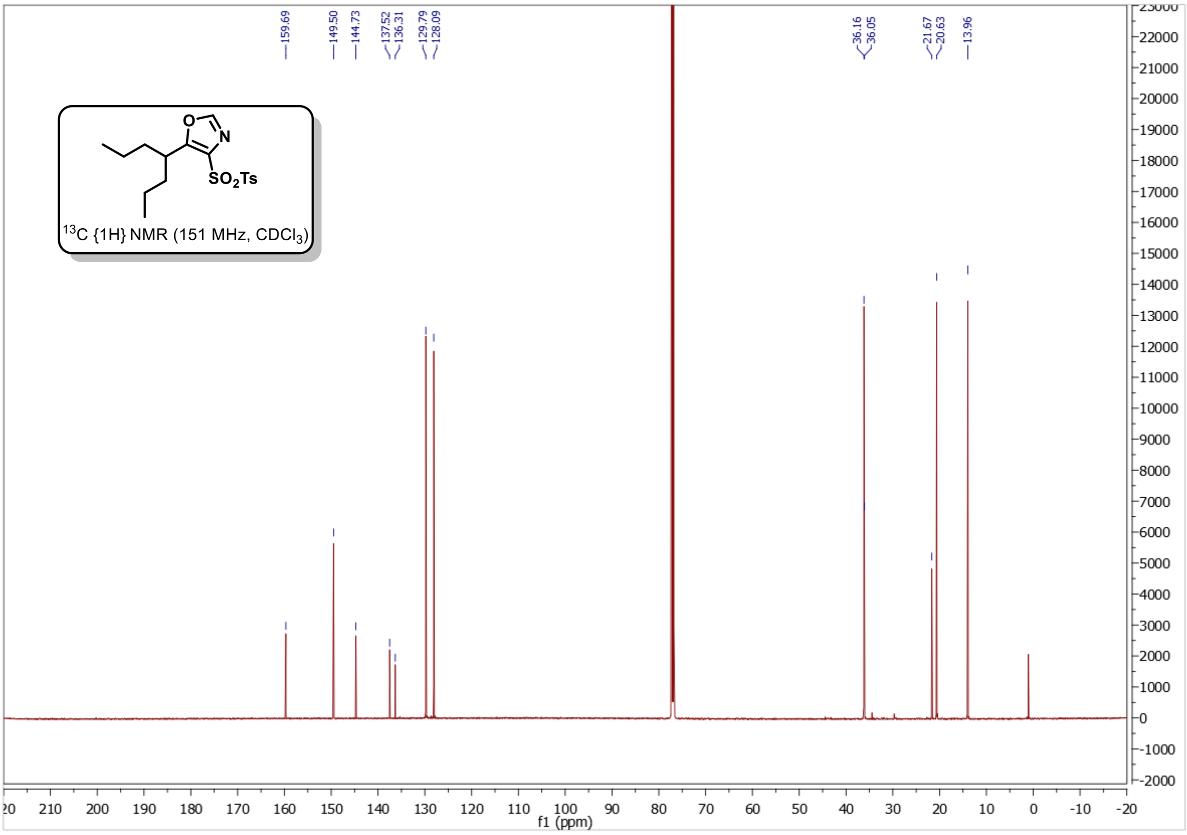

Ethyl 5-(4-(*N*,*N*-dipropylsulfamoyl)phenyl)oxazole-4-carboxylate (3j'a):

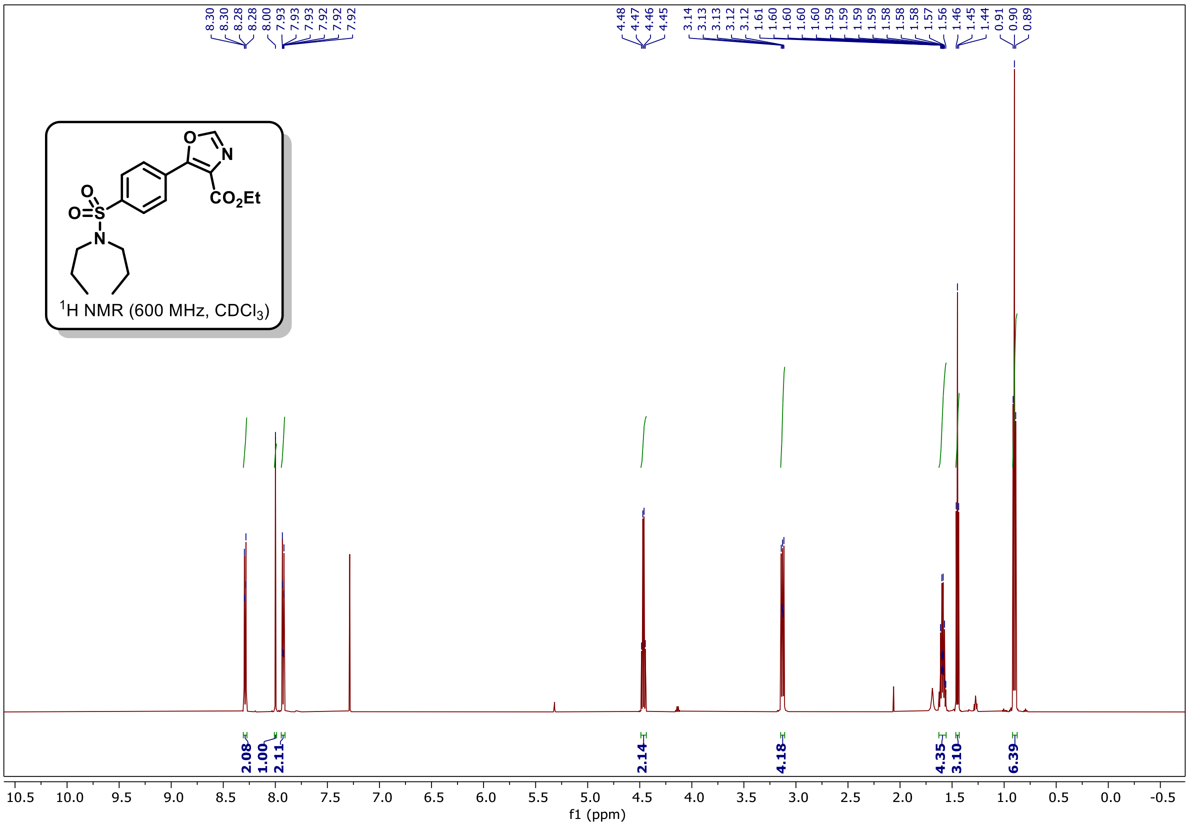

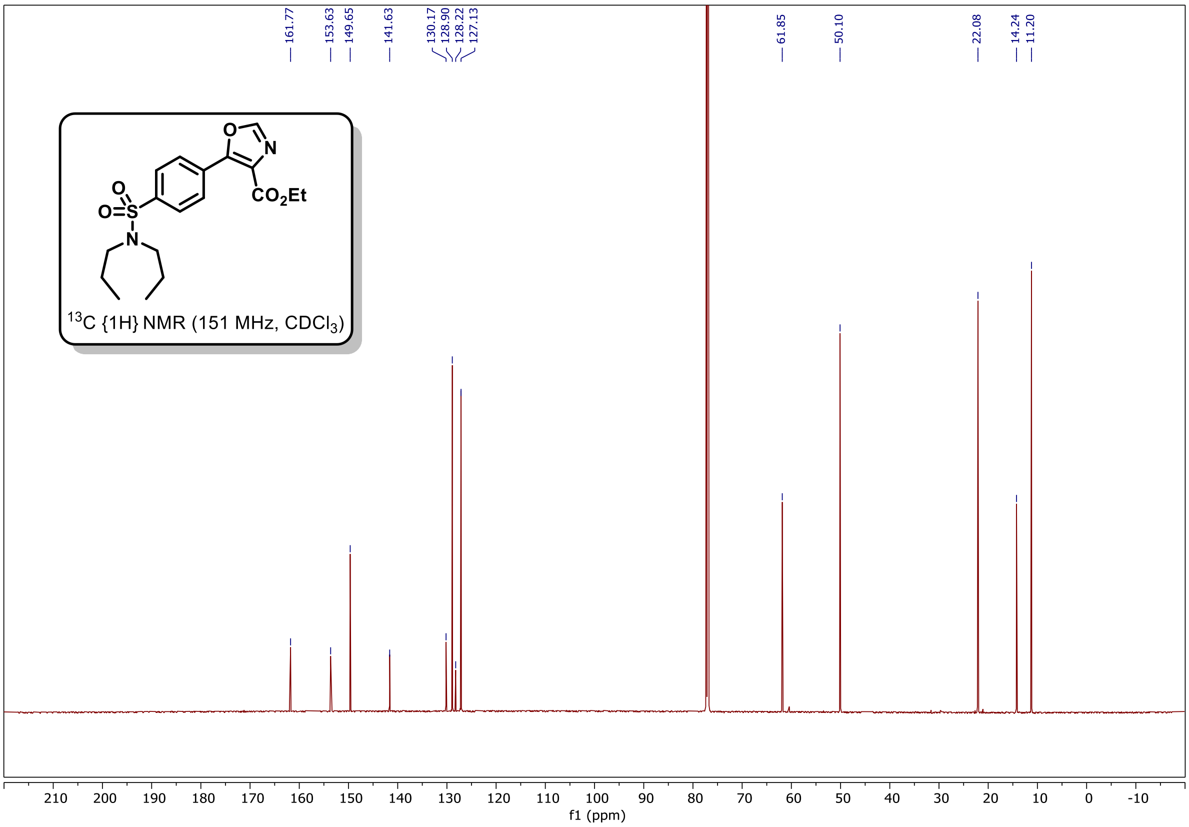

Crude **1H NMR** for **3wc**:

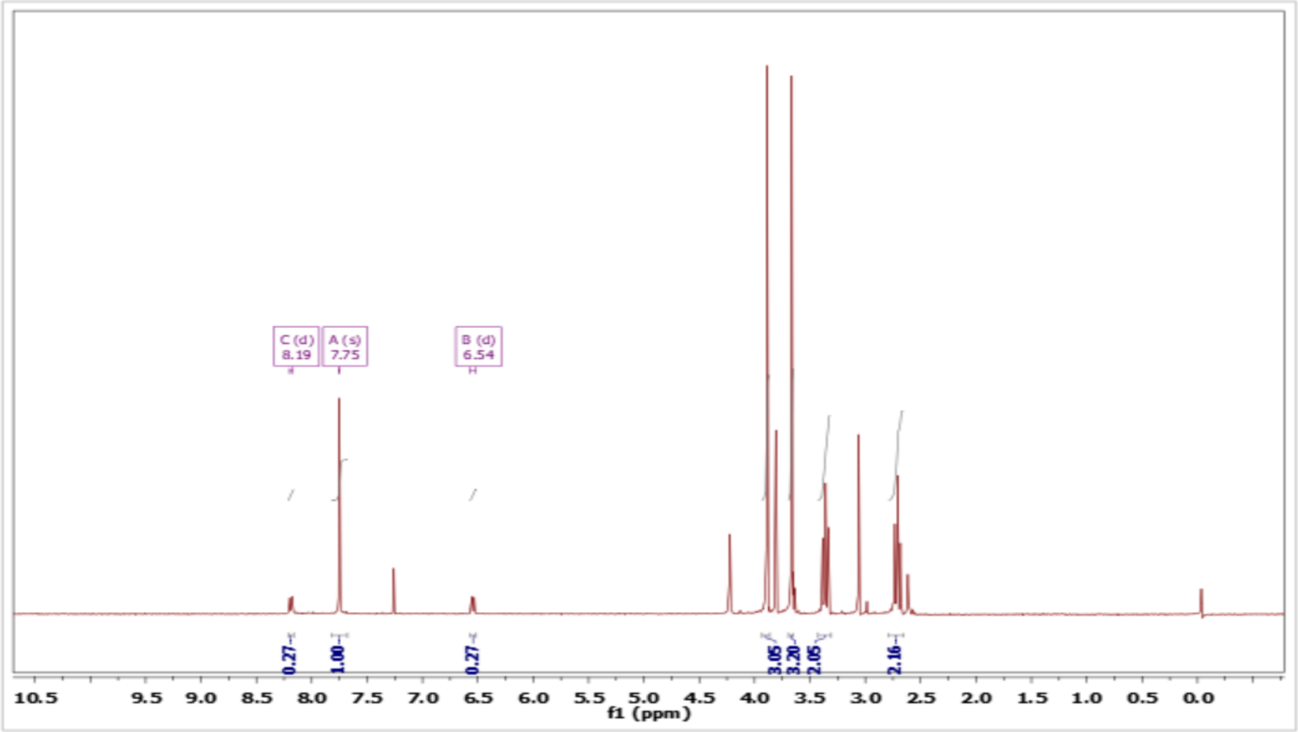

1H NMR for **3wc** after 1N HCl washing:

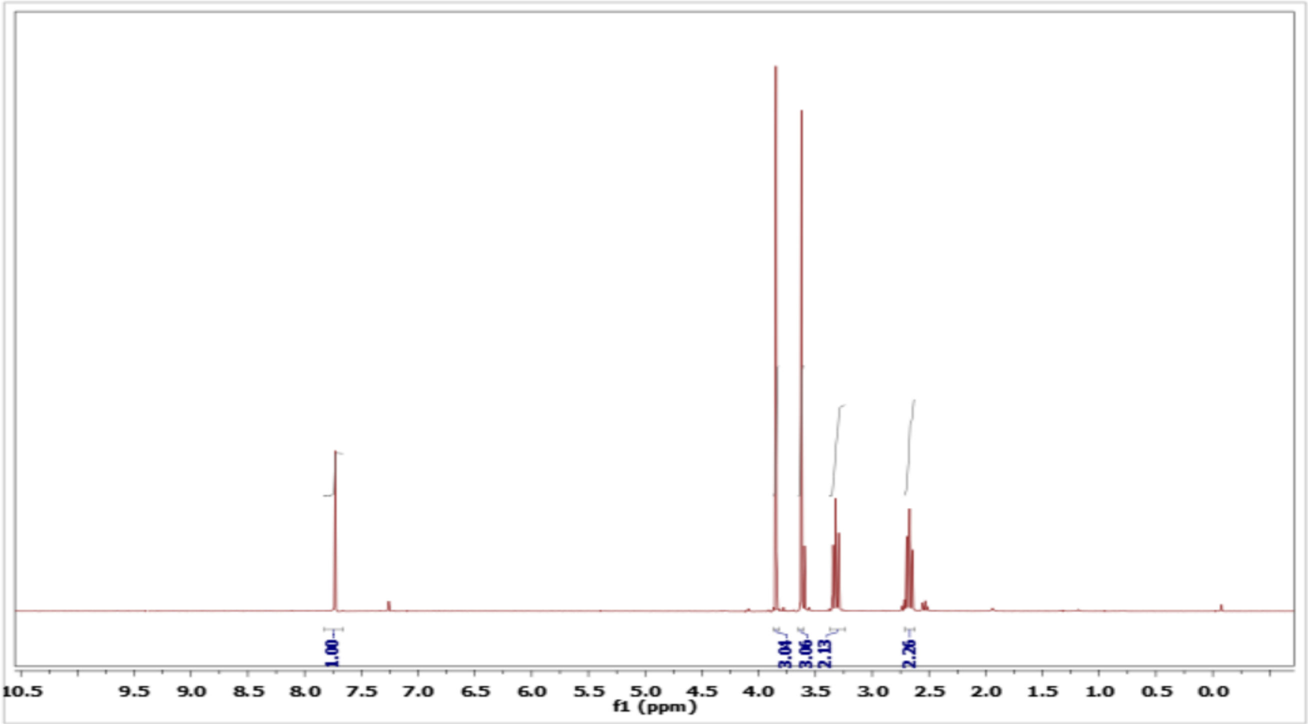

^{1}H NMR for **DMAP** after recrystallization:

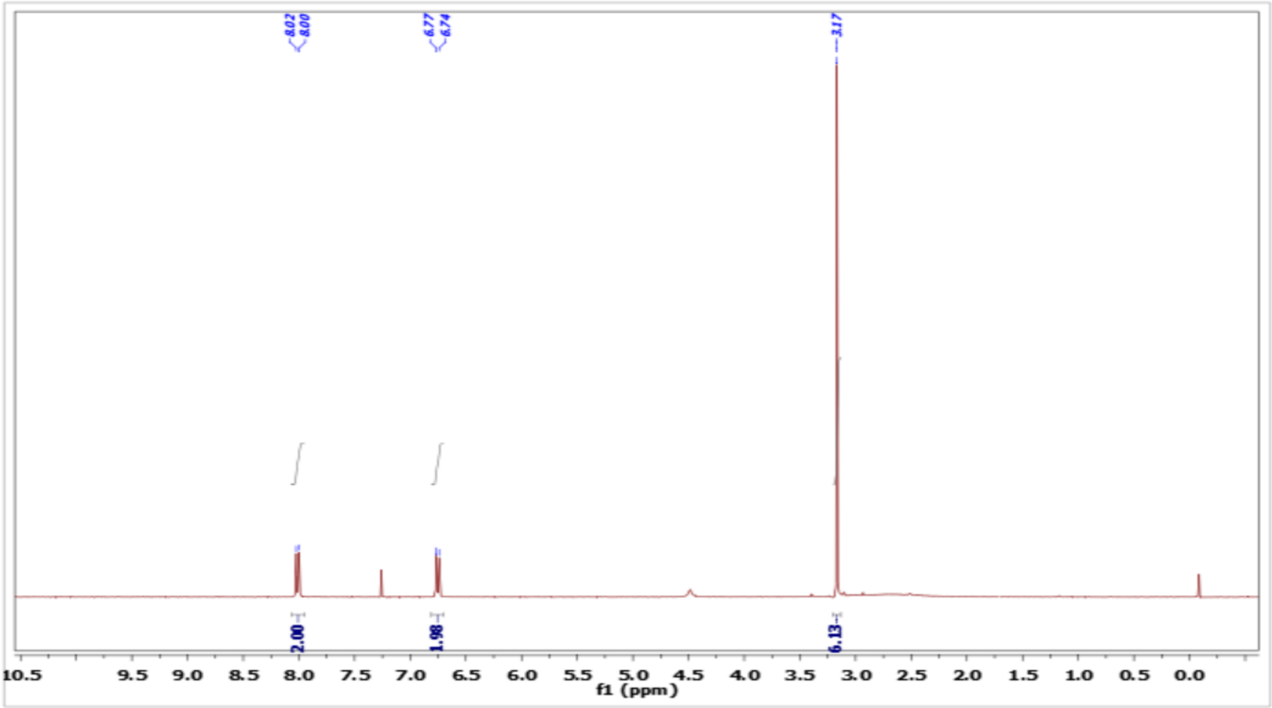

REFERENCES

(1) Ou, C.-H.; Pan, Y.-M.; Tang, H.-T. Electrochemically promoted *N*-heterocyclic carbene polymer-catalyzed cycloaddition of aldehyde with isocyanide acetate. *Science China*. **2022**, *65*, 1873-1878.

(2) Pavankumar, P.; Suraj, M. S.; Manjunatha, M. R.; Geeta, M. P.; Vivek, K. G.; Lohit, N.; Kalkhambkar, R. G. Synthesis, in vitro and theoretical studies on newly synthesized deep blue emitting 4-(p-methylphenylsulfonyl-5-aryl/alkyl)oxazole analogues for biological and optoelectronic applications. *J. Mol. Liq.* **2022**, *360*, 119520.

(3) Marcus, B.; Marcus B.; Ian R, B.; Ley, Steven, V.; Christoper, D. S.; Geoffrey, K. T. Fully Automated Continuous Flow Synthesis of 4,5-Disubstituted Oxazoles. *Org. Lett.* **2006**, *8*, 5231-5234.

(4) Kanchan, M.; Indrajit, D. *α*-Keto Thioesters as Building Blocks for Accessing *γ*-Hydroxybutenolides and Oxazoles. *Adv. Synth. Catal.* **2017**, *15*, 2692-2698.

(5) Anthony, A. E.; Jianwen, A. F.; Joseph, P. L.; Zachary K. S.; Fidalgo, J. D. V. Preparation of heteroarylaminopyrazole compounds as LRRK2 inhibitors and therapeutic uses thereof. *World Intellectual Property Organization*, WO2017087905 A1 **2017**-05-26.

(6) Pashikanti, G.; Chavan, L. N.; Liebeskind, L. S.; Goodman, M. M. Synthetic Efforts toward the Synthesis of a Fluorinated Analog of 5-Aminolevulinic Acid: Practical Synthesis of Racemic and Enantiomerically Defined 3-Fluoro-5-aminolevulinic Acid. *J. Org. Chem*. **2024**, *89*, 12176-12186.

(7) Tormyshev, V. M.; Tormyshev, V. M.; Mikhalina, T. V.; Rogozhnikova, O. Yu.; Troitskaya, T. I.; Trukhin, D. V. A combinatorially convenient version of synthesis of 5-substituted oxazole-4-carboxylic acid ethyl esters. *Russ. J. Org. Chem*. **2006,** *42*, 1031-1035.

(8) Zhang, X.; Yuan, Q.; Zhang, H.; Shen, Z. J.; Zhao, L.; Yang, C.; Guo, L.; Xia, W. Electrochemical synthesis of oxazoles *via* a phosphine-mediated deoxygenative [3 + 2] cycloaddition of carboxylic acids. *Green Chem.,* **2023**, *25*, 1435–1441.

(9) Talebizadeh, M.; Darehkordi, A.; Anary-Abbasinejad, M. A simple and efficient method for the synthesis of 4-tosyloxazoles from tosylmethyl isocyanide with α-ketoimidoyl chlorides. *J. Org. Chem*. **2018**, *5*,194-202.
